# Alpha-CaMKII deficiency causes immature dentate gyrus, a novel candidate endophenotype of psychiatric disorders

**DOI:** 10.1186/1756-6606-1-6

**Published:** 2008-09-10

**Authors:** Nobuyuki Yamasaki, Motoko Maekawa, Katsunori Kobayashi, Yasushi Kajii, Jun Maeda, Miho Soma, Keizo Takao, Koichi Tanda, Koji Ohira, Keiko Toyama, Kouji Kanzaki, Kohji Fukunaga, Yusuke Sudo, Hiroshi Ichinose, Masashi Ikeda, Nakao Iwata, Norio Ozaki, Hidenori Suzuki, Makoto Higuchi, Tetsuya Suhara, Shigeki Yuasa, Tsuyoshi Miyakawa

**Affiliations:** 1 Genetic Engineering and Functional Genomics Group, Frontier Technology Center, Kyoto University Graduate School of Medicine, Yoshida-Konoe-cho, Sakyo-ku, Kyoto, Japan; 2 Department of Psychiatry, Kyoto University Graduate School of Medicine, 54 Shogoin-Kawahara-cho, Sakyo-ku, Kyoto, Japan; 3 Japan Science and Technology Agency, CREST, Saitama, Japan; 4 Japan Science and Technology Agency, BIRD, Saitama, Japan; 5 Department of Ultrastructural Research, National Institute of Neuroscience, National Center of Neurology and Psychiatry, 4-1-1 Ogawa-higashi, Kodaira, Tokyo, Japan; 6 Department of Pharmacology, Nippon Medical School, 1-1-5 Sendagi, Bunkyo-ku, Tokyo, Japan; 7 Mitsubishi Tanabe Pharma Corporation, 1000 Kamoshida-cho, Aoba-ku, Yokohama, Japan; 8 Department of Molecular Neuroimaging, Molecular Imaging Center, National Institute of Radiological Sciences, 4-9-1 Anagawa, Inage-ku, Chiba, Japan; 9 Division of Systems Medicine, Institute for Comprehensive Medical Science, Fujita Health University, 1-98 Dengakugakubo, Kutsukake-cho, Toyoake, Japan; 10 Graduate School of Pharmaceutical Sciences, Tohoku University, Aramaki, Aoba-ku, Sendai, Japan; 11 Graduate School of Bioscience and Biotechnology, Tokyo Institute of Technology, 4259 Nagatsuta-cho, Midori-ku, Yokohama, Japan; 12 Graduate School of Medicine, Fujita Health University, 1-98 Dengakugakubo, Kutsukake-cho, Toyoake, Japan; 13 Graduate School of Medicine, Nagoya University, 65 Tsuruma-cho, Showa-ku, Nagoya, Japan; 14 Center for Genetic Analysis of Behavior, National Institute for Physiological Sciences, Myodaiji, Okazaki, Japan

## Abstract

Elucidating the neural and genetic factors underlying psychiatric illness is hampered by current methods of clinical diagnosis. The identification and investigation of clinical endophenotypes may be one solution, but represents a considerable challenge in human subjects. Here we report that mice heterozygous for a null mutation of the alpha-isoform of calcium/calmodulin-dependent protein kinase II (alpha-CaMKII+/-) have profoundly dysregulated behaviours and impaired neuronal development in the dentate gyrus (DG). The behavioral abnormalities include a severe working memory deficit and an exaggerated infradian rhythm, which are similar to symptoms seen in schizophrenia, bipolar mood disorder and other psychiatric disorders. Transcriptome analysis of the hippocampus of these mutants revealed that the expression levels of more than 2000 genes were significantly changed. Strikingly, among the 20 most downregulated genes, 5 had highly selective expression in the DG. Whereas BrdU incorporated cells in the mutant mouse DG was increased by more than 50 percent, the number of mature neurons in the DG was dramatically decreased. Morphological and physiological features of the DG neurons in the mutants were strikingly similar to those of immature DG neurons in normal rodents. Moreover, c-Fos expression in the DG after electric footshock was almost completely and selectively abolished in the mutants. Statistical clustering of human post-mortem brains using 10 genes differentially-expressed in the mutant mice were used to classify individuals into two clusters, one of which contained 16 of 18 schizophrenic patients. Nearly half of the differentially-expressed probes in the schizophrenia-enriched cluster encoded genes that are involved in neurogenesis or in neuronal migration/maturation, including calbindin, a marker for mature DG neurons. Based on these results, we propose that an "immature DG" in adulthood might induce alterations in behavior and serve as a promising candidate endophenotype of schizophrenia and other human psychiatric disorders.

## Background

**Figure 1:**
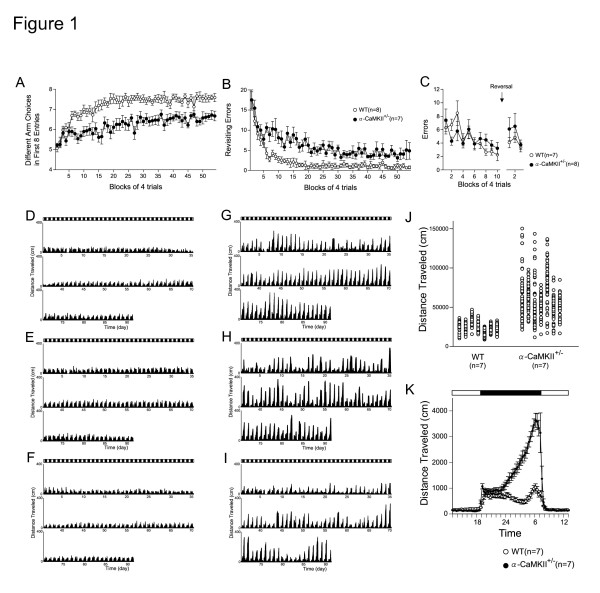
**Dysregulated Behaviors of Alpha-CaMKII+/- Mice**. (A, B) In the spatial working memory version of the eight-arm radial maze, the alpha-CaMKII+/- mice performed significantly worse than control mice with respect to the number of different arm choices in the first eight entries (P = 0.0022) and made significantly more revisiting errors than controls (P = 0.0026). (C) Mutant mice had normal performance in reference memory tasks in the eight-arm radial maze (P = 0.6394). Activity level of control mice (D-F) and alpha-CaMKII+/- mice (G-I) in their home cage. Mutant mice were hyperactive and showed a periodic mood-change-like activity pattern. (J) The variability in the activity in the home cage during the dark period was greater in mutant mice (coefficient of variation, P = 0.0088, Mann-Whitney U test). (K) The activity of mutant mice increased throughout the dark period (genotype effect, P < 0.0001, interaction between genotype and time, P < 0.0001, repeated measures ANOVA). Error bars indicate s.e.m.

Elucidating the neural and genetic factors underlying psychiatric illness is hampered by the current methods of clinical diagnosis [1]. The identification and investigation of clinical endophenotypes might be one solution [2], but represents a considerable challenge in human subjects. Therefore, establishing animal models of psychiatric disorders is essential for understanding the pathogenesis/pathophysiology of the disorders [3-6]. Previously, we reported that forebrain-specific calcineurin (CN) knockout mice have severe working/episodic-like memory deficits [7], and exhibit multiple abnormal behaviors related to schizophrenia [8]. Schizophrenia is significantly associated with a variation in the 8p21.3 gene, *PPP3CC*, which encodes the CNA gamma subunit of calcineurin [9-11]. Based on these findings, we speculated that we could efficiently obtain a mouse model of psychiatric disorders by applying a comprehensive behavioral test battery [12] to various strains of mice bearing mutations of the genes encoding the molecules involved in CN signaling pathways or CN related neural mechanisms [13]. We assessed seven different strains of mutant mice: mice lacking type 3 isoform ryanodine receptor, neuronal nitric oxide synthase, adenomatous polyposis coli, calcium/calmodulin-dependent protein kinase IV, pituitary adenylate cyclase-activated polypeptide, nuclear factor of activated T cells c2/c3/c4 [14] or alpha-isoform of calcium/calmodulin-dependent protein kinase II (alpha-CaMKII). Four strains exhibited increased locomotor activity, and three strains exhibited abnormal social behavior (Miyakawa, unpublished observations). Among them, the only mutant mouse strain that exhibited a significant working memory deficit, a proposed functional endophenotype of schizophrenia and other psychiatric disorders [15], was heterozygous for a null mutation of the alpha-isoform of CaMKII (alpha-CaMKII+/-) (Figure 1A and 1B). CaMKII is a ubiquitous serine/threonine protein kinase that is abundant in the brain (up to 2% of the total protein); a holoenzyme that consists of four isozymes (α, β, γ, δ); phosphorylates protein substrates, such as AMPA receptors, synapsin I, tyrosine hydroxylase, L-type Ca^2+ ^channels, and MAP-2, and itself by autophosphorylation; and is important for long-term potentiation, synaptic plasticity, and memory formation [16-18]. CaMKII is situated downstream of CN in a model [19].

Here we report that alpha-CaMKII+/- mice have profoundly dysregulated behaviors and impaired neuronal development in the DG. The behavioral abnormalities include a severe working memory deficit and an exaggerated infradian rhythm, which are similar to symptoms seen in schizophrenia and other psychiatric disorders. Transcriptome analysis of the hippocampus of these mutants revealed that the expression levels of more than 2000 genes were significantly changed. Strikingly, among the 20 most downregulated genes, 5 had highly selective expression in the DG. Whereas BrdU incorporated cells in the mutant mouse DG was increased by more than 50 percent, the number of mature neurons in the DG was dramatically decreased. Morphological and physiological features of the DG neurons in the mutants were strikingly similar to those of immature DG neurons in normal rodents. Statistical clustering of human post-mortem brains using 10 genes differentially-expressed in the mutant mice were used to classify individuals into two clusters, one of which contained 16 of 18 schizophrenic patients. Nearly half of the differentially-expressed probes in the schizophrenia-enriched cluster encoded genes that are involved in neurogenesis or in neuronal migration/maturation. Based on these results, we propose that an "immature DG" in adulthood might induce alterations in behavior and serve as a promising candidate endophenotype of human psychiatric disorders.

## Results

### Severe working memory deficits and exaggerated infradian rhythm in alpha-CaMKII+/- mice

Alpha-CaMKII+/- mice have decreased anxiety-like behavior, increased aggressive behavior [20], and deficits in long-term memory and the establishment of permanent memories [21,22]. Consistent with a previous report [20], we found that alpha-CaMKII+/- mice also exhibit high levels of aggression towards cage mates: By the age of 3 mo, more than half of their cage mates, both wild-type controls and mutants, were killed by the mutants. While severe working memory deficits were observed in both in the eight-arm radial maze task (Figure 1A and 1B) and delayed alternation task using a modified T-maze (data not shown), their performance in reference memory tasks using the same apparatuses were normal (Figure 1C). In addition to their severe cognitive deficits, these mutants showed an array of other profoundly dysregulated behaviors. The behavioral phenotypes of these mice include increased locomotor activity in novel situations, decreased anxiety-like behavior, and decreased depression-like behavior (see Additional file 1, Figure S1). Normal mice have a constant activity pattern in their home cage (Figure 1D–1F). The pattern of locomotor activity of the mutants, however, changed over time (Figure 1G–1I). They exhibited periodic mood-change-like behavior in their home cage. One cycle was approximately 1 to 2 wk. The mutants had more variable activity patterns in the total distance traveled in the dark period in their home cages (Figure 1J). The locomotor activity pattern of the mutants during a single day was also remarkably different from that of control mice. While wild-type mice generally had two peaks in activity during the dark phase, the activity of the mutant mice steadily increased until morning (Figure 1K).

### Selective changes in dentate gyrus of alpha-CaMKII+/- mice

**Figure 2:**
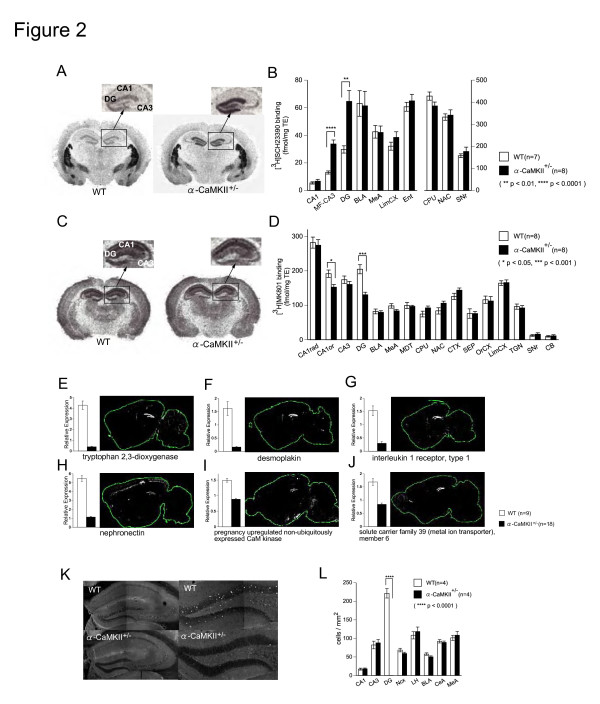
**Specific Abnormalities in the DG of Alpha-CaMKII+/- Mice**. (A, B) Dopamine D1 receptor binding was highly increased in the DG of alpha-CaMKII+/- mice. (C, D) NMDA receptor binding was similarly downregulated in the DG and CA1 of alpha-CaMKII+/- mice. (E-J) Among the downregulated genes in the hippocampus of alpha-CaMKII+/- mice, several genes were expressed selectively in the DG (Allen Brain Atlas [23] [Internet]. Seattle, WA: Allen Institute for Brain Science. © 2004–2008. Available from: .). Graphs indicate the relative expression levels of each gene in the microarray experiment, which are confirmed by quantitative RT-PCR (see Additional file 1, Figure S6). (K, L) c-Fos expression following electric foot shock was selectively decreased in the DG of mutant mice at the age of 8 weeks. BLA, basolateral amygdaloid nucleus; MeA, medial amygdaloid nucleus; LimCX, limbic cortex; Ent, entorhinal cortex; CPU, caudate nucleus/putamen; NAC, nucleus accumbens; SNr, substantia nigra pars reticulata; CA1rad, stratum radiatum of CA1; CA1or, stratum oriens of CA1; MDT, mediodorsal thalamic nucleus; CTX, cingulated cortex; SEP, septum; OrCX, orbital cortex; TGN, tegmental nuclei; CB, cerebellum; Ncx, neocortex; LH, lateral hypothalamus; CeA, central amygdaloid nucleus. * P < 0.05, ** P < 0.01, *** P < 0.001, **** P < 0.0001. Error bars indicate s.e.m.

**Figure 3:**
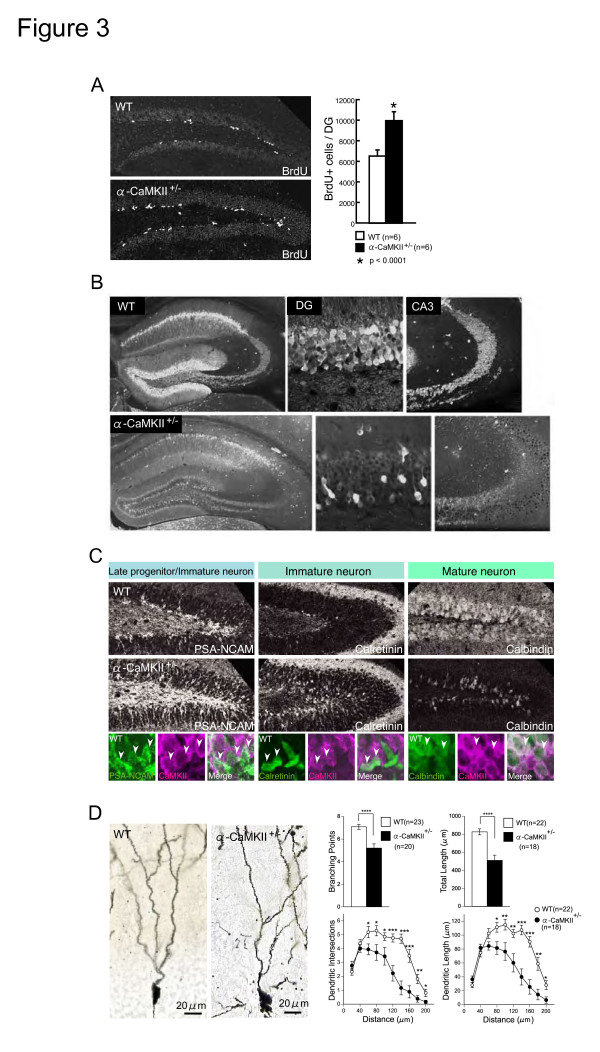
**Immature DG of Alpha-CaMKII+/- Mice**. (A) The number of BrdU-positive cells in the subgranular zone of the DG is increased in the alpha-CaMKII +/- mice at 7 weeks old, as observed 1 d after BrdU administration. The number of BrdU-positive cells within the DG was 52.8% higher (p = 0.00001) in the alpha-CaMKII +/- mice than in the wild-type mice. (B) In alpha-CaMKII+/- mice, the number of cells expressing calbindin (a mature-neuron marker) was decreased. (C) In alpha-CaMKII+/- mice, the number of cells expressing PSA-NCAM (a late-progenitor and immature-neuron marker) and calretinin (an immature-neuron marker) was markedly increased. CaMKII was co-expressed in a subset of PSA-NCAM-, calretinin-, and calbindin-expressing cells, indicated by the white arrowheads. For the analysis of expression of markers related to neurogenesis and differentiation, at least five mice were examined in each group. (D) Rapid Golgi staining showed that the number of Golgi-impregnated cells was decreased in the DG of alpha-CaMKII+/- mice (see Additional file 1, Figure S9) and that Golgi-impregnated cells in the DG of alpha-CaMKII+/- mice had less branching and shorter dendrites. * P < 0.05, ** P < 0.01, *** P < 0.001, **** P < 0.0001. Error bars indicate s.e.m.

These marked changes in behavior led us to examine the brains of the alpha-CaMKII mutants. In the mutants, the amount of alpha CaMKII was decreased by 20% to 60% in the cingulate cortex, amygdala, and hippocampus, and the amount of phosphorylated beta-CaMKII was increased in the hippocampus, which is probably a compensatory effect. The amounts of proteins, such as CN and phosphorylated extracellular signal-regulated kinase, were also altered (see Additional file 1, Figure S2). There were no dramatic differences in monoamine content in any region investigated, except for an increase in striatal dopamine turnover in the mutant mice (see Additional file 1, Figure S3). Next, we analyzed the transcriptome of the hippocampus, a region that has an essential role in working memory and locomotor activity in rodents, of alpha-CaMKII+/- mice, by using Affymetrix GeneChips. There were more than 2000 genes that were significantly up- or downregulated (see Additional file 2, Table S1 and S2). There was a more than two-fold increase in the gene expression levels of dopamine D1A receptors, therefore we performed autoradiography studies for five major neurotransmitter receptors and two transporters (see Additional file 1, Figure S4 and S5). Consistent with the microarray results, dopamine D1-like receptor binding was dramatically and selectively increased in the DG of alpha-CaMKII+/- mice (Figure 2A and 2B). N-methyl-D-aspartate (NMDA) receptor binding was downregulated in the hippocampus, especially in the DG (Figure 2C and 2D). Based on the highly selective changes in receptor binding, we examined the location of genes whose expression levels were changed in the mutant brain. A database search using Allen Brain Atlas [23] revealed that, among the 20 most downregulated genes in the alpha-CaMKII +/- hippocampus, 5 genes (*DSP*, *TDO2*, *NPNT*, *IL1R1*, and *PNCK*) had highly selective expression in the DG (Figure 2E–2J, and Additional file 1, Figure S6). Moreover, c-Fos expression in the DG 2 h after electric shock was almost completely and selectively abolished in alpha-CaMKII+/- mice, which suggests that their DG was functionally downregulated (Figure 2K, 2L and Additional file 1, Figure S7). These findings in the DG, a region in which 3000 to 4000 neurons are born each day in rodents [24], led us to examine the cell proliferation in the mutant mouse DG. BrdU-labeled cells were dramatically increased by 53% in the mutants (Figure 3A). Consistent with this finding, many of the genes involved in the brain derived neurotrophic factor – mitogen-activated protein kinase (BDNF-MAPK) pathway, which has an important role in neurogenesis [25], were significantly upregulated or downregulated in the hippocampus of the alpha-CaMKII+/- mice, including a 32 to 35% up-regulation of BDNF probes (see Additional file 1, Figure S8). Dysregulation of the expression of BDNF-MAPK pathway genes could be related to the increased cell proliferation in the mutants.

### The dentate gyrus of alpha-CaMKII+/- mice is immature

**Figure 4:**
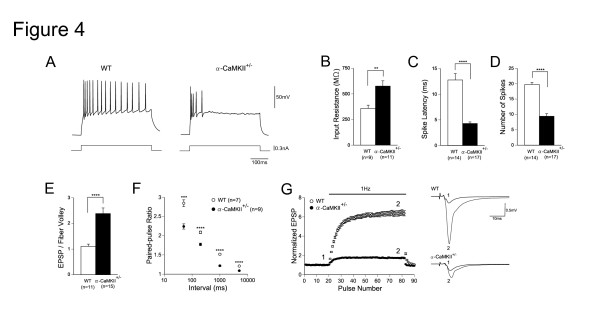
**Altered Granule Cell Excitability and Mossy Fiber Synaptic Transmission in Alpha-CaMKII+/- Mice**. (A) Examples of action potential firing of granule cells in wild-type and mutant mice evoked by steady depolarizing currents (320 pA, 400 ms). (B) Increased input resistance in mutant mice (P = 0.0029). (C) Reduced spike latency in mutant mice (P < 0.0001). Depolarizing currents (320 pA) were injected into granule cells and the latency of the first action potential was measured. (D) The maximal number of spikes evoked by 400 ms depolarizing currents was decreased in mutant mice (P < 0.0001). (E) The efficacy of basal transmission at the mossy fiber synapse was increased in mutant mice (P < 0.0001). The ratio of the peak EPSP amplitude to fiber volley amplitude is shown. (F) Reduced paired-pulse facilitation ratios of EPSPs at intervals ranging from 50 to 5000 ms in mutant mice. (G) Mutant mice had greatly reduced frequency facilitation at 1 Hz. Sample EPSPs were recorded at the time indicated by the numbers in the graph. Error bars indicate s.e.m.

There are distinct stages in adult neurogenesis in the DG. Each stage has a few markers and specific morphological and physiological properties [24,26]. The number of cells expressing Polysialic acid-NCAM (PSA-NCAM), a marker for late-stage progenitor cells and immature neurons, and calretinin, a marker for immature neurons, was dramatically increased in the DG of the mutant mice. In normal mice, after granule cells migrate into the granule cell layer, PSA-NCAM positive cells were hardly detected in the granule cell layer. In the mutants, however, there were many PSA-NCAM positive cells in the granule cell layer. Moreover, the amount of calbindin, a marker for mature neurons in the DG, -positive cells was dramatically reduced (Figure 3B and 3C). Thus, in the mutants, the number of immature neurons is increased and the number of mature neurons is decreased (in this report, "immature" means that the control of the precursor cell proliferation and the functional differentiation of the newly born cells were hampered.). Furthermore, rapid Golgi staining in the mutant DG showed that dendritic branching and length were decreased, which are the morphological properties of immature neurons (Figure 3D and Additional file 1, Figure S9). Consistent with these immunohistochemical and morphological findings, the neurons in mutant DG had electrophysiological features that are characteristic of immature DG neurons [27-29], including high input resistance, high excitability, small spike amplitude and a decreased number of spikes during sustained depolarization (Figure 4A–4D, and Additional file 1, Figure S10). Moreover, the mutants had extremely abnormal transmission at the synapses between granule cell axons, mossy fibers (MFs), and the CA3 pyramidal cells. That is, basal transmission was increased and large facilitation characteristic of the MF synapse was markedly decreased in mutants (Figure 4E–4G, and Additional file 1, Figure S10). The synaptic facilitation is generally known to be mediated by presynaptic mechanisms. Since the frequency facilitation at the MF synapse is very small at the early postnatal developmental stages in normal mice [30], the greatly reduced MF facilitation in the mutant mice lends further support to the hypothesis that the mutant granule cells in alpha-CaMKII+/- mice are immature. At the ultra structural level, the MF-CA3 synapses were poorly developed in the hippocampus of the mutant mice (see Additional file 1, Figure S11), which could be related to the abnormal transmission at the synapse. Collectively, results of analyses using molecular markers, morphological assessment using Golgi staining, and electrophysiological analysis using whole cell patch clamp all indicated that the vast majority of the mutant DG granule cells failed to develop into mature neurons. The qualitative difference in the DG granule cell developmental stage accompanied by the severely impaired neuronal functions might also account for the high number of genes that were differentially expressed in the mutant hippocampus.

### Human post-mortem brain analysis using the biomarkers derived from alpha-CaMKII+/- mice

**Figure 5:**
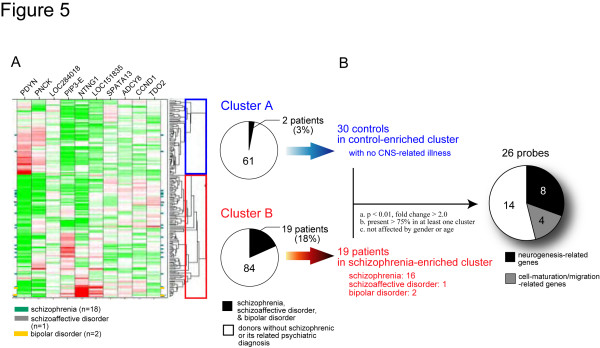
**Clustering of Human Post-Mortem Brains by Biomarkers Derived from Alpha-CaMKII+/- Mice**. (A) We selected 10 genes (*ADCY8*, *CCND1*, *LOC151835*, *LOC284018*, *NTNG1*, *PDYN*, *PIP3-E*, *PNCK*, *SPATA13 *and *TDO2*), which were differentially expressed in the mutant hippocampus, as a set of biomarkers to characterize the mutants, and performed the statistical clustering of the expression data of these 10 genes in 166 hippocampi of human post-mortem brains. The cluster analysis classified the subjects into two clusters (Cluster A and Cluster B), and Cluster B contained significantly more schizophrenic, schizoaffective and bipolar patients than Cluster A (P = 0.0041, χ^2 ^test). (B) We compared the gene expression profile between the schizophrenic patients in Cluster B (= schizophrenia-enriched cluster) (n = 19) and the controls with no major psychiatric diagnosis and no CNS-related illness in Cluster A (= control-enriched cluster) (n = 30), using the following criteria; (i) gene expression level was significantly different between two groups (p < 0.01, fold change > 2.0), (ii) present flags were marked in over 75% of hippocampi in at least one of the two groups, (iii) gene expression was not affected by age and gender. We found that 26 probes met these criteria and nearly half of them encoded genes which are known to be involved in neurogenesis or cell-migration/maturation.

To assess if our findings derived from the mutant mice could be related to human psychiatric disorders, comprehensive gene expression data of human post-mortem brains was analyzed using a specific biomarker set derived from alpha-CaMKII+/- mice. In this study, we selected 10 genes (*ADCY8*, *CCND1*, *LOC151835*, *LOC284018*, *NTNG1*, *PDYN*, *PIP3-E*, *PNCK*, *SPATA13*, and *TDO2*), which were differentially expressed in the mutant hippocampus, as a set of biomarkers to characterize the mutants (see Additional file 1, Figure S12). Statistical clustering of the expression data of these 10 genes in 166 hippocampi of human post-mortem brains was performed. The analysis classified the subjects into two clusters; one cluster contained 16 of 18 schizophrenic patients and 1 schizoaffective and 2 bipolar patients (Figure 5A). Further, we compared the gene expression profile between the schizophrenic patients in the schizophrenia-enriched cluster and the controls with no major psychiatric diagnosis in the control-enriched cluster, and found 26 differentially-expressed probes in the schizophrenic patients. Almost half of these differentially-expressed genes are involved in neurogenesis or neuronal migration/maturation, including calbindin, a maker for mature neurons in DG (Figure 5B, Table 1, Additional file 1, Figure S12 and S13, and Additional file 2, Table S3 and S4). These results indicate that schizophrenia subgroup could be classified using the biomarkers derived from the alpha-CaMKII+/- mice, and that the functional alterations in the hippocampus could be a potential intermediate phenotype for this schizophrenia subgroup, consistent with proposals that the hippocampus is central to the pathophysiology of schizophrenia [31,32].

**Table 1:** Differentially Expressed Probes in the Schizophrenic Patients of the Schizophrenia-enriched Cluster.

**Expression**	**Probe ID**	**Gene Name**	**p-value (t-Test)**	**FC Signed Magnitude**	**Gene Symbol**
*UP*	*213920_at*	*Cut-like 2 (Drosophila)*	*6.38E-06*	*2.77*	*CUTL2*
UP	215532_x_at	Zinc finger protein 492	1.30E-06	2.44	ZNF492
UP	214735_at	Phosphoinositide-binding protein PIP3-E	1.19E-04	2.4	PIP3-E
UP	220232_at	Stearoyl-CoA desaturase 4, biosynthesis of monounsaturated fatty acids from saturated fatty acids	2.35E-05	2.34	SCD4
UP	204730_at	Regulating synaptic membrane exocytosis 3 (RIMS3), Ca2+-binding C2 domain	2.02E-04	2.22	RIMS3
**UP**	**216153_x_at**	**Reversion-inducing-cysteine-rich protein with kazal motifs**	**1.92E-04**	**2.21**	**RECK**
*UP*	*216511_s_at*	*Transcription factor 7-like 2 (T-cell specific, HMG-box) (T-cellspecific transcription factor 4)*	*1.04E-03*	*2.19*	*TCF7L2(TCF4)*
UP	215182_x_at	mRNA; cDNA DKFZp586E121 (from clone DKFZp586E121)	9.75E-04	2.12	unknown
*UP*	*216037_x_at*	*Transcription factor 7-like 2 (T-cell specific, HMG-box) (T-cellspecific transcription factor 4)*	*9.49E-05*	*2.04*	*TCF7L2(TCF4)*
UP	215801_at	mRNA; cDNA DKFZp434G1615 (from clone DKFZp434G1615)	7.57E-05	2.01	unknown
UP	222249_at	KIAA1651 protein	1.03E-03	2.01	unknown

**DOWN**	**209687_at**	**Chemokine (C-X-C motif) ligand 12 (stromal cell-derived factor 1)**	**2.79E-03**	**-2.01**	**CXCL12**
DOWN	206662_at	Glutaredoxin (thioltransferase)	2.70E-04	-2.04	GLRX
DOWN	207768_at	Early growth response 4	8.53E-03	-2.06	EGR4
*DOWN*	*208650_s_at*	*CD24 antigen (small cell lung carcinoma cluster 4 antigen)*	*3.43E-04*	*-2.21*	*CD24*
DOWN	203235_at	Thimet oligopeptidase 1	3.05E-03	-2.25	THOP1
DOWN	214432_at	ATPase, Na+/K+ transporting, alpha 3 polypeptide	2.43E-04	-2.28	ATP1A3
DOWN	202802_at	Deoxyhypusine synthase	3.39E-03	-2.34	DHPS
**DOWN**	**205626_s_at**	**Calbindin 1, 28 kDa**	**5.36E-05**	**-2.46**	**CALB1**
DOWN	219389_at	Hypothetical protein FLJ10052	6.06E-05	-2.71	FLJ10052
*DOWN*	*266_s_at*	*CD24 antigen (small cell lung carcinoma cluster 4 antigen)*	*3.91E-04*	*-2.75*	*CD24*
DOWN	207307_at	5-hydroxytryptamine(serotonin) receptor 2C	1.49E-03	-2.82	HTR2C
*DOWN*	*208651_x_at*	*CD24 antigen (small cell lung carcinoma cluster 4 antigen)*	*4.25E-05*	*-2.92*	*CD24*
**DOWN**	**206935_at**	**Protocadherin 8**	**1.46E-06**	**-2.92**	**PCDH8**
*DOWN*	*216379_x_at*	*CD24 antigen (small cell lung carcinoma cluster 4 antigen)*	*4.17E-06*	*-2.97*	*CD24*
*DOWN*	*209771_x_at*	*CD24 antigen (small cell lung carcinoma cluster 4 antigen)*	*2.30E-05*	*-3.23*	*CD24*

Total 26 probes were determined as highly significantly affected independently from age and gender in schizophrenic patients of their specific cluster. Neurogenesis-related genes are shown in italics and cell maturation/migration-related genes are in bold. Note that calbindin, a maker for mature neurons in DG, is significantly downregulated in the schizophrenic patients.

## Discussion

In this study, alpha-CaMKII+/- mice had dysregulated behaviors, such as increased locomotor activity, a severe working memory deficit, and exaggerated infradian rhythm. Transcriptome analysis and comprehensive autoradiography studies indicated that the mutants had marked abnormalities in gene expression and receptor binding in the hippocampus, specifically in the DG. Results of analyses using molecular markers, morphological assessment using Golgi staining, and electrophysiological analysis using the whole cell patch clamp technique were consistent with the idea that most of the neurons in the DG of the mutant mice failed to develop to maturity, which likely caused the functional impairment of the granule cells. To our knowledge, this is the first in vivo study showing that an immature portion of a brain can be embedded in an adult brain of animals. Furthermore, our transcriptome analysis of human post-mortem brain revealed that biomarkers derived from mutant mice could be used to classify humans into two clusters, one of which had a higher susceptibility to schizophrenia and differentially expressed genes related to neural development, including calbindin, a marker for mature DG neurons. These findings suggest that the "immature DG" or its equivalent is potentially common internal brain phenotype reflecting or affecting behavioral traits and susceptibility to psychiatric disorders.

These findings raise the question of how alpha-CaMKII deficiency causes immature DG phenotype. CaMKII is implicated in dendrite morphogenesis and stabilization [33-36]. Based on the fact that the DG phenotype of the mutants is accompanied by changes in the expression of many genes, it is reasonable to speculate that alpha-CaMKII contributes to regulate the gene expression required for the maturation of DG neurons. The CaMKII-NeuroD pathway specifies dendritic morphogenesis in primary granule neurons in cerebellar slices [34]; CaMKII phosphorylates a transcription factor NeuroD at Ser336 in an activity-dependent manner and thereby orchestrates a program of gene expression and stimulates dendritic growth. Although the brain region investigated by Gaudilliere et al. was not the DG, considering the high expression of NeuroD in the hippocampus [37], it is possible that the impaired CaMKII-NeuroD pathway underlies the abnormality of DG maturation of the mutants. Also, Greenberg and colleagues reported that activity-dependent phosphorylation of MeCP2 at serine 421 is mediated by CaMKII and phosphorylation of the site controls the ability of MeCP2 to regulate activity-dependent BDNF transcription and dendritic growth [35], raising the possibility that the immature DG of the mutant mice is due to a disruption of the program that regulates gene expression triggered by this pathway. The changes in expression of many genes involved in the BDNF-MAPK pathway in the hippocampus of the mutant mice (see Additional file 1, Figure S8) is of interest in relation to their findings. Degradation of scaffold protein Liprina1 by alpha-CaMKII regulates leukocyte common antigen-related receptor tyrosine phosphatase distribution and dendrite development [36]. Impairment of this mechanism could provide an alternative or additional candidate for the development of an immature DG in the mutants. Further studies are required to specify the mechanisms responsible for the immaturity of the DG neurons of the mutants as well as the factors making the DG particularly sensitive to the amount of CaMKII compared to other brain areas.

The DG neurons in the mutants also had remarkable functional abnormalities, including higher input resistance, slightly depolarized resting membrane potentials, high excitability, decreased number of spikes during sustained depolarization, and greatly reduced facilitation at MF-CA3 synapses. The morphological immaturity, that is, the reduced dendritic arborization, is consistent with the higher input resistance of the mutant DG neurons [29]. Although the other electrophysiological properties are hard to explain based solely on the morphological immaturity, they are very similar to those of immature DG neurons of normal adult animals [27] or neurons in the early postnatal period [30]. Those unique physiological properties might be explained by some of the differentially expressed channels/receptors and their functional modulators found in our gene chip analysis and binding assays but, given the changes in expression of more than 2000 genes, identifying the actual determinants of those properties requires understanding the neuron as a complex system regulated by a network of a number of molecules, and not just a single pathway.

The selective and all-or-none type reduction of c-Fos expression in the DG after behavioral stimulation is likely to reflect the lower ability of the neurons to keep firing after stimulation in the mutant mice. It is easy to imagine that such extreme functional abnormalities of firing properties of neurons, together with marked impairment of synaptic facilitation at MF-CA3 synapses, change the DG function in a manner qualitatively different from normal DG, which would subsequently cause abnormal DG-dependent behavior. Because the alpha-CaMKII+/- mice we used are conventional global knockout mice, it is technically difficult to determine the specific brain region that caused each behavioral abnormality. The dramatic and specific abnormalities in the DG, as assessed using several different assays, however, strongly suggest that at least some of the behavioral deficits are due to the "immature DG". Indeed, the DG has an important role in locomotor activity [38] and the formation of spatial working memory [38,39], and adult neurogenesis in the hippocampus is related to anxiety-related behavior [40], voluntary exercise [41], and memory formation [42]. Also, it is possible that DG dysfunction during development could cause secondary functional alterations in other areas in the brain. Early neonatal hippocampal inactivation alters the development and plasticity of prefrontal cortical circuitry and produces a constellation of behavioral and cellular changes that mimic many aspects of schizophrenia [43-45]. Alpha-CaMKII is expressed postnatally and dramatically increases from day 5 after birth [46,47]. Postnatal alterations in DG maturation might trigger changes in intracortical connections, which subsequently cause some of the behavioral phenotypes exhibited by the mutants.

Many of the behavioral abnormalities in alpha-CaMKII+/- mice are similar to those observed in patients with psychiatric disorders; impairment in working memory is a proposed schizophrenia endophenotype [15], and exaggerated infradian rhythm is a prominent feature of bipolar disorder [48]. Based on these findings, we performed an association analysis of alpha-CaMKII genes with schizophrenia and bipolar disorder. Although there were trends in the associations of several haplotype-tagging single nucleotide polymorphisms with the diseases, our study did not support a major contribution of a genetic mutation of alpha-CaMKII in the susceptibility to either bipolar disorder or schizophrenia in a Japanese population (see Additional file 1, Figure S14, and Additional file 2, Table S5). However, recently, Weinberger's group reported that a polymorphism in the gene for alpha-CaMKII is associated with a risk for schizophrenia and that this single nucleotide polymorphism modulates working memory function even in healthy young controls carrying the schizophrenia-associated risk allele [49]. Considering the fact that only a 50% reduction of alpha-CaMKII mRNA caused such a dramatic and qualitative change of the nature of the DG neurons, any genetic or environmental alterations affecting CaMKII signaling pathways could result in a similar change or similar endophenotypes. For example, the NMDA receptor is a major upstream molecule of CaMKII, and its hypofunction, which is implicated in schizophrenia pathophysiology [50], could result in endophenotypes similar to immature DG observed in alpha-CaMKII+/- mice. Schizophrenia, bipolar disorder, and other related psychiatric disorders are considered to be biologically heterogeneous populations, due to the limitation of the current methods of psychiatric diagnosis. Thus an endophenotype-based analysis would be preferable for establishing a biological underpinning for the classification of psychiatric disorders, rather than an analysis based on the current diagnostic methods [2,6,51].

Statistical clustering of human post-mortem hippocampal samples using the genes/biomarkers derived from the study of alpha-CaMKII+/- mice revealed that human can be classified into two groups, one of which seems to have higher susceptibility to schizophrenia. The two groups differ in their expression of genes related to neurogenesis or neuronal migration/maturation. Though the exact nature of the differences in these two groups is unknown, it is likely that such a difference in gene expression could reflect the differences in development and functions of the DG. In particular, the approximately 60% reduction of calbindin, a marker for mature neurons in DG, in the schizo-enriched cluster is of special interest, suggesting the possibility that an "immature DG" might commonly exist in human. Recently, it was reported that Disc1, a susceptibility gene for schizophrenia, is highly expressed in DG in adulthood [52,53] and is important for organizing newly generated as well as mature neurons and for regulating the neuronal integration in the DG, which may contribute to the pathophysiology of psychiatric disorders conferred by this gene [54,55]. "Immature DG", that is associated with dramatic functional disturbance of DG, may render higher susceptibility to schizophrenia and/or other related psychiatric disorders. Altered CaMKII-related signaling or any other genetic/environmental factors that might cause a similar or equivalent endophenotype in the DG could affect susceptibility to schizophrenia or its related psychiatric disorders. Once reliable endophenotypes based on biologically well-defined pathophysiology, as exemplified by "immature DG", are established, diagnostic clinical criteria and classification of psychiatric disorders would be re-organized. A clear disadvantage of our endophenotype-based approach is that we don't have any methods to identify immature DG in living human at present. However, this problem would be solved in the near future, considering the remarkable advances in molecular and functional non-invasive imaging techniques. The products of the genes that were differentially expressed between the two groups in human post-mortem hippocampus and/or between the two genotypes of the mutant mice could also be utilized for diagnosing or subtyping psychiatric patients, if a small chemical probe is developed that can selectively recognize the gene products. Such an endophenotype-based diagnosis would lead to better treatment strategies with appropriate drugs for each disorder subtype.

## Conclusion

**Figure 6:**
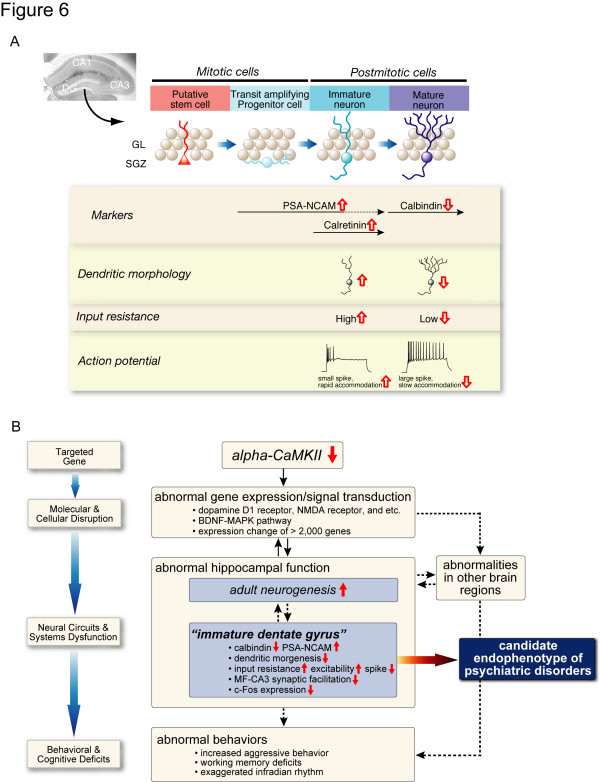
**Immature DG of Alpha-CaMKII+/- Mice**. (A) Cells proliferate in the subgranular layer (SGL), and migrate and differentiate into mature neurons in the granular layer (GL) in the adult hippocampus. Each stage of the neuronal development has cell markers and specific morphological and physiological properties. In the DG of alpha-CaMKII+/- mice, the number of cells expressing Polysialic acid-NCAM (PSA-NCAM), a marker for late-stage progenitor cells and immature neurons, and calretinin, a marker for immature neurons, was dramatically increased, whereas the amount of calbindin, a marker for mature neurons in the DG, -positive cells was markedly reduced. Electrophysiological study of the DG neurons showed that input resistance was high and the number of spikes during sustained depolarization was decreased in mutant mice. Furthermore, morphological analysis revealed that dendritic branching and length were decreased in the mutant DG. Collectively, the mutant DG granule cells had many features that are characteristic of immature DG neurons. Red arrows represent the specific changes in the DG of alpha-CaMKII+/- mice. (B) A schematic model of gene-to-behavior pathways in alpha-CaMKII+/- mice. Alpha-CaMKII deficiency leads multiple abnormal gene expression and signal transduction, which causes "immature DG" and impaired function of hippocampus and other brain regions, resulting in abnormal behaviors of alpha-CaMKII+/- mice. Based on the finding that expression changes of genes related to neurogenesis and neural maturation/migration, including calbindin, in hippocampus is associated with higher incidence of schizophrenic patients, "Immature DG" and its equivalent hippocampal functional abnormalities may serve as a promising candidate endophenotype of psychiatric disorders, such as schizophrenia and bipolar disorders.

In summary, we show that alpha-CaMKII heterozygous knockout mice have dysregulated behaviors, including a severe working memory deficit and an exaggerated infradian rhythm. Most neurons in the mutant DG failed to mature, with differential expressions of more than 2000 genes in hippocampus. Transcriptome analysis of human post-mortem brain revealed that biomarkers derived from mutants classified individuals into two clusters, one of which had higher susceptibility to schizophrenia and differentially expressed genes related to neural development, including calbindin, a marker for mature DG neurons. Taken together, we propose "immature DG" as a candidate endophenotype of schizophrenia and other psychiatric disorders (Figure 6).

## Methods

### Behavioral analysis

#### Animals and experimental design

alpha-CaMKII+/- mice were obtained from Jackson Laboratories (Bar Harbor, Maine). We used heterozygous alpha-CaMKII knockout mice, because it is hard to obtain homozygotes, due to a difficulty of mating between heterozygous male and heterozygous female mice. Mice were housed one per cage in a room with a 12-hr light/dark cycle (lights on at 7:00 a.m.) with access to food and water ad libitum. Behavioral testing was performed between 9:00 a.m. and 6:00 p.m. After the tests, the apparatus were cleaned with super hypochlorous water to prevent a bias due to olfactory cues. All behavioral tests were conducted in a manner similar to those described previously [14,56,57]. All behavioral testing procedures were approved by the Animal Care and Use Committee of Kyoto University Graduate School of Medicine.

#### Neurological screen

Neurological screen was performed with 9 to 10-wk-old male mice. The righting, whisker touch, and ear twitch reflexes were evaluated. A number of physical features, including the presence of whiskers or bald hair patches, were also recorded.

#### Neuromuscular strength

Neuromuscular strength was performed with 9 to 10-wk-old male mice, and tested with the grip strength test and wire hang test. A grip strength meter (O'Hara & Co., Tokyo, Japan) was used to assess forelimb grip strength. Mice were lifted and held by their tail so that their forepaws could grasp a wire grid. The mice were then gently pulled backward by the tail with their posture parallel to the surface of the table until they released the grid. The peak force applied by the forelimbs of the mouse was recorded in Newtons (N). Each mouse was tested three times, and the greatest value measured was used for statistical analysis. In the wire hang test, the mouse was placed on a wire mesh that was then inverted and waved gently, so that the mouse gripped the wire. Latency to fall was recorded, with a 60 s cut-off time.

#### Rotarod test

Motor coordination and balance were tested with the rotarod test. The rotarod test, using an accelerating rotarod (UGO Basile Accelerating Rotarod), was performed by placing 11 to 12-wk-old mice on rotating drums (3 cm diameter) and measuring the time each animal was able to maintain its balance on the rod. The speed of the rotarod accelerated from 4 to 40 rpm over a 5-min period.

#### Open field test

Locomotor activity was measured using an open field test. Open field test was performed with 10 to 11-wk-old male mice. Each mouse was placed in the center of the open field apparatus (40 × 40 × 30 cm; Accuscan Instruments, Columbus, OH). Total distance traveled (in cm), vertical activity (rearing measured by counting the number of photobeam interruptions), time spent in the center, the beam-break counts for stereotyped behaviors, and number of fecal boli were recorded. Data were collected for 120 min.

#### Light/dark transition test

Light/dark transition test was performed with 9 to 10-wk-old male mice, as previously described [58]. The apparatus used for the light/dark transition test consisted of a cage (21 × 42 × 25 cm) divided into two sections of equal size by a partition containing a door (O'Hara & Co., Tokyo, Japan). One chamber was brightly illuminated (390 lux), whereas the other chamber was dark (2 lux). Mice were placed into the dark side and allowed to move freely between the two chambers with the door open for 10 min. The total number of transitions between chambers, time spent in each side, first latency to enter the light side and distance traveled were recorded automatically.

#### Elevated plus-maze test

Elevated plus-maze test was performed with 10 to 11-wk-old male mice. The elevated plus-maze (O'Hara & Co., Tokyo, Japan) consisted of two open arms (25 × 5 cm) and two enclosed arms of the same size, with 15-cm high transparent walls. The arms and central square were made of white plastic plates and were elevated to a height of 55 cm above the floor. To minimize the likelihood of animals falling from the apparatus, 3-mm high plastic ledges were provided for the open arms. Arms of the same type were arranged at opposite sides to each other. Each mouse was placed in the central square of the maze (5 × 5 cm), facing one of the closed arms. Mouse behavior was recorded during a 10-min test period. The number of entries into, and the time spent in open and enclosed arms, were recorded. For data analysis, we used the following four measures: the percentage of entries into the open arms, the time spent in the open arms (s), the number of total entries, and total distance traveled (cm). Data acquisition and analysis were performed automatically using Image EP software.

#### Hot plate test

The hot plate test was used to evaluate sensitivity to a painful stimulus. 10 to 11-wk-old mice were placed on a 55.0 (± 0.3)°C hot plate (Columbus Instruments), and latency to the first hind-paw response was recorded. The hind-paw response was defined as either a foot shake or a paw lick.

#### Startle response/prepulse inhibition tests

Startle response/prepulse inhibition tests were performed with 11 to 12-wk-old male mice. A startle reflex measurement system was used (O'Hara & Co., Tokyo, Japan) to measure startle response and prepulse inhibition. A test session began by placing a mouse in a plastic cylinder where it was left undisturbed for 10 min. White noise (40 ms) was used as the startle stimulus for all trial types. The startle response was recorded for 140 ms (measuring the response every 1 ms) starting with the onset of the prepulse stimulus. The background noise level in each chamber was 70 dB. The peak startle amplitude recorded during the 140 ms sampling window was used as the dependent variable. A test session consisted of six trial types (i.e., two types for startle stimulus only trials, and four types for prepulse inhibition trials). The intensity of the startle stimulus was 110 or 120 dB. The prepulse sound was presented 100 ms before the startle stimulus, and its intensity was 74 or 78 dB. Four combinations of prepulse and startle stimuli were used (74–110, 78–110, 74–120, and 78–120). Six blocks of the six trial types were presented in pseudorandom order such that each trial type was presented once within a block. The average inter-trial interval was 15 s (range: 10–20 s).

#### Social interaction test in a novel environment

Social interaction test in a novel environment was performed with 10 to 11-wk-old male mice. Two mice of identical genotypes that were previously housed in different cages, were placed into a box together (40 × 40 × 30 cm) and allowed to explore freely for 10 min. Social behavior was monitored by a CCD camera, which was connected to a Macintosh computer. Analysis was performed automatically using Image SI software. Total duration of contact, the number of contacts, the number of active contacts, mean duration per contact, and total distance traveled were measured. The number of active contacts was defined as follows. Images were captured at 1 frame per second, and distance traveled between two successive frames was calculated for each mouse. If the two mice contacted each other and the distance traveled by either mouse was longer than 5 cm, the behavior was considered as 'active contact'.

#### Porsolt forced swim test

Porsolt forced swim test was performed with 11 to 12-wk-old male mice. The apparatus consisted of four plastic cylinders (20 cm height × 10 cm diameter). The cylinders were filled with water (23°C) up to a height of 7.5 cm. Mice were placed into the cylinders, and their behavior recorded over a 10-min test period. Data acquisition and analysis were performed automatically, using Image PS software (see 'Image Analysis'). Distance traveled was measured by Image OF software (see 'Image Analysis') using stored image files.

#### Eight-arm radial maze test

Eight-arm radial maze test was performed with 13 to 14-wk-old male mice. Fully-automated eight-arm radial maze apparatuses (O'Hara & Co., Tokyo, Japan) were used. The floor of the maze was made of white plastic, and the wall (25 cm high) consisted of transparent plastic. Each arm (9 × 40 cm) radiated from an octagonal central starting platform (perimeter 12 × 8 cm) like the spokes of a wheel. Identical food wells (1.4 cm deep and 1.4 cm in diameter) with pellet sensors were placed at the distal end of each arm. The pellets sensors were able to automatically record pellet intake by the mice. The maze was elevated 75 cm above the floor and placed in a dimly-lit room with several extra-maze cues. During the experiment, the maze was maintained in a constant orientation. One week before pretraining, animals were deprived of food until their body weight was reduced to 80% to 85% of the initial level. Pretraining started on the 8th day. Each mouse was placed in the central starting platform and allowed to explore and consume food pellets scattered on the whole maze for a 30-min period (one session per mouse). After completion of the initial pretraining, mice received another pretraining to take a food pellet from each food well after being placed at the distal end of each arm. A trial was finished after the mouse consumed the pellet. This was repeated eight times, using eight different arms, for each mouse. After these pretraining trials, actual maze acquisition trials were performed. In the spatial working memory task of the eight-arm radial maze, all eight arms were baited with food pellets. Mice were placed on the central platform and allowed to obtain all eight pellets within 25 min. A trial was terminated immediately after all eight pellets were consumed or 25 min had elapsed. An 'arm visit' was defined as traveling more than 5 cm from the central platform. The mice were confined at the center platform for 5 s after each arm choice. The animals went through one trial per day. For each trial, arm choice, latency to obtain all pellets, distance traveled, number of different arms chosen within the first eight choices, the number of arm revisited, and omission errors were automatically recorded. In the reference memory task of the eight-arm radial maze, one of the eight arms was consistently baited with one food pellet in the food well and a trial was terminated immediately after the one pellet was consumed. Data acquisition, control of guillotine doors, and data analysis were performed by Image RM software (see 'Image analysis').

#### Locomotor activity monitoring in home cage

Circadian rhythm monitoring in home cage was performed with 18 to 19-wk-old male mice. A system that automatically analyzes the locomotor activity of mice in their home cage was used [8]. The system contains a home cage (29 × 18 × 12 cm) and a filtered cage top, separated by a 13-cm-high metal stand containing an infrared video camera, which is attached to the top of the stand. Each mouse was individually housed in each home cage, and their locomotor activity was monitored for at least 3 mo. Outputs from the video cameras were fed into a Macintosh computer. Images from each cage were captured at a rate of one frame per second, and distance travelled was measured automatically using Image HA software (see 'Image analysis').

#### Image analysis

The applications used for the behavioral studies (Image LD, Image EP, Image RM, Image FZ, Image SI, and Image HA) were based on the public domain NIH Image program (developed at the U.S. National Institutes of Health and available on the Internet at ) and ImageJ program , which were modified for each test by Tsuyoshi Miyakawa (available through O'Hara & Co., Tokyo, Japan).

#### Statistical analysis

Statistical analysis was conducted using StatView (SAS Institute, Cary, NC). Data were analyzed by two-way ANOVA, or two-way repeated measures ANOVA, unless noted otherwise. Values in tables and graphs were expressed as mean ± s.e.m.

### Golgi impregnation

Golgi impregnation was performed using FD Rapid GolgiStain™ kit according to the manufacturer's directions (FD NeuroTechnologies, Inc., Ellicott City, MD). Briefly, the brains of 45-wk old control mice (n = 5) and mutant mice (n = 5) were obtained after cervical dislocation, immersed in the impregnation solution for one week in the dark, and cryoprotected for 48 h. After sectioning (100 μm-thick) and mounting on adhesive-coated slides, sections were stained in a development solution. After the slides containing hippocampal sections were scanned with a light microscope (Zeiss Axioplan2) outfitted with a CCD camera, the resulting images were analyzed by the ImageJ software . All slides were coded prior to the quantitative analysis, and the code was broken only after the analysis was completed. To be selected for analysis, Golgi-impregnated neurons had to satisfy the following criteria: (1) dark and consistent impregnation throughout the extent of the dendrites, (2) relative isolation from neighboring impregnated cells, and (3) cell bodies that were located toward the middle of the slice with dendrites lying in the same plane as the cell body. The number of dendritic intersections and dendritic length were measured in successive radial segments of 20 μm distance, using the center of the soma as a reference point (Sholl's analysis). Images of Golgi-impregnated neurons were captured using a Keyence Biozero BZ-8000.

### Biochemical assay of monoamine levels

Levels of dopamine, noradrenaline, serotonin, and their metabolites in the brain samples were determined by high-performance liquid chromatography (HPLC) with electrochemical detection [59]. Control mice (n = 9) and mutant mice (n = 9) (10 to 12-wk-old) were killed by decapitation after which their hippocampus, striatum, nucleus accumbens, and medial prefrontal cortex were immediately dissected and frozen in liquid nitrogen. Wet tissue samples were weighed and stored at -80°C until subsequent analysis. Tissues were homogenized in 9 volumes of 0.2 M perchloric acid prior to centrifugation at 20,000 g for 10 min. After the supernatant was neutralized with sodium acetate, the samples were analyzed by HPLC.

### Electrophysiology

Adult male alpha-CaMKII+/- mice and their wild-type littermates (13 to 18 wk old) were used for electrophysiological experiments. Mice were decapitated under halothane anesthesia and both hippocampi were isolated. Transverse hippocampal slices (370 mm) were cut using a tissue slicer (Leica VT1000S, Nussloch, Germany) in sucrose-containing saline composed of (in mM): sucrose 72, NaCl 80; KCl, 2.5; NaH_2_PO_4_, 1.0; NaHCO_3_, 26.2; glucose, 20;CaCl_2_, 0.5; MgCl_2_, 7; kynurenic acid, 0.1 – 0.2 (equilibrated with 95% O_2_/5% CO_2_). Slices were then incubated for 30 min at 30°C in standard saline (see below) and maintained in a humidified interface holding chamber at room temperature (24 – 27°C) before use. Electrophysiological recordings were made from slices placed in a submersion-type chamber superfused at 2 ml/min with the standard saline composed of (in mM): NaCl 125; KCl, 2.5; NaH_2_PO_4_, 1.0; NaHCO_3_, 26.2; glucose, 11;CaCl_2_, 2.5; MgCl_2_, 1.3 (equilibrated with 95% O_2_/5% CO_2_). Bath temperature was maintained at 26.5 – 27.5°C using an automated temperature controller. All procedures were approved by the institutional Animal Care and Use Committee.

Whole-cell recordings were made from granule cells in the dentate gyrus by using the blind whole-cell patch-clamp technique. Current-clamp recordings were made with a pipette filled with a solution composed of (in mM) potassium gluconate 140, HEPES 20, NaCl 8, MgATP 2, Na_2_GTP 0.3, EGTA 0.05 (pH adjusted to 7.2 with KOH). The recording pipette was placed in the middle third of the granule cell layer. Resting membrane potentials were measured immediately after rupture of the patch membrane. Liquid junction potentials were not corrected. Hyperpolarizing currents (4 – 16 pA, 400 ms) were injected through the recording pipette to measure the input resistance. Depolarizing currents (400 ms) were injected to evaluate action potential firing properties. The current intensity was increased by 20 to 40 pA steps, and the number of action potentials and the latency of the first action potential were measured.

Field excitatory postsynaptic potentials (EPSPs) arising from mossy fibers (MFs) were recorded from the CA3 region of the hippocampus. Bipolar tungsten stimulating electrodes were placed in the dentate gyrus granule cell layer to activate MFs and a recording glass electrode filled with 2 M NaCl was placed in the stratum lucidum of the CA3 region. Single electrical stimulations were delivered at a frequency of 0.05 Hz unless otherwise specified. An agonist of group II metabotropic glutamate receptors, (2S,2'R,3'R)-2-(2',3'-Dicarboxycyclopropyl)glycine (DCG-IV), which selectively suppresses the MF input, was applied at the end of each experiment. DCG-IV was less effective in mutant mice (92.8 ± 1.0% block in wild-type mice, n = 20; 87.6 ± 0.5% block in mutant mice, n = 23; p < 0.0001). Given changes in the expression level of various genes in these mutant mice, this reduction of the DCG-IV effect might be due to altered expression of metabotropic glutamate receptors and/or molecules involved in downstream signaling. The EPSP amplitude was measured throughout the study following a previously described method unless otherwise specified [60]. In most experiments, the experimenter was blind to the genotype of the mice. All recordings were made using a Multiclamp 700B amplifier (Molecular Devices, Sunnyvale, CA), filtered at 2 to 10 kHz and stored in a personal computer via a digital interface (digitized at 5 – 20 kHz). DCG-IV was purchased from Tocris (Bristol, UK). All values are expressed as means ± s.e.m. Statistical significance was evaluated by two-tailed Student's t-test with a significance level of p less than 0.05.

### Microarray analysis

Microarray experiments were performed with 12-wk-old male mice (3 control mice and 3 mutant mice) and 26 to 40-wk old mice (6 control mice and 15 mutant mice). The older mice had been tested in the behavioral test battery, including general health and neurological screen, light/dark transition test, open field test, elevated plus maze test, social interaction test, rotarod, prepulse inhibition, and Porsolt forced swim test. RNA was isolated by using the TRIzol method (Invitrogen, Carlsbad, CA) from the hippocampus of control mice (n = 9) and alpha-CaMKII+/- mice (n = 18), followed by purification, using RNeasy columns (Qiagen, Valencia, CA). Double-stranded cDNA was synthesized from the total RNA, and in vitro transcription reaction was then performed on biotin-labeled RNA that was made from the cDNA. Labeled RNA was hybridized with Mouse Genome 430 2.0 Array (Affymetrix, Santa Clara, CA) containing 45101 probe sets, and washed according to the manufacturer's recommendations. The hybridized probe array was then stained with streptavidin-conjugated phycoerythrin, and each GeneChip was scanned by an Affymetrix GeneChip Scanner 3000 (GCS3000). GeneChip analysis was performed with Microarray Analysis Suite version 5.0. All of the genes represented on the GeneChip were globally normalized. Two-way analysis of variance was performed for each probe, and the probes, for which the expression levels were significantly changed (genotype effect, p < 0.05) and not affected by the interaction between genotype and age (p > 0.05), were identified. Expression data and other information used in this paper will be available at the ArrayExpress database (, accession number: E-MEXP-962).

### Quantitative RT-PCR

RT-PCR was performed to validate the microarray results. Total RNA was isolated from 27 to 29-wk-old control mice and alpha-CaMKII+/- mice. First-strand cDNA was prepared from 2 ug of DNase I-treated total RNA using SuperScript III reverse transcriptase (Invitrogen, Carlsbad, CA). The expression of related genes was quantified using the SYBR green reagent (2× SYBR Green PCR Master Mix; Qiagen, Valencia, CA) following the instructions of the manufacturer. Quantitative PCR was performed using DNA Engine Opticon 2 Real-Time PCR Detection System(Bio-Rad, Hercules, CA) with conditions as follows: 15 min at 95°C, then 45 cycles of 15 sec at 94°C, 30 sec at 60°C, 30 sec at 72°C and 1 min at 65°C. β-actin was amplified from all samples to normalize expression. The following primers were used: *tryptophan 2,3-dioxygenase*(1–105), 5'-ATGAGTGGGTGCCCGTTTG and 5'-GGCTCTGTTTACACCAGTTTGAG; *desmoplakin*(7–113), 5'-GCTGAAGAACACTCTAGCCCA and 5'-ACTGCTGTTTCCTCTGAGACA; *interleukin 1 receptor, type I*(16–149), 5'-GTGCTACTGGGGCTCATTTGT and 5'-GGAGTAAGAGGACACTTGCGAAT; *nephronectin*(88–206), 5'-GTTTCTTCAATCGGCCTATGTCG and 5'-ATCCTTGCTAGTCTCTGCCTT; *pregnancy upregulated non-ubiquitously expressed CaM kinase*(142–293), 5'-AAGAAAGCACTTCGGGGCAA and 5'-AGTTCACCACCTGTTACCAGC; *solute carrier family 39 (metal ion transporter), member 6*(419–524), 5'-GTCACACGGTTGCTGGTAAAA and 5'-GGGCGAGATCCTTTCCCTAGA; *β-actin*(851–962), 5'-AGTGTGACGTTGACATCCGTA and 5'-GCCAGAGCAGTAATCTCCTTCT. Ct values used were the means of duplicate replicates.

### Human genetic study

#### Subjects

In the association analyses, two independent samples were examined: for the initial screening, 376 patients with schizophrenia (195 males and 181 females; mean age ± standard deviation (SD) 42.5 ± 14.8 y), 115 patients with bipolar disorder (56 males and 59 females; 46.2 ± 13.7 y), and 347 controls (194 males and 153 females; 34.6 ± 13.6 y), and for the following confirmation analysis, 368 patients with schizophrenia (204 males and 164 females; 52.8 ± 13.7 y), 149 patients with bipolar disorder (76 males and 73 females; 47.1 ± 15.2 y), and 382 controls (202 males and 180 females; 40.3 ± 13.6 y).

Sixty-four controls were used as subjects for linkage disequilibrium (LD) evaluation. These subjects were also included in the initial screening scan. Characterization details and psychiatric assessment of these subjects were identical to those published elsewhere [61,62]. All subjects were unrelated to each other and ethnically Japanese.

After the study was described to the subjects, written informed consent was obtained from each subject. This study was approved by the Ethics Committee at Fujita Health University and Nagoya University School of Medicine.

#### Mutation search

Primer pairs were designed using information from the GenBank sequence (accession number: NT-029289.10) and 23 amplified regions, which covered the coding exon and 5'-flanking region 968 bp upstream. The details are as previously described [63]. Sequences of primer pairs are available on request.

#### Single nucleotide polymorphism (SNP) selection and Linkage Disequilibrium (LD) evaluation

For the evaluation of LD, we included SNPs from databases (dbSNP, NCBI; and SNPbrowser2.0, Applied Biosystems, Foster City, CA). First we determined 'LD blocks' with the criteria D' > 0.8, using HAPLOVIEW ver 3.0 software [64]. We excluded the minor allele frequencies of SNPs less than 0.05. Next, haplotype-tagging SNPs were selected within each LD block for 90% haplotype coverage using SNPtagger software [65].

#### SNP genotyping

We used TaqMan assays, primer extension using denaturing high performance liquid chromatography, PCR-RFLP assays, and direct sequencing. Primer pair sequences are available upon request.

#### Statistical analysis

Genotype deviation from the Hardy-Weinberg equilibrium (HWE) was evaluated by c^2 ^test (SAS/Genetics, release 8.2, SAS Institute Japan Inc., Tokyo, Japan). Marker-trait association was evaluated allele/genotype-wise with c^2 ^test (SPSS 10.0 J, SPSS Japan Inc.), and haplotype-wise with the log likelihood ratio test (COCAPHASE 2.403 program [66]). Rare haplotypes found in less than 5% of cases and control subjects were excluded from the association analysis to provide greater sensitivity and accuracy. The significance level for all statistical tests was 0.05.

### Autoradiography

Frozen brains were obtained from 25-wk-old alpha-CaMKII+/- mice and control mice, and were cut into 10-mm-thick coronal sections with a HM560 cryotome (Carl Zeiss, Oberkochen, Germany). The sections were mounted on slide glass (Matsunami Glass, Osaka, Japan) and stored at -80°C until analyzed. Dopamine D1 and NMDA receptor levels were determined analyzing specific binding of [^3^H]SCH233090 (1 nM) and [^3^H]MK801 (5 nM), respectively, to the brain slices using autoradiography. The buffers and incubation times were as described in previous reports [67,68]. Non-specific binding of [^3^H]SCH23390 and [^3^H]MK801 was determined by adding 1 mM of flupenxiol and 200 mM of ketamine, respectively, to the reaction. Dopamine D2, dopamine transporter, serotonin transporter, and central benzodiazepine binding site levels were determined by [^3^H]raclopride (2 nM), [^3^H]GBR12935 (1 nM), [^3^H]citalopram (1 nM), and [^3^H]flumazenil (1 nM), respectively. The buffers and incubation times were as described in previous reports [67,69-71]. Non-specific binding of [^3^H]raclopride, [^3^H]GBR12935, [^3^H]citalopram and [^3^H]flumazenil were determined by adding 1 mM of butaclamol, 10 mM of nomifensine, 1 mM of fluoxetine, and 10 mM of flunitrazepam, respectively. Following the incubation, the samples were rinsed with ice-cold buffer, and desalted with ice-cold distilled water. The slices were subsequently dried under warm blowing air and exposed to an imaging plate (Fuji Film, Tokyo, Japan) for 10 to 14 days. The imaging plate was subsequently scanned with a BAS5000 system (Fuji Film). Regions of interest (ROIs) were defined on the images using a Multi Gauge^® ^software (Fuji Film), and densitometric assay for each ROI was performed using autoradiographic [^3^H]micro-scales (GE Healthcare Bio-Sciences Corp., Piscataway, NJ).

### Immunoblotting Analysis

After the brains of 11 to 12-wk-old control mice (n = 6) and mutant mice (n = 6) were dissected, hippocampus (HF), striatum (Str), cingulate cortex (CC), and amygdala (AM) tissues were homogenized in 300 μl of homogenizing buffer containing 50 mM Tris-HCl (pH 7.4), 0.5% Triton X-100, 4 mM EGTA, 10 mM EDTA, 1 mM Na_3_VO_4_, 40 mM sodium pyrophosphate, 50 mM NaF, 100 nM calyculin A, 50 μg/ml leupeptin, 25 μg/ml pepstatin A, 50 μg/ml trypsin inhibitor, and 1 mM dithiothreitol. Insoluble material was removed by a 10-min centrifugation at 15,000 rpm. After determining protein concentration in supernatants using Bradford's solution, samples were boiled for 3 min in Laemmli's sample buffer [72]. Samples containing equivalent amounts of protein were subjected to sodium dodecyl sulfate-polyacrylamide gel electrophoresis. Proteins were transferred to an Immobilon PVDF membrane for 2 h at 70 V. After blocking with TTBS solution (50 mM Tris-HCl, pH 7.5, 150 mM NaCl, and 0.1% Tween 20) containing 2.5% bovine serum albumin for 1 h at room temperature, membranes were incubated overnight at 4°C with anti-phospho CaMKII (1:5000) [73], anti-CaMKII (1:5000) [73], calcineurin A (1:2000) [74], anti-phospho-ERK (1:2000, Cell Signaling, Beverly, MA). Bound antibodies were visualized using the enhanced chemiluminescence detection system (Amersham Life Science, Buckinghamshire, UK) and analyzed semiquantitatively using the National Institutes of Health Image program.

### Immunohistochemistry

For the analysis of the expression of markers related to neurogenesis and differentiation, at least five (7-wk-old) wild-type and heterozygote mice were examined. There was a consistent difference between the genotypes. Mice were deeply anesthetized with sodium pentobarbital and transcardially perfused with 4% paraformaldehyde (PFA) and 0.5% picric acid in 0.1 M phosphate buffered saline (PBS). The brains were removed and further immersion-fixed in the same fixative at 4°C for 2 h and 14-mm-thick coronal sections were prepared on a cryostat (Leica). The sections were washed with Tris-buffered saline containing Tween 20 (pH 7.4). For immunostaining, the cryostat sections were incubated at 4°C for 18 h with the following primary antibodies: mouse anti-polysialic acid-NCAM (PSA-NCAM) monoclonal antibody (IgM, 1:500 dilution; CHEMICON, Temecula, CA), rabbit anti-calretinin 7699/4 polyclonal antibody (1:3000 dilution; SWANT, Bellinzona, Switzerland), rabbit anti-calbindin D-28k polyclonal antibody (1:3000 dilution; SWANT), and mouse anti-calmodulin-dependent protein kinase II (CAMKII) monoclonal antibody (IgG, 1:500 dilution; CHEMICON). For detection of the antigen localization, the sections were incubated at 4°C for 2 h with Alexa Fluor (488 or 594)-conjugated goat anti-mouse IgM or IgG (1:400 dilution; Invitrogen) and/or Alexa Fluor (488 or 594)-conjugated goat anti-rabbit IgG (1:400 dilution; Invitrogen). Fluorescent signals were detected using a confocal laser-scanning microscope (LSM5 Pascal, Zeiss) or a fluorescence microscope (Axioplan-2, Zeiss).

### Analysis of c-Fos expression following footshock

For the analysis of c-Fos expression, electric shocks (0.6 mA, 125 V) were delivered to control mice (8 wk-old, n = 4; 13 wk-old, n = 8) and alpha-CaMKII+/- mice (8 wk-old, n = 4; 13 wk-old, n = 6) 10 times through the metal grids in the bottom of a chamber. The duration of each foot shock was 1 s, and the interval between pairs of shocks was 30 s. Two hours after exposure to the last foot shock, mice were perfusion-fixed with 4% PFA and 0.5% picric acid in 0.1 M PBS. The brains were removed and further immersion-fixed in the same fixative at 4°C overnight and 70-μm-thick coronal sections were prepared on a Vibratome. The immunostaining was performed by incubating free-floating sections with rabbit anti-c-Fos polyclonal antibody (Santa Cruz Biotechnology, 1:5000 dilution) at 4°C overnight. After rinse with PBS, the sections were incubated at 4°C overnight with Alexa Fluor 488-conjugated goat anti-rabbit IgG (1:300 dilution; Invitrogen), and the fluorescent signals were detected as described above. The number of c-Fos-immunoreactive cells in the specified regions or subdivisions were counted on the confocal images in a blinded manner.

### BrdU labeling analyses

Mice (7-wk-old) received four injections of 5-bromo-2-deoxyuridine (BrdU) (Sigma, St. Louis, MO), one every 2 h, at 50 mg/kg body weight (10 mg/ml in PBS). Mice were perfusion-fixed with 4% PFA and 0.5% picric acid in 0.1 M PBS 1 d after the BrdU injections. The brains were removed and further immersion-fixed in the same fixative at 4°C for 2 h and 14-μm-thick coronal sections were prepared on a cryostat (Leica). The sections were boiled in 0.01 M citric acid for 10 min and incubated in 2N HCl for 10 min at 37°C, and washed with PBS. The sections were incubated at 4°C for 18 h with mouse BrdU monoclonal antibody (IgG, 1:100 dilution; Becton Dickinson, San Jose, CA). After washing with PBS, the sections were incubated at 4°C for 2 h with Alexa Fluor 488-conjugated goat anti-mouse IgG (1:400 dilution; Invitrogen), and the fluorescent signals were detected as described above. For quantitative analysis, every fourth section (14 mm; throughout the DG in its rostrocaudal extension) was used for counting, and the total cell number was obtained by multiplying the number of counted cells by 4 [75]. The cells were counted in a blind manner.

### Electron microscopy

The mice (8 to 13-wk-old) were perfused transcardially with a solution of 2% PFA and 2.5% glutaraldehyde in PBS. The brains were removed and coronal sections (100 mm thick) were prepared on a Vibratome. Sections containing the dorsal hippocampus were osmicated, dehydrated, and embedded in epoxy resin. Ultrathin sections of the hippocampal CA3 region were prepared and stained with lead citrate and uranyl acetate, and observed under a Hitachi H-7000 electron microscope.

### Gene expression studies using postmortem brain tissue

The expression data for 166 hippocampal genes from the BioExpress database [76,77] (GeneLogic) were used in this study (see Additional file 2, Table S3). Differential analysis of mouse GeneChip data indicates that 130 mouse probes (n = 3, p < 0.05 [t-test], fold change > 1.5) were affected in mutants, independent of age or experience. Fifty-seven human orthologues corresponding to the 130 mouse probes were present in at least 10% of the 166 hippocampi and were identified as biomarkers. Of the 166 hippocampi, there were 18 from patients with schizophrenia, 2 with bipolar disorder, and 1 with schizoaffective disorder (see Additional file 2, Table S3). Variance analysis of hippocampal expression levels was performed to identify the 10 best genes of the 57 biomarkers so that the 21 hippocampi of patients with psychiatric disorders would be distinguished from the remaining 147 hippocampi (see Additional file 2, Table S4). Cluster analysis of the 166 hippocampi based on the expression levels of the 10 best biomarkers was performed using Spotfire DecisionSite™ For Functional Genomics . Of the 166 hippocampi, 44 were obtained from donors with no known history of CNS-related illness. Of the 44 donors with no CNS-related illness, 28 were over 50 years old and 26 were male. We defined age- and gender-affected probes by differential expression analysis of older (over 50 years old) vs. younger donors with no CNS-related illness and of male vs. female donors with no CNS-related illness, respectively (p < 0.05, fold change > 1.8). For cluster-based differential gene expression analysis, age- and gender-affected probes were eliminated and expression levels of other probes in clustered psychiatric hippocampi were compared with those in the other cluster obtained from donors with no CNS-related illness as specific controls (p < 0.01, fold change > 2.0). In addition, only probes whose present flags were marked in over 75% of hippocampi in at least one of the two groups (case and specific controls) were considered significant.

## Competing interests

The authors declare that they have no competing interests.

## Authors' contributions

TM is responsible for the original concept and overall design of the research. NY, KTakao, KTanda, KO, KToyama, and TM performed the behavioral test battery, microarray analysis, and morphometric analysis using Golgi staining of mutant mice. MM, MS, and SY performed the immunohistochemistry study, analysis of c-Fos expression, BrdU labelling analysis, and electron microscopy study of mutant mice. KKobayashi and HS performed electrophysiological experiments on mutant mice. YK and KKanzaki performed postmortem brain analysis. JM, MH, and TS performed autoradiography analysis of mutant mice. KF performed immunoblotting Analysis. YS and HI performed biochemical assays. MI, NI, and NO performed the human genetic study. TM, NY, KTakao, MM, SY, KKobayashi, YK, JM, KF, HI, and MI wrote the manuscript. All authors read and approved the final manuscript.

## Availability and requirements











## Supplementary Material

Additional file 1 Supplementary figures. Supplementary Figures S1–S14 are included in this file. Click here for file

Additional file 2 Supplementary tables. Supplementary Tables S1–S5 are included in this file. Click here for file

## References

[B1] Bearden CE, Reus VI, Freimer NB (2004). Why genetic investigation of psychiatric disorders is so difficult. Curr Opin Genet Dev.

[B2] Gottesman II, Gould TD (2003). The endophenotype concept in psychiatry: etymology and strategic intentions. Am J Psychiatry.

[B3] Gainetdinov RR, Mohn AR, Caron MG (2001). Genetic animal models: focus on schizophrenia. Trends Neurosci.

[B4] Powell CM, Miyakawa T (2006). Schizophrenia-relevant behavioral testing in rodent models: a uniquely human disorder?. Biol Psychiatry.

[B5] Chen J, Lipska BK, Weinberger DR (2006). Genetic mouse models of schizophrenia: from hypothesis-based to susceptibility gene-based models. Biol Psychiatry.

[B6] Arguello PA, Gogos JA (2006). Modeling madness in mice: one piece at a time. Neuron.

[B7] Zeng H, Chattarji S, Barbarosie M, Rondi-Reig L, Philpot BD, Miyakawa T, Bear MF, Tonegawa S (2001). Forebrain-specific calcineurin knockout selectively impairs bidirectional synaptic plasticity and working/episodic-like memory. Cell.

[B8] Miyakawa T, Leiter LM, Gerber DJ, Gainetdinov RR, Sotnikova TD, Zeng H, Caron MG, Tonegawa S (2003). Conditional calcineurin knockout mice exhibit multiple abnormal behaviors related to schizophrenia. Proc Natl Acad Sci USA.

[B9] Gerber DJ, Hall D, Miyakawa T, Demars S, Gogos JA, Karayiorgou M, Tonegawa S (2003). Evidence for association of schizophrenia with genetic variation in the 8p21.3 gene, PPP3CC, encoding the calcineurin gamma subunit. Proc Natl Acad Sci USA.

[B10] Yamada K, Gerber DJ, Iwayama Y, Ohnishi T, Ohba H, Toyota T, Aruga J, Minabe Y, Tonegawa S, Yoshikawa T (2007). Genetic analysis of the calcineurin pathway identifies members of the EGR gene family, specifically EGR3, as potential susceptibility candidates in schizophrenia. Proc Natl Acad Sci USA.

[B11] Liu YL, Fann CS, Liu CM, Chang CC, Yang WC, Hung SI, Yu SL, Hwang TJ, Hsieh MH, Liu CC (2007). More evidence supports the association of PPP3CC with schizophrenia. Mol Psychiatry.

[B12] Crawley JN (2000). What's Wrong With My Mouse? Behavioral Phenotyping of Transgenic and Knockout Mice.

[B13] Takao K, Miyakawa T (2006). Investigating Gene-to-Behavior Pathways in Psychiatric Disorders: The Use of a Comprehensive Behavioral Test Battery on Genetically Engineered Mice. Ann N Y Acad Sci.

[B14] Arron JR, Winslow MM, Polleri A, Chang CP, Wu H, Gao X, Neilson JR, Chen L, Heit JJ, Kim SK (2006). NFAT dysregulation by increased dosage of DSCR1 and DYRK1A on chromosome 21. Nature.

[B15] Goldman-Rakic PS (1994). Working memory dysfunction in schizophrenia. J Neuropsychiatry Clin Neurosci.

[B16] Silva AJ, Stevens CF, Tonegawa S, Wang Y (1992). Deficient hippocampal long-term potentiation in alpha-calcium-calmodulin kinase II mutant mice. Science.

[B17] Silva AJ, Paylor R, Wehner JM, Tonegawa S (1992). Impaired spatial learning in alpha-calcium-calmodulin kinase II mutant mice. Science.

[B18] Elgersma Y, Sweatt JD, Giese KP (2004). Mouse genetic approaches to investigating calcium/calmodulin-dependent protein kinase II function in plasticity and cognition. J Neurosci.

[B19] Winder DG, Sweatt JD (2001). Roles of serine/threonine phosphatases in hippocampal synaptic plasticity. Nat Rev Neurosci.

[B20] Chen C, Rainnie DG, Greene RW, Tonegawa S (1994). Abnormal fear response and aggressive behavior in mutant mice deficient for alpha-calcium-calmodulin kinase II. Science.

[B21] Frankland PW, O'Brien C, Ohno M, Kirkwood A, Silva AJ (2001). Alpha-CaMKII-dependent plasticity in the cortex is required for permanent memory. Nature.

[B22] Frankland PW, Bontempi B, Talton LE, Kaczmarek L, Silva AJ (2004). The involvement of the anterior cingulate cortex in remote contextual fear memory. Science.

[B23] Lein ES, Hawrylycz MJ, Ao N, Ayres M, Bensinger A, Bernard A, Boe AF, Boguski MS, Brockway KS, Byrnes EJ (2007). Genome-wide atlas of gene expression in the adult mouse brain. Nature.

[B24] Cameron HA, McKay RD (2001). Adult neurogenesis produces a large pool of new granule cells in the dentate gyrus. J Comp Neurol.

[B25] Abrous DN, Koehl M, Le Moal M (2005). Adult neurogenesis: from precursors to network and physiology. Physiol Rev.

[B26] Kempermann G, Jessberger S, Steiner B, Kronenberg G (2004). Milestones of neuronal development in the adult hippocampus. Trends Neurosci.

[B27] Schmidt-Hieber C, Jonas P, Bischofberger J (2004). Enhanced synaptic plasticity in newly generated granule cells of the adult hippocampus. Nature.

[B28] Ambrogini P, Lattanzi D, Ciuffoli S, Agostini D, Bertini L, Stocchi V, Santi S, Cuppini R (2004). Morpho-functional characterization of neuronal cells at different stages of maturation in granule cell layer of adult rat dentate gyrus. Brain Res.

[B29] Liu X, Tilwalli S, Ye G, Lio PA, Pasternak JF, Trommer BL (2000). Morphologic and electrophysiologic maturation in developing dentate gyrus granule cells. Brain Res.

[B30] Marchal C, Mulle C (2004). Postnatal maturation of mossy fibre excitatory transmission in mouse CA3 pyramidal cells: a potential role for kainate receptors. J Physiol.

[B31] Tamminga CA, Holcomb HH (2005). Phenotype of schizophrenia: a review and formulation. Mol Psychiatry.

[B32] Harrison PJ (2004). The hippocampus in schizophrenia: a review of the neuropathological evidence and its pathophysiological implications. Psychopharmacology (Berl).

[B33] Lisman J, Schulman H, Cline H (2002). The molecular basis of CaMKII function in synaptic and behavioural memory. Nat Rev Neurosci.

[B34] Gaudilliere B, Konishi Y, de la Iglesia N, Yao G, Bonni A (2004). A CaMKII-NeuroD signaling pathway specifies dendritic morphogenesis. Neuron.

[B35] Zhou Z, Hong EJ, Cohen S, Zhao WN, Ho HY, Schmidt L, Chen WG, Lin Y, Savner E, Griffith EC (2006). Brain-specific phosphorylation of MeCP2 regulates activity-dependent Bdnf transcription, dendritic growth, and spine maturation. Neuron.

[B36] Hoogenraad CC, Feliu-Mojer MI, Spangler SA, Milstein AD, Dunah AW, Hung AY, Sheng M (2007). Liprinalpha1 degradation by calcium/calmodulin-dependent protein kinase II regulates LAR receptor tyrosine phosphatase distribution and dendrite development. Dev Cell.

[B37] Lee JK, Cho JH, Hwang WS, Lee YD, Reu DS, Suh-Kim H (2000). Expression of neuroD/BETA2 in mitotic and postmitotic neuronal cells during the development of nervous system. Dev Dyn.

[B38] Jeltsch H, Bertrand F, Lazarus C, Cassel JC (2001). Cognitive performances and locomotor activity following dentate granule cell damage in rats: role of lesion extent and type of memory tested. Neurobiol Learn Mem.

[B39] Vann SD, Brown MW, Erichsen JT, Aggleton JP (2000). Fos imaging reveals differential patterns of hippocampal and parahippocampal subfield activation in rats in response to different spatial memory tests. J Neurosci.

[B40] Dranovsky A, Hen R (2006). Hippocampal neurogenesis: regulation by stress and antidepressants. Biol Psychiatry.

[B41] van Praag H, Kempermann G, Gage FH (1999). Running increases cell proliferation and neurogenesis in the adult mouse dentate gyrus. Nat Neurosci.

[B42] Aimone JB, Wiles J, Gage FH (2006). Potential role for adult neurogenesis in the encoding of time in new memories. Nat Neurosci.

[B43] Lipska BK, Weinberger DR (1993). Delayed effects of neonatal hippocampal damage on haloperidol-induced catalepsy and apomorphine-induced stereotypic behaviors in the rat. Brain Res Dev Brain Res.

[B44] Weinberger DR (1995). From neuropathology to neurodevelopment. Lancet.

[B45] Lipska BK (2004). Using animal models to test a neurodevelopmental hypothesis of schizophrenia. J Psychiatry Neurosci.

[B46] Bayer KU, Lohler J, Schulman H, Harbers K (1999). Developmental expression of the CaM kinase II isoforms: ubiquitous gamma- and delta-CaM kinase II are the early isoforms and most abundant in the developing nervous system. Brain Res Mol Brain Res.

[B47] Petralia RS, Sans N, Wang YX, Wenthold RJ (2005). Ontogeny of postsynaptic density proteins at glutamatergic synapses. Mol Cell Neurosci.

[B48] APA (2000). Diagnostic and statistical manual of mental disorders: DSM-IV-TR, text revision edn.

[B49] Rasetti R, Malone C, Mattay VS, Rivero O, Callicott JH, Meyer-Lindenberg A, Rujescu D, Straub RE, Weinberger DR (2007). Genetic variation in CAMK2A affects brain structure and function in normal individuals. 37th annual meeting of the Society for Neuroscience; San Diego, California.

[B50] Harrison PJ, Weinberger DR (2005). Schizophrenia genes, gene expression, and neuropathology: on the matter of their convergence. Mol Psychiatry.

[B51] Manji HK, Gottesman II, Gould TD (2003). Signal transduction and genes-to-behaviors pathways in psychiatric diseases. Sci STKE.

[B52] Austin CP, Ma L, Ky B, Morris JA, Shughrue PJ (2003). DISC1 (Disrupted in Schizophrenia-1) is expressed in limbic regions of the primate brain. Neuroreport.

[B53] Meyer KD, Morris JA (2008). Immunohistochemical analysis of Disc1 expression in the developing and adult hippocampus. Gene Expr Patterns.

[B54] Duan X, Chang JH, Ge S, Faulkner RL, Kim JY, Kitabatake Y, Liu XB, Yang CH, Jordan JD, Ma DK (2007). Disrupted-In-Schizophrenia 1 regulates integration of newly generated neurons in the adult brain. Cell.

[B55] Kvajo M, McKellar H, Arguello PA, Drew LJ, Moore H, MacDermott AB, Karayiorgou M, Gogos JA (2008). A mutation in mouse Disc1 that models a schizophrenia risk allele leads to specific alterations in neuronal architecture and cognition. Proc Natl Acad Sci USA.

[B56] Miyakawa T, Yamada M, Duttaroy A, Wess J (2001). Hyperactivity and intact hippocampus-dependent learning in mice lacking the M1 muscarinic acetylcholine receptor. J Neurosci.

[B57] Ihara M, Yamasaki N, Hagiwara A, Tanigaki A, Kitano A, Hikawa R, Tomimoto H, Noda M, Takanashi M, Mori H (2007). Sept4, a Component of Presynaptic Scaffold and Lewy Bodies, Is Required for the Suppression of alpha-Synuclein Neurotoxicity. Neuron.

[B58] Takao K, Miyakawa T (2006). Light/dark transition test for mice. Journal of Visualized Experiments.

[B59] Sumi-Ichinose C, Urano F, Kuroda R, Ohye T, Kojima M, Tazawa M, Shiraishi H, Hagino Y, Nagatsu T, Nomura T, Ichinose H (2001). Catecholamines and serotonin are differently regulated by tetrahydrobiopterin. A study from 6-pyruvoyltetrahydropterin synthase knockout mice. J Biol Chem.

[B60] Kobayashi K, Suzuki H (2007). Dopamine selectively potentiates hippocampal mossy fiber to CA3 synaptic transmission. Neuropharmacology.

[B61] Ikeda M, Iwata N, Suzuki T, Kitajima T, Yamanouchi Y, Kinoshita Y, Inada T, Ujike H, Ozaki N (2005). Association analysis of chromosome 5 GABAA receptor cluster in Japanese schizophrenia patients. Biol Psychiatry.

[B62] Ikeda M, Iwata N, Suzuki T, Kitajima T, Yamanouchi Y, Kinoshita Y, Ozaki N (2006). No association of serotonin transporter gene (SLC6A4) with schizophrenia and bipolar disorder in Japanese patients: association analysis based on linkage disequilibrium. J Neural Transm.

[B63] Suzuki T, Iwata N, Kitamura Y, Kitajima T, Yamanouchi Y, Ikeda M, Nishiyama T, Kamatani N, Ozaki N (2003). Association of a haplotype in the serotonin 5-HT4 receptor gene (HTR4) with Japanese schizophrenia. Am J Med Genet B Neuropsychiatr Genet.

[B64] Barrett JC, Fry B, Maller J, Daly MJ (2005). Haploview: analysis and visualization of LD and haplotype maps. Bioinformatics.

[B65] Ke X, Cardon LR (2003). Efficient selective screening of haplotype tag SNPs. Bioinformatics.

[B66] Dudbridge F (2003). Pedigree disequilibrium tests for multilocus haplotypes. Genet Epidemiol.

[B67] Mansour A, Meador-Woodruff JH, Bunzow JR, Civelli O, Akil H, Watson SJ (1990). Localization of dopamine D2 receptor mRNA and D1 and D2 receptor binding in the rat brain and pituitary: an in situ hybridization-receptor autoradiographic analysis. J Neurosci.

[B68] Sakurai SY, Penney JB, Young AB (1993). Regionally distinct N-methyl-D-aspartate receptors distinguished by quantitative autoradiography of [3H]MK-801 binding in rat brain. J Neurochem.

[B69] D'Amato RJ, Largent BL, Snowman AM, Snyder SH (1987). Selective labeling of serotonin uptake sites in rat brain by [3H]citalopram contrasted to labeling of multiple sites by [3H]imipramine. J Pharmacol Exp Ther.

[B70] Richfield EK (1991). Quantitative autoradiography of the dopamine uptake complex in rat brain using [3H]GBR 12935: binding characteristics. Brain Res.

[B71] Sur C, Fresu L, Howell O, McKernan RM, Atack JR (1999). Autoradiographic localization of alpha5 subunit-containing GABAA receptors in rat brain. Brain Res.

[B72] Laemmli UK (1970). Cleavage of structural proteins during the assembly of the head of bacteriophage T4. Nature.

[B73] Fukunaga K, Goto S, Miyamoto E (1988). Immunohistochemical localization of Ca2+/calmodulin-dependent protein kinase II in rat brain and various tissues. J Neurochem.

[B74] Morioka M, Fukunaga K, Yasugawa S, Nagahiro S, Ushio Y, Miyamoto E (1992). Regional and temporal alterations in Ca2+/calmodulin-dependent protein kinase II and calcineurin in the hippocampus of rat brain after transient forebrain ischemia. J Neurochem.

[B75] Maekawa M, Takashima N, Arai Y, Nomura T, Inokuchi K, Yuasa S, Osumi N (2005). Pax6 is required for production and maintenance of progenitor cells in postnatal hippocampal neurogenesis. Genes Cells.

[B76] Sequeira A, Gwadry FG, Ffrench-Mullen JM, Canetti L, Gingras Y, Casero RA, Rouleau G, Benkelfat C, Turecki G (2006). Implication of SSAT by gene expression and genetic variation in suicide and major depression. Arch Gen Psychiatry.

[B77] Papapetropoulos S, Ffrench-Mullen J, McCorquodale D, Qin Y, Pablo J, Mash DC (2006). Multiregional gene expression profiling identifies MRPS6 as a possible candidate gene for Parkinson's disease. Gene Expr.

