# Supplementary material for: Alpha-CaMKII deficiency causes immature dentate gyrus, a novel candidate endophenotype of psychiatric disorders
Source: Mol Brain. 2008 Sep 10;1:6. doi: 10.1186/1756-6606-1-6 (PMC2562999; doi:10.1186/1756-6606-1-6)
Supplement: Additional file 1 — Supplementary figures. Supplementary Figures S1–S14 are included in this file. [file 1756-6606-1-6-S1.pdf]

Figure S1

I. Increased locomotor activity

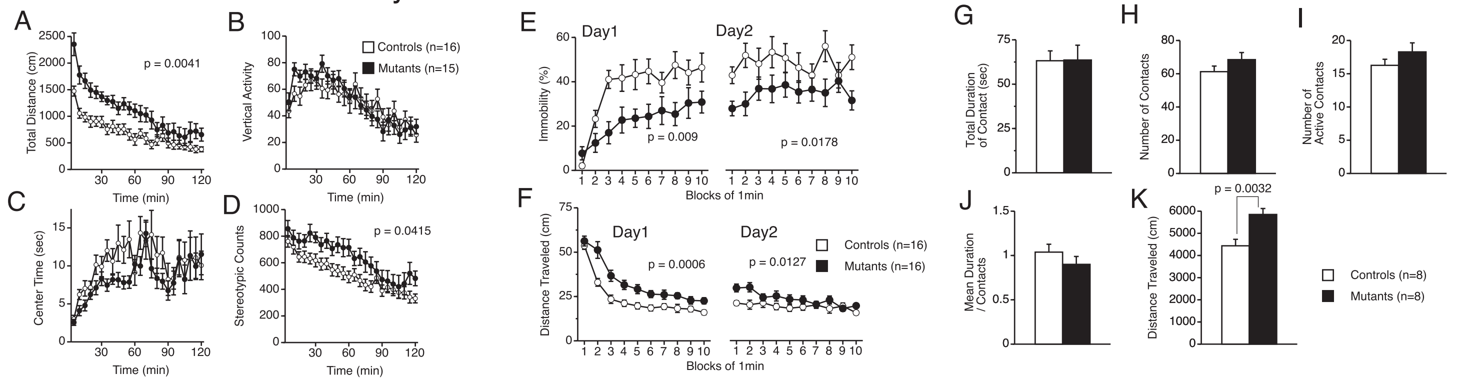

II. Decreased anxiety-like behavior

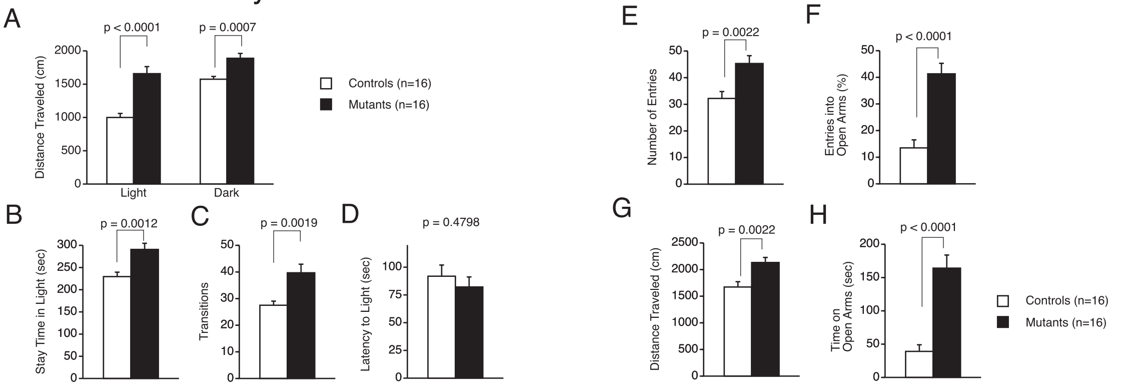

III. Other behavioral tests

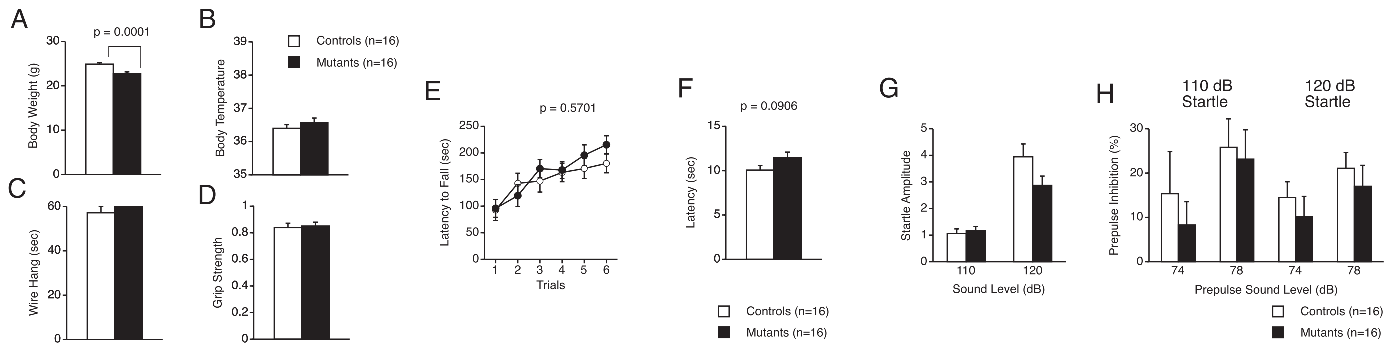

Figure S2

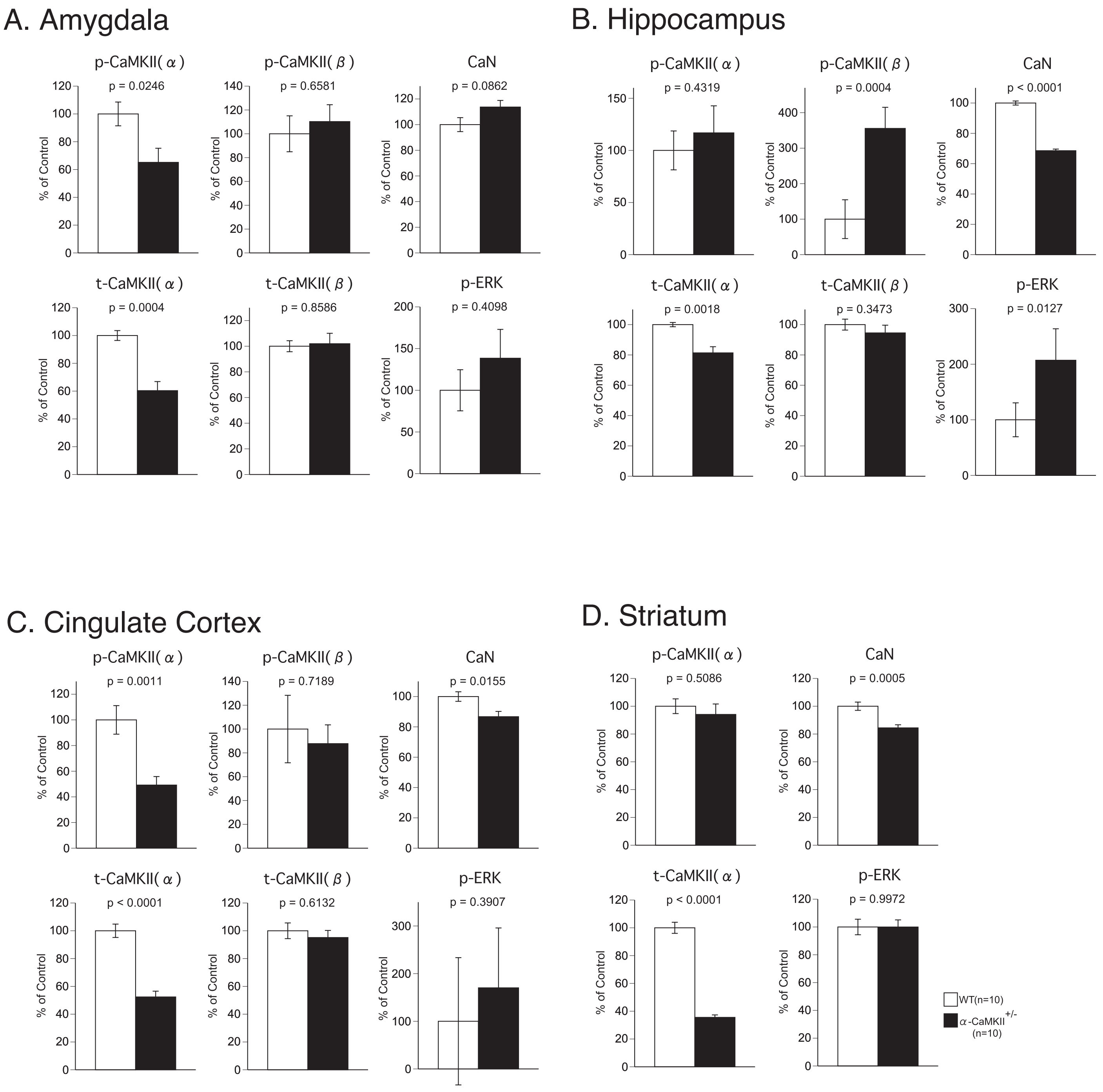
A. Amygdala
p-CaMKII(α)
p = 0.0246
p-CaMKII(β)
p = 0.6581
CaN
p = 0.0862
t-CaMKII(α)
p = 0.0004
t-CaMKII(β)
p = 0.8586
p-ERK
p = 0.4098
B. Hippocampus
p-CaMKII(α)
p = 0.4319
p-CaMKII(β)
p = 0.0004
CaN
p < 0.0001
t-CaMKII(α)
p = 0.0018
t-CaMKII(β)
p = 0.3473
p-ERK
p = 0.0127
C. Cingulate Cortex
p-CaMKII(α)
p = 0.0011
p-CaMKII(β)
p = 0.7189
CaN
p = 0.0155
t-CaMKII(α)
p < 0.0001
t-CaMKII(β)
p = 0.6132
p-ERK
p = 0.3907
D. Striatum
p-CaMKII(α)
p = 0.5086
CaN
p = 0.0005
t-CaMKII(α)
p < 0.0001
p-ERK
p = 0.9972
% of Control
WT(n=10)
α-CaMKII+/- (n=10)

Figure S3

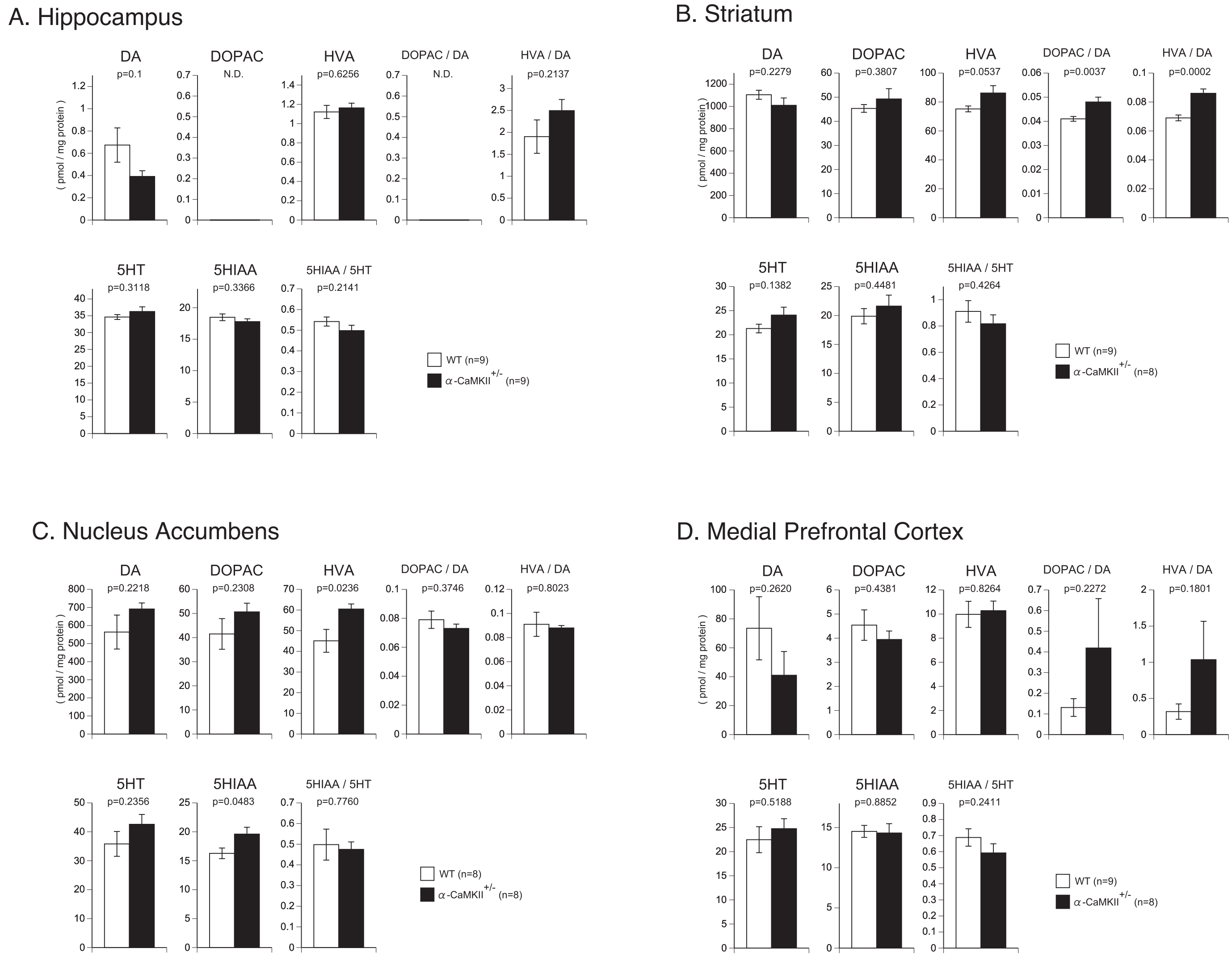
A. Hippocampus
DA
p=0.1
DOPAC
N.D.
HVA
p=0.6256
DOPAC / DA
N.D.
HVA / DA
p=0.2137
(pmol / mg protein)
5HT
p=0.3118
5HIAA
p=0.3366
5HIAA / 5HT
p=0.2141
WT (n=9)
α-CaMKII+/- (n=9)
B. Striatum
DA
p=0.2279
DOPAC
p=0.3807
HVA
p=0.0537
DOPAC / DA
p=0.0037
HVA / DA
p=0.0002
(pmol / mg protein)
5HT
p=0.1382
5HIAA
p=0.4481
5HIAA / 5HT
p=0.4264
WT (n=9)
α-CaMKII+/- (n=8)
C. Nucleus Accumbens
DA
p=0.2218
DOPAC
p=0.2308
HVA
p=0.0236
DOPAC / DA
p=0.3746
HVA / DA
p=0.8023
(pmol / mg protein)
5HT
p=0.2356
5HIAA
p=0.0483
5HIAA / 5HT
p=0.7760
WT (n=8)
α-CaMKII+/- (n=8)
D. Medial Prefrontal Cortex
DA
p=0.2620
DOPAC
p=0.4381
HVA
p=0.8264
DOPAC / DA
p=0.2272
HVA / DA
p=0.1801
(pmol / mg protein)
5HT
p=0.5188
5HIAA
p=0.8852
5HIAA / 5HT
p=0.2411
WT (n=9)
α-CaMKII+/- (n=8)

Figure S4

A. Dopamine D1 Receptor

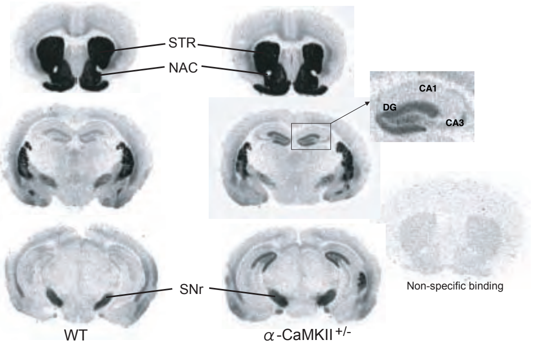

B. NMDA Receptor

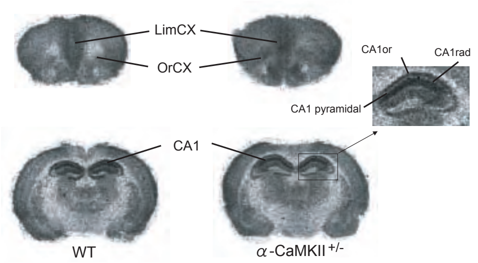

C. Dopamine D2 Receptor

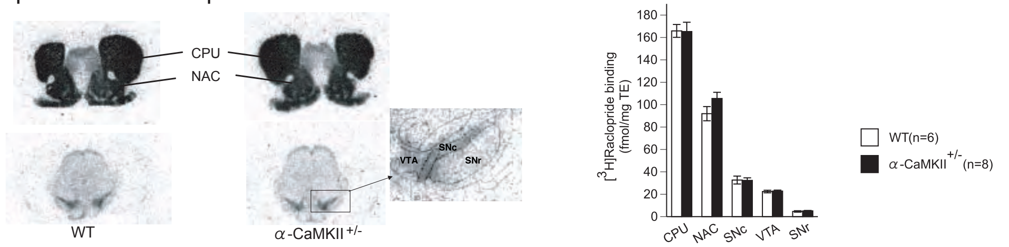

D. Serotonin 5-HT1A Receptor

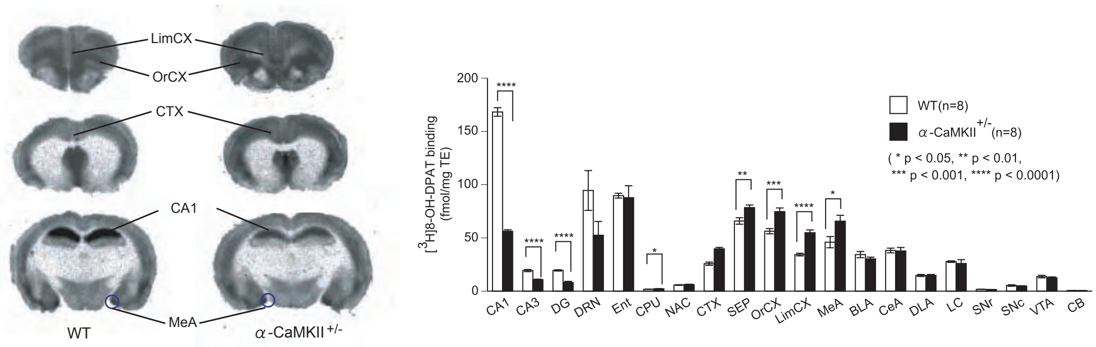

E. Central-type Benzodiazepine Receptor

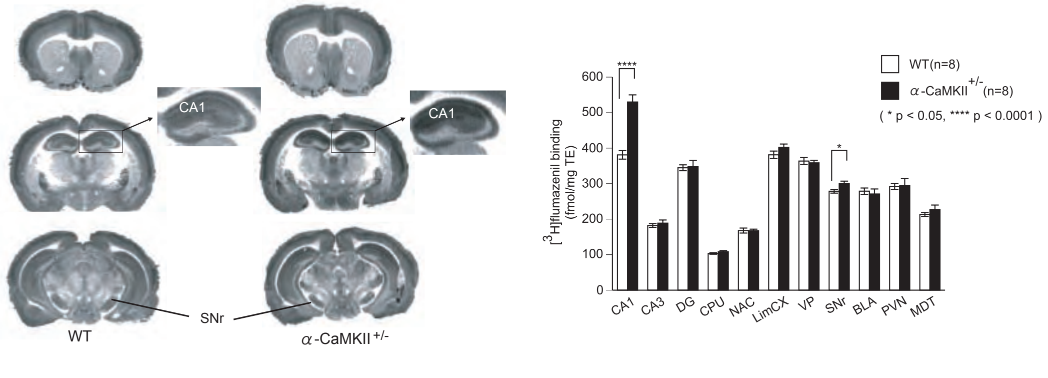

Figure S5

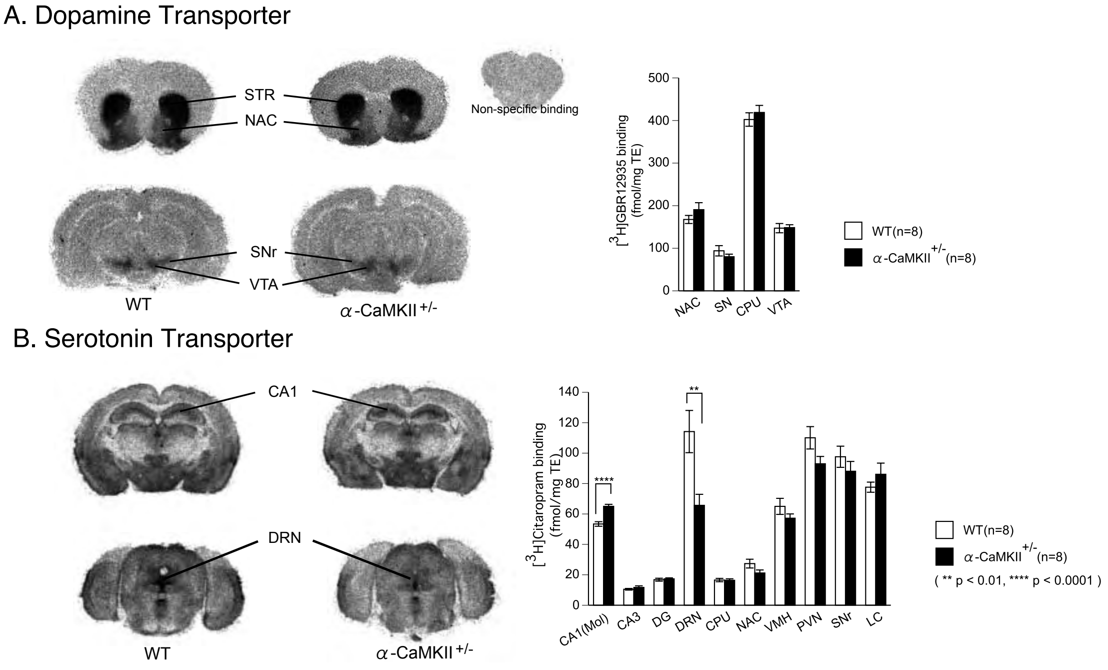

Figure S6

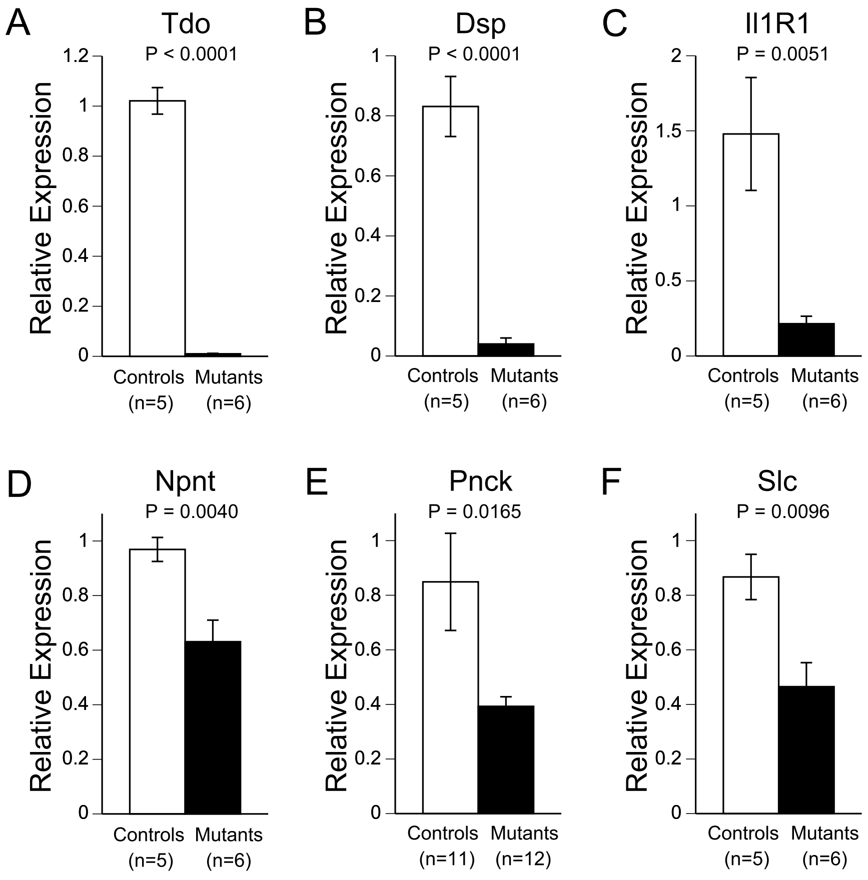

Figure S7

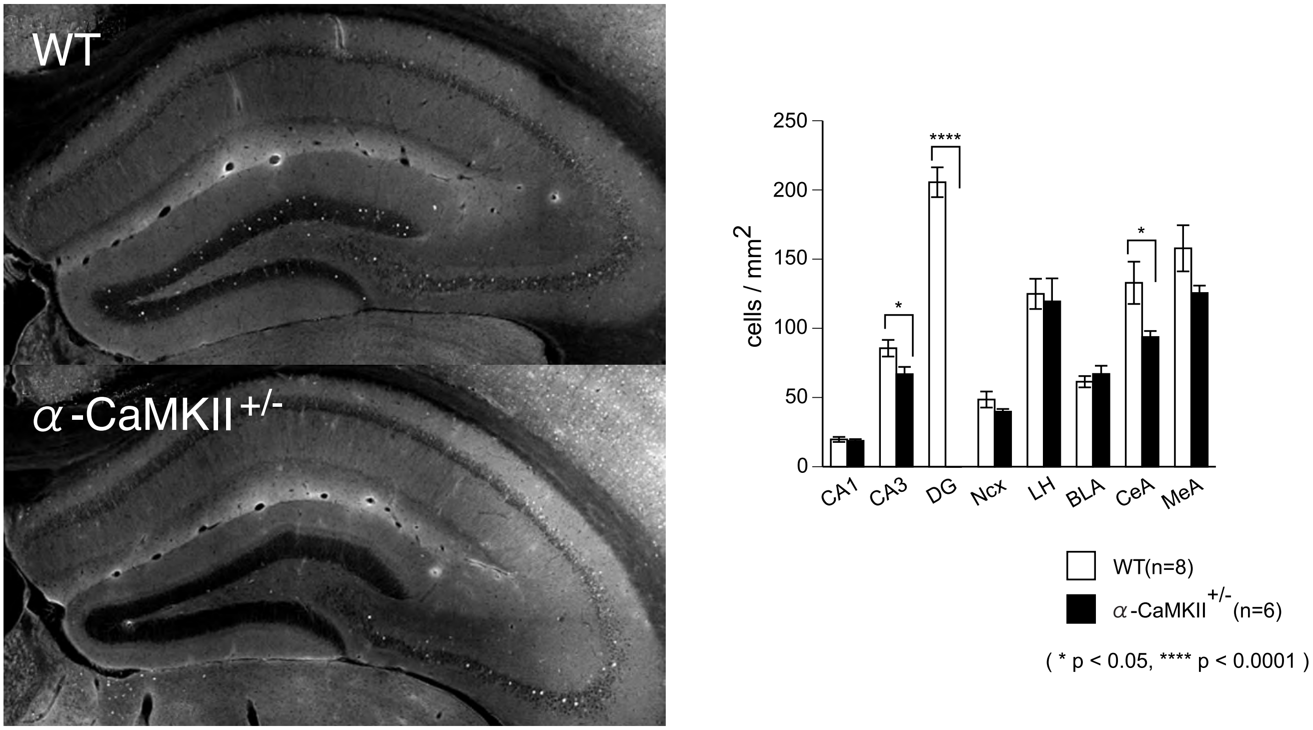

Figure S8

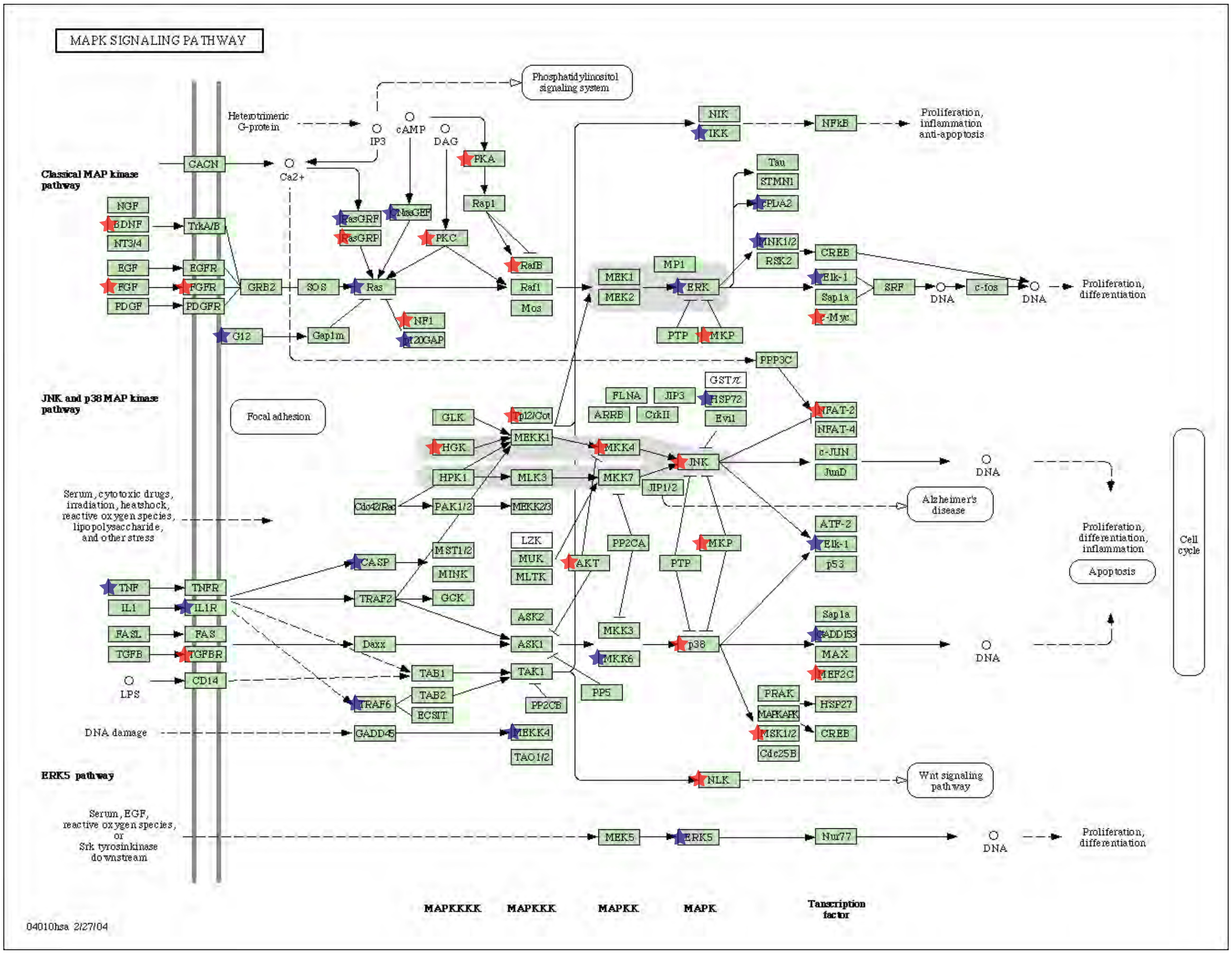

Figure S9

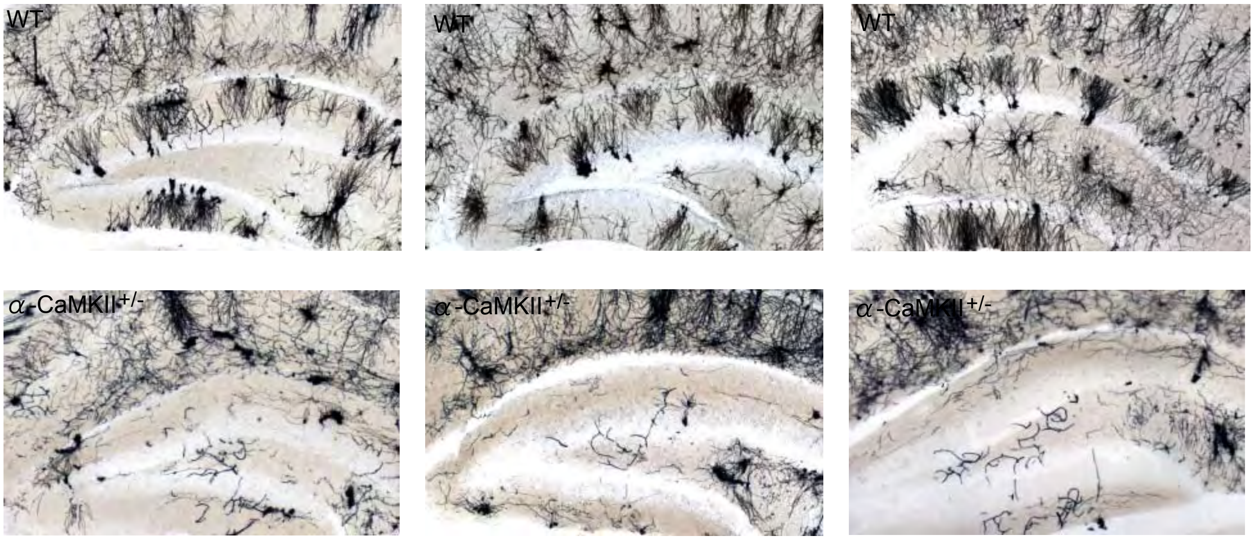

Figure S10

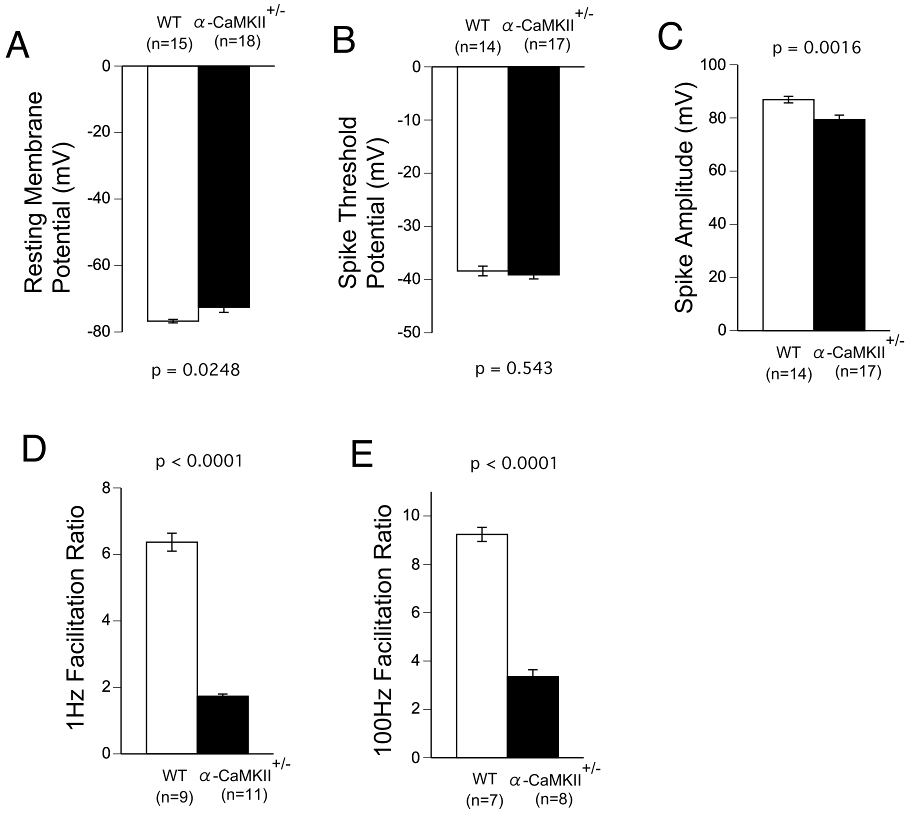

Figure S11

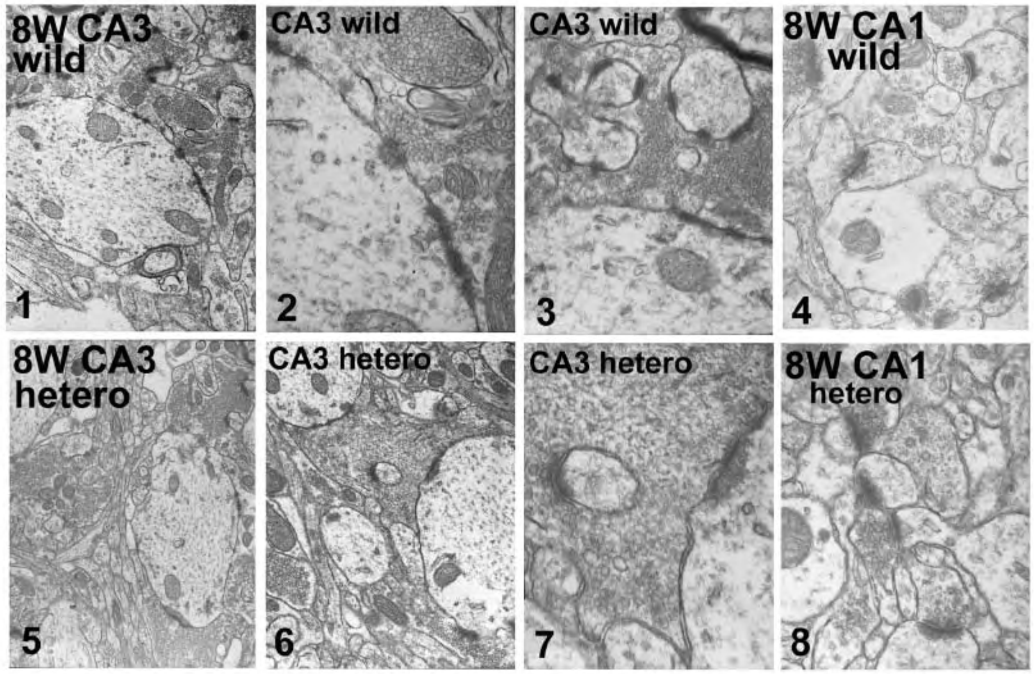

Figure S12

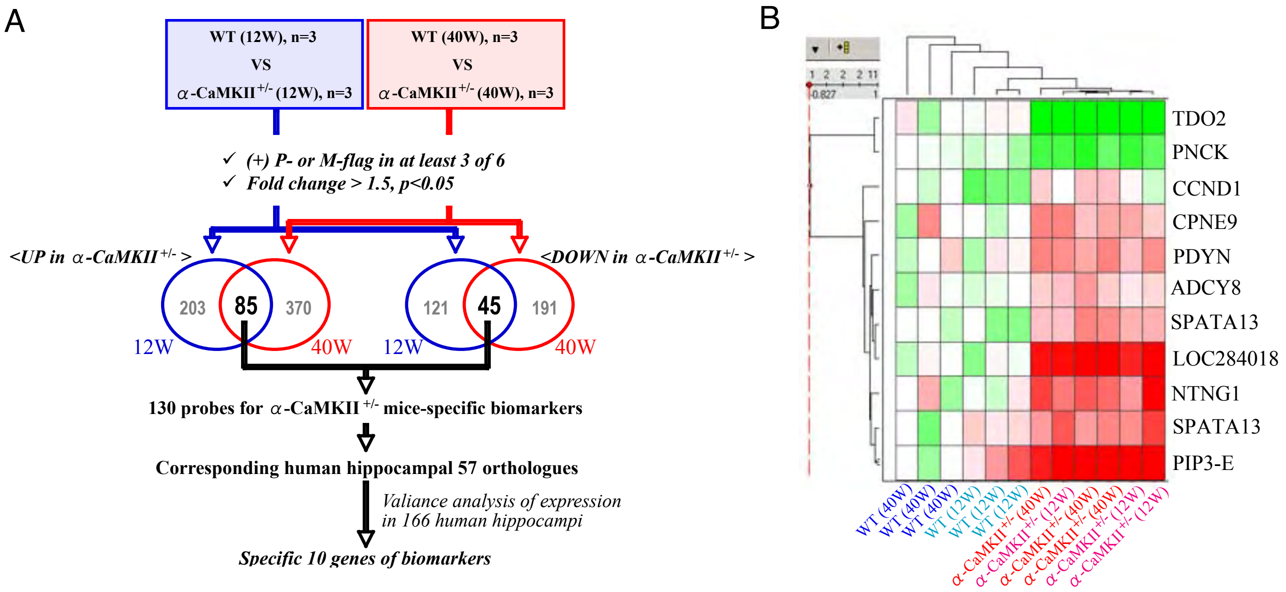

Figure S13

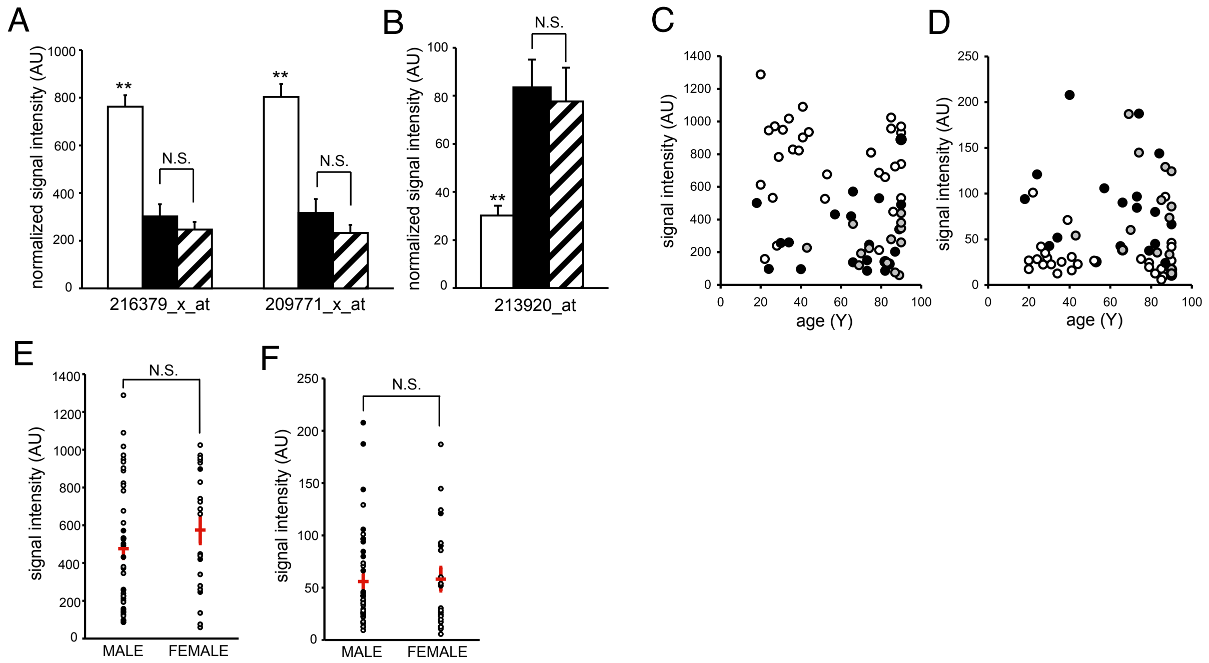

Figure S14

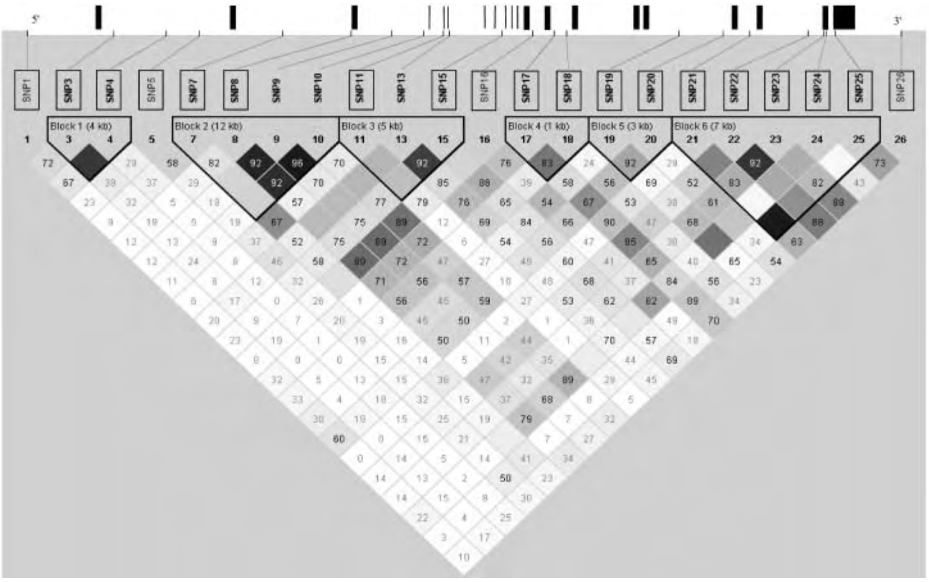

Supplementary Figure Legends

Figure S1. The results of the behavioural test battery of alpha-CaMKII+/- mice. alpha-CaMKII+/- mice showed increased locomotor activity in the open field test (I. A-D), and decreased depression-like behaviour in the Porsolt forced swim test (I. E, F). In the social interaction test in a novel environment, mutant mice were hyperactive, but social behaviour was not impaired (I. G-K). Anxiety-like behaviour was markedly decreased both in the light/dark transition test (II. A-D) and in the elevated plus maze test (II. E-H). Body weight was lower in mutants (III. A), but there were no differences between control and mutant mice in body temperature (III. B), wire hang test (III. C), grip strength test (III. D), rotarod test (III. E), hot plate test (III. F), acoustic startle response (III. G), and prepulse inhibition test (III. H). Error bars indicate s.e.m.

Figure S2. Immunoblotting analysis of major kinases / phosphatases of alpha-CaMKII+/- mice. The total amount of alpha CaMKII is decreased by 20% to 60% in the amygdala (A), hippocampus (B), cingulate cortex (C), and striatum (D). In the hippocampus of mutant mice, the amount of phosphorylated beta-CaMKII was increased, and, interestingly, the amount of calcineurin was significantly decreased. Error bars indicate s.e.m.

Figure S3. Quantification of monoamine content by HPLC. In alpha-CaMKII+/- mice, the levels of dopamine (DA), dihydroxyphenylacetic acid (DOPAC), and

homovanillic acid (HVA) were not changed in the hippocampus (A), striatum (B), nucleus accumbens (C), and medial prefrontal cortex (D), but dopamine turnover was accelerated in the striatum. No remarkable changes were observed in the levels of 5-tyrosine hydroxylase (5TH), 5-hydroxytryptamine (5HIAA), and 5-hydroxytryptamine (5HT) turnover. Error bars indicate s.e.m.

Figure S4. Autoradiography analysis of neurotransmitter receptors of alpha-CaMKII+/- mice. Alterations in radiolabeling of D1 receptor (A) and NMDA receptor (B) were detected in mutant mice. There was no significant change in D2 receptor binding levels in mutant mice (C). 5-HT1A receptor binding levels were decreased in CA1, CA3, and DG, and increased in the septum (SEP), orbital cortex (OrCX), limbic cortex (LimCX), and medial amygdaloid nucleus (MeA) (D). Central-type benzodiazepine receptor levels were increased in the hippocampal CA1 and substantia nigra pars reticulata (SNr) (E). CPU, caudate nucleus/putamen; NAC, nucleus accumbens; CA1rad, stratum radiatum of CA1; CA1or, stratum oriens of CA1; SNc, substantia nigra pars compacta; VTA, ventral tegmental area; CTX, cingulated cortex; DRN, dorsal raphe nucleus; Ent, entorhinal cortex; BLA, basolateral amygdaloid nucleus; CeA, central amygdaloid nucleus; DLA, dorsolateral amygdaloid nucleus; LC, locus coeruleus; VP, ventroposterior thalamic nucleus; PVN, paraventricular thalamic nucleus; MDT, mediodorsal thalamic nucleus. Error bars indicate s.e.m.

Figure S5. Autoradiography analysis of neurotransmitter transporters of alpha-CaMKII+/- mice. There was no significant change in levels of dopamine

transporter binding between control and mutant mice (A). The serotonin transporter level in mutant mice was increased in CA1 and decreased in dorsal raphe nucleus (DRN) (B). CA1 (Mol), molecular layer of hippocampal CA1; CPU, caudate nucleus/putamen; NAC, nucleus accumbens; VMH, ventromedial hypothalamic nucleus; PVN, paraventricular thalamic nucleus; SNr, substantia nigra pars reticulata; LC, locus coeruleus. Error bars indicate s.e.m.

Figure S6. Validation of the microarray results by quantitative RT-PCR. The mRNA expressions of *tryptophan 2,3-dioxygenase*(A), *desmoplakin*(B), *interleukin 1 receptor, type I*(C), *nephronectin*(D), *pregnancy upregulated non-ubiquitously expressed CaM kinase*(E), and *solute carrier family 39 (metal ion transporter), member 6*(F) were significantly decreased in the hippocampus of alpha-CaMKII+/- mice. Error bars indicate s.e.m.

Figure S7. c-Fos expression of 13-week-old alpha-CaMKII+/- mice. c-fos expression following electric foot shock was decreased in the DG of 13-week-old mutant mice to a similar extent of that of 8-week-old mutants. Ncx, neocortex; LH, lateral hypothalamus; CeA, central amygdaloid nucleus; MeA, medial amygdaloid nucleus. Error bars indicate s.e.m.

Figure S8. Differentially expressed genes involved in BDNF-MAPK pathways. The genes for which the expression levels were significantly changed (two-way ANOVA, for genotype effect, $p < 0.05$), were analyzed using the public databases "DAVID" (http://david.abcc.ncifcrf.gov/) and "KEGG"

(http://www.genome.ad.jp/kegg/). Upregulated genes are indicated by red stars, and downregulated genes are shown by blue stars.

Figure S9. Photomicrographs of Golgi-impregnated neurons in DG of alpha-CaMKII+/- mice and control mice. The number of Golgi-impregnated neurons in the DG was remarkably decreased in alpha-CaMKII+/- mice (lower figures) compared to control mice (upper figures).

Figure S10. Electrophysiology study of alpha-CaMKII+/- mice. Resting membrane potential was slightly depolarized in mutant mice (A). There was no change in spike threshold potential (B). Spike amplitude was lower in mutant mice (C). The 1-Hz and 100-Hz facilitation ratios were decreased in mutant mice (D, E). EPSP slopes were measured to calculate 100-Hz facilitation ratios. Error bars indicate s.e.m.

Figure S11. Electron microscope images of mossy fiber terminals in CA3 hippocampal region of alpha-CaMKII+/- mice. The formation of postsynaptic densities (PSD) and synaptic vesicles in the mossy fiber terminals were significantly reduced in mutant mice. In the contrast, the formation of PSD in the CA1 region was hardly affected in the mutant.

Figure S12. Specific hippocampal biomarkers for alpha-CaMKII+/- mice determined by differential expression analysis. Marker selection process is schematically represented (A). Expression profiles of 10 selected hippocampal biomarker genes are shown as a heat map (B). TDO2, tryptophan 2,3-

dioxygenase; PNCK, pregnancy upregulated non-ubiquitously CaM kinase; CCND1, cyclin D1; CPNE9, copine family member IX (LOC151835, copine-like protein); PDYN, prodynorphin; ADCY8, adenylate cyclase 8; SPATA13, spermatogenesis associated 13; NTNG1, netrin G1; PIP3-E, phosphoinositide-binding protein PIP3-E.

Figure S13. Differential expression of neurogenesis-related genes, CD24 and CUTL2, in hippocampi of clustered schizophrenic patients and controls. Normalized signal intensities of human Affymetrix probes for CD24 (216379_x_at in panels A, C and E; 209771_x_at in the panel A) and CUTL2 (213920_at in panels B, D and F) are shown. Values of mean+standard error are represented in bar graphs. Open, closed and hatched bars indicate donors without any CNS-related illness (n=30) in A cluster, specific patients with schizophrenic psychiatric diagnosis (n=19) in their own cluster (B cluster) and donors without psychiatric diagnosis (n=14) in the B cluster, respectively (A, B). The A and B clusters are shown in Figure 5. Corresponding values are individually indicated by an open, closed and gray dots, respectively (C-F). There is no significant correlation between expression levels and age (r^2=0.046, p=0.091 for CD24 in the panel C; r^2=0.00075, p=0.83 for CUTL2 in the panel D). Gender has also no significant effect on the expression levels (E, F). Red bars represent mean±standard error (E, F). ** p<0.01 vs. patients; N.S., not significant. (Tukey test in panels A and B, or t-test in panels E and F).

Figure S14. Linkage disequilibrium (LD) mapping of the human alpha-CaMKII gene and selected haplotype-tagging single nucleotide polymorphisms (htSNPs).
