# Supplementary material for: Alpha-CaMKII deficiency causes immature dentate gyrus, a novel candidate endophenotype of psychiatric disorders
Source: Mol Brain. 2008 Sep 10;1:6. doi: 10.1186/1756-6606-1-6 (PMC2562999; doi:10.1186/1756-6606-1-6)
Supplement: Additional file 2 — Supplementary tables. Supplementary Tables S1–S5 are included in this file. [file 1756-6606-1-6-S2.pdf]

Table S1

AffyID	GeneSymbol	GeneName	GenotypeEffect	AgeEffect	GenotypeAgeEffect	FoldChange
1435494_s_at	Dsp	desmoplakin	7.80826E-13	0.02609849	0.07667232	0.030804409
1419093_at	Tdo2	tryptophan 2,3-dioxygenase	2.58935E-12	0.02282035	0.140441	0.091837595
1452107_s_at	Npnt	Nephronectin	4.38022E-11	0.0237779	0.3282854	0.155621508
1435493_at	Dsp	desmoplakin	2.09973E-10	0.6083208	0.35077	0.036149346
1433681_x_at	CAPN3	CALPAIN 3	3.45309E-10	0.5129352	0.2590779	0.400079718
1419533_at	NHLH1	NESCIENT HELIX LOOP HELIX 1	5.09421E-10	0.6557419	0.1809421	6.064206131
1440849_at	6330417G04RIK	RIKEN CDNA 6330417G04 GENE	2.61509E-09	0.08842818	0.1563052	0.617701168
1448738_at	Calb1	calbindin-28K	2.85029E-09	0.004076686	0.9237763	0.541313043
1456934_at	---	---	3.2961E-09	0.5001476	0.3990841	0.488694237
1438710_at	Htr1a	5-hydroxytryptamine (serotonin) receptor 1A	4.51651E-09	0.01248947	0.3978848	0.488241969
1437125_at	Camk2a	calcium/calmodulin-dependent protein kinase II alpha	5.14192E-09	0.03323322	0.7471775	0.557550204
1453766_a_at	4931407K02RIK	RIKEN CDNA 4931407K02 GENE	5.42565E-09	0.01515114	0.07177448	0.37503591
1437226_x_at	Marcksl1	MARCKS-like 1	6.20397E-09	1.21985E-05	0.3843127	0.464735895
1425053_at	Isoc1	isochorismatase domain containing 1	8.51862E-09	0.3058534	0.4993614	0.541751189
1448950_at	IL1R1	INTERLEUKIN 1 RECEPTOR, TYPE I	1.94144E-08	0.01002575	0.06881165	0.193795662
1422711_a_at	PNCK	pregnancy upregulated non-ubiquitously expressed CaM kinase	2.03359E-08	0.941677	0.6354997	0.592686717
1428809_at	1810010H24RIK	RIKEN CDNA 1810010H24 GENE	2.16699E-08	0.4806871	0.16367689	3.068518765
1439854_at	AI838259	EXPRESSED SEQUENCE AI838259	2.5192E-08	0.8249145	0.1467698	0.482725912
1415935_at	Smoc2	SPARC related modular calcium binding 2	3.61754E-08	0.9555314	0.7034351	0.482937906
1431362_a_at	Smoc2	SPARC related modular calcium binding 2	5.27571E-08	0.4286949	0.2231905	0.410280495
1436532_at	DCAMKL3	DOUBLECORTIN AND CAM KINASE-LIKE 3	6.15391E-08	0.01007432	0.2369647	0.505840335
1429355_at	3300001K11RIK	RIKEN CDNA 3300001K11 GENE	8.32977E-08	0.3581192	0.07239313	1.96652709
1444150_at	Epb4.1	erythrocyte protein band 4.1	2.00196E-07	0.8057454	0.8408025	2.452332943
1418110_a_at	INPP5D	INOSITOL POLYPHOSPHATE-5-PHOSPHATASE D	2.39076E-07	0.003866117	0.2986411	1.514745934
1437091_at	ACCN4	AMILORIDE-SENSITIVE CATION CHANNEL 4, PITUITARY	2.82406E-07	0.9173669	0.1618937	1.708657524
1426601_at	SLC37A1	SOLUTE CARRIER FAMILY 37 (GLYCEROL-3-PHOSPHATE TRANSPORTER), MEMBER 1	5.49997E-07	0.3082207	0.1370467	2.364293397
1436127_at	CRHBP	CORTICOTROPIN RELEASING HORMONE BINDING PROTEIN	6.14282E-07	0.3068857	0.9028977	0.52481244
1429162_at	1500015A07RIK	RIKEN CDNA 1500015A07 GENE	6.21154E-07	0.4455292	0.3513842	0.619673983
1417391_a_at	Il16	interleukin 16	7.34573E-07	0.001492084	0.6991607	0.522788757
1455145_at	---	---	7.51534E-07	0.2222231	0.6677684	0.619888207
1441634_at	Ntng1	netrin G1	1.14888E-06	0.1184961	0.3113388	2.495794957
1422678_at	Dgat2	diacylglycerol O-acyltransferase 2	1.31476E-06	0.01069925	0.7783189	0.538616367
1431946_a_at	Apba2bp	amyloid beta (A4) precursor protein-binding, family A, member 1 binding protein	1.43164E-06	0.1774008	0.2112282	2.327988091
1437865_at	Spata13	spermatogenesis associated 13	1.5136E-06	0.000173011	0.1472761	1.71189481
1455401_at	Camkk2	calcium/calmodulin-dependent protein kinase kinase 2, beta	1.62236E-06	0.07823273	0.6499253	1.516324078
1424674_at	Slc39a6	solute carrier family 39 (metal ion transporter), member 6	1.72909E-06	0.0507446	0.7784477	0.498328603
1434129_s_at	Lhfpl2	lipoma HMGIC fusion partner-like 2	1.92298E-06	0.07765295	0.6525452	0.648801509
1425863_a_at	Ptpro	protein tyrosine phosphatase, receptor type, O	2.51724E-06	9.42245E-09	0.189972	1.377829731
1416072_at	CD34	CD34 ANTIGEN	2.53628E-06	0.000856461	0.2862298	1.58259522
1418809_at	PIRA1	PAIRED-IG-LIKE RECEPTOR A1	2.60244E-06	0.1681435	0.1070049	0.187722786
1425232_x_at	Arr3	arrestin 3, retinal	2.97602E-06	0.9702583	0.3481693	4.747538628
1438310_at	---	---	3.10494E-06	0.2043566	0.7407074	0.481302606
1416251_at	Mcm6	minichromosome maintenance deficient 6 (MIS5 homolog, S. pombe) (S. cerevisiae)	3.17964E-06	0.000235121	0.2401213	0.582450003
1460043_at	---	---	3.41545E-06	0.01072847	0.6926692	0.576788102
1460437_at	PSCD4	PLECKSTRIN HOMOLOGY, SEC7 AND COILED/COIL DOMAINS 4	3.55702E-06	0.001787764	0.07308535	0.475429166
1424525_at	GRP	GASTRIN RELEASING PEPTIDE	3.89637E-06	0.9183787	0.4079159	3.07325791
1448823_at	CXCL12	CHEMOKINE (C-X-C MOTIF) LIGAND 12	4.15387E-06	0.007714741	0.1003053	1.779142628
1457213_a_at	Dgkh	diacylglycerol kinase, eta	4.28691E-06	0.00140158	0.1814087	0.621582678
1440030_at	KCNT2	POTASSIUM CHANNEL, SUBFAMILY T, MEMBER 2	4.74741E-06	0.004444551	0.3763487	2.243666984
1450219_at	Htr1a	5-hydroxytryptamine (serotonin) receptor 1A	5.02181E-06	0.6102558	0.6893615	0.545071923
1455925_at	PRDM8	PR DOMAIN CONTAINING 8	5.60895E-06	0.3823469	0.1309533	0.547159897
1417777_at	LTB4DH	LEUKOTRIENE B4 12-HYDROXYDEHYDROGENASE	6.81071E-06	0.1216152	0.3300815	0.552434646
1434013_at	Ablim3	actin binding LIM protein family, member 3	6.90797E-06	0.7497033	0.6553869	0.688031967
1455082_at	Cblb	Casitas B-lineage lymphoma b	8.5433E-06	0.02355481	0.697795	0.585212724
1424675_at	Slc39a6	solute carrier family 39 (metal ion transporter), member 6	9.44505E-06	0.08022991	0.1315532	0.653239598
1450329_a_at	Arr3	arrestin 3, retinal	9.76891E-06	0.2328506	0.1219081	4.387125519
1451804_a_at	LRRC16	LEUCINE RICH REPEAT CONTAINING 16	1.03541E-05	0.6702009	0.4539123	0.759082193
1439578_at	Lsm11	U7 snRNP-specific Sm-like protein LSM11	1.05261E-05	0.967071	0.9413416	1.461096612
1440761_at	SSBP2	SINGLE-STRANDED DNA BINDING PROTEIN 2	1.07582E-05	0.000602744	0.4961755	0.479202864
1439327_at	Ccbe1	collagen and calcium binding EGF domains 1	1.07872E-05	0.28766009	0.6468348	0.374233039
1429402_at	Glt8d2	glycosyltransferase 8 domain containing 2	1.14272E-05	0.0949719	0.4220833	0.542805454
1423915_at	OLFML2B	OLFACTOMEDIN-LIKE 2B	1.27725E-05	0.01575689	0.09620764	0.684164121
1416658_at	Frzb	frizzled-related protein	1.29068E-05	0.01585719	0.622829	0.555570756
1443876_at	Camk2a	Calcium/calmodulin-dependent protein kinase II alpha	1.35961E-05	0.004169342	0.5472687	0.523393387
1415922_s_at	Marcksl1	MARCKS-like 1	1.43481E-05	0.3215486	0.1092545	0.670359513
1449337_at	Tdo2	tryptophan 2,3-dioxygenase	1.43995E-05	0.2172683	0.7368353	0.518791156
1438007_at	AI851790	EXPRESSED SEQUENCE AI851790	1.60133E-05	0.000213598	0.35793	0.673431713
1416266_at	PDYN	PRODYNORPHIN	1.66676E-05	0.619489	0.6768647	1.699868621
1422677_at	Dgat2	diacylglycerol O-acyltransferase 2	1.72826E-05	0.04712177	0.3823081	0.598174146
1426712_at	SLC6A15	SOLUTE CARRIER FAMILY 6 (NEUROTRANSMITTER TRANSPORTER), MEMBER 15	1.76109E-05	0.6700746	0.1279997	0.711545032
1423494_at	2310042E22RIK	RIKEN CDNA 2310042E22 GENE	1.81197E-05	0.3253714	0.1926436	2.095673208
1433888_at	Atp2b2	ATPase, Ca++ transporting, plasma membrane 2	1.92616E-05	0.010892	0.1053032	1.295724966
1456656_at	Lin7a	lin-7 homolog A (C. elegans)	2.04536E-05	0.02926636	0.7919776	1.583414364
1436858_at	MBNL2	MUSCLEBLIND-LIKE 2	2.08473E-05	2.79143E-12	0.4221382	1.0846019
1419230_at	Krt12	keratin 12	2.14864E-05	0.08964405	0.1089164	1.957100255
1457311_at	Camk2a	calcium/calmodulin-dependent protein kinase II alpha	2.15093E-05	0.514289	0.5180424	0.545500381
1438019_at	IPPK	INOSITOL 1,3,4,5,6-PENTAKISPHOSPHATE 2-KINASE	2.28765E-05	0.002168871	0.05975466	0.755469655
1423233_at	CEBPD	CCAAT/ENHANCER BINDING PROTEIN (C/EBP), DELTA	2.30463E-05	0.08577387	0.658632	0.510859736
1445124_at	A930009L07RIK	RIKEN CDNA A930009L07 GENE	2.48877E-05	0.02787981	0.3508091	0.661165234
1428397_at	B3galt5	UDP-Gal:betaGlcNAc beta 1,3-galactosyltransferase, polypeptide 5	2.6742E-05	0.0354575	0.9144467	0.722238905
1450195_at	Ndst4	N-deacetylase/N-sulfotransferase (heparin glucosaminyl) 4	2.87635E-05	0.04425084	0.5428699	1.431482709
1451753_at	Plxna2	plexin A2	3.06327E-05	2.02572E-05	0.1126331	1.250933301
1440099_at	---	---	3.22951E-05	0.001093931	0.07069256	0.336751923
1459626_at	---	---	3.31418E-05	0.1263535	0.09027028	2.137346379
1429403_x_at	Glt8d2	glycosyltransferase 8 domain containing 2	3.49897E-05	0.281869	0.366182	0.530489975
1435705_at	LOC232875	SIMILAR TO HYPOTHETICAL PROTEIN FLJ12895	3.62119E-05	0.1875445	0.4534812	0.675737377
1429191_at	Dhx33	DEAH (Asp-Glu-Ala-His) box polypeptide 33	3.66729E-05	0.0433907	0.7136216	0.630418735
1451547_at	0610009A07RIK	RIKEN CDNA 0610009A07 GENE	3.72678E-05	0.00271349	0.2846474	0.604340175
1431230_a_at	Btbd9	BTB (POZ) domain containing 9	3.75617E-05	3.83021E-08	0.2864805	0.629987112
1445473_at	Azi2 /// Diras2	5-azacytidine induced gene 2 /// DIRAS family, GTP-binding RAS-like 2	3.86242E-05	0.000449719	0.1423418	1.56843149
1438852_x_at	Mcm6	minichromosome maintenance deficient 6 (MIS5 homolog, S. pombe) (S. cerevisiae)	3.88643E-05	0.4735671	0.1769227	0.474995862
1437075_at	FRMD3	FERM DOMAIN CONTAINING 3	4.0997E-05	0.3159474	0.9996335	2.56703778
1456051_at	Drd1a	dopamine receptor D1A	4.39717E-05	0.9246468	0.064316929	1.919175514
1455271_at	ALKBH3	ALKB, ALKYLATION REPAIR HOMOLOG 3 (E. COLI)	4.63193E-05	0.05782602	0.2820479	0.558953874
1448246_at	HDAC1	HISTONE DEACETYLASE 1 PSEUDOGENE	4.65453E-05	0.5060697	0.761297	0.71101007
1441380_at	2810439F02Rik	RIKEN cDNA 2810439F02 gene	4.77743E-05	0.3391013	0.4090777	0.639190851
1436634_at	ROBO3	ROUNDABOUT HOMOLOG 3 (DROSOPHILA)	4.89428E-05	0.1028577	0.6389669	1.953262982
1437528_x_at	A730017C20Rik	RIKEN cDNA A730017C20 gene	5.24187E-05	0.2076285	0.3264134	0.501343859
1437292_at	A330019N05Rik	RIKEN cDNA A330019N05 gene	5.36772E-05	0.01694393	0.6222912	0.594834272
1437746_at	Lrrtm1	leucine rich repeat transmembrane neuronal 1	5.56152E-05	0.1028321	0.1133861	0.584850192
1415993_at	SQLE	SQUALENE EPOXIDASE	5.8865E-05	0.07614893	0.1540821	1.251264001
1417504_at	Calb1	calbindin-28K	5.9859E-05	0.1271919	0.9634228	0.553889343
1439808_at	A130090K04Rik	RIKEN cDNA A130090K04 gene	6.08101E-05	0.6259474	0.4806006	2.093092013
1437371_at	9930021J17RIK	RIKEN CDNA 9930021J17 GENE	6.59555E-05	0.1508854	0.1915238	0.277400172
1444441_at	---	---	6.79411E-05	0.3486813	0.8289593	0.610046751
1418478_at	LMO1	LIM DOMAIN ONLY 1	6.88733E-05	0.4004544	0.9891043	0.601495483
1415698_at	GOLPH2	GOLGI PHOSPHOPROTEIN 2	6.89265E-05	0.01141514	0.2942591	0.597555311
1448424_at	Frzb	frizzled-related protein	6.97283E-05	0.000927996	0.7418769	0.541170951
1438428_at	Jph1	Junctophilin 1	7.0928E-05	0.01057784	0.5829174	0.550272676
1451280_at	Arpp21	cyclic AMP-regulated phosphoprotein, 21	7.65527E-05	0.01718104	0.1428043	1.411211532
1425060_s_at	Rsn	restin (Reed-Steinberg cell-expressed intermediate filament-associated protein)	7.83234E-05	0.02299296	0.09897452	1.480842679
1441253_at	Rfx3	Regulatory factor X, 3 (influences HLA class II expression)	7.87644E-05	0.003960258	0.2016817	0.565787208
1437360_at	Pcdh19	protocadherin 19	8.74855E-05	0.3298968	0.616656	0.669150178
1443322_at	---	---	8.87328E-05	0.03017075	0.9441813	0.513674237
1445130_at	AA407111	EXPRESSED SEQUENCE AA407111	9.15056E-05	0.06632708	0.2128353	2.431352147
1441559_at	LOC627626	similar to CG11212-PA	9.25296E-05	0.4618652	0.5257065	1.791444478
1425850_a_at	Nek6 /// LOC674247	NIMA (never in mitosis gene a)-related expressed kinase 6 /// similar to NIMA (never in mitosis gene a)-related expressed kinase 6	9.75892E-05	0.000567357	0.5580133	0.710904156
1429214_at	ADAMTSL2	ADAMTS-LIKE 2	0.00010128	0.2964585	0.2721882	0.664932408
1440084_at	AI663975	Expressed sequence AI663975	0.000109254	0.000571912	0.2748655	0.777550061
1453002_at	Sox11	SRY-box containing gene 11	0.000111322	0.1059127	0.4284215	0.657091523
1420858_at	Pkia	protein kinase inhibitor, alpha	0.000114875	1.05464E-05	0.2222794	0.701990306
1434130_at	Lhfpl2	lipoma HMGIC fusion partner-like 2	0.000117179	0.01195758	0.9462231	0.705879186
1443491_at	Ptprk	Protein tyrosine phosphatase, receptor type, K	0.000121423	9.74339E-06	0.5934554	1.538146607
1437385_at	Ccbe1	collagen and calcium binding EGF domains 1	0.000121926	0.7170577	0.6877613	0.426224392
1425533_a_at	STAU2	STAUFEN (RNA BINDING PROTEIN) HOMOLOG 2 (DROSOPHILA)	0.000126952	0.3140577	0.399322	1.298227401
1456044_at	NEDD4L	NEURAL PRECURSOR CELL EXPRESSED, DEVELOPMENTALLY DOWN-REGULATED GENE 4-LIKE	0.000131526	0.07770524	0.1830045	0.686877681
1447115_at	C78409	EXPRESSED SEQUENCE C78409	0.000136362	0.08247098	0.1445856	0.421962785
1448547_at	Rassf3	Ras association (RalGDS/AF-6) domain family 3	0.000143367	0.05411048	0.3487955	1.426797763
1444298_at	A130090K04Rik	RIKEN cDNA A130090K04 gene	0.000150963	0.2292643	0.5190685	3.140915895
1417363_at	ZFP61	ZINC FINGER PROTEIN 61	0.00015786	0.3998465	0.8782383	0.773509118
1454847_at	Lhfpl2	lipoma HMGIC fusion partner-like 2	0.000160311	0.07481739	0.403673	0.79629746
1421225_a_at	Slc4a4	solute carrier family 4 (anion exchanger), member 4	0.000164502	0.9012077	0.6843878	0.538814963
1424945_at	Chrdl1	chordin-like 1	0.000164547	0.3700796	0.1683964	2.332701434
1448978_at	NGEF	NEURONAL GUANINE NUCLEOTIDE EXCHANGE FACTOR	0.000166422	0.3104113	0.354776	1.698189172
1437876_at	FNDC6	FIBRONECTIN TYPE III DOMAIN CONTAINING 6	0.000168124	0.2082334	0.2338692	0.27719959
1429100_at	2010109K11Rik	RIKEN cDNA 2010109K11 gene	0.000170879	0.04298721	0.879413	0.669826886
1438193_at	Nrxn3	neurexin III	0.000174919	2.22545E-10	0.05754492	1.153118194
1448553_at	MYH7	MYOSIN, HEAVY POLYPEPTIDE 7, CARDIAC MUSCLE, BETA	0.0001754	0.3142843	0.734748	1.805521288
1445380_at	A230072C01RIK	RIKEN CDNA A230072C01 GENE	0.000177714	0.6260711	0.2800188	0.820799274
1439015_at	Gfra1	glial cell line derived neurotrophic factor family receptor alpha 1	0.000178537	0.002221515	0.0878148	0.706177878
1436464_at	---	13 days embryo heart cDNA, RIKEN full-length enriched library, clone:D330003D13 product:unclassifiable, full insert sequence	0.000180159	4.11496E-06	0.2855587	0.578294805
1440273_at	---	---	0.000192573	0.2872523	0.9562602	0.557440405
1450366_at	HRK	HARAKIRI, BCL2 INTERACTING PROTEIN (CONTAINS ONLY BH3 DOMAIN)	0.000207151	0.05527394	0.05062386	0.651451498
1440564_at	Prokr2	prokineticin receptor 2	0.000210698	0.04409448	0.686814	1.869396481
1431375_s_at	Parva	parvin, alpha	0.000216504	0.3833025	0.2020547	1.402296132
1416402_at	Abcb10	ATP-binding cassette, sub-family B (MDR/TAP), member 10	0.000220751	0.007810955	0.878277	0.690288162
1448326_a_at	CRABP1	CELLULAR RETINOIC ACID BINDING PROTEIN I	0.000227183	0.001420792	0.1702345	2.074396642
1452123_s_at	Frmd4b	FERM domain containing 4B	0.000234135	0.467406	0.3125	0.694976254
1431936_a_at	NEU2	NEURAMINIDASE 2	0.000246787	0.2514314	0.7058029	2.658928819
1420938_at	Hs6st2	heparan sulfate 6-O-sulfotransferase 2	0.000250175	0.04581173	0.06990693	1.407330305
1435411_at	---	Transcribed locus	0.000251419	0.05595245	0.66936949	0.749099134
1457155_at	ALDH2	ALDEHYDE DEHYDROGENASE 2, MITOCHONDRIAL	0.000254672	6.12219E-05	0.06899051	0.735065828

1417355_at	PEG3	PATERNALLY EXPRESSED 3	0.000260316	0.05291179	0.1889314	1.218290896
1442445_at	2610027H17RIK	RIKEN CDNA 2610027H17 GENE	0.000260789	9.45933E-05	0.3298047	0.62369538
1456093_at	ZFP536	ZINC FINGER PROTEIN 536	0.000262094	0.2270198	0.3568086	1.40697566
1454966_at	Itga8	integrin alpha 8	0.000265307	0.181105	0.6313862	0.574379585
1452703_at	4631427C17Rik	RIKEN cDNA 4631427C17 gene	0.000266559	0.2425378	0.6833427	0.686381194
1441166_at	A330050F15Rik	RIKEN cDNA A330050F15 gene	0.000282624	0.2647657	0.8102525	0.724306381
1429022_at	ADCYAP1R1	ADENYLATE CYCLASE ACTIVATING POLYPEPTIDE 1 RECEPTOR 1	0.000283561	0.5911502	0.3499483	0.743535617
1416756_at	DNAJB1	DNAJ (HSP40) HOMOLOG, SUBFAMILY B, MEMBER 1	0.000292458	0.3517747	0.187475	1.250620749
1447340_at	4631408O11RIK	RIKEN CDNA 4631408O11 GENE	0.000302296	0.2195051	0.06033197	2.062618622
1448698_at	Ccnd1	cyclin D1	0.000302403	0.393879	0.7540753	1.37506983
1423596_at	Nek6 /// LOC674247	NIMA (never in mitosis gene a)-related expressed kinase 6 /// similar to NIMA (never in mitosis gene a)-related expressed kinase 6	0.000306777	0.000724369	0.7444122	0.714109482
1451534_at	SCGN	SECRETAGOGIN, EF-HAND CALCIUM BINDING PROTEIN	0.000317693	0.07306503	0.6304236	0.667113168
1442157_at	A930041H05RIK	RIKEN CDNA A930041H05 GENE	0.000330141	0.002763003	0.1362671	1.441245159
1457558_at	A330050F15Rik	RIKEN cDNA A330050F15 gene	0.000330641	0.28354059	0.4543908	0.562228026
1447491_at	LRRC8D	LEUCINE RICH REPEAT CONTAINING 8D	0.000337316	0.000674619	0.1577863	0.801824396
1428602_at	Kcnj9	potassium inwardly-rectifying channel, subfamily J, member 9	0.000337425	0.02328887	0.087541	1.33122311
1438326_at	3300001M20RIK	RIKEN CDNA 3300001M20 GENE	0.000338907	8.18806E-09	0.2939136	0.765387133
1416783_at	TAC1	TACHYKININ 1	0.000342519	4.27467E-06	0.1912741	0.779541252
1444422_at	Pcdh19	protocadherin 19	0.000348332	0.01025842	0.9653787	0.749036762
1459619_at	EPB4.1L2	ERYTHROCYTE PROTEIN BAND 4.1-LIKE 2	0.000350994	0.6647238	0.7158628	1.47049762
1419730_at	4631427C17Rik	RIKEN cDNA 4631427C17 gene	0.000356659	0.2054534	0.7107432	0.530823127
1440056_at	---	---	0.000357627	0.5241263	0.7801649	0.667314382
1435311_s_at	Fbxo7	F-box only protein 7	0.000363407	0.2667043	0.39325	1.361893487
1417290_at	LRG1	LEUCINE-RICH ALPHA-2-GLYCOPROTEIN 1	0.000370403	0.6892512	0.9548202	3.89275294
1434195_at	PRSS35	PROTEASE, SERINE, 35	0.000372919	0.5365725	0.5729582	0.656345332
1417625_s_at	CMKOR1	CHEMOKINE ORPHAN RECEPTOR 1	0.000384652	0.5330373	0.7321008	0.616383672
1437060_at	OLFM4	OLFACTOMEDIN 4	0.000399851	0.7797704	0.494543	0.645849096
1449070_x_at	Apcdd1	adenomatosis polyposis coli down-regulated 1	0.000405235	0.003445898	0.2586282	0.6832348
1438450_at	Lin7a	lin-7 homolog A (C. elegans)	0.00044516	0.3590395	0.08866436	1.459101335
1420009_at	WWC1	CDNA SEQUENCE BC037006	0.000446985	0.8091927	0.9768587	3.246228347
1438075_at	FEM1C	FEM-1 HOMOLOG C (C.ELEGANS)	0.000448166	0.1587452	0.1464907	1.283708176
1437568_at	MMP16	MATRIX METALLOPEPTIDASE 16	0.000448307	9.42702E-05	0.6664409	0.772551022
1418304_at	PCDH21	PROTOCADHERIN 21	0.00045411	0.2810025	0.5533385	0.592212374
1431393_at	SIX6OS1	RIKEN CDNA 4921504I02 GENE	0.000458129	0.006326523	0.5107176	0.657859618
1431905_s_at	4933427G17Rik	RIKEN cDNA 4933427G17 gene	0.000458758	0.1267248	0.8708775	0.559363798
1443225_at	ACVR1C	ACTIVIN A RECEPTOR, TYPE IC	0.000460009	0.9352097	0.5705102	1.77659807
1433939_at	A730046J16	HYPOTHETICAL PROTEIN A730046J16	0.000460733	0.2694786	0.6314214	1.317655128
1421471_at	NPY1R	NEUROPEPTIDE Y RECEPTOR Y1	0.000467232	0.4101227	0.8948635	1.631051881
1439689_at	9030224M15RIK	RIKEN CDNA 9030224M15 GENE	0.00046992	0.001180232	0.05605254	1.333392111
1442614_at	Il1rap	interleukin 1 receptor accessory protein	0.000476572	0.07927656	0.3360559	0.697044849
1428657_at	Rreb1	ras responsive element binding protein 1	0.00048732	0.2699668	0.8661403	0.577384327
1457729_at	GPR12	G-PROTEIN COUPLED RECEPTOR 12	0.000509568	0.9096535	0.4783524	0.73202285
1419231_s_at	Krt12	keratin 12	0.000511281	0.792182	0.1878026	1.740404103
1440215_at	C130086A10	HYPOTHETICAL PROTEIN C130086A10	0.000517376	0.03162541	0.6065104	0.710720872
1443196_at	---	---	0.000523461	0.006979651	0.1188838	1.663151737
1439019_at	Fras1	Fraser syndrome 1 homolog (human)	0.000525175	0.8039105	0.9586032	1.477698967
1451972_at	Glcci1	glucocorticoid induced transcript 1	0.000526784	0.7224774	0.7650407	1.349952898
1420402_at	Atp2b2	ATPase, Ca++ transporting, plasma membrane 2	0.000528526	0.002150443	0.206829	1.580263223
1438309_at	---	---	0.000535424	0.309216	0.3642143	1.817579391
1427450_x_at	Myo1b	myosin IB	0.000544434	0.07649313	0.31834	1.514965066
1454818_at	GMEB2	GLUCOCORTICOID MODULATORY ELEMENT BINDING PROTEIN 2	0.000557182	0.9529804	0.3157971	1.274375207
1437375_at	---	0 day neonate thymus cDNA, RIKEN full-length enriched library, clone:A430010E21 product:unclassifiable, full insert sequence	0.000565806	0.3820279	0.6825714	0.502729749
1429591_at	TACC1	TRANSFORMING, ACIDIC COILED-COIL CONTAINING PROTEIN 1	0.000572051	8.16425E-05	0.7377495	1.29908053
1445297_at	---	---	0.000575949	0.1113447	0.1891607	1.224837951
1458010_at	---	---	0.000576339	0.003577188	0.3756734	1.544224137
1456722_at	Chrdl1	chordin-like 1	0.000587863	0.7627504	0.5689023	1.884424643
1437247_at	FOSL2	FOS-LIKE ANTIGEN 2	0.000587934	0.5597199	0.4454171	1.795867386
1418382_at	Apcdd1	adenomatosis polyposis coli down-regulated 1	0.00058983	0.6193384	0.6028065	0.711575455
1418810_at	Crhr1	corticotropin releasing hormone receptor 1	0.000589948	0.6360034	0.4078086	1.573111514
1441610_at	N28178	expressed sequence N28178	0.000595041	0.7968254	0.2866748	1.218206338
1442174_at	Tspan18	tetraspanin 18	0.00059779	0.1446278	0.7053649	0.621587094
1417676_a_at	Ptpro	protein tyrosine phosphatase, receptor type, O	0.000600938	0.000344671	0.3586915	1.281644177
1429123_at	RAB27A	RAB27A, MEMBER RAS ONCOGENE FAMILY	0.000602793	0.7988219	0.728725	0.630736189
1444492_at	PTPRD	PROTEIN TYROSINE PHOSPHATASE, RECEPTOR TYPE, D	0.000605742	0.04267448	0.1569284	1.560922793
1448989_a_at	Myo1b	myosin IB	0.000614674	0.02372215	0.1858923	1.409261497
1426972_at	SEC24D	SEC24 RELATED GENE FAMILY, MEMBER D (S. CEREVISIAE)	0.00061969	0.08829215	0.08010835	0.650566594
1443930_at	BC052046	cDNA sequence BC052046	0.000625838	0.03445614	0.9206352	0.653952653
1453076_at	9130211I03RIK	RIKEN CDNA 9130211I03 GENE	0.00062584	0.154908	0.5379484	2.112116707
1417300_at	SMPDL3B	SPHINGOMYELIN PHOSPHODIESTERASE, ACID-LIKE 3B	0.00063486	0.7741984	0.7602493	0.533146654
1430237_at	CLDN22	CLAUDIN 22	0.000638248	0.5325018	0.9598387	0.662004293
1450712_at	Kcnj9	potassium inwardly-rectifying channel, subfamily J, member 9	0.000645581	0.3166965	0.2176285	1.541646573
1429982_at	4933426K21RIK	RIKEN CDNA 4933426K21 GENE	0.000647113	0.05212072	0.861086	0.659822201
1451970_at	E330036I19RIK	RIKEN CDNA E330036I19 GENE	0.000654573	0.2148321	0.1961528	1.37211871
1452110_at	MTRR	5-METHYLTETRAHYDROFOLATE-HOMOCYSTEINE METHYLTRANSFERASE REDUCTASE	0.000664652	0.01681263	0.08085038	0.822499495
1460353_at	TMEM48	TRANSMEMBRANE PROTEIN 48	0.000665179	0.000977746	0.752787	0.738943516
1423306_at	2010002N04RIK	RIKEN CDNA 2010002N04 GENE	0.000670145	0.9393164	0.1547886	0.780381582
1440968_at	RASSF5	RAS ASSOCIATION (RALGDS/AF-6) DOMAIN FAMILY 5	0.000681455	0.6623367	0.07076725	0.680398585
1426083_a_at	Btg1	B-cell translocation gene 1, anti-proliferative	0.000727815	0.4067262	0.2059762	0.618457638
1426167_a_at	Camk4	calcium/calmodulin-dependent protein kinase IV	0.000729095	0.03167661	0.1627457	1.354461238
1453263_at	MAK10	MAK10 HOMOLOG, AMINO-ACID N-ACETYLTRANSFERASE SUBUNIT, (S. CEREVISIAE)	0.000745763	0.00050344	0.1379038	1.140544558
1418743_a_at	Tesc	tescalcin	0.000762736	0.53811279	0.2028326	1.412664931
1441608_at	---	---	0.000770032	0.01580723	0.4709601	1.366679068
1458177_at	EBPL	EMOPAMIL BINDING PROTEIN-LIKE	0.000771717	0.3252931	0.1033491	0.628824032
1459602_at	CCNI	CYCLIN I	0.000778714	0.237446	0.09979429	1.36389945
1446332_at	Pcdhgc3	Protocadherin gamma subfamily C, 3	0.000779902	0.210633	0.2295728	1.554208568
1456397_at	Cdh4	cadherin 4	0.000809052	0.8647249	0.8462554	1.411949
1450520_at	CACNG3	CALCIUM CHANNEL, VOLTAGE-DEPENDENT, GAMMA SUBUNIT 3	0.000835362	0.01417108	0.07709662	1.255803475
1444338_at	---	---	0.000840265	0.002769319	0.1686604	1.865171001
1438188_x_at	SLC25A29	SOLUTE CARRIER FAMILY 25 (MITOCHONDRIAL CARRIER, PALMITOYLCARNITINE TRANSPORTER), MEMBER 29	0.000856135	7.34496E-06	0.9966446	0.842992478
1452870_at	---	---	0.000864448	0.8996363	0.08363088	0.647865487
1426218_at	Glcci1	glucocorticoid induced transcript 1	0.000880661	0.6065454	0.2295962	1.411001585
1459358_at	Pkp2	Plakophilin 2	0.000889043	0.005023525	0.1706165	0.688100209
1444835_at	BC030499	CDNA SEQUENCE BC030499	0.000894683	0.4356973	0.1253121	2.126052352
1417215_at	RAB27B	RAB27B, MEMBER RAS ONCOGENE FAMILY	0.000898661	0.8063673	0.8953132	0.688614762
1419748_at	ABCD2	ATP-BINDING CASSETTE, SUB-FAMILY D (ALD), MEMBER 2	0.000909502	0.01275477	0.3841873	1.247979557
1422825_at	CART	COCAINE AND AMPHETAMINE REGULATED TRANSCRIPT	0.000921101	0.0990048	0.9331054	0.61744792
1444956_at	---	---	0.000924746	0.9072413	0.3961552	0.738442586
1441693_at	ADAMTS3	ADAM METALLOPEPTIDASE WITH THROMBOSPONDIN TYPE 1 MOTIF, 3	0.000937849	0.003166711	0.4835131	0.71604599
1447907_x_at	Apba2bp	Amyloid beta (A4) precursor protein-binding, family A, member 1 binding protein	0.00095392	0.1488966	0.9157765	1.932293455
1453321_at	FNDC1	FIBRONECTIN TYPE III DOMAIN CONTAINING 1	0.00095838	0.4910492	0.2392053	0.683814124
1460619_at	4931419K03RIK	RIKEN CDNA 4931419K03 GENE	0.000996492	0.62524329	0.9171043	0.752370651
1444633_at	---	---	0.001019597	0.7397393	0.6336044	1.94452512
1436200_at	LONRF3	LON PEPTIDASE N-TERMINAL DOMAIN AND RING FINGER 3	0.001031352	0.9121484	0.8039801	0.79396473
1446802_at	---	---	0.001034193	0.01948977	0.08872428	1.212302926
1433558_at	DAB2IP	DISABLED HOMOLOG 2 (DROSOPHILA) INTERACTING PROTEIN	0.001036356	5.63921E-06	0.4431257	1.344244928
1449205_at	OVOL2	RIKEN CDNA 1810007D21 GENE	0.001048296	0.003816281	0.06631763	1.468091901
1427049_s_at	SMO	SMOOTHENED HOMOLOG (DROSOPHILA)	0.001050152	0.553677	0.9547994	0.747461136
1416623_at	THBS3	THROMBOSPONDIN 3	0.001056748	5.54418E-07	0.1487686	0.696509771
1420916_at	PRPF40A	PRE-MRNA PROCESSING FACTOR 40 HOMOLOG A (YEAST)	0.001057581	0.06024302	0.3021323	0.80987554
1451458_at	Tmem2	transmembrane protein 2	0.001061904	2.65636E-06	0.1199951	1.121932921
1451260_at	ALDH1B1	ALDEHYDE DEHYDROGENASE 1 FAMILY, MEMBER B1	0.001097577	0.1991193	0.4835122	0.659962357
1454656_at	Spata13	spermatogenesis associated 13	0.001099452	0.1572385	0.2534262	1.705851
1451809_s_at	Rwdd3	RWD domain containing 3	0.001107407	0.4913359	0.1796123	0.59014995
1456048_at	CPEB3	CYTOPLASMIC POLYADENYLATION ELEMENT BINDING PROTEIN 3	0.001114794	0.8214491	0.2456117	1.150375789
1429430_at	---	---	0.001123937	6.36313E-05	0.5486808	0.785737918
1434769_at	Btbd9	BTB (POZ) domain containing 9	0.001128529	0.8434598	0.61148329	0.739955964
1436839_at	---	---	0.001140201	0.1493749	0.8325577	0.554047544
1433762_at	C630043F03RIK	RIKEN CDNA C630043F03 GENE	0.001141293	0.5915534	0.9637301	0.673775832
1433793_s_at	Nrip2	nuclear receptor interacting protein 2	0.001154277	0.2265338	0.7680679	0.656134359
1432360_a_at	Tmtc4	transmembrane and tetratricopeptide repeat containing 4	0.001157633	0.5699465	0.6108644	0.770240553
1460447_at	Pus7l	pseudouridylate synthase 7 homolog (S. cerevisiae)-like	0.001176564	0.6803296	0.507961	0.643875893
1421318_at	Ndst4	N-deacetylase/N-sulfotransferase (heparin glucosaminyl) 4	0.001194321	0.07733214	0.9861193	1.409456818
1422539_at	EXTL2	EXOTOSES (MULTIPLE)-LIKE 2	0.00119918	0.03588431	0.9097782	0.652580997
1416895_at	EFNA1	EPHRIN A1	0.001205047	0.006519054	0.7662269	0.657839967
1417877_at	2310005P05RIK	RIKEN CDNA 2310005P05 GENE	0.001211299	0.05017766	0.759454	0.734768358
1459847_x_at	Gfra2	glial cell line derived neurotrophic factor family receptor alpha 2	0.001217423	0.001708824	0.6354227	0.714496321
1432289_a_at	JSRP1	RIKEN CDNA 2300003C06 GENE	0.001224004	0.064215869	0.777097	1.394476225
1417784_at	Als2	amyotrophic lateral sclerosis 2 (juvenile) homolog (human)	0.001241354	0.1988736	0.1005594	0.755594525
1429164_at	PRSS36	PROTEASE, SERINE, 36	0.001249599	0.2649862	0.1670568	0.826172867
1419072_at	GSTM7	GLUTATHIONE S-TRANSFERASE, MU 7	0.001273227	0.915471	0.3060907	0.671500737
1425213_at	6430514L14RIK	RIKEN CDNA 6430514L14 GENE	0.001274394	0.03851793	0.1927553	1.18053319
1434777_at	Mycl1	v-myc myelocytomatosis viral oncogene homolog 1, lung carcinoma derived (avian)	0.001274455	0.03267041	0.741472	0.6035368
1431074_a_at	Pitpnc1	phosphatidylinositol transfer protein, cytoplasmic 1	0.00128031	0.03292087	0.06018238	1.196797418
1430167_a_at	Rwdd3	RWD domain containing 3	0.001286034	0.01616284	0.6803485	0.587522251
1452974_at	NOL8	NUCLEOLAR PROTEIN 8	0.00130408	0.01009497	0.1805941	0.83879293
1429372_at	Sox11	SRY-box containing gene 11	0.001349283	0.000594926	0.5713623	0.726946704
1447863_s_at	NR4A2	NUCLEAR RECEPTOR SUBFAMILY 4, GROUP A, MEMBER 2	0.001352898	0.1915886	0.3048197	1.227879903
1451043_at	Nek6	NIMA (never in mitosis gene a)-related expressed kinase 6	0.001360992	0.01453319	0.3091818	0.576400787
1434107_at	SPATA2	SPERMATOGENESIS ASSOCIATED 2	0.001368416	0.07530943	0.5299585	0.722402023
1448554_s_at	MYH6	MYOSIN, HEAVY POLYPEPTIDE 6, CARDIAC MUSCLE, ALPHA	0.001370883	0.2982653	0.9363205	1.940223189
1432931_at	---	---	0.001376018	3.48855E-05	0.1230212	1.432734011
1459632_at	CRIM1	CYSTEINE RICH TRANSMEMBRANE BMP REGULATOR 1 (CHORDIN LIKE)	0.001401186	0.02989806	0.08670433	1.395107593
1455720_at	ADAMTS2	A DISINTEGRIN-LIKE AND METALLOPEPTIDASE (REPROLYSIN TYPE) WITH THROMBOSPONDIN TYPE 1 MOTIF, 2	0.001412114	0.1856033	0.990388	0.749782954
1435444_at	ATF6	ACTIVATING TRANSCRIPTION FACTOR 6	0.001453501	0.02934134	0.7093555	0.804449105
1456603_at	1500005K14Rik	RIKEN cDNA 1500005K14 gene	0.001459079	0.2090189	0.9435484	0.566207368
1417051_at	PCDH8	PROTOCADHERIN 8	0.001463606	0.04807032	0.9731635	0.764442288
1454653_at	CPNE9	COPINE FAMILY MEMBER IX	0.001465008	0.517414799	0.6768533	1.744410294
1449740_s_at	Dsg2	desmoglein 2	0.001474276	0.1852935	0.9293622	0.58455043
1427068_x_at	4933439F18Rik	RIKEN cDNA 4933439F18 gene	0.001477424	0.9438037	0.5394977	0.663741804
1431164_at	Rragd	Ras-related GTP binding D	0.001487009	0.001747012	0.09848617	1.435628684
1434447_at	AI838057	EXPRESSED SEQUENCE AI838057	0.00153985	0.6071353	0.5230982	1.580113855
1458469_at	Cblb	Casitas B-lineage lymphoma b	0.00154446	0.001004628	0.4820008	0.666170253
1430929_at	9430082L08RIK	RIKEN CDNA 9430082L08 GENE	0.001550574	0.301402	0.8582124	1.591745727
1437695_at	Prokr2	prokineticin receptor 2	0.001579836	0.6613693	0.2997749	1.55051961
1438995_at	6330500D04RIK	RIKEN CDNA 1700108N18 GENE	0.001585629	0.6001647	0.06639939	0.729843539
1451474_a_at	PARP8	POLY (ADP-RIBOSE) POLYMERASE FAMILY, MEMBER 8	0.001596286	0.1255726	0.728961	1.412610023
1450893_a_at	UBAP1	UBIQUITIN-ASSOCIATED PROTEIN 1	0.001613209	1.94066E-06	0.2203785	1.075713528
1429556_at	2610024B07RIK	RIKEN CDNA 2610024B07 GENE	0.001619452	0.321131	0.6858663	1.371327133
1444239_at	BC023488	CDNA SEQUENCE BC023488	0.001671215	0.7516396	0.437973	0.405333376

1437382_at	Acvr2a	Activin receptor IIA	0.001679687	0.04039179	0.73557	0.664644821
1423180_at	Kcnb1	potassium voltage gated channel, Shab-related subfamily, member 1	0.00173526	0.494451	0.485177	1.377454151
1447493_at	A530088H08Rik	RIKEN cDNA A530088H08 gene	0.001739215	0.116369	0.5590798	0.671127354
1437306_at	C130092O11RIK	RIKEN CDNA C130092O11 GENE	0.001746823	0.9945353	0.06938976	1.372005621
1453286_at	Plxna2	plexin A2	0.001751353	0.1140773	0.2992017	1.245545397
1429906_at	A930035E12RIK	RIKEN CDNA A930035E12 GENE	0.001783319	8.27359E-05	0.176593	1.469990979
1433699_at	TNFAIP3	TUMOR NECROSIS FACTOR, ALPHA-INDUCED PROTEIN 3	0.00182239	0.408289	0.6129867	0.764642987
1439073_at	ZFP160	RIKEN CDNA 6720480D16 GENE	0.001825262	0.6024086	0.07429413	1.335043577
1457496_at	---	---	0.001825464	0.005051657	0.3828893	1.581004519
1433792_at	Nrip2	nuclear receptor interacting protein 2	0.001831938	0.8315431	0.5822663	0.678171351
1436011_at	ELMO2	RIKEN CDNA 1190002F24 GENE	0.001849156	0.1586772	0.7680614	0.715474386
1437339_s_at	Pcsk5	proprotein convertase subtilisin/kexin type 5	0.001852866	0.9748423	0.4739355	0.510970972
1442603_at	Pb1	Polybromo 1	0.001862726	0.9587058	0.9589642	0.710964739
1444305_at	LOC381400	SIMILAR TO MYH7B PROTEIN	0.001866114	0.9390867	0.6052182	1.497084659
1458073_at	9330156P08RIK	RIKEN CDNA 9330156P08 GENE	0.001880347	0.2506359	0.1465677	1.817495672
1427416_x_at	LOC674944	similar to dual specificity phosphatase 7	0.001888665	0.2378063	0.4529077	1.381492971
1460300_a_at	LTK	LEUKOCYTE TYROSINE KINASE	0.001904364	0.03588079	0.09104937	1.259478148
1417419_at	Ccnd1	cyclin D1	0.001923435	0.9188458	0.621764	1.389226218
1433716_x_at	Gfra2	glial cell line derived neurotrophic factor family receptor alpha 2	0.00194143	0.6441185	0.9761226	0.589642247
1441370_at	TMCC1	HYPOTHETICAL PROTEIN LOC212904	0.001947073	0.000894833	0.3070357	1.324933469
1459552_at	D4BWG0951E	DNA SEGMENT, CHR 4, BRIGHAM & WOMEN'S GENETICS 0951 EXPRESSED	0.001969892	0.3453088	0.6862388	0.656198304
1425088_at	SCNN1A	SODIUM CHANNEL, NONVOLTAGE-GATED, TYPE I, ALPHA	0.001982165	0.001043262	0.3088074	0.665908904
1455250_at	Sh3bp4	SH3-domain binding protein 4	0.00198734	0.3002513	0.118991	1.251138571
1451421_a_at	ROGDI	LEUCINE ZIPPER DOMAIN PROTEIN	0.001993385	0.003358685	0.7618704	0.732542092
1423395_at	Tsnax	translin-associated factor X	0.001997468	0.01499031	0.1661535	1.311403876
1433893_s_at	Spag5	sperm associated antigen 5	0.002003174	0.4575088	0.5020399	1.451442549
1428946_at	UBE1L2	UBIQUITIN-ACTIVATING ENZYME E1-LIKE 2	0.002008689	0.06547071	0.9137541	1.35092604
1440765_at	Fras1	Fraser syndrome 1 homolog (human)	0.002044737	0.7797992	0.4975831	1.963584532
1421925_at	Mapk11	mitogen-activated protein kinase 11	0.002058871	0.2294072	0.7796376	1.431623477
1457198_at	Nrp1	neuropilin 1	0.002079462	0.001245739	0.065348279	0.789363602
1420611_at	PRKACB	PROTEIN KINASE, CAMP DEPENDENT, CATALYTIC, BETA	0.002115789	0.1391557	0.2352055	1.111996559
1424341_s_at	PCDHA9	PROTOCADHERIN ALPHA 9	0.002122168	0.3835737	0.1656558	0.90935388
1435564_at	C230078M08RIK	RIKEN CDNA C230078M08 GENE	0.002126732	0.02726122	0.7502737	0.789057125
1426908_at	Galnt7	UDP-N-acetyl-alpha-D-galactosamine: polypeptide N-acetylgalactosaminyltransferase 7	0.002143216	0.4654404	0.4462413	1.132348362
1432228_at	4933406B15RIK	RIKEN CDNA 4933406B15 GENE	0.002185774	0.2533959	0.4701839	0.575561987
1418847_at	ARG2	ARGINASE TYPE II	0.002199091	0.3186483	0.1644911	0.602924694
1440721_at	5930433N17RIK	RIKEN CDNA 5930433N17 GENE	0.00220886	0.1858834	0.3681371	1.418325688
1426797_at	2700094F01Rik	RIKEN cDNA 2700094F01 gene	0.002212024	0.9275622	0.6104745	0.800664296
1419426_s_at	CCL21C	CHEMOKINE (C-C MOTIF) LIGAND 21A	0.002214243	0.004695359	0.06865577	1.257463378
1456759_at	LRRC4C	LEUCINE RICH REPEAT CONTAINING 4C	0.00221461	0.4091444	0.2249322	1.256031505
1448686_at	Il16	interleukin 16	0.002226614	0.4718047	0.6928443	0.616264149
1440455_at	AI848599	EXPRESSED SEQUENCE AI848599	0.00224745	0.7606911	0.5163439	1.254294094
1450842_a_at	CENPA	CENTROMERE AUTOANTIGEN A	0.002254832	0.09196765	0.6285902	1.579245318
1445118_at	---	---	0.002279182	0.790276	0.5984705	0.32736631
1457137_at	---	---	0.002282962	0.8013571	0.2632338	1.380720997
1428990_at	2310047K21RIK	RIKEN CDNA 2310047K21 GENE	0.002305537	0.02004053	0.0849734	0.808641244
1427975_at	2210403B10RIK	RIKEN CDNA 2210403B10 GENE	0.002313581	0.01341933	0.9859467	0.710429032
1424310_at	MOCS2	MOLYBDENUM COFACTOR SYNTHESIS 2	0.002326232	0.09088144	0.2114025	0.827162368
1435959_at	Arhgap15	Rho GTPase activating protein 15	0.002335082	0.06743794	0.555227	0.608707133
1430407_at	3110035C09RIK	RIKEN CDNA 3110035C09 GENE	0.002362953	0.727983	0.8152098	1.505969828
1427742_a_at	KLF6	KRUPPEL-LIKE FACTOR 6	0.002371487	0.002319291	0.55285169	1.16806835
1438611_at	TUBA7	TUBULIN, ALPHA 3	0.002374351	0.4604048	0.3896162	2.448985558
1416818_at	Parva	parvin, alpha	0.002380197	0.008516735	0.2905245	1.42062797
1420806_at	FGF16	FIBROBLAST GROWTH FACTOR 16	0.002426912	0.4261487	0.6289859	1.403279081
1420667_at	Doc2b	double C2, beta	0.00243034	0.1555437	0.8012464	0.636104632
1433833_at	Fndc3b	fibronectin type III domain containing 3B	0.002432995	0.108788	0.1120496	1.181280738
1421991_a_at	Igfbp4	insulin-like growth factor binding protein 4	0.002476039	0.0656398	0.2700266	1.356177625
1426411_a_at	STRBP	SPERMATID PERINUCLEAR RNA BINDING PROTEIN	0.002479268	2.79779E-05	0.1111878	1.098033697
1431098_at	Rsn	restin (Reed-Steinberg cell-expressed intermediate filament-associated protein)	0.002483746	0.05127478	0.09270339	1.979517387
1418881_at	EFCBP2	EF HAND CALCIUM BINDING PROTEIN 2	0.002507072	0.01494399	0.6156061	1.409863762
1448303_at	GPNMB	GLYCOPROTEIN (TRANSMEMBRANE) NMB	0.002518961	0.7179739	0.3333186	0.628089784
1424987_at	5430435G22RIK	RIKEN CDNA 5430435G22 GENE	0.002520537	0.197925	0.06156917	1.389382316
1433758_at	NISCH	IMIDAZOLINE RECEPTOR I-1-LIKE PROTEIN	0.002529438	2.4959E-08	0.1152466	0.780764743
1449286_at	Ntng1	netrin G1	0.002564994	0.8277615	0.2170329	1.494316035
1455868_a_at	TUBGCP2	TUBULIN, GAMMA COMPLEX ASSOCIATED PROTEIN 2	0.002599971	0.000680276	0.461312	0.775526063
1455344_at	AW494418	EXPRESSED SEQUENCE AW494418	0.002606251	0.2321098	0.601785	0.787928748
1435577_at	DAB1	DISABLED HOMOLOG 1 (DROSOPHILA)	0.002610985	0.1561446	0.5692391	1.238087934
1421896_at	Elk1	ELK1, member of ETS oncogene family	0.002615438	0.2344754	0.9203218	0.634627217
1448545_at	Sdc2	syndecan 2	0.002618702	0.5133508	0.521022	1.253485655
1440555_at	Rragd	Ras-related GTP binding D	0.00262113	0.004942608	0.2931255	1.27670233
1459537_at	4631427C17Rik	RIKEN cDNA 4631427C17 gene	0.002636665	0.002740214	0.1944407	0.656831126
1420340_at	4930413O22RIK	RIKEN CDNA 4930413O22 GENE	0.002673497	0.006810979	0.1860981	0.834567836
1422552_at	RPRM	REPRIMO, TP53 DEPENDENT G2 ARREST MEDIATOR CANDIDATE	0.002742006	0.187218	0.3135224	0.741151593
1434690_at	LYCAT	LYSOCARDIOLIPIN ACYLTRANSFERASE	0.002748119	0.3716402	0.1947155	1.1223407
1421821_at	LDLR	LOW DENSITY LIPOPROTEIN RECEPTOR	0.002770765	0.4708911	0.206769	1.198338087
1427685_a_at	Synj2	synaptojanin 2	0.002791243	0.03214055	0.3202033	1.341507322
1456129_at	E030013G06RIK	RIKEN CDNA E030013G06 GENE	0.002800238	0.977969	0.613485	0.392914766
1425934_a_at	B4GALT4	UDP-GAL:BETAGLCNAC BETA 1,4-GALACTOSYLTRANSFERASE, POLYPEPTIDE 4	0.002803544	0.003356542	0.57326829	0.728274415
1451964_at	MIA2	MELANOMA INHIBITORY ACTIVITY 2	0.002819707	0.27799629	0.2331639	2.301042477
1425052_at	---	CDNA, clone:Y2G0147N17, strand:unspecified	0.002819952	0.28671029	0.245799	0.719336182
1441018_at	USP24	UBIQUITIN SPECIFIC PROTEASE 24	0.00284573	0.3471024	0.7409226	1.35198367
1438523_x_at	1500001A10RIK	RIKEN CDNA 1500001A10 GENE	0.002858532	0.2009917	0.7998469	0.47679978
1428398_at	B3galt5	UDP-Gal:betaGlcNAc beta 1,3-galactosyltransferase, polypeptide 5	0.002877781	0.05280458	0.6395714	0.794302137
1430703_at	LOC546515	SIMILAR TO KERATIN ASSOCIATED PROTEIN 9-1	0.002898796	0.321029	0.09418252	0.285149055
1427401_at	Chrna5	cholinergic receptor, nicotinic, alpha polypeptide 5	0.002899101	0.1387745	0.822963	0.669686132
1419722_at	KLK8	PROTEASE, SERINE, 19 (NEUROPSIN)	0.00291059	0.016436369	0.7605146	1.405990389
1419592_at	UNC5C	UNC-5 HOMOLOG C (C. ELEGANS)	0.00291112	0.00047012	0.09761327	1.145700513
1455629_at	Drd1a	dopamine receptor D1A	0.002914916	0.5967964	0.83413	1.788282514
1457193_at	---	---	0.002920767	0.6767904	0.2299507	1.227968673
1435645_at	MMD	MONOCYTE TO MACROPHAGE DIFFERENTIATION-ASSOCIATED	0.002936654	0.1592673	0.07663362	1.198660195
1435847_at	CDC42BPA	EXPRESSED SEQUENCE AI462003	0.002944464	0.02718867	0.3494781	1.129914876
1428991_at	HRASLS	HRAS-LIKE SUPPRESSOR	0.002970996	0.4975422	0.59881169	0.710739541
1434852_at	PLA2G2F	PHOSPHOLIPASE A2, GROUP IIF	0.002984579	0.3462078	0.064713839	0.355162199
1456242_at	---	---	0.00298491	0.0568951	0.2284971	0.362290461
1448496_a_at	ING1	INHIBITOR OF GROWTH FAMILY, MEMBER 1	0.002988581	2.19812E-08	0.1218883	1.071427648
1417111_at	Man1a	mannosidase 1, alpha	0.003004849	0.02159618	0.3630912	1.311586169
1428535_at	9430020K01RIK	RIKEN CDNA 9430020K01 GENE	0.003016259	0.1056557	0.7043294	1.574963887
1449175_at	GPR65	G-PROTEIN COUPLED RECEPTOR 65	0.003033385	0.7190088	0.06420755	2.443651606
1427038_at	PENK1	PREPROENKEPHALIN 1	0.003034564	0.52252	0.7818784	1.688526927
1427186_a_at	Mef2a	myocyte enhancer factor 2A	0.003037817	0.900066	0.2502638	0.812567834
1458539_at	R3hdm1	R3H domain 1 (binds single-stranded nucleic acids)	0.003040693	0.03973051	0.9672823	1.358874869
1459715_at	1500011J06RIK	RIKEN CDNA 1500011J06 GENE	0.003042151	0.01867443	0.5552006	0.637217
1437857_at	Dpy19l3	dpy-19-like 3 (C. elegans)	0.003066528	0.2170245	0.3233033	1.377710879
1429249_at	4833424O15Rik	RIKEN cDNA 4833424O15 gene	0.003073392	5.47805E-05	0.3033078	1.241136415
1417954_at	SST	SOMATOSTATIN	0.003076157	0.015761	0.5861447	0.609510262
1448249_at	Gpd1	glycerol-3-phosphate dehydrogenase 1 (soluble)	0.003077751	0.1808897	0.2706397	1.271984209
1418790_at	ZFP312	ZINC FINGER PROTEIN 312	0.003081384	0.1312621	0.5299336	1.331702517
1451469_at	D530005L17RIK	RIKEN CDNA B430108F07 GENE	0.003084337	0.003714741	0.06146618	0.869448562
1439647_at	LOC433698	HYPOTHETICAL LOC433698	0.003093695	0.4302166	0.4230974	1.199463685
1425606_at	SLC5A8	SOLUTE CARRIER FAMILY 5 (IODIDE TRANSPORTER), MEMBER 8	0.00309857	0.9131834	0.7273167	2.984182824
1444925_at	---	---	0.003104255	0.5705787	0.1060109	2.387366547
1428113_at	Tmtc4	transmembrane and tetratricopeptide repeat containing 4	0.003131439	0.2107715	0.1649961	0.663699406
1453670_at	USP50	UBIQUITIN SPECIFIC PEPTIDASE 50	0.003150383	0.5337185	0.2764112	0.459486826
1440484_at	UNC5D	UNC-5 HOMOLOG D (C. ELEGANS)	0.003179394	0.063618209	0.4369021	1.443164672
1429692_s_at	GCH1	GTP CYCLOHYDROLASE 1	0.003181096	0.01059714	0.2901472	0.715447818
1456331_at	9130023H24RIK	RIKEN CDNA 9130023H24 GENE	0.003205639	0.7380961	0.1592993	0.764235194
1448817_at	PLEKHH2	PLECKSTRIN HOMOLOGY DOMAIN CONTAINING, FAMILY H (WITH MYTH4 DOMAIN) MEMBER 2	0.003212564	0.06728341	0.1101765	0.656194044
1446324_at	---	---	0.00322183	0.03883305	0.5971817	1.327655768
1454303_at	EPN2	EPSIN 2	0.003250421	0.6251944	0.1175727	0.591931979
1427489_at	Itga8	integrin alpha 8	0.003301542	0.000816503	0.905123	0.660086071
1422072_a_at	GSTM6	GLUTATHIONE S-TRANSFERASE, MU 6	0.003308135	0.02476388	0.6619156	0.75982882
1429051_s_at	Sox11	SRY-box containing gene 11	0.003314051	1.04272E-05	0.9161658	0.701403093
1417012_at	Sdc2	syndecan 2	0.003331925	0.01032393	0.9612363	1.310241926
1448723_at	RDH7	RETINOL DEHYDROGENASE 7	0.003358421	0.093278	0.05781352	0.409506928
1458083_at	---	---	0.00336385	0.05009098	0.05658651	1.90384272
1421174_at	IRF4	INTERFERON REGULATORY FACTOR 4	0.003368042	0.0640432	0.07911675	0.642484253
1446218_at	PPFIA2	HYPOTHETICAL PROTEIN 5330438O12	0.003368691	0.4958418	0.6702543	1.986638476
1442555_at	KCND3	POTASSIUM VOLTAGE-GATED CHANNEL, SHAL-RELATED FAMILY, MEMBER 3	0.003392199	0.004527288	0.06156268	1.280636629
1436592_at	---	12 days embryo head cDNA, RIKEN full-length enriched library, clone:3010015L05 product:unclassifiable, full insert sequence	0.003393983	0.07215205	0.5242181	0.644059275
1439171_at	C530008M17RIK	RIKEN CDNA 4931405B09 GENE	0.003405197	0.8711078	0.6181848	2.404705763
1455528_at	---	---	0.003412261	0.6400464	0.6751271	0.712506799
1424638_at	CDKN1A	CYCLIN-DEPENDENT KINASE INHIBITOR 1A (P21)	0.003437554	0.6854168	0.6086116	1.53797426
1424682_at	ATPBD1C	ATP BINDING DOMAIN 1 FAMILY, MEMBER C	0.003455116	0.01693153	0.8174637	0.795421099
1430937_at	CKM	CREATINE KINASE, MUSCLE	0.003459121	0.003495149	0.1686819	0.720852496
1438673_at	---	---	0.00350411	0.09599864	0.8854986	0.708689076
1417045_at	BID	BH3 INTERACTING DOMAIN DEATH AGONIST	0.003504665	0.004899969	0.4857479	0.699790512
1418778_at	9030408N13RIK	RIKEN CDNA 9030408N13 GENE	0.003543487	0.09714983	0.9905313	0.771941898
1434261_at	SIPA1L2	SIGNAL-INDUCED PROLIFERATION-ASSOCIATED 1 LIKE 2	0.003543745	0.3099267	0.3518877	0.756425932
1455163_at	GUF1	EXPRESSED SEQUENCE AA407526	0.003546859	2.87038E-07	0.6340407	0.783306334
1457085_at	---	---	0.003549171	0.2132985	0.31757419	2.868473451
1450211_at	Lrp1b	low density lipoprotein-related protein 1B (deleted in tumors)	0.003582166	0.000337293	0.7427729	0.784089884
1444147_at	Pcsk2	proprotein convertase subtilisin/kexin type 2	0.00358407	0.03472364	0.2142042	1.512207985
1446769_at	2810439F02Rik	RIKEN cDNA 2810439F02 gene	0.003589706	0.64044749	0.4600712	0.725930178
1447478_at	Ran	RAN, member RAS oncogene family	0.003633216	0.01352357	0.4137897	0.642028599
1458981_at	LOC638482	WD REPEAT DOMAIN 70	0.003754236	0.4211288	0.4698688	4.249759634
1425277_at	SLIT1	SLIT HOMOLOG 1 (DROSOPHILA)	0.003761444	0.2338509	0.7560774	0.664565168
1427331_at	ADORA1	ADENOSINE A1 RECEPTOR	0.003783016	0.000651168	0.8330973	0.663777251
1444040_at	LAIR1	LEUKOCYTE-ASSOCIATED IG-LIKE RECEPTOR 1	0.00378879	0.134943	0.3674968	0.766040129
1417700_at	Rab38	Rab38, member of RAS oncogene family	0.003789033	2.14103E-05	0.8219266	1.419115818
1424958_at	CAR8	CARBONIC ANHYDRASE 8	0.003844253	0.01484913	0.2609167	1.495206288
1453668_at	3110052M02Rik	RIKEN cDNA 3110052M02 gene	0.003871664	0.371285	0.2958023	1.565133322
1457642_at	5730507N06RIK	RIKEN CDNA 5730507N06 GENE	0.003887885	0.375927	0.8077295	0.719811067
1441005_at	2610304G08RIK	RIKEN CDNA 2610304G08 GENE	0.003912406	0.1658566	0.1016846	0.744583894
1446859_at	---	---	0.003947272	0.1124189	0.6086746	2.337669619
1426765_at	COMMD7	COMM DOMAIN CONTAINING 7	0.003950688	0.7764203	0.9924573	0.869935038
1427686_at	LOC632660	VOMERONASAL 2, RECEPTOR, 13	0.003984711	0.07670351	0.15708639	0.232242561
1454729_at	TMEM108	RANSMEMBRANE PROTEIN 108	0.003986495	2.66073E-06	0.08206162	1.101846376
1422644_at	SH3BGR	SH3-BINDING DOMAIN GLUTAMIC ACID-RICH PROTEIN	0.003994171	0.4747887	0.1334671	1.776360426
1429844_at	2310043J07RIK	RIKEN CDNA 2310043J07 GENE	0.003998275	0.00126217	0.7465097	0.580348017

1427196_at	WNK4	WNK LYSINE DEFICIENT PROTEIN KINASE 4	0.004001409	0.8349315	0.711122	1.79183844
1434051_s_at	Hspa12a	heat shock protein 12A	0.004008756	0.54471309	0.1563803	1.215117514
1425413_at	Rfx3	regulatory factor X, 3 (influences HLA class II expression)	0.004038118	0.3978921	0.066285529	0.72909018
1438702_at	---	---	0.004106134	0.003107681	0.2411812	1.259830656
1430698_a_at	D14Ertd581e	DNA segment, Chr 14, ERATO Doi 581, expressed	0.004111237	0.8855833	0.65492379	0.718037277
1427925_at	STX17	SYNTAXIN 17	0.00411221	0.2895206	0.4633972	0.72867522
1428384_at	D4Bwg0951e	DNA segment, Chr 4, Brigham & Women's Genetics 0951 expressed	0.004116906	0.05845922	0.8342578	0.670386853
1447025_at	UBE2E2	UBIQUITIN-CONJUGATING ENZYME E2E 2 (UBC4/5 HOMOLOG, YEAST)	0.004120787	0.05483038	0.5151943	1.315888881
1458620_at	LOC332993	SIMILAR TO UBIQUITIN A-52 RESIDUE RIBOSOMAL PROTEIN FUSION PRODUCT 1	0.004128333	0.5620697	0.58762669	0.33442906
1419638_at	Efnb2	ephrin B2	0.004133247	0.3158265	0.50295	1.343660553
1457528_at	Slc4a7	solute carrier family 4, sodium bicarbonate cotransporter, member 7	0.004177594	0.00036293	0.7067562	0.61777437
1426115_a_at	Kcnj9	potassium inwardly-rectifying channel, subfamily J, member 9	0.004178497	0.8032124	0.824982	1.759440715
1455765_a_at	ABCC8	ATP-BINDING CASSETTE, SUB-FAMILY C (CFTR/MRP), MEMBER 8	0.004208127	0.000638697	0.5138617	0.676975609
1442095_at	Asxl3	additional sex combs like 3 (Drosophila)	0.004229411	0.2684004	0.07808693	1.1921268
1442837_at	---	---	0.004252799	0.62464109	0.809327	2.834500676
1422540_at	FBLN1	FIBULIN 1	0.004289857	0.003829405	0.8851269	0.614152771
1427177_at	Fyco1	FYVE and coiled-coil domain containing 1	0.004294494	0.9932577	0.4433076	0.79914202
1434153_at	SHB	SRC HOMOLOGY 2 DOMAIN-CONTAINING TRANSFORMING PROTEIN B	0.004302764	0.8560033	0.908638	1.48678369
1452161_at	TIPARP	TCDD-INDUCIBLE POLY(ADP-RIBOSE) POLYMERASE	0.004307401	0.006390009	0.1978319	1.206061258
1431496_at	B230104F01RIK	RIKEN CDNA B230104F01 GENE	0.004326662	0.1001636	0.08472249	0.507065888
1427728_at	CHRNG	CHOLINERGIC RECEPTOR, NICOTINIC, GAMMA POLYPEPTIDE	0.004348294	0.6161536	0.1192238	2.713758362
1453080_at	9130022K13RIK	RIKEN CDNA 9130022K13 GENE	0.004361579	0.9265856	0.6326769	0.260075283
1451871_a_at	GHR	GROWTH HORMONE RECEPTOR	0.004394831	0.01315646	0.8143499	1.56447954
1452624_at	Lrrtm1	leucine rich repeat transmembrane neuronal 1	0.004410201	1.75368E-09	0.09855046	0.758830796
1422088_at	Mycl1	v-myc myelocytomatosis viral oncogene homolog 1, lung carcinoma derived (avian)	0.00442022	0.02137067	0.1554219	0.644792514
1446237_at	Akap9	A kinase (PRKA) anchor protein (yotiao) 9	0.004436014	0.946461	0.3056546	1.762766905
1436769_at	PSMA1	PROTEASOME (PROSOME, MACROPAIN) SUBUNIT, ALPHA TYPE 1	0.004445471	0.797484	0.2820138	0.539337173
1427665_a_at	NFIC	NUCLEAR FACTOR I/C	0.004445666	0.6058722	0.4727632	1.931454325
1422221_at	HAND2	HEART AND NEURAL CREST DERIVATIVES EXPRESSED TRANSCRIPT 2	0.004471885	0.4005059	0.3870322	2.452813166
1454763_at	ANKRD17	ANKYRIN REPEAT DOMAIN 17	0.004488594	0.03332724	0.8540021	1.138316463
1455632_at	GNB5	GUANINE NUCLEOTIDE BINDING PROTEIN, BETA 5	0.004541135	0.529984	0.3452762	1.440083954
1418995_at	---	---	0.004550313	0.4243694	0.5891077	0.690217887
1444722_at	PSME4	PROTEASOME (PROSOME, MACROPAIN) ACTIVATOR SUBUNIT 4	0.004590158	0.2713532	0.06432952	0.793984018
1426383_at	CRY2	CRYPTOCHROME 2 (PHOTOLYASE-LIKE)	0.004596424	0.0747643	0.6846672	1.181276829
1434431_x_at	ADORA2B	ADENOSINE A2B RECEPTOR	0.004598378	0.2416206	0.2585774	0.719331205
1444169_at	3110052M02Rik	RIKEN cDNA 3110052M02 gene	0.00460571	0.2611484	0.6879995	1.464910881
1453777_a_at	NDST3	RIKEN CDNA 4921531K01 GENE	0.004636012	0.9797167	0.6289282	1.495119546
1446073_at	YAF2	YY1 ASSOCIATED FACTOR 2	0.004638485	0.000238377	0.05706189	0.520415832
1454529_at	4930518C04RIK	RIKEN CDNA 4930518C04 GENE	0.004674708	0.7702303	0.05682453	2.170610075
1449491_at	CARD10	CASPASE RECRUITMENT DOMAIN FAMILY, MEMBER 10	0.004750833	0.08910355	0.4880417	1.269636072
1428372_at	ST5	SUPPRESSION OF TUMORIGENICITY 5	0.00475275	0.2115799	0.3450327	0.732023875
1446255_at	KCNQ1	POTASSIUM VOLTAGE-GATED CHANNEL, SUBFAMILY Q, MEMBER 1	0.004767956	0.1743414	0.1700572	1.559110764
1424474_a_at	Camkk2	calcium/calmodulin-dependent protein kinase kinase 2, beta	0.004791302	0.2933521	0.4943917	1.502359596
1438213_at	A830018L16RIK	RIKEN CDNA A830018L16 GENE	0.004842968	0.3216865	0.9343686	0.776900923
1440506_at	SLC7A2	SOLUTE CARRIER FAMILY 7 (CATIONIC AMINO ACID TRANSPORTER, Y+ SYSTEM), MEMBER 2	0.004861527	0.9787995	0.6018137	0.63411475
1458351_s_at	KLHL2	KELCH-LIKE 2, MAYVEN (DROSOPHILA)	0.004877336	0.94748	0.4594371	0.67746938
1440325_at	SMARCA2	SWI/SNF RELATED, MATRIX ASSOCIATED, ACTIN DEPENDENT REGULATOR OF CHROMATIN, SUBFAMILY A, MEMBER 2	0.004879402	0.7602567	0.2990749	0.729427377
1430316_at	4930579E17RIK	RIKEN CDNA 4930579E17 GENE	0.004947402	0.7175509	0.814113	0.823574224
1436305_at	Ibrdc1	IBR domain containing 1	0.004960279	4.12078E-05	0.3744767	1.398654448
1456596_at	6430550H21RIK	EXPRESSED SEQUENCE AW260253	0.00498224	0.000524296	0.06545969	0.669864143
1431864_at	FBXO24	F-BOX ONLY PROTEIN 24	0.004985912	0.2058565	0.07152773	0.505066723
1439041_at	SLC39A10	SOLUTE CARRIER FAMILY 39 (ZINC TRANSPORTER), MEMBER 10	0.005006758	0.000351511	0.153062	1.172929189
1437082_at	Akap9	A kinase (PRKA) anchor protein (yotiao) 9	0.005023017	0.01305182	0.3498189	1.176781125
1439754_at	SOX12	SRY-BOX CONTAINING GENE 12	0.005028449	0.05663566	0.06245433	0.633399964
1418392_a_at	GBP4	GUANYLATE NUCLEOTIDE BINDING PROTEIN 4	0.005066811	0.7763975	0.1378248	0.674616662
1421530_a_at	GRM8	GLUTAMATE RECEPTOR, METABOTROPIC 8	0.005067501	0.7561452	0.3537733	1.423418519
1442341_at	KALRN	RIKEN CDNA 2210407G14 GENE	0.00506974	0.001132361	0.905379	1.463419901
1427060_at	MAPK3	PROTEIN KINASE, MITOGEN ACTIVATED KINASE 3	0.005080086	0.605224	0.4577927	0.788543447
1431014_at	CECR2	CAT EYE SYNDROME CHROMOSOME REGION, CANDIDATE 2 HOMOLOG (HUMAN)	0.005080106	0.3244494	0.4465205	0.692287123
1444683_at	---	---	0.00510448	0.3462227	0.0960744	0.621035327
1425248_a_at	Tyro3	TYRO3 protein tyrosine kinase 3	0.005107816	0.2896422	0.928668	1.398709667
1457212_at	---	Transcribed locus	0.005125981	0.2781517	0.7146505	1.480738825
1427738_at	D0KIST2	DNA SEGMENT, KIST 2	0.005151203	0.5414169	0.05959282	0.484459386
1416305_at	Sh3bp4	SH3-domain binding protein 4	0.005162017	0.730101	0.9062075	1.345125414
1448546_at	Rassf3	Ras association (RalGDS/AF-6) domain family 3	0.005164649	0.5527128	0.2042128	1.378105554
1421488_at	Rabgap1l	RAB GTPase activating protein 1-like	0.005180691	0.000202949	0.345198	1.379265487
1441024_at	STIM2	STROMAL INTERACTION MOLECULE 2	0.00518752	0.05844426	0.5717998	0.651763766
1450339_a_at	Bcl11b	B-cell leukemia/lymphoma 11B	0.005206022	0.000823295	0.9778594	0.645814017
1447952_at	AUH	AU RNA BINDING PROTEIN/ENOYL-COENZYME A HYDRATASE	0.005230539	0.4213596	0.09672197	1.327525266
1442119_at	AI449212	EXPRESSED SEQUENCE AI449212	0.005248949	0.6503496	0.369854	0.77864918
1458499_at	Pde10a	phosphodiesterase 10A	0.005252192	0.02624791	0.4188227	0.774616936
1440113_at	MTA1	METASTASIS ASSOCIATED 1	0.005258732	0.02058084	0.2159561	0.463634852
1442050_at	Zfp608 /// D430007A19Rik	zinc finger protein 608 /// RIKEN cDNA D430007A19 gene	0.005288092	0.1480431	0.1240171	1.207742962
1436582_at	ZDHHC15	ZINC FINGER, DHHC DOMAIN CONTAINING 15	0.005298344	0.004472658	0.1954462	0.703145143
1421834_at	Pip5k1a	phosphatidylinositol-4-phosphate 5-kinase, type 1 alpha	0.005312821	0.005251164	0.5577443	0.807114576
1440990_at	BC056349	CDNA SEQUENCE BC056349	0.005333686	0.1798648	0.4499934	0.787581526
1440613_at	PERLD1	PER1-LIKE DOMAIN CONTAINING 1	0.005383633	0.3760538	0.0996638	2.618653548
1454569_at	4930480K02RIK	RIKEN CDNA 4930480K02 GENE	0.00538704	0.0871007	0.07816479	2.96312803
1442447_at	---	---	0.005394734	0.4066016	0.06789738	0.685507183
1417810_a_at	PACSIN2	PROTEIN KINASE C AND CASEIN KINASE SUBSTRATE IN NEURONS 2	0.005399087	0.5606495	0.6732446	0.669034757
1441974_at	---	Transcribed locus	0.00540149	0.09392376	0.2136229	1.432823522
1418271_at	BHLHB5	BASIC HELIX-LOOP-HELIX DOMAIN CONTAINING, CLASS B5	0.005413308	0.1149079	0.4446327	0.661742066
1457267_at	IRF6	INTERFERON REGULATORY FACTOR 6	0.005413401	0.05606757	0.719621	0.833855532
1445810_at	C85163	EXPRESSED SEQUENCE C85163	0.005439075	0.3987945	0.1725436	2.021083379
1449428_at	CLDN18	CLAUDIN 18	0.005475912	0.08608799	0.1087346	2.966535036
1426338_a_at	Ntng1	netrin G1	0.005492046	0.9873319	0.383038	1.58917882
1443888_at	---	---	0.005551557	0.002886453	0.953701	0.757669494
1436012_s_at	SCRN2	SECERNIN 2	0.005577528	0.6719472	0.05899909	0.608852463
1455836_at	PAPOLA	POLY (A) POLYMERASE ALPHA	0.005588645	0.000962087	0.2032804	1.154577092
1450389_s_at	Pip5k1a	phosphatidylinositol-4-phosphate 5-kinase, type 1 alpha	0.005644645	0.3992816	0.4986139	0.726974657
1421992_a_at	Igfbp4	insulin-like growth factor binding protein 4	0.005676891	0.754703	0.9000421	1.408769989
1452424_at	Gpr23	G protein-coupled receptor 23	0.005692563	0.5228424	0.08044831	0.705907552
1448967_at	NIPSNAP3A	NIPSNAP HOMOLOG 3A (C. ELEGANS)	0.005702072	0.2794173	0.253576	0.735249746
1441531_at	PLCB4	PHOSPHOLIPASE C, BETA 4	0.005745859	0.9892666	0.4782513	1.573419803
1446309_at	5830483C08Rik	RIKEN cDNA 5830483C08 gene	0.005763772	0.6482055	0.104764	0.408168062
1451129_at	CALB2	CALBINDIN 2	0.005772721	0.9020158	0.06551436	1.317178667
1443216_at	TBC1D5	TBC1 DOMAIN FAMILY, MEMBER 5	0.005800485	0.033484689	0.08140123	0.642131082
1431651_at	C330001K17RIK	RIKEN CDNA C330001K17 GENE	0.005894304	0.8669784	0.3840967	1.77387771
1455683_a_at	TBC1D8	TBC1 DOMAIN FAMILY, MEMBER 8	0.005906004	0.9334195	0.1364929	1.48143117
1457391_at	VAMP3	VESICLE-ASSOCIATED MEMBRANE PROTEIN 3	0.005909972	0.7887013	0.9673007	3.53349433
1446942_at	---	---	0.005934768	0.4599237	0.4030151	2.587230604
1450449_a_at	2900002H16RIK	HYPOTHETICAL PROTEIN LOC58241	0.005961674	0.01814568	0.3274418	1.216403197
1456874_at	Flrt2	fibronectin leucine rich transmembrane protein 2	0.005995465	0.04846813	0.1378313	1.206435191
1434948_at	RAB11FIP2	RAB11 FAMILY INTERACTING PROTEIN 2 (CLASS I)	0.006015805	0.1114777	0.977923	0.782949968
1442378_x_at	---	---	0.006033929	0.1239146	0.063728049	0.647152519
1454073_at	4930455D15RIK	RIKEN CDNA 4930455D15 GENE	0.006034242	0.31639479	0.06032368	1.486615611
1427410_at	DLEU2	DELETED IN LYMPHOCYTIC LEUKEMIA, 2	0.006088959	0.2599524	0.8392806	1.578090549
1455819_at	Rod1	ROD1 regulator of differentiation 1 (S. pombe)	0.00612525	0.1503841	0.4043274	0.85278362
1437769_at	EPS15L1	EPIDERMAL GROWTH FACTOR RECEPTOR PATHWAY SUBSTRATE 15-LIKE 1	0.006134319	0.8764875	0.5685845	1.655050088
1433557_at	CBX7	CHROMOBOX HOMOLOG 7	0.00618016	0.1194259	0.5011978	1.238317731
1419843_at	CALR4	CALRETICULIN 4	0.006180285	0.53777889	0.58535269	0.350213406
1438036_x_at	AW061290	EXPRESSED SEQUENCE AW061290	0.00619493	0.09988832	0.9380958	0.703999295
1454543_at	STK40	SERINE/THREONINE KINASE 40	0.006197962	0.4654078	0.2630215	2.101856218
1425287_at	ZFP189	ZINC FINGER PROTEIN 189	0.006200977	0.1883892	0.197978	0.714164363
1420370_s_at	CSNB	CASEIN BETA	0.006203014	0.4805688	0.05064354	2.113687485
1460405_at	ARHGEF10L	RHO GUANINE NUCLEOTIDE EXCHANGE FACTOR (GEF) 10-LIKE	0.006247232	0.01203997	0.1049012	1.199648372
1456413_at	Pde4dip	phosphodiesterase 4D interacting protein (myomegalin)	0.006247459	0.00113795	0.09872538	1.161166357
1456238_at	ZFP133	ZINC FINGER PROTEIN 133	0.006298187	0.1067581	0.588173	0.716046645
1421879_at	MTMR1	MYOTUBULARIN RELATED PROTEIN 1	0.006309978	0.02415872	0.7409228	1.232748025
1439666_at	2010001J22RIK	RIKEN CDNA 2010001J22 GENE	0.006364122	0.8143963	0.1222434	1.503740881
1426031_a_at	NFATC2	NUCLEAR FACTOR OF ACTIVATED T-CELLS, CYTOPLASMIC, CALCINEURIN-DEPENDENT 2	0.006403951	0.8909397	0.1690614	1.670018306
1456318_at	CLEC1A	C-TYPE LECTIN DOMAIN FAMILY 1, MEMBER A	0.006429956	0.03694542	0.4757851	0.710052441
1453061_at	2610018O07RIK	RIKEN CDNA 2610018O07 GENE	0.006434466	0.1600988	0.1182197	0.776423219
1421541_a_at	MEF2B	MYOCYTE ENHANCER FACTOR 2B	0.006446689	0.02843886	0.1121666	1.685759166
1457384_at	---	---	0.006454179	0.6642265	0.8681412	0.709694159
1426476_at	Rasa1	RAS p21 protein activator 1	0.006455866	0.5396331	0.8071634	0.848433189
1450178_at	BRDT	BROMODOMAIN, TESTIS-SPECIFIC	0.006462225	0.05659381	0.3539546	1.3153966
1446221_at	ELN	ELASTIN	0.006493818	0.7462552	0.0944377	0.623007376
1446992_at	CCDC64	RIKEN CDNA 2210403N09 GENE	0.006527701	0.9479097	0.14376249	0.696782301
1416845_at	TMEM132A	HEAT SHOCK PROTEIN 5 BINDING PROTEIN 1	0.006530588	0.7039305	0.8016753	0.81195613
1424755_at	HIP1	RIKEN CDNA A930014B11 GENE	0.00653641	0.0866074	0.1222887	1.213795542
1460426_at	Pde4dip	phosphodiesterase 4D interacting protein (myomegalin)	0.006536913	0.88981	0.7837294	1.481968968
1459231_at	PKN1	PROTEIN KINASE C-LIKE 1	0.006553337	0.6348123	0.4727532	2.35377746
1455473_at	USP13	UBIQUITIN SPECIFIC PEPTIDASE 13 (ISOPEPTIDASE T-3)	0.006559064	0.09681143	0.7474675	0.773862448
1432281_a_at	ITGB6	INTEGRIN BETA 6	0.006560875	0.940293	0.4878317	0.648005311
1417040_a_at	BOK	BCL-2-RELATED OVARIAN KILLER PROTEIN	0.006565691	0.3999938	0.7870469	1.362316316
1460482_at	3110047P20RIK	RIKEN CDNA 3110047P20 GENE	0.006620833	0.6391206	0.1813364	1.261271076
1422839_at	NEUROG2	NEUROGENIN 2	0.00662163	0.6703076	0.573901	0.581560675
1435706_at	BTBD9	RIKEN CDNA 1700023F20 GENE	0.006626521	0.01702748	0.7707038	0.696415344
1428835_at	MYH14	MYOSIN, HEAVY POLYPEPTIDE 14	0.006633732	0.317955	0.05879245	1.194625621
1420720_at	NPTX2	NEURONAL PENTRAXIN 2	0.006633798	0.3836937	0.1961892	2.517797525
1421897_at	Elk1	ELK1, member of ETS oncogene family	0.00663929	0.569003	0.9482413	0.690248132
1421419_at	KCNK4	POTASSIUM CHANNEL, SUBFAMILY K, MEMBER 4	0.006671329	0.006007141	0.2188315	1.608722418
1444681_at	D14ERTD171E	DNA SEGMENT, CHR 14, ERATO DOI 171, EXPRESSED	0.006675725	0.01436424	0.83063	0.627057575
1448458_at	Top2b	topoisomerase (DNA) II beta	0.006678652	0.000120064	0.5197621	1.287286155
1434917_at	COBL	CORDON-BLEU	0.006701032	0.003371751	0.874117	1.382349131
1435648_at	B430119L13RIK	RIKEN CDNA B430119L13 GENE	0.006704013	0.1089492	0.0999479	0.650159226
1435367_at	MAPK4	MITOGEN-ACTIVATED PROTEIN KINASE 4	0.00670929	0.2375368	0.4714059	1.363969422
1418578_at	DGKA	DIACYLGLYCEROL KINASE, ALPHA	0.006738155	0.1502769	0.8443929	0.796766595
1437748_at	FUT11	FUCOSYLTRANSFERASE 11	0.006775241	6.87584E-05	0.06191382	1.102002302
1424481_s_at	LOC494468	EXPRESSED SEQUENCE AW556585	0.006790267	0.002412964	0.1791107	0.779537531
1424656_s_at	USP19	UBIQUITIN SPECIFIC PEPTIDASE 19	0.006796683	0.04895125	0.08199506	1.118713442
1449171_at	TTK	TTK PROTEIN KINASE	0.00682779	0.3663829	0.05813473	0.571954364
1436120_at	SETDB2	SET DOMAIN, BIFURCATED 2	0.006846319	0.1604923	0.362445	0.824457174
1453469_at	1810073G21RIK	RIKEN CDNA 1810073G21 GENE	0.006847843	0.4171884	0.8627283	0.388264997
1428046_a_at	ZFX	ZINC FINGER PROTEIN X-LINKED	0.006856586	0.001523559	0.2327696	1.334878076
1457708_at	MBD4	METHYL-CPG BINDING DOMAIN PROTEIN 4	0.006865967	0.5845806	0.3390681	0.804706992
1445069_at	---	---	0.006885771	0.1246035	0.1410154	3.986680418
1439618_at	Pde10a	phosphodiesterase 10A	0.006900433	0.08390849	0.1454756	0.64846185

1449422_at	Cdh4	cadherin 4	0.006904786	0.61547909	0.9027287	1.469933291
1434458_at	FST	FOLLISTATIN	0.006909815	0.9751701	0.7673402	0.451632118
1446209_at	USP40	UBIQUITIN SPECIFIC PEPTIDASE 40	0.006914489	0.3928273	0.0925498	0.823193471
1432219_at	NOL9	NUCLEOLAR PROTEIN 9	0.006914772	0.08527243	0.7154579	0.563052594
1420288_at	T25656	expressed sequence T25656	0.006914917	0.08721308	0.1139768	2.680393004
1434049_at	ENTPD3	ECTONUCLEOSIDE TRIPHOSPHATE DIPHOSPHOHYDROLASE 3	0.00691792	0.9380549	0.3530669	0.746652943
1458205_at	---	---	0.006919879	0.2606164	0.8301647	1.520639228
1425201_a_at	HYI	HYDROXYPYRUVATE ISOMERASE HOMOLOG (E. COLI)	0.006922727	0.2413584	0.1794593	0.829101716
1455025_at	PAQR9	PROGESTIN AND ADIPOQ RECEPTOR FAMILY MEMBER IX	0.006936826	0.2664281	0.7495527	0.863251628
1456773_at	NUPL2	NUCLEOPORIN LIKE 2	0.00694744	0.1668417	0.4652361	0.76274132
1435685_x_at	ABCC5	ATP-BINDING CASSETTE, SUB-FAMILY C (CFTR/MRP), MEMBER 5	0.006968604	0.002112138	0.3209479	0.720983679
1436447_at	A630026N12RIK	RIKEN CDNA A630026N12 GENE	0.007007768	0.01298234	0.6849206	0.802476178
1458114_at	SAMD12	STERILE ALPHA MOTIF DOMAIN CONTAINING 12	0.00705429	0.1935725	0.7920593	1.38458455
1457409_at	FUT9	FUCOSYLTRANSFERASE 9	0.007062036	0.0477162	0.5457826	1.218955435
1428202_at	1810037C20RIK	RIKEN CDNA 1810037C20 GENE	0.007063043	0.004661752	0.1580317	0.843760706
1417933_at	IGFBP6	INSULIN-LIKE GROWTH FACTOR BINDING PROTEIN 6	0.007094081	0.5560857	0.4583711	1.328700654
1440250_at	COL4A4	PROCOLLAGEN, TYPE IV, ALPHA 4	0.007133571	0.1134181	0.1316891	2.014964511
1431680_a_at	Ptprk	protein tyrosine phosphatase, receptor type, K	0.007174783	3.28235E-06	0.3771993	1.179492048
1449980_a_at	GABRD	GAMMA-AMINOBUTYRIC ACID (GABA-A) RECEPTOR, SUBUNIT DELTA	0.007181606	0.04107823	0.2641572	1.337365112
1440587_at	---	---	0.007188332	0.000158732	0.1040437	0.679796067
1420976_at	Man1a2	mannosidase, alpha, class 1A, member 2	0.00719338	0.01204148	0.2733725	1.251464749
1423630_at	CYGB	STELLATE CELL ACTIVATION ASSOCIATED PROTEIN	0.00721657	0.0033631	0.7147548	0.622737938
1453946_a_at	SDCCAG8	RIKEN CDNA 5730470G24 GENE	0.00723435	0.01759772	0.7568963	0.742595123
1445579_at	PPP1R1A	PROTEIN PHOSPHATASE 1, REGULATORY (INHIBITOR) SUBUNIT 1A	0.007242107	2.61902E-05	0.424258	1.211652796
1453533_at	4933403O08RIK	RIKEN CDNA 4933403O08 GENE	0.007340388	0.1903035	0.189002	3.36558853
1424007_at	GDF10	GROWTH DIFFERENTIATION FACTOR 10	0.007344236	0.001967042	0.8610148	0.693891361
1450440_at	Gfra1	glial cell line derived neurotrophic factor family receptor alpha 1	0.007375321	0.7714802	0.6311808	0.727299352
1453682_at	GABPB2	GA REPEAT BINDING PROTEIN, BETA 2	0.007378877	0.1830775	0.8479208	0.641622534
1436652_at	5830418K08RIK	RIKEN CDNA 5830418K08 GENE	0.007409393	0.49875	0.1172127	0.767795994
1439204_at	SCN3A	SODIUM CHANNEL, VOLTAGE-GATED, TYPE III, ALPHA	0.007409486	0.6160113	0.7801989	0.728698276
1439723_at	5730590G19RIK	RIKEN CDNA 5730590G19 GENE	0.007429916	0.760052	0.1057512	0.621545263
1435424_x_at	---	---	0.007457173	0.2093484	0.105414	0.702077058
1422353_at	POU4F3	POU DOMAIN, CLASS 4, TRANSCRIPTION FACTOR 3	0.007478505	6.76732E-05	0.2637273	1.698626112
1429537_at	5730406M06RIK	RIKEN CDNA 5730406M06 GENE	0.007528175	0.2477173	0.4570599	1.25412784
1445394_at	---	---	0.007533778	0.4585504	0.5272905	1.586158867
1424605_at	Pcsk5	proprotein convertase subtilisin/kexin type 5	0.007578938	0.1369809	0.130558	0.711510813
1458656_at	AW050198	EXPRESSED SEQUENCE AW050198	0.007584741	0.009045965	0.9033059	0.751264186
1449206_at	SYPL2	SYNAPTOPHYSIN-LIKE 2	0.007585852	0.1976434	0.13624919	1.451612377
1437781_at	---	---	0.007592198	0.7731823	0.1444279	0.506885261
1422836_at	MBNL3	MUSCLEBLIND-LIKE 3 (DROSOPHILA)	0.00759724	0.03532181	0.8822745	2.857953673
1439616_at	---	---	0.007602574	0.3885251	0.5274222	0.54926151
1422208_a_at	Gnb5	guanine nucleotide binding protein, beta 5	0.007602874	0.08941653	0.30407859	1.237665708
1438531_at	A730054J21RIK	RIKEN CDNA A730054J21 GENE	0.007628752	0.2839031	0.6203504	0.795213481
1443618_at	PDZD2	PDZ DOMAIN CONTAINING 2	0.007631083	0.2040155	0.4128052	2.767286898
1459679_s_at	Myo1b	myosin IB	0.007694639	0.1777786	0.2904401	1.515176639
1425600_a_at	PLCB1	PHOSPHOLIPASE C, BETA 1	0.007723938	0.0001402	0.433215	1.152541858
1435608_at	Znrf3 /// LOC631806	zinc and ring finger 3 /// similar to Goliath homolog precursor (Ring finger protein 130) (R-goliath)	0.007733432	0.16775309	0.5193117	0.781454923
1417149_at	P4HA2	PROCOLLAGEN-PROLINE, 2-OXOGLUTARATE 4-DIOXYGENASE (PROLINE 4-HYDROXYLASE), ALPHA II POLYPEPTIDE	0.007784709	0.2899968	0.1140307	1.263245706
1437763_at	DCUN1D3	DCN1, DEFECTIVE IN CULLIN NEDDYLATION 1, DOMAIN CONTAINING 3 (S. CEREVISIAE)	0.007788948	0.55029019	0.569411	1.227978146
1425051_at	Isoc1	isochorismatase domain containing 1	0.00780368	0.02092873	0.6574329	0.789022812
1427149_at	PLEKHA6	HYPOTHETICAL PROTEIN BC031133	0.007821571	0.06454198	0.2795887	1.335745503
1456648_at	Man1a	Mannosidase 1, alpha	0.007948988	0.4379848	0.7039622	1.234384744
1434186_at	Gpr23	G protein-coupled receptor 23	0.007953949	0.8614117	0.3899946	0.618602875
1438451_at	Grit	Rho GTPase-activating protein	0.007981646	0.0005411	0.1540918	1.14235706
1436439_at	1700016G14RIK	RIKEN CDNA 1700016G14 GENE	0.00800239	0.8247489	0.1745239	0.405676948
1448327_at	ACTN2	ACTININ ALPHA 2	0.00800563	0.5380552	0.9672326	0.821425542
1417967_at	MMS19L	MMS19 (MET18 S. CEREVISIAE)-LIKE	0.008037683	0.1161982	0.5500307	0.818569566
1439665_at	Gpr23	G protein-coupled receptor 23	0.008041693	0.1482931	0.552721	0.698340387
1418061_at	LTBP2	LATENT TRANSFORMING GROWTH FACTOR BETA BINDING PROTEIN 2	0.008046605	0.004286238	0.3412247	1.954993485
1434313_at	6330407D12RIK	RIKEN CDNA 6330407D12 GENE	0.008055855	0.7796855	0.4095038	0.609656447
1433169_at	5830456J23RIK	RIKEN CDNA 5830456J23 GENE	0.008079102	0.00065756	0.1927962	0.740994277
1439668_at	---	---	0.008087926	0.02856067	0.787312	0.745201131
1448914_a_at	Csf1	colony stimulating factor 1 (macrophage)	0.00813638	0.09580167	0.3253456	0.791371963
1455074_at	EFCAB1	EF HAND CALCIUM BINDING DOMAIN 1	0.008150894	0.4957243	0.2815038	0.767895269
1456591_x_at	AKAP3	A KINASE (PRKA) ANCHOR PROTEIN 3	0.008152225	0.4699934	0.2375414	1.547850375
1423235_at	AP3B1	ADAPTOR-RELATED PROTEIN COMPLEX 3, BETA 1 SUBUNIT	0.008155243	0.05319994	0.8078833	0.819665204
1434760_at	Lrrtm3	leucine rich repeat transmembrane neuronal 3	0.008195586	0.7670182	0.4415741	1.388039491
1429813_at	PANK1	PANTOTHENATE KINASE 1	0.008252602	0.3998037	0.08734593	0.466379755
1435607_at	GRM2	G PROTEIN-COUPLED RECEPTOR, FAMILY C, GROUP 1, MEMBER B	0.008252655	0.01893581	0.8601461	0.735642886
1451406_a_at	Pcsk5	proprotein convertase subtilisin/kexin type 5	0.008265029	0.8070914	0.3928511	0.619071576
1454011_a_at	RPA2	REPLICATION PROTEIN A2	0.00826901	0.000462988	0.1695503	0.777197037
1446353_at	TUBB6	TUBULIN, BETA 6	0.008317564	0.02429681	0.5593976	0.761739581
1445427_at	NLK	NEMO LIKE KINASE	0.008332603	0.04760269	0.85544	1.618718777
1448744_at	GALNS	GALACTOSAMINE (N-ACETYL)-6-SULFATE SULFATASE	0.008338795	0.28498	0.923629	0.785958618
1424963_at	RP1H	RETINITIS PIGMENTOSA 1 HOMOLOG (HUMAN)	0.008402646	0.2146266	0.6156245	0.397553628
1441142_at	2700081L22RIK	RIKEN CDNA 2700081L22 GENE	0.008417595	0.3457895	0.7701847	0.697525858
1458742_at	LPHN3	LATROPHILIN 3	0.008425256	0.5887265	0.7478604	1.239557021
1448797_at	ELK3	ELK3, MEMBER OF ETS ONCOGENE FAMILY	0.0084279	0.08184075	0.304296	0.751030408
1451627_a_at	SLC1A2	SOLUTE CARRIER FAMILY 1 (GLIAL HIGH AFFINITY GLUTAMATE TRANSPORTER), MEMBER 2	0.008440962	0.1352844	0.2401468	1.175999799
1450055_at	VSNL1	VISININ-LIKE 1	0.008445006	0.003935547	0.2816967	1.113393914
1448901_at	CPXM1	CARBOXYPEPTIDASE X 1 (M14 FAMILY)	0.008448442	0.001002078	0.07846673	1.126847133
1426608_at	---	---	0.008448893	0.75179	0.1024405	1.560578069
1458438_at	4933415L06RIK	RIKEN CDNA 4933415L06 GENE	0.008477647	0.4777715	0.4893274	0.806998123
1435892_at	Asxl3	additional sex combs like 3 (Drosophila)	0.00848125	0.01028027	0.06249341	1.154176585
1453713_s_at	4930546H06RIK	RIKEN CDNA 4930546H06 GENE	0.008511452	0.04018539	0.1510929	1.248971939
1436299_at	Gls	glutaminase	0.008530788	0.1531925	0.46156	1.203092076
1438476_a_at	CHD4	RIKEN CDNA 9530019N15 GENE	0.008543234	0.04898403	0.2639127	1.330447895
1456871_a_at	PHF20L1	PHD FINGER PROTEIN 20-LIKE 1	0.008546081	0.000137564	0.08451474	1.096581174
1431337_a_at	1810055E12RIK	RIKEN CDNA 1810055E12 GENE	0.0085595	0.04790533	0.5805072	0.859953713
1440060_at	LOC331480	HYPOTHETICAL LOC331480	0.008569497	0.2134927	0.973938	1.332830246
1439697_at	Il1rap	interleukin 1 receptor accessory protein	0.00858601	0.000914225	0.986754	0.716056569
1436234_at	4732471D19Rik	RIKEN cDNA 4732471D19 gene	0.008617343	0.8903546	0.7998522	0.783588777
1439341_at	AK220484	CDNA SEQUENCE AK220484	0.0086811	0.892259	0.7224162	0.737644863
1440032_at	E130008O17RIK	RIKEN CDNA E130008O17 GENE	0.008688106	0.03023362	0.2432511	1.315568416
1460220_a_at	Csf1	colony stimulating factor 1 (macrophage)	0.008694793	0.5937561	0.7611148	0.773271122
1453710_at	TMEM116	RIKEN CDNA 4930513P12 GENE	0.008701855	0.58273109	0.05696789	0.629401776
1426560_a_at	Npnt	nephronectin	0.008715761	0.7615768	0.4793632	0.61533869
1431871_at	TXNDC3	THIOREDOXIN DOMAIN CONTAINING 3 (SPERMATOZOA)	0.00871976	0.0634027	0.6293458	0.425305233
1417626_at	Pde4dip	phosphodiesterase 4D interacting protein (myomegalin)	0.008722797	0.4112889	0.9939586	1.296798628
1434761_at	Lrrtm3	leucine rich repeat transmembrane neuronal 3	0.008775104	0.8152745	0.7440047	1.403310336
1429183_at	Pkp2	plakophilin 2	0.00878133	4.2997E-05	0.8383278	0.742472857
1438006_at	4933439F18Rik	RIKEN cDNA 4933439F18 gene	0.008836289	0.002483826	0.9568293	0.82087604
1434889_at	PLEKHA7	PLECKSTRIN HOMOLOGY DOMAIN CONTAINING, FAMILY A MEMBER 7	0.0088424	0.7051713	0.9111043	1.48736009
1432746_at	6030442H21RIK	RIKEN CDNA 6030442H21 GENE	0.008900586	0.5091595	0.1654265	0.715944819
1425272_at	EMP2	EPITHELIAL MEMBRANE PROTEIN 2	0.008910297	0.0250319	0.2199481	1.269157592
1437858_at	Dpy19l3	dpy-19-like 3 (C. elegans)	0.008948078	0.3246398	0.865934	1.250931902
1453510_s_at	4930589M24RIK	RIKEN CDNA 4930438C08 GENE	0.00897021	0.03881051	0.7184901	0.808005145
1425581_s_at	Galnt7	UDP-N-acetyl-alpha-D-galactosamine: polypeptide N-acetylgalactosaminyltransferase 7	0.008973631	3.05012E-06	0.1189477	1.073558905
1422255_at	KCNA4	POTASSIUM VOLTAGE-GATED CHANNEL, SHAKER-RELATED SUBFAMILY, MEMBER 4	0.008987567	0.0796182	0.3370619	1.271013479
1437256_at	DCUN1D5	RIKEN CDNA 3110001A18 GENE	0.009086153	0.01166125	0.6345039	0.804877017
1442708_at	---	---	0.009137782	0.04364124	0.109173	1.295297376
1436184_at	GM632	GENE MODEL 632, (NCBI)	0.009222963	0.02383799	0.4842044	0.808441062
1436177_at	---	Transcribed locus	0.009233465	0.005600903	0.8594136	0.816784571
1457152_at	KCNB2	POTASSIUM VOLTAGE GATED CHANNEL, SHAB-RELATED SUBFAMILY, MEMBER 2	0.009263847	0.00014839	0.6174124	1.369722582
1417800_at	PARP2	POLY (ADP-RIBOSE) POLYMERASE FAMILY, MEMBER 2	0.009266755	0.1255764	0.8377258	0.797254013
1436714_at	LPP	LIM DOMAIN CONTAINING PREFERRED TRANSLOCATION PARTNER IN LIPOMA	0.009301955	0.008153835	0.1574878	0.82326572
1433325_at	9330154F10RIK	RIKEN CDNA 9330154F10 GENE	0.009448544	0.32383659	0.9484249	1.362217734
1448364_at	CCNG2	CYCLIN G2	0.009472471	0.0002716	0.458703	1.11455191
1436734_at	E130309F12Rik	RIKEN cDNA E130309F12 gene	0.009537468	0.3816194	0.09857642	1.26297534
1425671_at	HOMER1	HOMER HOMOLOG 1 (DROSOPHILA)	0.009549801	0.6947892	0.8092256	1.604464451
1428827_at	WHSC1	RIKEN CDNA 5830445G22 GENE	0.009557657	0.6071952	0.1735167	1.588020146
1434382_at	SERINC2	SERINE INCORPORATOR 2	0.009562432	0.621404	0.2012061	1.786370495
1450082_s_at	ETV5	RIKEN CDNA 8430401F14 GENE	0.009608253	0.08145517	0.9427153	1.319819726
1458425_at	---	---	0.009612545	0.6012514	0.1307615	1.275934571
1442411_at	Glcci1	Glucocorticoid induced transcript 1	0.009619775	0.00145032	0.5708142	1.479753211
1450223_at	Apaf1	apoptotic peptidase activating factor 1	0.009657353	0.5150641	0.2577881	0.659217866
1426818_at	SOAT1	STEROL O-ACYLTRANSFERASE 1	0.009671469	0.1508692	0.3595136	0.705052745
1459473_at	PDSS2	PRENYL (SOLANESYL) DIPHOSPHATE SYNTHASE, SUBUNIT 2	0.009692705	0.22525	0.9347688	1.584854063
1424092_at	Epb4.1	erythrocyte protein band 4.1	0.009717495	0.01980925	0.3755125	1.502483457
1437295_at	PKN2	PROTEIN KINASE N2	0.0097292	0.000131226	0.1229767	1.043072067
1417420_at	Ccnd1	cyclin D1	0.009749943	0.04141898	0.8819108	1.471402134
1430466_at	2310005G13RIK	RIKEN CDNA 2310005G13 GENE	0.009768778	0.05372555	0.1798771	0.308043226
1431268_at	1700047I17RIK	RIKEN CDNA 1700047I17 GENE	0.009796566	0.5016856	0.1380569	0.447607136
1460370_at	TOP1MT	DNA TOPOISOMERASE 1, MITOCHONDRIAL	0.009804646	0.4501406	0.7103154	0.781016185
1435403_at	---	---	0.009824078	0.2306461	0.8193454	0.66304475
1437594_x_at	PIGT	PHOSPHATIDYLINOSITOL GLYCAN, CLASS T	0.009826351	0.01530011	0.2056835	0.759266548
1449696_at	---	---	0.009839086	0.27693269	0.5724888	2.476958741
1438642_at	GALK2	GALACTOKINASE 2	0.009843265	0.8052437	0.812992	0.469386329
1436854_at	TRPC2	TRANSIENT RECEPTOR POTENTIAL CATION CHANNEL, SUBFAMILY C, MEMBER 2	0.009843436	0.1051564	0.3319697	0.752047329
1417545_at	TRPV4	TRANSIENT RECEPTOR POTENTIAL CATION CHANNEL, SUBFAMILY V, MEMBER 4	0.009894833	0.08493752	0.07576431	2.160361704
1418726_a_at	Tnnt2	troponin T2, cardiac	0.009969775	0.1576999	0.3051302	0.66412549
1438752_at	A230058F20RIK	RIKEN CDNA A230058F20 GENE	0.009999514	0.002761246	0.4528851	1.222650809
1416892_s_at	3110001A13RIK	RIKEN CDNA 3110001A13 GENE	0.0100279	0.3757953	0.4452408	0.634305357
1418592_at	Dnaja4	DnaJ (Hsp40) homolog, subfamily A, member 4	0.01003502	0.08396349	0.5471975	1.18684281
1418393_a_at	ITGA7	INTEGRIN ALPHA 7	0.01003841	0.004625604	0.6549511	0.743597323
1450724_at	DRCTNNB1A	DOWN-REGULATED BY CTNNB1, A	0.01004876	0.1882426	0.434371	0.905776354
1442013_at	4930532D21RIK	RIKEN CDNA 4930532D21 GENE	0.01006808	0.9735152	0.3858247	2.900105204
1436901_at	SYMPK	SYMPLEKIN	0.0100727	0.7428741	0.996976	0.473251537
1445549_at	---	---	0.01011536	0.09220197	0.7403225	0.723663218
1417630_at	MKNK1	MAP KINASE-INTERACTING SERINE/THREONINE KINASE 1	0.01011621	0.008745507	0.9956558	0.828608024
1452525_a_at	NF1	NEUROFIBROMATOSIS 1	0.0101588	0.5516039	0.4610039	2.024210263
1455325_at	A230057G18RIK	RIKEN CDNA A230057G18 GENE	0.01016009	0.06861428	0.680286	0.794724316
1455091_at	MSL2L1	MALE-SPECIFIC LETHAL 2-LIKE 1 (DROSOPHILA)	0.01022022	0.8982234	0.7715394	0.759295325
1427778_at	DEFB8	DEFENSIN BETA 8	0.01023796	0.13534579	0.07740533	0.290116684
1448745_s_at	Lor	loricrin	0.01027683	0.005380684	0.2377546	0.624704114
1456145_at	---	---	0.01028051	0.7026396	0.1018867	1.389782243
1422527_at	H2-DMA	HISTOCOMPATIBILITY 2, CLASS II, LOCUS DMA	0.01029382	0.1653095	0.6932896	0.825396934
1441111_at	RP23-427P14.1	SIMILAR TO MYOSIN LIGHT CHAIN KINASE	0.01033819	0.04592156	0.64487609	2.186056597
1427894_at	SLITL2	RIKEN CDNA 2610528G05 GENE	0.01034532	0.08274869	0.0908002	1.194711097

1418639_at	SFTPC	SURFACTANT ASSOCIATED PROTEIN C	0.01034834	0.1213087	0.1159914	0.839444169
1449325_at	FADS2	FATTY ACID DESATURASE 2	0.01035288	0.000532414	0.2786457	1.11449589
1459971_at	---	---	0.01037531	0.2275356	0.8498135	1.997119516
1441055_at	R75368	EXPRESSED SEQUENCE R75368	0.0104231	0.02858314	0.4702256	0.74793488
1455700_at	MTERFD3	MTERF DOMAIN CONTAINING 3	0.01045027	0.9514537	0.522018899	0.738194507
1437095_at	Tspan18	tetraspanin 18	0.0104558	0.9702629	0.9467906	0.309002894
1438275_at	C130046K22RIK	RIKEN CDNA C130046K22 GENE	0.01047562	0.381841	0.1020234	1.29829073
1422816_a_at	MUTYH	MUTY HOMOLOG (E. COLI)	0.01048202	0.2654607	0.4683883	1.447875168
1441251_a_at	2010001A14RIK	RIKEN CDNA 2010001A14 GENE	0.0104853	0.1778707	0.1244071	1.386936689
1439524_at	DNAJB4	DNAJ (HSP40) HOMOLOG, SUBFAMILY B, MEMBER 4	0.0104892	0.2976703	0.2679724	0.831702714
1450522_a_at	H1F0	H1 HISTONE FAMILY, MEMBER 0	0.01052352	0.000339044	0.2237754	0.789377461
1451498_at	BC004853	CDNA SEQUENCE BC004853	0.01059133	0.9505187	0.8453592	0.62862318
1458633_at	SNX8	SORTING NEXIN 8	0.01062554	0.275177	0.676396	1.565806203
1458264_at	4930412F12Rik	RIKEN cDNA 4930412F12 gene	0.01063373	0.5134213	0.1083398	0.448900045
1419794_at	2410089E03RIK	RIKEN CDNA 2410089E03 GENE	0.01065428	0.8467572	0.1899643	0.550066489
1457188_at	ARHGEF11	RHO GUANINE NUCLEOTIDE EXCHANGE FACTOR (GEF) 11	0.01066932	0.1293303	0.1200275	0.807720383
1456114_at	CDS1	CDP-DIACYLGLYCEROL SYNTHASE 1	0.0107175	0.1247904	0.3018718	1.122899647
1457000_at	BC060267	CDNA SEQUENCE BC060267	0.01076125	0.985349	0.52636	1.714781359
1433466_at	AI467606	EXPRESSED SEQUENCE AI467606	0.01078685	0.5182186	0.3329902	3.635199275
1416403_at	Abcb10	ATP-binding cassette, sub-family B (MDR/TAP), member 10	0.01079979	0.007597413	0.7095446	0.716820413
1435065_x_at	VAV2	VAV2 ONCOGENE	0.01089294	0.391762	0.2166189	0.719952175
1425709_at	Rnf17	ring finger protein 17	0.01091426	0.3649866	0.3271822	1.898165565
1445055_at	---	---	0.01092756	0.4197695	0.2036165	0.365559723
1452278_a_at	HACE1	HECT DOMAIN AND ANKYRIN REPEAT CONTAINING, E3 UBIQUITIN PROTEIN LIGASE 1	0.01093284	0.09756087	0.4807167	0.739698669
1418663_at	MPDZ	MULTIPLE PDZ DOMAIN PROTEIN	0.01096294	0.1786143	0.1545866	1.241194177
1436587_at	1700123D08RIK	RIKEN CDNA 1700123D08 GENE	0.01097554	0.81347	0.06708753	1.309446332
1444937_at	---	---	0.01101097	0.4021398	0.2687431	2.150813697
1423786_at	8430410A17RIK	RIKEN CDNA 8430410A17 GENE	0.011013	0.2532223	0.09565337	1.109760876
1421520_at	Jph1	junctophilin 1	0.01102413	0.001021108	0.5532901	0.667897173
1434353_at	SFMBT2	RIKEN CDNA D330030P06 GENE	0.01105366	0.04493453	0.4364445	0.702032291
1433702_at	D19WSU12E	DNA SEGMENT, CHR 19, WAYNE STATE UNIVERSITY 12, EXPRESSED	0.01107295	0.0824922	0.55112609	0.757777072
1453016_at	2900042B11RIK	RIKEN CDNA 2900042B11 GENE	0.01108589	0.3404909	0.783899	0.81629697
1450653_at	SPZ1	SPERMATOGENIC ZIP 1	0.01108849	0.2792377	0.175234	2.504974012
1456960_at	ADK	ADENOSINE KINASE	0.01108892	0.009461193	0.3832622	0.649864093
1459336_at	GUCY1A2	GUANYLATE CYCLASE 1, SOLUBLE, ALPHA 2	0.01109812	0.4375056	0.3104261	1.886058041
1418753_at	GFPT2	GLUTAMINE FRUCTOSE-6-PHOSPHATE TRANSAMINASE 2	0.01109897	0.5444221	0.2881065	1.212313251
1460427_a_at	ADAM28	A DISINTEGRIN AND METALLOPEPTIDASE DOMAIN 28	0.01110328	0.441273	0.09170274	0.495063674
1458976_at	C230035I16RIK	RIKEN CDNA C230035I16 GENE	0.0111129	0.799568	0.8014815	0.612911946
1437267_x_at	SETD1B	SET DOMAIN CONTAINING 1B	0.0111147	4.74876E-05	0.5372679	0.820040009
1417392_a_at	SLC7A7	SOLUTE CARRIER FAMILY 7 (CATIONIC AMINO ACID TRANSPORTER, Y+ SYSTEM), MEMBER 7	0.0111827	0.3435809	0.3089906	1.435359313
1416975_at	Stam2	signal transducing adaptor molecule (SH3 domain and ITAM motif) 2	0.01123585	0.001690013	0.2302767	1.216127905
1427647_at	BC022150	CDNA SEQUENCE BC022150	0.01124059	0.00988045	0.1694537	1.914104245
1427198_at	BC022960	CDNA SEQUENCE BC022960	0.0112965	0.1070323	0.7731576	0.653341721
1418237_s_at	COL18A1	PROCOLLAGEN, TYPE XVIII, ALPHA 1	0.01132114	0.2761782	0.8002319	2.487343723
1434925_at	4833424O15Rik	RIKEN cDNA 4833424O15 gene	0.01132419	0.04245032	0.1050575	1.18618341
1430864_at	TTLL9	TUBULIN TYROSINE LIGASE LIKE FAMILY 9	0.01135542	0.1032119	0.0751026	1.651366475
1460448_s_at	Ttc14	tetratricopeptide repeat domain 14	0.01141797	0.001267684	0.4690196	0.806307804
1436407_at	9430069J07RIK	RIKEN CDNA 9430069J07 GENE	0.01146449	0.8524252	0.3099615	0.752269137
1422293_a_at	KCTD1	POTASSIUM CHANNEL TETRAMERISATION DOMAIN CONTAINING 1	0.01146774	0.007589546	0.2739485	1.199370216
1435808_at	A230051G13RIK	RIKEN CDNA A230051G13 GENE	0.01156306	0.6444522	0.2149691	0.870854235
1442813_at	DGKI	DIACYLGLYCEROL KINASE, IOTA	0.01163317	0.01063697	0.2820211	1.233723331
1430844_at	1700018M17RIK	RIKEN CDNA 1700018M17 GENE	0.01164369	0.02104835	0.1533291	1.676882478
1418141_at	DCX	DOUBLECORTIN	0.01166534	0.6904953	0.266642	1.202735821
1430088_at	3000002G13RIK	RIKEN CDNA 3000002G13 GENE	0.01171093	0.5796435	0.8346046	0.831715499
1445695_at	ATXN1	ATAXIN 1	0.01171942	0.4090494	0.1329378	1.414313204
1453755_at	Lsm11	U7 snRNP-specific Sm-like protein LSM11	0.01172717	0.7627368	0.9173647	1.380106141
1434506_at	ARID2	AT RICH INTERACTIVE DOMAIN 2 (ARID-RFX LIKE)	0.0117424	0.003747552	0.1574817	1.075736056
1448312_at	Pcsk2	proprotein convertase subtilisin/kexin type 2	0.01174943	0.03341148	0.14980959	1.146099541
1442595_at	---	---	0.01176371	0.1660262	0.458289	1.419506792
1430092_at	SERAC1	DNA SEGMENT, CHR 17, ERATO DOI 141, EXPRESSED	0.01178038	0.2261766	0.54687889	1.263678148
1425275_at	Asph	aspartate-beta-hydroxylase	0.01180459	0.04777687	0.1227291	0.793623584
1434955_at	March1	MEMBRANE-ASSOCIATED RING FINGER (C3HC4) 1	0.01196547	0.8627459	0.3642616	0.799880775
1452675_at	RBM22	RNA BINDING MOTIF PROTEIN 22	0.0119781	0.04328899	0.5188426	0.719935662
1460144_at	BC052040	CDNA SEQUENCE BC052040	0.01198893	0.1660718	0.2247996	0.689291989
1428623_at	PLXNA1	PLEXIN A1	0.01199749	0.5898525	0.4713705	1.231451184
1422492_at	CPOX	COPROPORPHYRINOGEN OXIDASE	0.0120276	0.01117468	0.8175256	0.812938346
1429881_at	Arhgap15	Rho GTPase activating protein 15	0.012063	0.009774036	0.4845403	0.544841638
1428852_at	DOCK3	DEDICATOR OF CYTO-KINESIS 3	0.01208976	0.2078136	0.3087257	1.214838535
1416812_at	TIA1	CYTOTOXIC GRANULE-ASSOCIATED RNA BINDING PROTEIN 1	0.01209346	1.22293E-06	0.31412259	0.770001929
1453596_at	ID2	INHIBITOR OF DNA BINDING 2	0.012134	0.000296304	0.1101049	1.100092237
1458182_at	RBM18	RNA BINDING MOTIF PROTEIN 18	0.01225067	0.9024916	0.05602554	0.856488074
1438183_x_at	SORD	SORBITOL DEHYDROGENASE	0.01227953	0.4123898	0.3619913	0.735981401
1427045_at	SYNPO	RIKEN CDNA 9030217H17 GENE	0.01231766	0.3791388	0.685056	1.325289962
1455697_at	E130113E03RIK	RIKEN CDNA E130113E03 GENE	0.01232554	0.006693433	0.4940704	1.153898452
1429772_at	Plxna2	plexin A2	0.01233122	0.1714314	0.1590314	1.27465191
1433495_at	GLT25D1	GLYCOSYLTRANSFERASE 25 DOMAIN CONTAINING 1	0.01241057	0.5386101	0.5755797	0.773519431
1439757_s_at	Epha4	Eph receptor A4	0.01242375	0.0986708	0.879589	1.257816783
1450788_at	SAA1	SERUM AMYLOID A 1	0.01242508	0.9436224	0.358947	2.700022197
1443075_at	---	---	0.0124917	1.23714E-08	0.3030345	1.137925324
1421604_a_at	Klf3	Kruppel-like factor 3 (basic)	0.01252311	0.004359326	0.6958096	0.72985769
1453424_at	Fyco1	FYVE and coiled-coil domain containing 1	0.01254797	0.002039498	0.417057	0.808553093
1418092_s_at	TRIP10	THYROID HORMONE RECEPTOR INTERACTOR 10	0.01255603	0.1361371	0.4195562	1.389709329
1419338_at	ICRFP703B1614Q5.5	ICRFP703B1614Q5.5 GENE	0.01259014	0.05049662	0.356665	2.451834829
1418754_at	ADCY8	ADENYLATE CYCLASE 8	0.01259583	0.5658471	0.7291411	1.425502206
1441267_at	RSRC1	ARGININE/SERINE-RICH COILED-COIL 1	0.01265857	0.01500432	0.44024	0.587086095
1444341_at	8030451F13RIK	RIKEN CDNA 8030451F13 GENE	0.0126806	0.1027779	0.4203061	0.66869667
1424208_at	PTGER4	PROSTAGLANDIN E RECEPTOR 4 (SUBTYPE EP4)	0.01268983	0.6059567	0.371943	1.530800538
1442308_at	SMYD4	SET AND MYND DOMAIN CONTAINING 4	0.01271032	0.9838952	0.4187257	0.788026174
1439189_at	D630023B12RIK	RIKEN CDNA D630023B12 GENE	0.01271178	0.0905241	0.7909966	0.810660628
1446791_at	---	---	0.01273989	0.004215441	0.1668491	0.786155577
1446748_at	2010007H06RIK	RIKEN CDNA 2010007H06 GENE	0.01275595	0.7765871	0.3436684	2.300545616
1434087_at	MTHFR	5,10-METHYLENETETRAHYDROFOLATE REDUCTASE	0.01278597	0.3675277	0.66508009	0.833748331
1441920_x_at	HTF9C	HPAII TINY FRAGMENTS LOCUS 9C	0.01278884	0.1411733	0.5324421	0.712332532
1435634_at	Pcmtd1	protein-L-isoaspartate (D-aspartate) O-methyltransferase domain containing 1	0.01280421	0.04489151	0.9106935	0.794608823
1419194_s_at	GMFG	GLIA MATURATION FACTOR, GAMMA	0.01280897	0.7684835	0.3353837	0.721115896
1429085_at	VEZF1	VASCULAR ENDOTHELIAL ZINC FINGER 1	0.01285014	0.5059602	0.1383296	1.170374927
1417783_at	Als2	amyotrophic lateral sclerosis 2 (juvenile) homolog (human)	0.01294882	0.1771731	0.881201	0.789773508
1421929_at	Epha4	Eph receptor A4	0.01303247	3.52601E-05	0.3472927	1.190462293
1420183_at	Lor	Loricrin	0.01303863	0.8440594	0.7494676	0.697272424
1446374_at	ARHGEF10	RHO GUANINE NUCLEOTIDE EXCHANGE FACTOR (GEF) 10	0.01307985	0.03038855	0.05270361	0.849646985
1438765_at	Dhx33	DEAH (Asp-Glu-Ala-His) box polypeptide 33	0.01308264	0.08838734	0.07327438	0.715156743
1452293_at	SNX19	RIKEN CDNA 3526401K03 GENE	0.01312444	0.3840151	0.9413396	0.827405286
1424859_at	HOMER3	HOMER HOMOLOG 3 (DROSOPHILA)	0.01316961	0.008990007	0.66730009	0.824553025
1436945_x_at	STIM1	STROMAL INTERACTION MOLECULE 1	0.01317349	0.01429928	0.3165296	0.712113158
1430369_at	Epb4.1	erythrocyte protein band 4.1	0.01321629	0.7697523	0.2981724	1.472606325
1440271_at	---	Transcribed locus	0.0132644	0.06418831	0.07477637	1.200969331
1457030_at	MIRG	MIRNA CONTAINING GENE	0.01329095	0.7507958	0.3299725	1.23057394
1455684_at	MAP3K9	MITOGEN-ACTIVATED PROTEIN KINASE KINASE KINASE 9	0.01331831	0.33378	0.6600525	1.230822873
1435880_at	ANKRD50	ANKRIN REPEAT DOMAIN 50	0.01336535	0.6514272	0.6897443	1.284733654
1441728_at	SCN1A	SODIUM CHANNEL, VOLTAGE-GATED, TYPE I, ALPHA	0.01337888	0.9013822	0.158498	1.301277391
1449953_at	Itgb3bp	integrin beta 3 binding protein (beta3-endonexin)	0.0133796	0.13894219	0.7719883	0.719333979
1451925_at	Eda	ectodysplasin-A	0.01339726	0.5828437	0.07736341	1.83469266
1456990_at	---	---	0.01342018	0.7562276	0.1448109	0.696565222
1430738_at	4833419K08RIK	RIKEN CDNA 4833419K08 GENE	0.0134345	0.06675873	0.08467183	2.098061353
1456348_x_at	PAQR5	PROGESTIN AND ADIPOQ RECEPTOR FAMILY MEMBER V	0.01347233	0.5641563	0.1027067	0.778667049
1424084_at	Rod1	ROD1 regulator of differentiation 1 (S. pombe)	0.01348009	0.03493304	0.1103933	0.621596633
1423065_at	DNMT3A	DNA METHYLTRANSFERASE 3A	0.01348391	0.06116435	0.6895624	1.429009394
1430388_a_at	SULF2	SULFATASE 2	0.01349999	0.4750469	0.4928491	1.214545892
1444747_at	Pcmtd1	Protein-L-isoaspartate (D-aspartate) O-methyltransferase domain containing 1	0.01352517	0.006977521	0.2477732	0.650849956
1453598_at	3110082D06Rik	RIKEN cDNA 3110082D06 gene	0.01357299	0.1355375	0.9175201	1.523259701
1447206_at	ARHGAP21	RHO GTPASE ACTIVATING PROTEIN 21	0.01360235	0.05893116	0.1179434	1.261437846
1451444_s_at	2700008B19RIK	RIKEN CDNA 2700008B19 GENE	0.01362392	0.0129461	0.9941113	0.797993699
1417173_at	CREBL1	CAMP RESPONSIVE ELEMENT BINDING PROTEIN-LIKE 1	0.01371955	4.6337E-07	0.7455977	0.798607023
1439897_at	---	---	0.01371965	0.04017024	0.06813692	1.206576573
1416762_at	S100A10	S100 CALCIUM BINDING PROTEIN A10 (CALPACTIN)	0.01371993	0.01684295	0.3287685	1.299148904
1450937_at	Lin7c	lin-7 homolog C (C. elegans)	0.01373073	0.0853929	0.3987667	0.801264052
1423747_a_at	PDK1	PYRUVATE DEHYDROGENASE KINASE, ISOENZYME 1	0.01374781	0.007593772	0.1338813	0.755670739
1451004_at	Acvr2a	activin receptor IIA	0.01374913	0.3838914	0.8045099	0.769664161
1438628_x_at	CNTN3	CONTACTIN 3	0.01379728	0.002239701	0.448181	0.772666476
1440073_at	RRM1	RIBONUCLEOTIDE REDUCTASE M1	0.01380055	0.2447044	0.1488068	2.241028382
1447617_at	CDK2	CYCLIN-DEPENDENT KINASE 2	0.01382606	0.2989106	0.1182302	3.054517972
1421926_at	Mapk11	mitogen-activated protein kinase 11	0.01383329	0.4824841	0.4484448	1.343048145
1428375_at	4932415G12RIK	RIKEN CDNA 4932415G12 GENE	0.01383947	0.09037941	0.780544	0.764836241
1432594_at	4933413I22RIK	RIKEN CDNA 4933413I22 GENE	0.0138584	0.2636569	0.9411127	2.51851225
1425010_at	ZFP119	ZINC FINGER PROTEIN 119	0.01387632	0.7981046	0.8712267	0.658739625
1433507_a_at	LOC637089	HIGH MOBILITY GROUP NUCLEOSOMAL BINDING DOMAIN 2	0.01389181	0.8211253	0.223103	0.744004922
1443612_at	---	---	0.01389776	0.4479313	0.4347423	0.786645515
1426877_a_at	Pb1	polybromo 1	0.01394502	0.3455968	0.7634952	0.820491156
1429078_a_at	COPS7A	COP9 (CONSTITUTIVE PHOTOMORPHOGENIC) HOMOLOG, SUBUNIT 7A (ARABIDOPSIS THALIANA)	0.01394532	1.36803E-05	0.1168861	1.043953177
1417436_at	LARGE	LIKE-GLYCOSYLTRANSFERASE	0.01395824	0.06732644	0.892414	1.285172779
1427692_a_at	CASK	CALCIUM/CALMODULIN-DEPENDENT SERINE PROTEIN KINASE (MAGUK FAMILY)	0.0139614	1.79549E-05	0.1562863	1.100588357
1420289_at	T25656	expressed sequence T25656	0.01397118	0.394721	0.2635998	0.583526187
1416740_at	COL5A1	PROCOLLAGEN, TYPE V, ALPHA 1	0.01398613	0.003232428	0.05794322	0.778433739
1450685_at	Arpp19	cAMP-regulated phosphoprotein 19	0.01400066	0.1306741	0.6595474	1.245127692
1417228_at	CAPN1	CALPAIN 1	0.01400178	0.0639191	0.339881	1.25443836
1454288_at	5830468K08RIK	RIKEN CDNA 5830468K08 GENE	0.01400285	0.2769832	0.2443179	1.716174596
1451531_at	CBWD1	COBW DOMAIN CONTAINING 1	0.01402523	0.4105769	0.654453	0.758079184
1459180_at	---	---	0.01402699	0.05135834	0.1985377	2.027637191
1422751_at	Tle1	transducin-like enhancer of split 1, homolog of Drosophila E(spl)	0.01404787	0.8861231	0.858172	0.823822733
1431225_at	Sox11	SRY-box containing gene 11	0.01406597	7.00562E-07	0.7765059	0.744799134
1459108_a_at	YEATS2	YEATS DOMAIN CONTAINING 2	0.01407026	0.000211893	0.2087979	0.818669486
1420859_at	Pkia	protein kinase inhibitor, alpha	0.0141286	0.4743714	0.2135804	0.672718033
1459809_x_at	1700063D05RIK	RIKEN CDNA 1700063D05 GENE	0.01413463	0.09773138	0.06615863	0.505485427
1416563_at	CTPS	CYTIDINE 5'-TRIPHOSPHATE SYNTHASE	0.01414101	0.2259231	0.6223433	0.755122644
1455301_at	BQ952480	EXPRESSED SEQUENCE BQ952480	0.01415615	0.4312971	0.759762	0.735476606
1424475_at	Camkk2	calcium/calmodulin-dependent protein kinase kinase 2, beta	0.01418074	0.5315966	0.8894917	1.419962316
1445076_at	---	---	0.01423578	0.3230298	0.91238	2.933123011
1422967_a_at	Tfrc	transferrin receptor	0.0142723	0.02220857	0.1594608	1.317093095
1415874_at	SPRY1	SPROUTY HOMOLOG 1 (DROSOPHILA)	0.01429356	0.03199422	0.5006394	0.770381988

1452484_at	CAR7	CARBONIC ANHYDRASE 7	0.01431174	1.6345E-05	0.7514179	1.146964853
1419169_at	MAPK6	MITOGEN ACTIVATED PROTEIN KINASE 4	0.01431187	0.04708881	0.1764764	1.192409937
1460321_at	CNTN4	AXONAL-ASSOCIATED CELL ADHESION MOLECULE	0.01431785	0.179471	0.09822935	1.188079283
1444742_at	ASB14	ANKYRIN REPEAT AND SOCS BOX-CONTAINING PROTEIN 14	0.0144356	0.001030066	0.6487079	0.484564309
1422875_at	CD84	CD84 ANTIGEN	0.01443918	0.9929265	0.3398166	0.820186924
1457446_at	OPCML	OPIOID BINDING PROTEIN/CELL ADHESION MOLECULE-LIKE	0.01444036	0.8030494	0.4204454	1.104101047
1427344_s_at	RASD2	RASD FAMILY, MEMBER 2	0.01447291	0.1717629	0.8071305	1.344278223
1434206_s_at	PPP2R5C	PROTEIN PHOSPHATASE 2, REGULATORY SUBUNIT B (B56), GAMMA ISOFORM	0.01447325	0.000450757	0.3590457	1.142261808
1440116_at	D630045J12RIK	RIKEN CDNA D630045J12 GENE	0.01448594	0.2661442	0.3893249	1.313795313
1420863_at	DCTN4	DYNACTIN 4	0.01448687	1.27269E-06	0.06585429	1.067049891
1429528_at	RAE1	RAE1 RNA EXPORT 1 HOMOLOG (S. POMBE)	0.01449422	0.000301838	0.130666	0.72743407
1441872_x_at	---	---	0.01449887	0.04852784	0.2163955	0.802254835
1431096_at	2810013E07RIK	RIKEN CDNA 2810013E07 GENE	0.01452173	0.4639346	0.7658116	1.214202141
1455114_at	UNG2	URACIL DNA GLYCOSYLASE 2	0.01459454	0.7137034	0.3995528	0.730796308
1432385_a_at	AGTPBP1	ATP/GTP BINDING PROTEIN 1	0.01462944	2.06832E-07	0.4513203	1.072536078
1426228_at	KRAS	V-KI-RAS2 KIRSTEN RAT SARCOMA VIRAL ONCOGENE HOMOLOG	0.01463861	0.1653777	0.1378439	0.277741895
1444857_at	C87312	EXPRESSED SEQUENCE C87312	0.01469541	0.31396629	0.2955202	0.32698207
1444700_at	Nrxn3	Neurexin III	0.01471879	0.01380275	0.560058	1.753430199
1440034_at	Stam2	signal transducing adaptor molecule (SH3 domain and ITAM motif) 2	0.01473302	0.8209325	0.3644538	1.46866864
1429943_at	CTBS	CHITOBIASE, DI-N-ACETYL-	0.01473453	0.00046217	0.27053709	0.741618334
1423503_at	JAM3	RIKEN CDNA 1110002N23 GENE	0.01477894	0.04578748	0.05102539	1.10210378
1416562_at	Gad1	glutamic acid decarboxylase 1	0.01486335	0.002307308	0.9763202	0.773319249
1457595_at	---	---	0.01489246	0.879279	0.3091564	2.210785839
1429429_s_at	Pcmtd1	protein-L-isoaspartate (D-aspartate) O-methyltransferase domain containing 1	0.01489564	0.3204702	0.06603377	0.830910131
1425388_a_at	TPK1	THIAMIN PYROPHOSPHOKINASE	0.01489723	0.8199376	0.4403206	0.803283868
1439101_at	D830007F02RIK	RIKEN CDNA D830007F02 GENE	0.01489964	0.06077438	0.2661805	0.607030132
1417878_at	E2F1	E2F TRANSCRIPTION FACTOR 1	0.01490231	0.1685346	0.2804586	1.650828737
1456967_at	TRIM66	KIAA0298 HYPOTHETICAL PROTEIN (HUMAN)	0.01492154	0.01807018	0.339114	1.346434297
1431749_a_at	Rasgrp1	RAS guanyl releasing protein 1	0.01494251	6.44541E-05	0.8605856	1.241384893
1456168_at	Ccnjl	Cyclin J-like	0.01495803	0.9546377	0.3736506	0.727980699
1421595_at	9630031F12Rik	RIKEN cDNA 9630031F12 gene	0.01497003	0.02158468	0.6735401	0.80373569
1423318_at	RAD18	RAD18 HOMOLOG (S. CEREVISIAE)	0.01501785	0.04143731	0.6917924	0.549957312
1454212_x_at	4930453O09RIK	RIKEN CDNA 4930453O09 GENE	0.01503681	0.5730456	0.1389613	0.584878376
1457166_at	AA536749	EXPRESSED SEQUENCE AA536749	0.01504068	0.0023647	0.6864146	1.315544385
1452117_a_at	FYB	FYN BINDING PROTEIN	0.01504985	0.07760122	0.3289472	1.448382732
1457615_at	---	---	0.01506807	0.8206508	0.4927571	0.541770103
1446069_at	Ablim3	Actin binding LIM protein family, member 3	0.0151077	0.0400133	0.05633661	0.686191583
1453250_at	4921511C04RIK	RIKEN CDNA 4921511C04 GENE	0.0151112	0.7075807	0.796177	0.431458103
1442581_at	KSR1	KINASE SUPPRESSOR OF RAS 1	0.01512604	0.1334756	0.1570192	0.818879994
1455493_at	SYNE1	SYNAPTIC NUCLEAR ENVELOPE 1	0.01512654	0.6359034	0.5916586	1.261658421
1426934_at	NHSL1	NHS-LIKE 1	0.01519089	0.008125816	0.6323646	0.697033257
1443359_at	ODZ4	ODD OZ/TEN-M HOMOLOG 4 (DROSOPHILA)	0.01524488	0.07037986	0.1410952	1.449365718
1424760_a_at	SMYD2	RIKEN CDNA 1110020E07 GENE	0.01527202	0.1856618	0.16307749	1.122677617
1441375_at	LRIG1	LEUCINE-RICH REPEATS AND IMMUNOGLOBULIN-LIKE DOMAINS 1	0.01528033	0.9164515	0.5899593	1.799015497
1442035_at	Chrna5	cholinergic receptor, nicotinic, alpha polypeptide 5	0.01530184	0.4003324	0.5890455	0.736293759
1452777_a_at	6330412F12RIK	RIKEN CDNA 6330412F12 GENE	0.01530407	0.00016467	0.1316794	1.085070713
1434321_at	6620401K05RIK	RIKEN CDNA 6620401K05 GENE	0.0153168	0.1996225	0.4058252	1.216024306
1426832_at	6330505F04RIK	RIKEN CDNA 6330505F04 GENE	0.01538948	0.0106948	0.802542	0.785471998
1453069_at	PIK3CB	PHOSPHATIDYLINOSITOL 3-KINASE, CATALYTIC, BETA POLYPEPTIDE	0.01539738	0.006825302	0.1882957	1.191792039
1445217_at	A730073F16RIK	RIKEN CDNA A730073F16 GENE	0.01541555	0.01876697	0.2043241	1.65007575
1440277_at	AU041474	EXPRESSED SEQUENCE AU041474	0.01547996	0.03404367	0.14491589	0.842885074
1446208_at	TDRD5	TUDOR DOMAIN CONTAINING 5	0.01548804	0.2085974	0.1084911	0.547300452
1459978_x_at	Ccnjl	cyclin J-like	0.01550218	0.5748562	0.9105308	0.752540145
1440932_at	TRIM17	TRIPARTITE MOTIF PROTEIN 17	0.01552962	0.9375215	0.4004027	0.777947833
1432111_at	4930542D17RIK	RIKEN CDNA 4930542D17 GENE	0.01554459	0.04333607	0.4215917	0.769665532
1416316_at	SLC27A2	SOLUTE CARRIER FAMILY 27 (FATTY ACID TRANSPORTER), MEMBER 2	0.01556671	0.2449473	0.1123171	0.473647775
1419966_at	TUBB2B	TUBULIN, BETA 2B	0.015625819	0.2392809	0.09628279	1.603817284
1441178_at	DTWD2	DTW DOMAIN CONTAINING 2	0.01564788	0.8189462	0.3981034	0.638035108
1454075_s_at	NUDT13	NUDIX (NUCLEOSIDE DIPHOSPHATE LINKED MOIETY X)-TYPE MOTIF 13	0.01568349	0.03636165	0.1642058	0.848144477
1427946_s_at	DPYD	DIHYDROPYRIMIDINE DEHYDROGENASE	0.01572054	0.915299	0.408219	0.817879648
1426683_at	CNOT6	CCR4-NOT TRANSCRIPTION COMPLEX, SUBUNIT 6	0.01573219	0.8796525	0.4228852	1.138945437
1421094_at	ZBTB33	ZINC FINGER AND BTB DOMAIN CONTAINING 33	0.01579089	0.01515471	0.7072626	1.426725854
1451791_at	TFPI	TISSUE FACTOR PATHWAY INHIBITOR	0.01579318	0.05788872	0.3250552	0.829542009
1418383_at	Apcdd1	adenomatosis polyposis coli down-regulated 1	0.01579922	0.001695823	0.1092744	0.70776455
1417011_at	Sdc2	syndecan 2	0.0158007	0.00218757	0.09854159	1.148793016
1420416_at	SEMA3A	SEMA DOMAIN, IMMUNOGLOBULIN DOMAIN (IG), SHORT BASIC DOMAIN, SECRETED, (SEMAPHORIN) 3A	0.015827339	0.000871091	0.3420789	1.201629872
1433615_at	TMEM117	TRANSMEMBRANE PROTEIN 117	0.01585049	0.3882041	0.1792634	0.644272715
1450193_at	HCN1	HYPERPOLARIZATION-ACTIVATED, CYCLIC NUCLEOTIDE-GATED K+ 1	0.01586342	0.000373787	0.1684061	1.107649274
1453287_at	5730557B15RIK	RIKEN CDNA 5730557B15 GENE	0.015889859	0.005821053	0.2038549	1.187563363
1434098_at	GLRA2	GLYCINE RECEPTOR, ALPHA 2 SUBUNIT	0.01592045	0.414067	0.1828674	0.730751688
1426223_at	2810439F02Rik	RIKEN cDNA 2810439F02 gene	0.01598469	0.2377252	0.5143829	0.785826057
1459906_at	Dgkh	Diacylglycerol kinase, eta	0.01603166	0.5070808	0.28661759	0.730719341
1421973_at	Gfra1	glial cell line derived neurotrophic factor family receptor alpha 1	0.016057519	0.263157	0.7180497	0.682118704
1429011_x_at	SNRP70	U1 SMALL NUCLEAR RIBONUCLEOPROTEIN POLYPEPTIDE A	0.01608244	0.1820408	0.3639839	0.523017099
1454666_at	Klf3	Kruppel-like factor 3 (basic)	0.01609457	0.9540296	0.8136163	0.759610118
1459746_at	R3hdm1	R3H domain 1 (binds single-stranded nucleic acids)	0.01613615	0.000554198	0.3525642	1.200064192
1427528_a_at	Epha7	Eph receptor A7	0.01614762	0.000154818	0.8980062	0.668780328
1427735_a_at	ACTA1	ACTIN, ALPHA 1, SKELETAL MUSCLE	0.01620508	0.01732737	0.3619572	1.205873983
1421735_a_at	ST8SIA5	SIALYLTRANSFERASE 8 (ALPHA-2, 8-SIALYLTRANSFERASE) E	0.01620865	0.0954948	0.2621539	1.151557479
1460591_at	ESR1	ESTROGEN RECEPTOR 1 (ALPHA)	0.01622349	0.192405	0.9813805	0.628153541
1430733_at	4932441P12RIK	RIKEN CDNA 4932441P12 GENE	0.0162777	0.2094016	0.6979641	0.540991604
1452344_at	Synj2	synaptojanin 2	0.01632672	0.7435198	0.3027498	1.235971778
1438815_at	HIST2H2AA2	HISTONE 2, H2AA1	0.01635556	0.03937298	0.9123403	0.726772882
1453407_at	1700018L24RIK	RIKEN CDNA 1700018L24 GENE	0.01644994	0.01418599	0.2029402	0.740170171
1430353_at	GLIS3	GLIS FAMILY ZINC FINGER 3	0.01645526	0.7419447	0.7248987	0.673622779
1415861_at	TYRP1	TYROSINASE-RELATED PROTEIN 1	0.01646553	0.2727063	0.7099485	0.597193976
1440077_at	6330549D23RIK	RIKEN CDNA 6330549D23 GENE	0.01647839	0.2929986	0.3010587	0.638600979
1442449_at	SLC6A2	SOLUTE CARRIER FAMILY 6 (NEUROTRANSMITTER TRANSPORTER, NORADRENALIN), MEMBER 2	0.01650081	0.4121415	0.9381406	2.207945309
1447112_s_at	CRYL1	CRYSTALLIN, LAMDA 1	0.01650236	0.721475	0.1156236	0.773282717
1458836_at	Calb1	calbindin-28K	0.016543849	0.01524835	0.6577171	0.616622654
1430045_at	Tsnax	translin-associated factor X	0.01662183	0.2599799	0.9574008	1.506253266
1434016_at	ZNRF2	ZINC AND RING FINGER 2	0.01662336	0.002959084	0.1610133	0.823638588
1452966_at	9130430L19RIK	RIKEN CDNA 9130430L19 GENE	0.01665849	0.5681591	0.751809	0.774662698
1453126_at	LRFN2	LEUCINE RICH REPEAT AND FIBRONECTIN TYPE III DOMAIN CONTAINING 2	0.01668157	0.996656	0.2912194	1.236770691
1419237_at	USP29	UBIQUITIN SPECIFIC PEPTIDASE 29	0.01672061	0.003150871	0.3626081	1.293752648
1455083_at	AI315324	EXPRESSED SEQUENCE AI315324	0.0167617	0.002828884	0.08704945	1.214978279
1452983_at	CEP57	RIKEN CDNA 4921510P06 GENE	0.016769679	0.0132504	0.5781276	0.785004514
1436450_at	---	---	0.0168838	0.3446799	0.3444766	1.115395222
1427498_a_at	Spag5	sperm associated antigen 5	0.01691875	0.4778448	0.9717535	1.605473369
1431380_at	5730409L17RIK	RIKEN CDNA 5730409L17 GENE	0.01697102	0.07675586	0.256042	1.402907518
1418060_a_at	MAPK7	MITOGEN ACTIVATED PROTEIN KINASE 7	0.01707227	0.03714782	0.578978	0.598941402
1441564_at	GM47	GENE MODEL 47, (NCBI)	0.01709049	0.661638	0.3793446	1.877501083
1430106_at	1700126L10RIK	RIKEN CDNA 1700126L10 GENE	0.0171548	0.1889752	0.6087091	1.964073747
1434754_at	GARNL4	GTPASE ACTIVATING RANGAP DOMAIN-LIKE 4	0.01716358	0.65522329	0.1750043	0.773372518
1427069_at	Fbxo28	F-box protein 28	0.01716929	0.03887524	0.4217065	1.150395175
1451355_at	ASAH3L	RIKEN CDNA 2410116I05 GENE	0.0171984	0.003413011	0.2571287	0.86020449
1438105_at	LOC632687	HYPOTHETICAL PROTEIN LOC632686	0.01724267	0.722535	0.1750495	0.749549474
1427439_s_at	PRMT5	PROTEIN ARGININE N-METHYLTRANSFERASE 5	0.01731887	0.9501491	0.2270921	1.116606054
1441628_at	DIAP3	DIAPHANOUS HOMOLOG 3 (DROSOPHILA)	0.01733423	0.002730623	0.5877269	0.405660315
1416561_at	Gad1	glutamic acid decarboxylase 1	0.01733459	0.3499238	0.3340359	0.761336322
1447101_at	RNF122	RING FINGER PROTEIN 122	0.01734843	0.000495335	0.2696847	0.575800521
1431109_at	5430406M13RIK	RIKEN CDNA 5430406M13 GENE	0.01736744	0.3367901	0.1273649	1.181394139
1432125_at	Ap3m2	adaptor-related protein complex 3, mu 2 subunit	0.01739849	0.4860998	0.800546	0.444382529
1417343_at	FXYD6	FXYD DOMAIN-CONTAINING ION TRANSPORT REGULATOR 6	0.0174097	0.1068963	0.1809808	1.209158443
1454785_at	DUSP11	DUAL SPECIFICITY PHOSPHATASE 11 (RNA/RNP COMPLEX 1-INTERACTING)	0.01743046	0.001788407	0.0827133	1.075322855
1440838_at	AI852064	EXPRESSED SEQUENCE AI852064	0.01743422	0.2569937	0.807891	1.261429059
1424519_at	MTG1	MITOCHONDRIAL GTPASE 1 HOMOLOG (S. CEREVISIAE)	0.01744866	0.2151717	0.848991	0.8007185
1452380_at	Epha7	Eph receptor A7	0.01745089	0.6234207	0.3116857	0.599198785
1444524_at	---	---	0.01745508	0.08316285	0.4708008	1.700428932
1455436_at	Diras2	DIRAS family, GTP-binding RAS-like 2	0.01745933	0.6031919	0.3407843	1.196763953
1441599_at	2900075B16RIK	RIKEN CDNA 2900075B16 GENE	0.0174618	0.01913852	0.1387336	1.349230851
1449670_x_at	GPR137B	G PROTEIN-COUPLED RECEPTOR 137B	0.01746477	0.000483647	0.3376938	0.868388736
1453513_at	RNPC3	RNA-BINDING REGION (RNP1, RRM) CONTAINING 3	0.01748074	0.119851	0.06187992	0.778401963
1433715_at	CPNE7	COPINE VII	0.01748883	0.0940512	0.789267	1.224785087
1460668_at	GAL	GALANIN	0.01753173	0.9553096	0.3713278	1.336461836
1425249_a_at	Tyro3	TYRO3 protein tyrosine kinase 3	0.01754221	0.4723163	0.805244	1.40394627
1430811_a_at	CDCA1	CELL DIVISION CYCLE ASSOCIATED 1	0.01755066	0.9833881	0.5629256	0.778251393
1450577_at	SSTR3	SOMATOSTATIN RECEPTOR 3	0.0175645	0.04830426	0.1904954	1.264923469
1425841_at	SLC26A7	SOLUTE CARRIER FAMILY 26, MEMBER 7	0.01757021	0.2476997	0.8383738	0.41707649
1430179_at	4921507G05RIK	RIKEN CDNA 4921507G05 GENE	0.01757222	0.682023	0.13476759	1.599864814
1423007_a_at	Gfra2	glial cell line derived neurotrophic factor family receptor alpha 2	0.01758845	0.493018	0.366258	0.730057595
1423876_at	AI450540	expressed sequence AI450540	0.0175901	0.9251487	0.3074075	1.094995182
1436099_at	AI836003	EXPRESSED SEQUENCE AI836003	0.01760237	0.5333115	0.3110383	1.349030274
1451191_at	CRABP2	CELLULAR RETINOIC ACID BINDING PROTEIN II	0.01764716	0.02277456	0.4815211	2.179716856
1442316_x_at	TRP53BP1	TRANSFORMATION RELATED PROTEIN 53 BINDING PROTEIN 1	0.01768926	0.4049944	0.08044415	0.837602558
1455411_at	AQP11	AQUAPORIN 11	0.01769052	0.03697794	0.5266327	0.735274258
1439173_at	Hook1	hook homolog 1 (Drosophila)	0.01771374	0.1196096	0.7064801	0.769348382
1430640_a_at	PRKAR2B	PROTEIN KINASE, CAMP DEPENDENT REGULATORY, TYPE II BETA	0.01773629	0.831259	0.3659341	1.219958893
1452447_at	0610007P08RIK	RIKEN CDNA 1700019D06 GENE	0.0177469	0.919678	0.3381997	0.789796385
1418266_at	ALOX12B	ARACHIDONATE 12-LIPOXYGENASE, 12R TYPE	0.01778932	0.9991971	0.7474094	1.704154805
1426360_at	ZC3H11A	ZINC FINGER CCCH TYPE CONTAINING 11A	0.01779375	0.07427541	0.9850557	1.256095543
1436109_at	AI317395	EXPRESSED SEQUENCE AI317395	0.01780841	0.5986037	0.5024261	0.576622848
1450080_at	CXX1C	CAAX BOX 1 HOMOLOG C (HUMAN)	0.01782597	0.3423157	0.1358927	1.133137008
1449158_at	KCNK2	POTASSIUM CHANNEL, SUBFAMILY K, MEMBER 2	0.0178392	0.024507	0.399569	0.74666532
1427652_x_at	Synj2	synaptojanin 2	0.01784046	0.8181505	0.6455722	1.464996652
1447596_at	---	---	0.01787589	0.9053386	0.630343	1.65893871
1447870_x_at	1110002E22RIK	RIKEN CDNA 1110002E22 GENE	0.01792578	0.04451181	0.2468696	0.692314046
1451404_at	ORF19	OPEN READING FRAME 19	0.01795172	0.3352077	0.2038972	0.810568267
1457702_at	Gpr12	G-protein coupled receptor 12	0.01796567	0.950421	0.2239847	0.611421003
1435829_at	B930008K04RIK	RIKEN CDNA B930008K04 GENE	0.01800803	0.01379413	0.4965056	0.751605707
1421846_at	WSB2	WD REPEAT AND SOCS BOX-CONTAINING 2	0.01801751	4.52623E-05	0.4687176	1.099794229
1421187_at	CCR2	CHEMOKINE (C-C MOTIF) RECEPTOR 2	0.01804587	0.01831181	0.07608514	1.495025193
1457365_at	---	---	0.0180476	0.7734478	0.7012486	0.628089382
1424221_at	SUSD4	SUSHI DOMAIN CONTAINING 4	0.01809136	0.00177401	0.3286464	1.095939132
1424415_s_at	Spon1	spondin 1, (f-spondin) extracellular matrix protein	0.01809771	0.5764333	0.2115694	0.75801622
1445327_at	CYFIP1	CYTOPLASMIC FMR1 INTERACTING PROTEIN 1	0.01811423	0.08282051	0.5389537	2.006757501
1440042_at	ANK2	EXPRESSED SEQUENCE AI835472	0.01812772	0.1706738	0.06776276	1.513506074
1459194_at	2210408I21RIK	RIKEN CDNA 2210408I21 GENE	0.01815104	0.1707576	0.06927703	1.151631145
1448775_at	---	---	0.01823324	0.8397998	0.9777692	1.484230795

1428859_at	PAOX	POLYAMINE OXIDASE (EXO-N4-AMINO)	0.01823749	0.08577803	0.6878419	0.735785119
1421101_a_at	Ldb2	LIM domain binding 2	0.01823874	0.2941424	0.242712	1.332026434
1454030_at	SAPS3	SAPS DOMAIN FAMILY, MEMBER 3	0.01827146	0.0170514	0.4595935	1.171757576
1419167_at	PRAP1	PROLINE-RICH ACIDIC PROTEIN 1	0.01828251	0.2227828	0.0673243	0.7481639
1439994_at	---	---	0.01828729	0.3089617	0.9609834	0.777920935
1416660_at	EIF3S10	EUKARYOTIC TRANSLATION INITIATION FACTOR 3, SUBUNIT 10 (THETA)	0.01829943	1.24896E-06	0.07427238	1.05691321
1458186_at	---	---	0.01831599	0.7857885	0.3579565	0.853084698
1421457_a_at	SAMSN1	SAM DOMAIN, SH3 DOMAIN AND NUCLEAR LOCALIZATION SIGNALS, 1	0.01834855	0.104454	0.4912259	0.78783046
1457935_at	TSHZ2	SEROLOGICALLY DEFINED COLON CANCER ANTIGEN 33 LIKE	0.01838124	0.16578209	0.2995518	0.425012886
1456482_at	PIK3R3	PHOSPHATIDYLINOSITOL 3 KINASE, REGULATORY SUBUNIT, POLYPEPTIDE 3 (P55)	0.01842876	0.000152836	0.1293815	1.083235465
AFFX-TransRecMur/X57349_M_at	Tfrc	transferrin receptor	0.01843252	0.000649431	0.284183	1.18981615
1416940_at	PPIF	PEPTIDYLPROLYL ISOMERASE F (CYCLOPHILIN F)	0.01847919	0.004216518	0.7092992	0.853822412
1439764_s_at	C330012H03RIK	RIKEN CDNA C330012H03 GENE	0.01849834	0.6343072	0.5363307	2.715914309
1443589_at	DXERTD242E	DNA SEGMENT, CHR X, ERATO DOI 242, EXPRESSED	0.01850068	0.1386918	0.4520099	1.361778935
1449920_at	CYP19A1	CYTOCHROME P450, FAMILY 19, SUBFAMILY A, POLYPEPTIDE 1	0.01850301	0.1083865	0.8238803	0.496696961
1427748_at	IGK-V1	IMMUNOGLOBULIN KAPPA CHAIN VARIABLE 1 (V1)	0.01853113	0.4124349	0.3607899	0.487672897
1458180_at	---	---	0.01854075	0.05096164	0.7854399	1.500892077
1435655_at	RPL12	RIBOSOMAL PROTEIN L12	0.01855886	0.335023	0.6139984	0.848376515
1440343_at	RPS6KA5	RIBOSOMAL PROTEIN S6 KINASE, POLYPEPTIDE 5	0.0185655	0.01676405	0.1223354	0.700042526
1447120_at	BTRC	BETA-TRANSDUCIN REPEAT CONTAINING PROTEIN	0.01858908	0.04136528	0.3295764	1.289778215
1440340_at	SDF4	STROMAL CELL DERIVED FACTOR 4	0.01864115	0.04359851	0.6454575	0.736047112
1457229_at	GPR173	G-PROTEIN COUPLED RECEPTOR 173	0.01866779	0.09278871	0.4608574	0.789811036
1454822_x_at	Apcdd1	adenomatosis polyposis coli down-regulated 1	0.01868536	0.6066306	0.4263041	0.609872882
1451283_at	1810073G14RIK	RIKEN CDNA 1810073G14 GENE	0.01874509	0.006651688	0.4190895	0.759478469
1449677_s_at	TMEM38B	TRANSMEMBRANE PROTEIN 38B	0.01879477	0.1041784	0.2259673	0.899717731
1420790_x_at	KLRA16	KILLER CELL LECTIN-LIKE RECEPTOR, SUBFAMILY A, MEMBER 16	0.0187984	0.02225696	0.4511391	2.622256673
1419043_a_at	IIGP1	INTERFERON INDUCIBLE GTPASE 1	0.01879971	0.6179097	0.8797763	0.687481258
1440063_at	FARSLA	PHENYLALANINE-TRNA SYNTHETASE-LIKE, ALPHA SUBUNIT	0.01887109	0.8469835	0.2128387	2.211737573
1460490_at	MRPL15	MITOCHONDRIAL RIBOSOMAL PROTEIN L15	0.0189026	0.06872575	0.652925	2.336465485
1421794_at	KLRC3	KILLER CELL LECTIN-LIKE RECEPTOR SUBFAMILY C, MEMBER 3	0.01893049	0.4488791	0.1667158	0.311654731
1448469_at	NID1	NIDOGEN 1	0.01893388	0.3952305	0.8182	2.642538814
1429712_at	LOC628308	ETHANOL INDUCED 1	0.01897389	0.000225497	0.3300607	0.775862122
1450949_at	KATNA1	KATANIN P60 (ATPASE-CONTAINING) SUBUNIT A1	0.01898373	0.03095386	0.2815678	1.144462568
1433300_at	2900005I04RIK	RIKEN CDNA 2900005I04 GENE	0.01899848	0.007940052	0.2607495	1.286554328
1430710_at	---	---	0.01899884	0.1059774	0.08009792	0.643110827
1441544_at	CNNM3	CYCLIN M3	0.01903639	0.7204962	0.1534982	1.203950972
1450047_at	Hs6st2	heparan sulfate 6-O-sulfotransferase 2	0.01909249	0.7908552	0.4333547	1.229002545
1440120_at	GNB2L1	GUANINE NUCLEOTIDE BINDING PROTEIN (G PROTEIN), BETA POLYPEPTIDE 2 LIKE 1	0.01909661	0.00431812	0.4230385	0.809058331
1442064_at	AW556556	EXPRESSED SEQUENCE AW556556	0.01910397	0.7949696	0.2607881	1.528644206
1425126_at	Ncam1	neural cell adhesion molecule 1	0.01914166	0.4554274	0.6655837	0.553144092
1424161_at	DDX27	DEAD (ASP-GLU-ALA-ASP) BOX POLYPEPTIDE 27	0.0191584	0.1579441	0.1656367	1.133436274
1431294_at	SPEER7-PS1	SPERMATOGENESIS ASSOCIATED GLUTAMATE (E)-RICH PROTEIN 7, PSEUDOGENE 1	0.01917082	0.6418098	0.758766	2.14906414
1445870_at	CDC42EP2	RIKEN CDNA 1110008C05 GENE	0.01919322	0.5166621	0.13493459	0.845391265
1424979_at	APH1A	ANTERIOR PHARYNX DEFECTIVE 1A HOMOLOG (C. ELEGANS)	0.01919754	0.000217458	0.3647063	1.10213786
1447204_at	Map4k5	Mitogen-activated protein kinase kinase kinase kinase 5	0.01930691	0.00157247	0.9008677	1.309449193
1437573_at	PPA2	PYROPHOSPHATASE (INORGANIC) 2	0.01931903	0.104478	0.2690481	0.682800545
1433141_at	4921504P05RIK	RIKEN CDNA 4921504P05 GENE	0.01932159	0.2885072	0.1521372	0.644831691
1439673_at	E230008O15RIK	RIKEN CDNA E230008O15 GENE	0.01932803	0.3311669	0.5608405	0.758480169
1439990_at	---	---	0.01933061	0.02407448	0.906664	0.823973336
1415909_at	STIP1	STRESS-INDUCED PHOSPHOPROTEIN 1	0.01933507	0.1224743	0.5030212	1.163357991
1421481_at	TNFRSF9	TUMOR NECROSIS FACTOR RECEPTOR SUPERFAMILY, MEMBER 9	0.01939674	0.32266089	0.646244	0.672251324
1457993_at	---	---	0.01941053	0.00184993	0.09465058	1.594962958
1445855_at	A030007L17RIK	RIKEN CDNA A030007L17 GENE	0.0194528	0.4267932	0.07921742	0.665528542
1440751_at	EP400	E1A BINDING PROTEIN P400	0.0194568	0.01272694	0.07513051	1.199674912
1435874_at	---	---	0.01949411	0.2073425	0.5435446	0.865590188
1447123_at	---	---	0.01950576	0.2205646	0.32016489	1.372723534
1423875_at	AI450540	expressed sequence AI450540	0.01950853	0.005773862	0.1922667	1.138499138
1432511_s_at	CEP27	CENTROSOMAL PROTEIN 27	0.01952073	0.146881	0.9970302	0.743107869
1430849_a_at	CGRRF1	CELL GROWTH REGULATOR WITH RING FINGER DOMAIN 1	0.01952386	0.6704484	0.3710377	1.168099893
1455095_at	HIST2H2BE	SIMILAR TO HISTONE H2B-616	0.01956242	0.8168337	0.2217677	0.818648388
1431433_at	SBF2	MYOTUBULARIN RELATED PROTEIN 13	0.0195808	0.8352656	0.3999933	1.607431738
1428748_at	5830428H23RIK	CDNA SEQUENCE BC055757	0.01958731	7.64126E-05	0.2709259	0.801030835
1433113_at	4921504P20RIK	RIKEN CDNA 4921504P20 GENE	0.01961413	0.09474632	0.05176088	0.456400812
1445062_at	ELOVL6	ELOVL FAMILY MEMBER 6, ELONGATION OF LONG CHAIN FATTY ACIDS (YEAST)	0.0196585	0.3146257	0.601024	1.443458394
1442725_at	---	---	0.01967741	0.07864452	0.4961774	0.787037504
1449141_at	FBLIM1	FILAMIN BINDING LIM PROTEIN 1	0.01969582	0.2344514	0.1783657	0.745171155
1429760_at	RPS6KA6	RIBOSOMAL PROTEIN S6 KINASE POLYPEPTIDE 6	0.01972372	0.629596	0.1084929	1.326926347
1457047_at	---	---	0.01977105	0.000852883	0.3841347	0.824445999
1440848_at	1110028C15RIK	RIKEN CDNA 1110028C15 GENE	0.0197868	0.8771729	0.07837208	0.633701121
1460570_at	PGBD5	PIGGYBAC TRANSPOSABLE ELEMENT DERIVED 5	0.019807	0.1013621	0.9422745	0.821717631
1458731_at	---	---	0.01983396	0.02438738	0.6998844	0.501414289
1460105_at	B3GALNT2	UDP-GALNAC:BETAGLCNAC BETA 1,3-GALACTOSAMINYLTRANSFERASE, POLYPEPTIDE 2	0.01986148	0.8389674	0.6822635	2.971798596
1437979_at	ZCCHC2	ZINC FINGER, CCHC DOMAIN CONTAINING 2	0.01992923	0.3409339	0.06879364	1.528108427
1428276_at	1110065L07RIK	RIKEN CDNA 1110065L07 GENE	0.01994112	0.7495248	0.8110994	0.78343747
1439543_at	1110064A23RIK	RIKEN CDNA 1110064A23 GENE	0.01996891	0.59824029	0.5249427	1.447372688
1423401_at	ETV6	ETS VARIANT GENE 6 (TEL ONCOGENE)	0.01999499	0.02752672	0.08721028	0.811140779
1431322_at	Igsf3	immunoglobulin superfamily, member 3	0.0200137	0.0121489	0.5506392	1.500795169
1429231_at	---	---	0.02004442	0.4089222	0.265491	0.759267007
1458606_at	---	---	0.02008465	0.1945443	0.05909001	1.939198185
1437052_s_at	Slc2a3	solute carrier family 2 (facilitated glucose transporter), member 3	0.02008814	0.1262378	0.7852876	0.88101547
1423353_at	CRISPLD1	CYSTEINE-RICH SECRETORY PROTEIN LCCL DOMAIN CONTAINING 1	0.02014796	0.1453471	0.372132	1.179171986
1430073_at	2900016B01RIK	RIKEN CDNA 2900016B01 GENE	0.02015798	0.6908706	0.7439287	1.410962549
1456099_at	D930017J03RIK	RIKEN CDNA D930017J03 GENE	0.02016927	0.3225094	0.7916108	1.600367524
1448478_at	TRFP	TRF (TATA BINDING PROTEIN-RELATED FACTOR)-PROXIMAL PROTEIN HOMOLOG (DROSOPHILA)	0.0202009	8.20967E-07	0.0720245	0.78663466
1418131_at	SAMHD1	SAM DOMAIN AND HD DOMAIN, 1	0.02020655	0.08836287	0.9734223	0.833263403
1460073_at	---	---	0.02020709	0.0995552	0.1602352	0.881428227
1418493_a_at	SNCA	SYNUCLEIN, ALPHA	0.02025004	0.05684284	0.9688417	0.755997744
1421018_at	1110018J18RIK	RIKEN CDNA 1110018J18 GENE	0.02033117	0.4189331	0.2223555	1.236506121
1444924_at	2700092H06RIK	RIKEN CDNA 2700092H06 GENE	0.02036182	0.5034459	0.3476221	0.726270964
1438857_x_at	IRAK1	INTERLEUKIN-1 RECEPTOR-ASSOCIATED KINASE 1	0.02041488	0.003301387	0.2544467	0.829544601
1453017_at	D5ERTD585E	DNA SEGMENT, CHR 5, ERATO DOI 585, EXPRESSED	0.02044003	0.1413495	0.2576573	1.160896829
1445953_at	D17ERTD191E	DNA SEGMENT, CHR 17, ERATO DOI 191, EXPRESSED	0.02044096	0.4632935	0.2771658	0.598302777
1455185_s_at	PHF16	PHD FINGER PROTEIN 16	0.02046398	0.8089611	0.5411915	0.774458153
1455373_at	---	---	0.02047563	0.06632018	0.2071783	1.251599777
1418831_at	PKP3	PLAKOPHILIN 3	0.02047851	0.4013533	0.05000294	0.774933397
1451624_a_at	PHOSPHO2	PHOSPHATASE, ORPHAN 2	0.02052352	0.1210071	0.1696556	1.158466147
1416981_at	FOXO1	FORKHEAD BOX O1	0.02053078	0.342918	0.3230763	1.164541559
1443868_at	ATP13A3	ATPASE TYPE 13A3	0.02057612	0.1733308	0.2347332	0.418084001
1437248_at	2700049A03RIK	RIKEN CDNA 2700049A03 GENE	0.02059532	0.57957479	0.9824974	1.58340143
1430853_a_at	3110045G13RIK	RIKEN CDNA 3110045G13 GENE	0.02062133	0.4454934	0.3908144	0.527837438
1416490_at	TMED6	TRANSMEMBRANE EMP24 PROTEIN TRANSPORT DOMAIN CONTAINING 6	0.02062897	0.8171087	0.1494577	2.230021577
1437506_at	ADAMTS6	A DISINTEGRIN-LIKE AND METALLOPEPTIDASE (REPROLYSIN TYPE) WITH THROMBOSPONDIN TYPE 1 MOTIF, 6	0.02065974	0.02206258	0.07163975	1.727519392
1446347_at	---	---	0.0206959	0.8169594	0.06842788	1.476778957
1428618_at	Hcfc2	host cell factor C2	0.02070142	0.000576926	0.7860073	0.78228264
1459329_at	TTC7	RIKEN CDNA 1110035E02 GENE	0.02070876	0.07034312	0.3339337	1.497638871
1416287_at	RGS4	REGULATOR OF G-PROTEIN SIGNALING 4	0.02071291	0.04330961	0.59556659	1.24622379
1454418_at	5330421C15RIK	RIKEN CDNA 5330421C15 GENE	0.02073109	0.1021767	0.5659246	1.248022171
1418591_at	Dnaja4	DnaJ (Hsp40) homolog, subfamily A, member 4	0.0207402	0.6987843	0.1269294	1.199877468
1457388_at	A830080L01RIK	RIKEN CDNA A830080L01 GENE	0.02074854	0.8832785	0.3000578	2.92751824
1443437_at	GM761	GENE MODEL 761, (NCBI)	0.02080457	0.3928341	0.1376649	0.618218897
1439804_at	2310015A10RIK	RIKEN CDNA 2310015A10 GENE	0.02081966	0.5755427	0.8703008	0.804357697
1443104_at	TBC1D9	TBC1 DOMAIN FAMILY, MEMBER 9	0.02084059	0.8568934	0.2309577	1.210128495
1422334_a_at	SFTPA1	SURFACTANT ASSOCIATED PROTEIN A1	0.02084776	0.6403206	0.8862631	3.099265466
1446154_at	BDH1	3-HYDROXYBUTYRATE DEHYDROGENASE, TYPE 1	0.02084929	0.8249376	0.3029006	1.540763731
1429717_at	Ipo11	importin 11	0.02088745	0.7583778	0.9628038	0.472487421
1460627_at	D130067I03RIK	RIKEN CDNA D130067I03 GENE	0.02091137	0.01399698	0.127484	0.675429964
1426434_at	TMEM43	TRANSMEMBRANE PROTEIN 43	0.0209224	0.01018636	0.7100251	0.850415287
1432739_at	2900060K15RIK	RIKEN CDNA 2900060K15 GENE	0.02096416	0.3350781	0.7010548	3.493105145
1419826_at	KIF17	KINESIN FAMILY MEMBER 17	0.02098386	0.01244456	0.2274126	1.405968699
1447843_at	PSMD13	PROTEASOME (PROSOME, MACROPAIN) 26S SUBUNIT, NON-ATPASE, 13	0.02108942	0.5332898	0.08360405	1.586277036
1446857_at	C87115	EXPRESSED SEQUENCE C87115	0.02110178	0.1397373	0.1417522	0.496519436
1451245_at	LRRC3B	LEUCINE RICH REPEAT CONTAINING 3B	0.02113126	0.4399442	0.8518744	1.315835125
1429360_at	Klf3	Kruppel-like factor 3 (basic)	0.02115098	0.2028335	0.5534391	0.748780733
1431836_x_at	5430432N15RIK	RIKEN CDNA 5430432N15 GENE	0.02119483	0.8629143	0.472294	0.530970866
1460402_at	BRPF1	BROMODOMAIN AND PHD FINGER CONTAINING, 1	0.0213065	0.00059732	0.3972823	0.77418435
1417951_at	ENO3	ENOLASE 3, BETA MUSCLE	0.02130963	0.4118724	0.6259774	0.76178184
1444905_at	D1ERTD705E	DNA SEGMENT, CHR 1, ERATO DOI 705, EXPRESSED	0.02133869	0.5556588	0.2140883	2.344907377
1448716_at	HBA-X	HEMOGLOBIN X, ALPHA-LIKE EMBRYONIC CHAIN IN HBA COMPLEX	0.02134989	0.2601254	0.4097329	0.526213133
1418666_at	PTX3	PENTRAXIN RELATED GENE	0.0213611	0.1026114	0.4715918	0.520640668
1428676_at	TMPRSS6	TRANSMEMBRANE SERINE PROTEASE 6	0.02136286	0.8251003	0.7759768	1.230766456
1435356_at	FBXO42	F-BOX PROTEIN 42	0.02151628	0.01467403	0.1944067	1.133320079
1415973_at	---	0 day neonate cerebellum cDNA, RIKEN full-length enriched library, clone:C230070N19 product:myristoylated alanine rich protein kinase C substrate, full insert sequence	0.02154823	0.005440231	0.4133721	0.753929231
1438228_at	4930452B06RIK	RIKEN CDNA 4930452B06 GENE	0.02159884	0.02938599	0.4535023	0.845754448
1436742_a_at	GM1967	GENE MODEL 1967, (NCBI)	0.0216103	0.4938059	0.7863217	2.791404849
1452097_a_at	Dusp7 /// LOC674944	dual specificity phosphatase 7 /// similar to dual specificity phosphatase 7	0.02165217	0.1650341	0.5371656	1.481791962
1441426_at	BC002230	CDNA SEQUENCE BC002230	0.02165994	0.5398091	0.3796396	1.922719673
1441839_s_at	BC024814	CDNA SEQUENCE BC024814	0.02166097	0.2098218	0.5151191	0.807703376
1417223_at	CD2BP2	CD2 ANTIGEN (CYTOPLASMIC TAIL) BINDING PROTEIN 2	0.02171317	0.02231212	0.4570655	0.799489461
1430044_at	4930415F15RIK	RIKEN CDNA 4930415F15 GENE	0.02172284	0.939443	0.3909228	1.64155835
1439446_at	BC048507	CDNA SEQUENCE BC048507	0.02172969	0.1733467	0.9994906	0.461103105
1445739_at	9430080K19Rik	RIKEN cDNA 9430080K19 gene	0.02174523	0.7617593	0.3322443	3.274197605
1439946_at	MEF2C	MYOCYTE ENHANCER FACTOR 2C	0.02174595	0.04932432	0.6685211	1.341689385
1456023_at	2210010B09RIK	RIKEN CDNA 2210010B09 GENE	0.02176545	0.3232595	0.7724973	0.851927245
1445487_at	RIF1	RAP1 INTERACTING FACTOR 1 HOMOLOG (YEAST)	0.02176984	0.5396632	0.4071706	0.474468116
1433280_at	4933417G07RIK	RIKEN CDNA 4933417G07 GENE	0.02179846	0.3816558	0.5165784	1.990345913
1418544_at	KCNIP3	KV CHANNEL INTERACTING PROTEIN 3, CALSENILIN	0.02181072	0.8159813	0.849039	1.26233675
1445211_at	SCMH1	SEX COMB ON MIDLEG HOMOLOG 1	0.02188603	0.003001533	0.29718489	0.816241898
1440814_x_at	Hs3st2	heparan sulfate (glucosamine) 3-O-sulfotransferase 2	0.02196219	0.01309992	0.8523566	1.400492457
1441820_at	CDKL2	CYCLIN-DEPENDENT KINASE-LIKE 2 (CDC2-RELATED KINASE)	0.02196381	0.8685975	0.2350901	2.375592227
1418260_at	HUNK	HORMONALLY UPREGULATED NEU-ASSOCIATED KINASE	0.02203338	0.03677683	0.847552	1.184133518
1437920_at	EPHA5	ECK-LIKE SEQUENCE 1	0.02207153	4.96924E-06	0.9262599	0.664630711
1429196_at	Rabgap1l	RAB GTPase activating protein 1-like	0.02208884	0.1458023	0.4229076	1.312341604
1422253_at	COL10A1	PROCOLLAGEN, TYPE X, ALPHA 1	0.02212269	0.9073112	0.2249609	0.545178667
1427784_at	DEFB13	DEFENSIN BETA 13	0.0221306	0.2158424	0.1029738	1.903133331
1440806_x_at	5730526G10RIK	RIKEN CDNA 5730526G10 GENE	0.02213769	0.2049949	0.9028226	1.62481887
1427942_at	PAMCI	PEPTIDYLGLYCINE ALPHA-AMIDATING MONOOXYGENASE COOH-TERMINAL INTERACTOR	0.0221485	0.09459372	0.8245726	1.898361434
1426478_at	Rasa1	RAS p21 protein activator 1	0.02214965	2.91055E-05	0.08752157	0.849651902
1423436_at	GSTA3	GLUTATHIONE S-TRANSFERASE, ALPHA 3	0.02219461	0.05737318	0.2813034	1.859299837
1440091_at	Mrg1	myeloid ecotropic viral integration site-related gene 1	0.02221363	0.000202575	0.31059379	0.614626334

1438827_at	Gls	Glutaminase	0.02224072	0.00317981	0.1206682	1.718841197
1455878_at	2700023E23RIK	RIKEN CDNA 2700023E23 GENE	0.02225336	0.03165353	0.9675577	0.780501759
1456854_at	NEURL	NEURALIZED-LIKE HOMOLOG (DROSOPHILA)	0.02226148	0.003980975	0.8565982	1.186392255
1426222_s_at	LOH11CR2A	LOSS OF HETEROZYGOSITY, 11, CHROMOSOMAL REGION 2, GENE A HOMOLOG (HUMAN)	0.02229385	0.3297865	0.05835083	1.265549245
1432582_at	3110054G05RIK	RIKEN CDNA 3110054G05 GENE	0.02230542	0.3296648	0.6725586	2.422233989
1449502_at	DAZL	DELETED IN AZOOSPERMIA-LIKE	0.02232827	0.06659827	0.3950868	0.751643532
1444884_at	PPT1	PALMITOYL-PROTEIN THIOESTERASE 1	0.0223702	0.9895457	0.1099257	0.811387333
1430689_at	2810019C22RIK	RIKEN CDNA 2810019C22 GENE	0.02244083	0.1491304	0.3862548	0.750153233
1456236_s_at	COMMD10	COMM DOMAIN CONTAINING 10	0.02250133	0.7622555	0.3820854	0.77444923
1448898_at	CCL9	CHEMOKINE (C-C MOTIF) LIGAND 9	0.02252203	0.07890362	0.6751509	0.71731557
1418873_at	SFXN4	SIDEROFLEXIN 4	0.02258554	0.8633011	0.3322647	0.785722263
1442689_at	D9ERTD278E	DNA SEGMENT, CHR 9, ERATO DOI 278, EXPRESSED	0.02258719	0.0853179	0.05876624	0.511241076
1458982_at	AU014965	EXPRESSED SEQUENCE AU014965	0.02259817	0.9776944	0.08498601	0.542088898
1425597_a_at	QK	QUAKING	0.0226189	0.01180777	0.8454564	1.118166861
1452268_at	2810485I05RIK	RIKEN CDNA 2810485I05 GENE	0.02262067	0.1436219	0.7603335	0.900485068
1441543_at	EYA3	EYES ABSENT 3 HOMOLOG (DROSOPHILA)	0.02264461	0.4439303	0.8490011	1.942686566
1427776_a_at	FGFR4	FIBROBLAST GROWTH FACTOR RECEPTOR 4	0.02264922	0.06598676	0.1813978	1.955778931
1444815_at	C85328	EXPRESSED SEQUENCE C85328	0.02265485	0.3476841	0.1625733	0.682665426
1440055_at	AI848100	EXPRESSED SEQUENCE AI848100	0.02267211	0.01142127	0.6053169	0.668395679
1436915_x_at	LAPTM4B	LYSOSOMAL-ASSOCIATED PROTEIN TRANSMEMBRANE 4B	0.02270925	0.001656232	0.3413824	0.779092335
1445964_at	---	---	0.02272361	0.8711425	0.5714452	1.671926038
1432052_at	EXOSC1	EXOSOME COMPONENT 1	0.02273092	0.9067858	0.7353354	0.841955972
1444215_at	---	---	0.02273233	0.6312114	0.480561	0.789421447
1452933_at	ANKRD39	RIKEN CDNA 9130416N05 GENE	0.02273587	0.08576618	0.0981641	0.842283675
1420280_x_at	COL9A3	PROCOLLAGEN, TYPE IX, ALPHA 3	0.0227532	0.2699106	0.1014037	1.417107078
1442921_at	MMP24	MATRIX METALLOPEPTIDASE 24	0.02279076	0.8426051	0.5603101	2.260946461
1416204_at	Gpd1	glycerol-3-phosphate dehydrogenase 1 (soluble)	0.02284271	0.928845	0.7713494	1.432787899
1452435_at	Wdr22	WD repeat domain 22	0.02289059	0.1994407	0.2396613	1.142869322
1430190_at	1700041C02RIK	RIKEN CDNA 1700041C02 GENE	0.02289126	0.9377018	0.1116886	0.81314459
1456097_a_at	Itgb3bp	integrin beta 3 binding protein (beta3-endonexin)	0.02290231	0.1230741	0.7278184	0.723657615
1442506_at	DNAJA1	DNAJ (HSP40) HOMOLOG, SUBFAMILY A, MEMBER 1	0.02294261	0.4869438	0.1193777	1.268385374
1453931_at	COL14A1	PROCOLLAGEN, TYPE XIV, ALPHA 1	0.02294678	0.165166	0.07324576	0.665034626
1424229_at	DYRK3	DUAL-SPECIFICITY TYROSINE-(Y)-PHOSPHORYLATION REGULATED KINASE 3	0.02296432	0.005660315	0.4291123	1.139343665
1424967_x_at	Tnnt2	troponin T2, cardiac	0.02301511	0.7112935	0.6209209	0.678705724
1457837_at	BC033606	PHOSPHOLIPASE B1	0.02302541	0.7049463	0.2050733	0.670317801
1429447_at	EVC2	ELLIS VAN CREVELD SYNDROME 2 HOMOLOG (HUMAN)	0.02303518	0.3169578	0.812916	0.808607171
1421176_at	Rasgrp1	RAS guanyl releasing protein 1	0.02305064	1.41932E-05	0.7278676	1.142789518
1427043_s_at	COVA1	CYTOSOLIC OVARIAN CARCINOMA ANTIGEN 1	0.02309362	0.3625534	0.8940571	0.719862715
1416396_at	SNX4	SORTING NEXIN 4	0.02310423	0.006712584	0.6314745	0.859001561
1454784_at	Hs3st2	heparan sulfate (glucosamine) 3-O-sulfotransferase 2	0.02314865	0.07880847	0.4486643	1.481660882
1418734_at	BE136769	EXPRESSED SEQUENCE BE136769	0.02318554	0.56288	0.09138047	0.436083799
1448573_a_at	CEACAM10	CEA-RELATED CELL ADHESION MOLECULE 10	0.02318591	0.3673879	0.9486636	2.425389811
1442945_at	2010109K09RIK	RIKEN CDNA 2010109K09 GENE	0.02324221	0.2779763	0.559031	1.391495055
1442243_at	Per3	period homolog 3 (Drosophila)	0.02326382	0.6208494	0.1382249	1.198025389
1440126_at	BC037704	CDNA SEQUENCE BC037704	0.02327787	0.5090958	0.09054625	0.661782977
1445200_at	---	---	0.02328227	0.1045221	0.6922556	2.025429157
1451475_at	PLXND1	PLEXIN D1	0.02329585	0.1585437	0.2900865	1.244441231
1437604_x_at	Apcdd1	adenomatosis polyposis coli down-regulated 1	0.02333107	0.07497052	0.3945335	0.735092393
1425421_at	RBBP6	RETINOBLASTOMA BINDING PROTEIN 6	0.02335376	0.08920496	0.1657922	0.771205566
1416527_at	RAB32	RAB32, MEMBER RAS ONCOGENE FAMILY	0.02336555	0.1763344	0.641259	0.6793604
1429875_at	1700034J05RIK	RIKEN CDNA 1700034J05 GENE	0.02338722	0.98607	0.1988083	0.54213452
1422153_a_at	ASB11	ANKYRIN REPEAT AND SOCS BOX-CONTAINING PROTEIN 11	0.02340471	0.4808852	0.6481941	0.78193266
1445465_at	N28178	expressed sequence N28178	0.02341082	0.2410908	0.2442978	1.171483737
1457056_at	FTCD	FORMIMINOTRANSFERASE CYCLODEAMINASE	0.02341784	0.5466062	0.2199728	2.276622733
1421818_at	BCL6	B-CELL LEUKEMIA/LYMPHOMA 6	0.02343676	0.9901726	0.55034229	0.706073262
1429629_at	EZH1	ENHANCER OF ZESTE HOMOLOG 1 (DROSOPHILA)	0.0234422	0.2244025	0.3619771	1.163876272
1460133_at	---	---	0.02351299	0.6729738	0.0930299	1.596988241
1434686_at	EXOSC8	EXOSOME COMPONENT 8	0.02353798	0.4787116	0.1848712	0.831191658
1424248_at	Arpp21	cyclic AMP-regulated phosphoprotein, 21	0.02353897	0.4991034	0.4254575	1.255168409
1430572_at	1810042K04RIK	RIKEN CDNA 1810042K04 GENE	0.02359328	0.4190923	0.07468653	1.63431729
1452783_at	Fndc3b	fibronectin type III domain containing 3B	0.02359779	0.0114248	0.331534	1.173866229
1453053_at	2610036L11RIK	RIKEN CDNA 2610036L11 GENE	0.02360834	0.8322507	0.4549141	0.718268118
1450957_a_at	SQSTM1	SEQUESTOSOME 1	0.0236249	0.7320797	0.5915045	1.060898658
1436206_at	FBXO10	F-BOX PROTEIN 10	0.02365614	0.1180741	0.966805	0.756036633
1445813_at	WDR27	WD REPEAT DOMAIN 27	0.02372984	0.65329669	0.1446604	1.507156972
1431001_at	9030624J02RIK	RIKEN CDNA 2610029J22 GENE	0.02373336	0.8648729	0.3591209	2.201563634
1432299_at	4921513I08RIK	RIKEN CDNA 4921513I08 GENE	0.02381809	0.219229	0.0615947	0.534620421
1433357_at	5730422E09RIK	RIKEN CDNA 5730422E09 GENE	0.02382327	0.5558469	0.7903887	2.355368242
1416914_s_at	MTVR2	MAMMARY TUMOR VIRUS RECEPTOR 2	0.02382631	0.01064205	0.5397272	1.177505967
1441300_at	KCNF1	POTASSIUM VOLTAGE-GATED CHANNEL, SUBFAMILY F, MEMBER 1	0.02383862	0.3071964	0.4016422	1.544228091
1417235_at	EHD3	EH-DOMAIN CONTAINING 3	0.02386344	0.5493169	0.9330312	0.799857074
1436537_at	ZFP629	ZINC FINGER PROTEIN 629	0.02387462	0.1613357	0.5871858	0.820209987
1446994_at	EXOSC2	EXOSOME COMPONENT 2	0.0238755	0.7279254	0.7148826	2.429454811
1454770_at	CCKBR	CHOLECYSTOKININ B RECEPTOR	0.02390417	0.6313713	0.2798984	1.194263564
1438565_at	A830010M20RIK	EXPRESSED SEQUENCE AI849226	0.02391924	0.00032252	0.7041547	1.12270459
1425833_a_at	HPCA	HIPPOCALCIN	0.02392542	0.000673859	0.5919145	1.134636521
1435858_at	---	---	0.02399025	0.2820789	0.8356082	0.66707706
1460233_at	GUCA2B	GUANYLATE CYCLASE ACTIVATOR 2B (RETINA)	0.02400455	0.1446655	0.9012465	0.586046954
1450768_at	DLGH1	DISCS, LARGE HOMOLOG 1 (DROSOPHILA)	0.02405399	0.5611963	0.7193547	1.203531785
1418230_a_at	LIMS1	RIKEN CDNA 4921524A02 GENE	0.0241135	0.003092212	0.065727629	1.086829223
1441848_at	---	---	0.02417313	0.2408342	0.9058587	1.351796596
1435772_at	KIF21B	KINESIN FAMILY MEMBER 21B	0.02421936	0.337274	0.9689241	1.278509154
1422258_at	CHRM3	CHOLINERGIC RECEPTOR, MUSCARINIC 3, CARDIAC	0.02423418	0.01607961	0.4716187	0.753638429
1444082_at	A730017C20Rik	RIKEN cDNA A730017C20 gene	0.02424407	0.7668341	0.3192814	0.761464677
1441397_at	PARD3	DNA SEGMENT, CHR 8, ERATO DOI 580, EXPRESSED	0.02424967	0.193142	0.3312718	0.736180483
1445805_x_at	KCNH3	POTASSIUM VOLTAGE-GATED CHANNEL, SUBFAMILY H (EAG-RELATED), MEMBER 3	0.02435232	0.8592085	0.861523	1.384713604
1458183_at	---	---	0.02438799	0.7788349	0.5642486	0.833808572
1457054_a_at	D130020L05RIK	RIKEN CDNA D130020L05 GENE	0.02440989	0.1426742	0.602013	0.782200674
1424220_a_at	PORCN	PORCUPINE HOMOLOG (DROSOPHILA)	0.02442256	0.06705508	0.99159	0.767286401
1426850_a_at	MAP2K6	MITOGEN ACTIVATED PROTEIN KINASE KINASE 6	0.02444035	0.002997161	0.8983544	0.808854473
1446637_at	PRDM10	PR DOMAIN CONTAINING 10	0.02446423	0.5792204	0.9457037	2.708457025
1431469_a_at	CXXC5	CXXC FINGER 5	0.02446625	0.133422	0.08816611	1.151829092
1442829_at	RIN2	RAS AND RAB INTERACTOR 2	0.0244664	0.04753179	0.1024735	1.998341159
1420461_at	MST1R	MACROPHAGE STIMULATING 1 RECEPTOR (C-MET-RELATED TYROSINE KINASE)	0.02447079	0.4533267	0.5421922	1.860201196
1421922_at	SH3BP5	SH3-DOMAIN BINDING PROTEIN 5 (BTK-ASSOCIATED)	0.02447502	0.001534888	0.637853	0.792106174
1453401_at	1700007G11RIK	RIKEN CDNA 1700007G11 GENE	0.02451622	0.5520744	0.4741006	2.6889956
1422737_at	NCOA3	NUCLEAR RECEPTOR COACTIVATOR 3	0.02452468	0.01947548	0.1761461	1.124144845
1445740_at	MASS1	MONOGENIC, AUDIOGENIC SEIZURE SUSCEPTIBILITY 1	0.02453528	0.002377364	0.2976891	1.574984611
1454055_at	1600029O15RIK	RIKEN CDNA 1600029O15 GENE	0.02457396	0.01476989	0.09592414	0.874084349
1437151_at	USP22	UBIQUITIN SPECIFIC PEPTIDASE 22	0.02457568	6.41016E-05	0.1864809	0.814556314
1429767_at	2810013P06RIK	RIKEN CDNA 2810013P06 GENE	0.02457877	0.6618835	0.7052198	0.801713213
1449913_at	ZFP2	ZINC FINGER PROTEIN 2	0.02465438	0.001092029	0.4099546	0.807504574
1451059_at	4933409D10RIK	RIKEN CDNA 4933409D10 GENE	0.02466145	0.5158011	0.955785	2.090923813
1456900_at	---	---	0.02467319	0.06392541	0.2176024	1.180303341
1428184_at	3110035E14RIK	RIKEN CDNA 3110035E14 GENE	0.02470465	0.06934632	0.8817956	1.231230944
1438543_at	Spata13	Spermatogenesis associated 13	0.02474368	0.139331	0.6373263	1.531262063
1418084_at	Nrp1	neuropilin 1	0.02481817	0.000735869	0.7319888	0.772023405
1450253_a_at	Map3k4	mitogen activated protein kinase kinase kinase 4	0.02481878	0.001895963	0.7089987	0.805379252
1445398_at	3732412D22RIK	RIKEN CDNA 3732412D22 GENE	0.02485832	0.02762409	0.1848649	0.653655619
1429041_at	LOC436177	SIMILAR TO CADHERIN-11 PRECURSOR (OSTEOBLAST-CADHERIN) (OB-CADHERIN) (OSF-4)	0.02486246	0.2645303	0.2102306	0.854662434
1443818_at	---	---	0.02489127	0.3533459	0.6809316	2.301971957
1459585_at	GAS6	GROWTH ARREST SPECIFIC 6	0.02490645	0.4400516	0.3514592	1.338554485
1444574_at	ELF2	E74-LIKE FACTOR 2	0.02491533	0.5897801	0.3448735	1.780175942
1453897_at	C030014A21RIK	RIKEN CDNA C030014A21 GENE	0.02491994	0.3017633	0.6846808	1.363213053
1448659_at	CASP7	CASPASE 7	0.02492759	0.05225748	0.5269669	0.857738753
1425518_at	Rapgef4	Rap guanine nucleotide exchange factor (GEF) 4	0.02493772	0.870145	0.55420309	0.733727611
1417110_at	Man1a	mannosidase 1, alpha	0.02496633	0.006529318	0.2534407	1.264886818
1422981_at	DGCR8	DIGEORGE SYNDROME CRITICAL REGION GENE 8	0.02497759	0.002237903	0.09774893	1.117807138
1416218_x_at	RPL37A	RIBOSOMAL PROTEIN L37A	0.02506277	3.14928E-05	0.4796219	1.436790463
1447667_x_at	MAP3K4	MITOGEN ACTIVATED PROTEIN KINASE KINASE KINASE 4	0.0251477	0.07613824	0.9194149	0.807838389
1422798_at	Cntnap2	contactin associated protein-like 2	0.02516105	0.2484256	0.62343079	0.822419843
1428156_at	GNG2	GUANINE NUCLEOTIDE BINDING PROTEIN (G PROTEIN), GAMMA 2 SUBUNIT	0.0251673	0.06051	0.4329508	0.801533117
1448250_at	9030425E11RIK	RIKEN CDNA 9030425E11 GENE	0.02517934	0.04212089	0.3584482	0.817083103
1456130_at	LOC553091	HYPOTHETICAL LOC553091	0.02518966	0.996301	0.4708458	0.560731041
1417787_at	DKKL1	DICKKOPF-LIKE 1	0.02522624	0.801554	0.1035084	1.680580106
1435131_at	ZFP13	ZINC FINGER PROTEIN 13	0.02531871	0.9432745	0.8620724	0.779139476
1433920_at	SEMA4C	SEMA DOMAIN, IMMUNOGLOBULIN DOMAIN (IG), TRANSMEMBRANE DOMAIN (TM) AND SHORT CYTOPLASMIC DOMAIN, (SEMAPHORIN) 4C	0.02533171	0.425669	0.09168133	1.134526542
1441784_at	---	Adult male hypothalamus cDNA, RIKEN full-length enriched library, clone:A230076B10 product:unclassifiable, full insert sequence	0.02533895	0.6265318	0.063643679	0.797103466
1450261_a_at	SLC10A1	SOLUTE CARRIER FAMILY 10 (SODIUM/BILE ACID COTRANSPORTER FAMILY), MEMBER 1	0.02534219	0.590564	0.1791935	0.617950283
1456217_at	D7WSU128E	DNA SEGMENT, CHR 7, WAYNE STATE UNIVERSITY 128, EXPRESSED	0.02536037	0.2139597	0.1638026	0.83499568
1429158_at	Fbxo28	F-box protein 28	0.02540791	0.001248736	0.1196808	1.106521694
1434762_at	A730041O15RIK	RIKEN CDNA A730041O15 GENE	0.0254487	3.27447E-05	0.847681	0.695966738
1436050_x_at	HES6	HAIRY AND ENHANCER OF SPLIT 6 (DROSOPHILA)	0.02545203	0.006047233	0.8497422	0.750865532
1447984_at	D1ERTD75E	DNA SEGMENT, CHR 1, ERATO DOI 75, EXPRESSED	0.02547148	0.0139196	0.819043	1.289381496
1420334_at	SLC12A8	SOLUTE CARRIER FAMILY 12 (POTASSIUM/CHLORIDE TRANSPORTERS), MEMBER 8	0.02550228	0.1001047	0.9620983	1.686059706
1428793_at	SLC36A1	SOLUTE CARRIER FAMILY 36 (PROTON/AMINO ACID SYMPORTER), MEMBER 1	0.0255654	0.6656188	0.731403	1.36069265
1443995_at	GM9	GENE MODEL 9, (NCBI)	0.02560336	0.01621312	0.07252547	0.829368339
1440206_at	A930024E05RIK	RIKEN CDNA A930024E05 GENE	0.02561593	0.967321	0.6686264	0.836087114
1443465_at	CSPG6	CHONDROITIN SULFATE PROTEOGLYCAN 6	0.02563556	0.8876933	0.1028061	0.602862973
1424927_at	GLIPR1	GLI PATHOGENESIS-RELATED 1 (GLIOMA)	0.02564037	0.1559225	0.3144685	1.210488925
1459462_at	GIG1	GLUCOCORTICOID INDUCED GENE 1	0.0256462	0.06643996	0.4085401	1.602605703
1441518_at	---	---	0.02571057	0.2592212	0.840029	2.650318196
1429656_at	RHOBTB1	RHO-RELATED BTB DOMAIN CONTAINING 1	0.02573342	0.00443759	0.8506283	1.848257222
1434222_at	SIPA1L1	SIGNAL-INDUCED PROLIFERATION-ASSOCIATED 1 LIKE 1	0.02575044	0.1533478	0.6716475	1.215546473
1459051_at	6530418L21Rik	RIKEN cDNA 6530418L21 gene	0.02575631	0.1457714	0.5244003	1.203305918
1437412_at	PRDX6-RS1	PEROXIREDOXIN 6, RELATED SEQUENCE 1	0.02576493	0.9255614	0.06336195	0.645889719
1418744_s_at	Tesc	tescalcin	0.0257855	0.3799611	0.3848783	1.349449423
1459186_at	C80165	EXPRESSED SEQUENCE C80165	0.0258525	0.2073246	0.4155528	4.1082505
1434207_at	2900057K09RIK	RIKEN CDNA 2900057K09 GENE	0.02588014	0.001444767	0.5825005	0.912078405
1431450_at	4930444P10RIK	RIKEN CDNA 4930444P10 GENE	0.02588165	0.1618924	0.1236833	1.626757111
1426562_a_at	OLFM1	OLFACTOMEDIN 1	0.02591587	0.02482773	0.7766991	0.780008636
1419447_s_at	TBC1D1	TBC1 DOMAIN FAMILY, MEMBER 1	0.02600068	0.00602532	0.2043659	1.111433182
1457159_at	GM101	GENE MODEL 101, (NCBI)	0.02601238	0.9580485	0.4822749	1.773723309
1435776_at	---	---	0.02607826	0.03580416	0.1760776	0.732592774
1425362_at	HRBL	HIV-1 REV BINDING PROTEIN-LIKE	0.02608202	0.01242084	0.727096	0.752070578
1433553_at	GARNL3	GTPASE ACTIVATING RANGAP DOMAIN-LIKE 3	0.02619448	0.8042097	0.2542839	1.246746223
1415700_a_at	SSR3	SIGNAL SEQUENCE RECEPTOR, GAMMA	0.02620547	0.6572438	0.4197166	0.86277239
1431057_a_at	Prss23	protease, serine, 23	0.02626031	0.3036504	0.4093871	1.319681688
1439333_at	Kcnv1	potassium channel, subfamily V, member 1	0.02626368	0.5355691	0.9383525	1.267291905

1438331_at	YPEL2	YIPPEE-LIKE 2 (DROSOPHILA)	0.02628811	0.03798494	0.1327741	1.183492433
1437599_at	CHCHD3	COILED-COIL-HELIX-COILED-COIL-HELIX DOMAIN CONTAINING 3	0.02629558	0.001342178	0.3365449	0.757781512
1436549_a_at	HNRPA1	HETEROGENEOUS NUCLEAR RIBONUCLEOPROTEIN A1	0.02631016	0.8989618	0.3248372	0.69806861
1459813_at	1700012D01RIK	RIKEN CDNA 1700012D01 GENE	0.02631379	0.150065	0.3800639	1.247002777
1446932_at	---	---	0.02634936	0.5556424	0.05643723	1.195576485
1443897_at	DDIT3	DNA-DAMAGE INDUCIBLE TRANSCRIPT 3	0.02636237	0.1603979	0.6693483	0.52840339
1458373_at	5830483C08Rik	RIKEN cDNA 5830483C08 gene	0.02636491	0.1281019	0.30403989	0.457723659
1443999_at	NEK2	NIMA (NEVER IN MITOSIS GENE A)-RELATED EXPRESSED KINASE 2	0.02638952	0.01649814	0.8681564	0.670888527
1440179_x_at	Ibrdc1	IBR domain containing 1	0.02644831	0.9487208	0.6475925	1.352995095
1437480_at	1110001A07RIK	RIKEN CDNA 1110001A07 GENE	0.02645965	0.2465091	0.3271314	0.799294459
1435323_a_at	OACT1	O-ACYLTRANSFERASE (MEMBRANE BOUND) DOMAIN CONTAINING 1	0.02646164	0.478784	0.2296797	0.769181508
1432584_at	1110004M10RIK	RIKEN CDNA 1110004M10 GENE	0.0264631	0.2768491	0.08816042	0.515647322
1430033_at	5330431K02RIK	RIKEN CDNA 5330431K02 GENE	0.02648686	1.07839E-05	0.1061905	0.783733253
1435511_at	SYN2	SYNAPSIN II	0.0264923	0.2920195	0.4328866	0.823771104
1435350_at	TRAF6	TNF RECEPTOR-ASSOCIATED FACTOR 6	0.02650297	0.8255118	0.5561598	0.787024572
1440581_at	A530088H08Rik	RIKEN cDNA A530088H08 gene	0.02651028	0.3271257	0.08701163	0.69535337
1421252_a_at	Mef2a	myocyte enhancer factor 2A	0.02654181	0.2507204	0.09999622	0.800817745
1429119_at	4833421E05RIK	RIKEN CDNA 4833421E05 GENE	0.026551	0.1116855	0.2123247	1.138108485
1429122_a_at	1700040I03RIK	RIKEN CDNA 1700040I03 GENE	0.02660763	0.07665851	0.4646549	1.232922246
1446380_at	9430076C15RIK	RIKEN CDNA 9430076C15 GENE	0.02666891	0.3265822	0.05184005	0.589651611
1459141_at	1810008I18RIK	RIKEN CDNA 1810008I18 GENE	0.02670001	0.8160038	0.2967422	0.560718061
1453807_at	6330563C09RIK	RIKEN CDNA 6330563C09 GENE	0.02670329	0.03733042	0.3236027	1.173228855
1447941_x_at	BRAF	BRAF TRANSFORMING GENE	0.02671369	0.1445554	0.07601548	1.13357953
1424382_at	RCN3	RETICULOCALBIN 3, EF-HAND CALCIUM BINDING DOMAIN	0.02671451	0.00874497	0.4992055	0.730246506
1432223_at	4930552N02RIK	RIKEN CDNA 4930552N02 GENE	0.02683633	0.6896833	0.9005694	4.062061047
1428207_at	BCL7A	B-CELL CLL/LYMPHOMA 7A	0.02688935	0.0792521	0.5448055	0.857383854
1444409_at	RPH3AL	RABPHILIN 3A-LIKE (WITHOUT C2 DOMAINS)	0.0269008	0.062368	0.7094607	0.746815514
1450323_at	5730493B19RIK	RIKEN CDNA 5730493B19 GENE	0.02692614	0.1268134	0.6026028	1.49152415
1446409_at	GTDC1	GLYCOSYLTRANSFERASE-LIKE DOMAIN CONTAINING 1	0.02699449	0.3405633	0.2010062	0.736418391
1442845_at	C130075A20RIK	RIKEN CDNA C130075A20 GENE	0.02701302	0.00959849	0.1839777	1.170686041
1445542_at	4930579C12RIK	RIKEN CDNA 4930579C12 GENE	0.02708041	0.1398133	0.3171761	1.786689584
1444904_at	CBFA2T1H	CBFA2T1 IDENTIFIED GENE HOMOLOG (HUMAN)	0.02712077	0.9899235	0.05920933	1.329236888
1456555_at	CCDC67	COILED-COIL DOMAIN CONTAINING 67	0.02715435	0.7943623	0.8394702	0.569303949
1449090_a_at	Yes1	Yamaguchi sarcoma viral (v-yes) oncogene homolog 1	0.02715445	0.3315001	0.80609	0.70532829
1452653_at	SLC25A22	SOLUTE CARRIER FAMILY 25 (MITOCHONDRIAL CARRIER, GLUTAMATE), MEMBER 22	0.02716873	0.0467822	0.773748	0.751786905
1442380_at	LCN4	LIPOCALIN 4	0.02719281	0.1702895	0.06639941	0.618119536
1420326_s_at	CRAMP1L	CRM, CRAMPED-LIKE (DROSOPHILA)	0.02720717	0.3972383	0.8211514	0.860723334
1416622_at	WBSCR16	WILLIAMS-BEUREN SYNDROME CHROMOSOME REGION 16 HOMOLOG (HUMAN)	0.02734077	0.001499114	0.05171627	0.933267211
1428141_at	GGA2	GOLGI ASSOCIATED, GAMMA ADAPTIN EAR CONTAINING, ARF BINDING PROTEIN 2	0.02734322	0.1698249	0.6322652	1.129765587
1422658_at	PLCZ1	PHOSPHOLIPASE C, ZETA 1	0.0273497	0.01275327	0.7454159	1.81617826
1439105_at	CDADC1	CYTIDINE AND DCMP DEAMINASE DOMAIN CONTAINING 1	0.02737451	0.5795671	0.05435432	0.905433405
1438169_a_at	Frmd4b	FERM domain containing 4B	0.02746414	0.708717	0.5545221	0.618028941
1424812_at	BC017158	CDNA SEQUENCE BC017158	0.02747831	0.4276189	0.3375987	0.854947558
1454972_at	ATCAY	ATAXIA, CEREBELLAR, CAYMAN TYPE HOMOLOG (HUMAN)	0.02750694	0.1355988	0.7229931	1.185790557
1449262_s_at	Lin7c	lin-7 homolog C (C. elegans)	0.02758348	1.75625E-06	0.8887706	0.718312878
1453244_at	5830416P10RIK	RIKEN CDNA 5830416P10 GENE	0.02759378	0.04186137	0.09685523	0.787619847
1419705_at	CAR5B	CARBONIC ANHYDRASE 5B, MITOCHONDRIAL	0.02760815	0.1982852	0.4066689	0.685388472
1456005_a_at	BCL2L11	BCL2-LIKE 11 (APOPTOSIS FACILITATOR)	0.02761281	0.02746144	0.06820748	1.141667268
1455038_at	---	---	0.02762566	0.9749951	0.249367	0.540573819
1460654_at	SLC30A3	SOLUTE CARRIER FAMILY 30 (ZINC TRANSPORTER), MEMBER 3	0.02768539	0.8172696	0.1681884	1.311919562
1446793_at	1110057K04RIK	RIKEN CDNA 1110057K04 GENE	0.02774118	0.9158436	0.1345822	0.806779716
1440367_at	PGS1	RIKEN CDNA 2610019F11 GENE	0.02774621	0.7751321	0.4376571	1.816278261
1431297_a_at	4933436C20RIK	RIKEN CDNA 4933436C20 GENE	0.02774891	0.2586408	0.6391515	0.66123162
1435265_at	---	---	0.02775278	0.1838527	0.16172329	1.253716632
1457946_at	OG9X	OG9 HOMEOBOX GENE	0.02776576	0.0300214	0.06332138	1.098119255
1419124_at	2210010L05RIK	RIKEN CDNA 2210010L05 GENE	0.02778191	0.05090636	0.1160882	1.15022055
1444345_at	---	---	0.02780911	0.7752592	0.4854681	1.478276449
1423407_a_at	Fbln2	fibulin 2	0.02781487	0.3801725	0.4404303	1.518374209
1435930_at	ZFP291	RIKEN CDNA D530014O03 GENE	0.02781593	0.1936661	0.6737513	0.78480609
1445359_at	ADCY1	ADENYLATE CYCLASE 1	0.02783848	0.02201534	0.8468409	1.314215302
1447174_at	DACH1	DACHSHUND 1 (DROSOPHILA)	0.02784489	0.06816481	0.6134526	0.682012217
1444164_at	PRPF39	PRP39 PRE-MRNA PROCESSING FACTOR 39 HOMOLOG (YEAST)	0.02784866	0.855758	0.5180718	0.849317244
1423427_at	ADCYAP1	ADENYLATE CYCLASE ACTIVATING POLYPEPTIDE 1	0.02789827	0.1758796	0.8435914	1.424584902
1416124_at	CCND2	CYCLIN D2	0.02797243	0.01989922	0.1570218	1.18669552
1458453_at	LMO7	LIM DOMAIN ONLY 7	0.02810492	0.1191204	0.1352267	1.156141799
1455702_at	---	---	0.02810981	0.7955148	0.1644258	1.194551697
1429168_at	BTBD4	RIKEN CDNA 4933406L05 GENE	0.02811331	0.8594686	0.718738	1.320931584
1442121_at	Arpp21	cyclic AMP-regulated phosphoprotein, 21	0.02814594	9.99896E-06	0.9411317	1.200270174
1439908_at	ZKSCAN1	ZINC FINGER WITH KRAB AND SCAN DOMAINS 1	0.0281877	0.00013647	0.06101484	1.058419604
1443595_at	CNOT6L	CCR4-NOT TRANSCRIPTION COMPLEX, SUBUNIT 6-LIKE	0.02818792	0.16492589	0.05052131	0.668272575
1427954_at	BC048403	CDNA SEQUENCE BC048403	0.02823095	0.09705938	0.8009839	0.8210838
1417262_at	PTGS2	PROSTAGLANDIN-ENDOPEROXIDE SYNTHASE 2	0.0282433	0.4324683	0.55770679	1.512779887
1419276_at	ENPP1	ECTONUCLEOTIDE PYROPHOSPHATASE/PHOSPHODIESTERASE 1	0.0282493	0.2345191	0.1613038	1.199468678
1456028_x_at	Marcks	Myristoylated alanine rich protein kinase C substrate	0.02825233	0.02988524	0.5282915	0.69177481
1421622_a_at	Rapgef4	Rap guanine nucleotide exchange factor (GEF) 4	0.0282704	7.09721E-07	0.8223147	0.759239684
1423542_at	KLK7	KALLIKREIN 7 (CHYMOTRYPTIC, STRATUM CORNEUM)	0.02827828	0.2455362	0.1620241	1.564578787
1423753_at	BAMBI	BMP AND ACTIVIN MEMBRANE-BOUND INHIBITOR, HOMOLOG (XENOPUS LAEVIS)	0.02831364	0.0306507	0.5175431	0.722606706
1433683_at	RBM35B	RNA BINDING MOTIF PROTEIN 35B	0.02833033	0.9564075	0.837179	2.639759968
1432554_at	2310040G07RIK	RIKEN CDNA 2310040G07 GENE	0.02838255	0.01392439	0.7456554	0.813509949
1418384_at	9430083G14RIK	RIKEN CDNA 9430083G14 GENE	0.02838426	0.7257844	0.951498	0.786920552
1428569_at	B230217C12RIK	RIKEN CDNA B230217C12 GENE	0.02840996	0.3984344	0.1065815	1.404881348
1437396_at	CREB3L2	CAMP RESPONSIVE ELEMENT BINDING PROTEIN 3-LIKE 2	0.02841595	0.2699736	0.1327753	1.159821771
1451008_at	ST8SIA3	ST8 ALPHA-N-ACETYL-NEURAMINIDE ALPHA-2,8-SIALYLTRANSFERASE 3	0.02841927	0.1637984	0.6910502	1.208351187
1429654_at	DPPA2	DEVELOPMENTAL PLURIPOTENCY ASSOCIATED 2	0.02842075	0.2871306	0.272118	2.206154784
1439731_at	E130309F12Rik	RIKEN cDNA E130309F12 gene	0.02846126	0.8341713	0.1515768	1.194145061
1421302_a_at	GNA15	GUANINE NUCLEOTIDE BINDING PROTEIN, ALPHA 15	0.0284628	0.1406099	0.05601144	0.853461601
1425416_s_at	PSRC1	PROLINE/SERINE-RICH COILED-COIL 1	0.02847282	0.07635108	0.8833155	1.37998518
1429606_at	4930527B16RIK	RIKEN CDNA 4930527B16 GENE	0.02850549	0.1716932	0.4499558	1.239885296
1421833_at	Pip5k1a	phosphatidylinositol-4-phosphate 5-kinase, type 1 alpha	0.02852333	0.771789	0.3320826	0.877554316
1442696_at	DHCR7	7-DEHYDROCHOLESTEROL REDUCTASE	0.02863866	0.6100848	0.05198304	0.607176487
1428195_at	4631427C17Rik	RIKEN cDNA 4631427C17 gene	0.02869157	1.90951E-05	0.6286018	0.752831068
1426005_at	DMP1	DENTIN MATRIX PROTEIN 1	0.02869637	0.477993	0.1205708	0.738295798
1450421_at	Tgfa	transforming growth factor alpha	0.02872785	0.00229842	0.4547898	0.862403664
1452474_a_at	ART3	ADP-RIBOSYLTRANSFERASE 3	0.02876239	0.8919958	0.09329204	1.240276469
1428497_at	SECISBP2	SECIS BINDING PROTEIN 2	0.0287838	0.2394483	0.8156219	1.187464224
1436630_at	---	---	0.02885559	0.08196272	0.4974395	0.863520996
1441843_s_at	5230400M03RIK	RIKEN CDNA 5230400M03 GENE	0.02886834	0.4677496	0.08228236	1.525086992
1454363_at	9430099H24RIK	RIKEN CDNA 9430099H24 GENE	0.02892956	0.4007979	0.2379663	2.304992826
1455703_at	AKT2	THYMOMA VIRAL PROTO-ONCOGENE 2	0.02893949	0.7007042	0.9561236	1.313215387
1435500_at	RAB26	RAB26, MEMBER RAS ONCOGENE FAMILY	0.02894044	0.2283889	0.7411815	0.781013873
1447865_x_at	PDZD11	PDZ DOMAIN CONTAINING 11	0.02897326	0.01787963	0.7104935	0.861136627
1450972_at	3110040N11RIK	RIKEN CDNA 3110040N11 GENE	0.028984	0.6296125	0.9523628	0.784763524
1430357_at	H3F3B	H3 HISTONE, FAMILY 3A	0.02903716	0.8066295	0.2127476	1.353207725
1437250_at	WDT2	WHN-DEPENDENT TRANSCRIPT 2	0.0290618	0.457215	0.6386535	0.75635384
1455649_at	TTC9	TETRATRICOPEPTIDE REPEAT DOMAIN 9	0.02907558	0.009746895	0.8632636	1.100873105
1459276_at	A430107D22RIK	RIKEN CDNA A430107D22 GENE	0.0290788	0.1795731	0.5266907	2.143456452
1433661_at	BC034204	CDNA SEQUENCE BC034204	0.02909783	0.5869191	0.3231817	0.742385237
1446079_at	ZMAT2	ZINC FINGER, MATRIN TYPE 2	0.02909907	0.0277438	0.5346795	0.863002634
1440112_at	Lrrtm3	leucine rich repeat transmembrane neuronal 3	0.02910926	0.000623419	0.9107908	1.261873462
1445665_at	SEPT6	SEPTIN 6	0.02913055	0.1673539	0.7333515	1.392596381
1456738_s_at	BRP16	BRAIN PROTEIN 16	0.02918594	0.6437808	0.7974236	0.868220787
1453313_at	SESN3	SESTRIN 3	0.02918841	0.01500814	0.1634931	1.164240846
1421263_at	GABRA3	GAMMA-AMINOBUTYRIC ACID (GABA-A) RECEPTOR, SUBUNIT ALPHA 3	0.02918919	0.0757953	0.1174744	1.132593572
1443097_at	EVI2A	ECOTROPIC VIRAL INTEGRATION SITE 2A	0.02920743	0.3408524	0.1306974	0.800028967
1425563_s_at	PCDH10	PROTOCADHERIN 10	0.02921385	0.833972	0.2090838	1.106572696
1445983_at	UBE2A	UBIQUITIN-CONJUGATING ENZYME E2A, RAD6 HOMOLOG (S. CEREVISIAE)	0.029217	0.9215384	0.6250216	2.343861704
1439788_at	BC065112	CDNA SEQUENCE BC065112	0.02922347	0.03730362	0.5552357	0.68369728
1455896_a_at	KCNK1	POTASSIUM CHANNEL, SUBFAMILY K, MEMBER 1	0.02923593	0.000485834	0.1598786	1.135919734
1432953_at	4921520E09RIK	RIKEN CDNA 4921520E09 GENE	0.02929995	0.808288	0.4553706	2.575343967
1454723_at	1110033M05RIK	RIKEN CDNA 1110033M05 GENE	0.0293204	0.03977151	0.8282704	0.886676311
1420068_at	HAT1	RIKEN CDNA 2410071B14 GENE	0.02932342	0.7600276	0.64835279	0.510755409
1450174_at	PTPRT	PROTEIN TYROSINE PHOSPHATASE, RECEPTOR TYPE, T	0.02932933	0.06184565	0.171288	1.224989442
1436788_at	---	---	0.02934673	6.33169E-07	0.7489424	0.731956798
1438768_at	4733401I12RIK	RIKEN CDNA 4733401I12 GENE	0.02945152	0.7729528	0.216294	0.623523691
1423938_at	LLGLH2	RIKEN CDNA 9130006H11 GENE	0.02945708	0.4074719	0.1333028	1.184735707
1445092_at	NR3C2	NUCLEAR RECEPTOR SUBFAMILY 3, GROUP C, MEMBER 2	0.02946596	0.5159311	0.6909495	1.335983362
1430122_at	9430002A10RIK	RIKEN CDNA 9430002A10 GENE	0.0295053	0.9661338	0.9638597	4.350058146
1452004_at	CALCA	CALCITONIN/CALCITONIN-RELATED POLYPEPTIDE, ALPHA	0.02951844	0.01001553	0.218719	0.70493051
1447914_x_at	4833411O04RIK	RIKEN CDNA 4833411O04 GENE	0.02955633	0.5777123	0.150566	0.774988321
1455048_at	Igsf3	immunoglobulin superfamily, member 3	0.02959754	0.08934909	0.1411778	1.127908818
1454094_at	4930471E19RIK	RIKEN CDNA 4930471E19 GENE	0.02961235	0.7207308	0.6660165	2.588003656
1417414_at	SEPT3	SEPTIN 3	0.02962364	0.002610458	0.7853455	0.780632151
1456229_at	HOXB3	HOMEO BOX B3	0.02963092	0.5854013	0.4994984	1.721674045
1440899_at	FMO5	FLAVIN CONTAINING MONOOXYGENASE 5	0.0296332	0.2151327	0.6553287	0.782091581
1436733_at	E130309F12Rik	RIKEN cDNA E130309F12 gene	0.02963675	0.000737807	0.07752514	1.078091476
1436778_at	CYBB	CYTOCHROME B-245, BETA POLYPEPTIDE	0.02977824	0.9163722	0.4639993	1.481897796
1441767_at	ZFP142	ZINC FINGER PROTEIN 142	0.02979588	0.1082049	0.227939	1.897278794
1436805_at	2810457I06RIK	RIKEN CDNA 2810457I06 GENE	0.02980316	0.005412473	0.6594638	0.87729995
1436600_at	TNRC9	TRINUCLEOTIDE REPEAT CONTAINING 9	0.02980957	0.4367119	0.3418983	0.692081532
1458783_at	B3GNT1	UDP-GLCNAC:BETAGAL BETA-1,3-N-ACETYLGLUCOSAMINYLTRANSFERASE 1	0.02983206	0.1687024	0.5231757	2.709765349
1426339_at	AK5	ADENYLATE KINASE 5	0.02983278	0.06371849	0.7460135	0.811192638
1452664_a_at	TM7SF3	TRANSMEMBRANE 7 SUPERFAMILY MEMBER 3	0.02984059	0.5954655	0.2524325	0.766134861
1456263_at	NOTCH4	NOTCH GENE HOMOLOG 4 (DROSOPHILA)	0.02985065	0.2139072	0.8678956	1.689586338
1422582_at	LEP	LEPTIN	0.02987132	0.0847373	0.2593193	0.436527852
1417155_at	MYCN	V-MYC MYELOCYTOMATOSIS VIRAL RELATED ONCOGENE, NEUROBLASTOMA DERIVED (AVIAN)	0.02991045	0.9975752	0.6839422	0.841732049
1434594_at	DNAHC1	DYNEIN, AXONEMAL, HEAVY CHAIN 1	0.02993737	0.9874913	0.7064981	0.828825673
1439351_at	A930031D07RIK	RIKEN CDNA A930031D07 GENE	0.02996743	0.3961262	0.602562	1.390279864
1441313_x_at	LHX9	LIM HOMEOBOX PROTEIN 9	0.03001054	0.002681911	0.2345309	0.721139644
1433579_at	TMEM30B	TRANSMEMBRANE PROTEIN 30B	0.03002807	0.9637886	0.3491474	0.54328286
1456765_at	6430511F03	HYPOTHETICAL PROTEIN 6430511F03	0.03003246	0.0885444	0.1534344	0.879506488
1435810_at	5730455O13RIK	RIKEN CDNA 5730455O13 GENE	0.03005324	0.498892	0.5851711	0.735636256
1434196_at	Dnaja4	DnaJ (Hsp40) homolog, subfamily A, member 4	0.03008323	0.1646058	0.8706015	1.189115923
1457713_at	ERCC5	EXCISION REPAIR CROSS-COMPLEMENTING RODENT REPAIR DEFICIENCY, COMPLEMENTATION GROUP 5	0.03015912	0.8172713	0.2082626	0.684963549
1438123_at	---	---	0.03023227	0.4165434	0.4772771	0.646668303
1439531_at	E130311K13RIK	RIKEN CDNA E130311K13 GENE	0.03025043	0.01283813	0.4379865	0.780575
1425483_at	TOX	THYMOCYTE SELECTION-ASSOCIATED HMG BOX GENE	0.03027072	0.04167484	0.2217979	1.157275528
1423277_at	Ptprk	protein tyrosine phosphatase, receptor type, K	0.03027518	0.02082117	0.06742979	1.061625528
1427767_a_at	CFTR	CYSTIC FIBROSIS TRANSMEMBRANE CONDUCTANCE REGULATOR HOMOLOG	0.03028399	0.8036517	0.6922023	2.798029197
1436767_at	LUC7L2	LUC7-LIKE 2 (S. CEREVISIAE)	0.03030214	0.004444901	0.9236456	0.769916831

1434442_at	D5ERTD593E	DNA SEGMENT, CHR 5, ERATO DOI 593, EXPRESSED	0.03037271	0.5724891	0.4689454	1.271882412
1428204_at	C030002O17RIK	RIKEN CDNA C030002O17 GENE	0.03044422	0.06812207	0.5032322	1.088743694
1453324_at	6330509M23RIK	RIKEN CDNA 6330509M23 GENE	0.03052875	0.6913863	0.57959	0.821496753
1456838_at	BC072620	CDNA SEQUENCE BC072620	0.0305907	0.07785224	0.9823758	1.210982802
1459532_at	GRM7	GLUTAMATE RECEPTOR, METABOTROPIC 7	0.03063126	0.01696841	0.985879	1.309291817
1419081_at	ATG10	AUTOPHAGY-RELATED 10 (YEAST)	0.0306523	0.6964258	0.7063649	0.710006738
1448650_a_at	POLE	POLYMERASE (DNA DIRECTED), EPSILON	0.03071251	0.04128191	0.1922769	0.510904594
1453840_at	PABPC1	POLY A BINDING PROTEIN, CYTOPLASMIC 1	0.03072717	0.1110428	0.1587829	0.814145012
1457241_at	LRRC48	LEUCINE RICH REPEAT CONTAINING 48	0.03075167	0.6219162	0.3287781	0.549266646
1417699_at	GTF2F1	RIKEN CDNA 2810405L04 GENE	0.03079584	6.31051E-07	0.08505538	1.016737227
1422168_a_at	Bdnf	brain derived neurotrophic factor	0.03088195	0.1390153	0.8932214	1.328692047
1456473_x_at	ARF2	ADP-RIBOSYLATION FACTOR 2	0.03088739	0.4220078	0.05573263	1.131840024
1423904_a_at	PVR	POLIOVIRUS RECEPTOR	0.03092701	0.8304499	0.8953158	0.823571328
1419929_at	D15ERTD55E	DNA SEGMENT, CHR 15, ERATO DOI 55, EXPRESSED	0.03095976	0.7213788	0.2552854	2.128851988
1438717_a_at	OSBPL6	OXYSTEROL BINDING PROTEIN-LIKE 6	0.0309773	0.08438845	0.1302146	1.217176166
1432839_at	6430598H11RIK	RIKEN CDNA 6430598H11 GENE	0.03098109	0.003365673	0.1635255	1.123884976
1456411_at	RCCD1	RCC1 DOMAIN CONTAINING 1	0.03099857	0.06843517	0.9687064	0.766656597
1431618_a_at	D14Ertd581e	DNA segment, Chr 14, ERATO Doi 581, expressed	0.03099887	0.6359992	0.7385851	0.854849234
1421155_at	B3GALT6	UDP-GAL:BETAGAL BETA 1,3-GALACTOSYLTRANSFERASE, POLYPEPTIDE 6	0.03100383	0.2961159	0.3856761	0.825672538
1448616_at	DVL2	DISHEVELLED 2, DSH HOMOLOG (DROSOPHILA)	0.03100802	0.7160406	0.1971251	1.418461166
1426539_at	USP11	UBIQUITIN SPECIFIC PEPTIDASE 11	0.0310127	0.2051804	0.741781	0.883128061
1434720_at	9530033F24RIK	RIKEN CDNA 9530033F24 GENE	0.03111025	0.5893303	0.2955581	0.81641823
1446861_at	GNS	GLUCOSAMINE (N-ACETYL)-6-SULFATASE	0.03112942	0.7580105	0.08817699	0.844620724
1442309_at	---	---	0.03113214	0.06441998	0.624839	1.305388232
1437455_a_at	Btg1	B-cell translocation gene 1, anti-proliferative	0.03123738	0.2050186	0.3222917	0.624381296
1449085_at	PHF10	PHD FINGER PROTEIN 10	0.0312786	0.006930547	0.3105624	1.090118001
1442135_at	GM237	GENE MODEL 237, (NCBI)	0.03129396	0.5491506	0.1757548	0.708160698
1420977_at	Man1a2	mannosidase, alpha, class 1A, member 2	0.03134084	0.000237376	0.2272642	1.095340076
1457762_at	TTC15	TETRATRICOPEPTIDE REPEAT DOMAIN 15	0.03134438	0.05138673	0.1459759	0.841264184
1436603_at	TBL2	TRANSDUCIN (BETA)-LIKE 2	0.03135108	0.7795308	0.972701	0.829143341
1449747_at	BCDIN3	BIN3, BICOID-INTERACTING 3, HOMOLOG (DROSOPHILA)	0.03138064	0.04044382	0.5427921	0.604103939
1451397_at	TNRC15	RIKEN CDNA 2610016F01 GENE	0.03139347	3.83638E-05	0.2174369	1.07073971
1435114_at	WDHD1	WD REPEAT AND HMG-BOX DNA BINDING PROTEIN 1	0.03142432	0.05175173	0.3507326	0.83854463
1418159_at	TCFCP2	TRANSCRIPTION FACTOR CP2	0.0314557	0.03309139	0.8427372	0.831342303
1416545_at	ZDHHC7	ZINC FINGER, DHHC DOMAIN CONTAINING 7	0.03146288	0.08833976	0.3969911	1.183258628
1431904_at	4933427G17Rik	RIKEN cDNA 4933427G17 gene	0.03146797	0.8717679	0.9886416	0.549371684
1438715_at	---	---	0.03147401	0.8808973	0.3328736	0.515329203
1435877_at	STK38L	SERINE/THREONINE KINASE 38 LIKE	0.03150664	0.000384093	0.29404409	1.074536029
1455359_at	C130080N23RIK	RIKEN CDNA C130080N23 GENE	0.03154391	0.218193	0.299096	1.185559317
1455276_x_at	1110004E09RIK	RIKEN CDNA 1110004E09 GENE	0.03156532	0.07902838	0.5492458	1.732909878
1438328_at	Hcfc2	host cell factor C2	0.03158644	0.2243732	0.973171	0.828574901
1429235_at	GALNTL2	UDP-N-ACETYL-ALPHA-D-GALACTOSAMINE:POLYPEPTIDE N-ACETYLGALACTOSAMINYLTRANSFERASE-LIKE 2	0.03159883	0.01640824	0.1000898	1.352288326
1434487_at	MEF2D	MYOCYTE ENHANCER FACTOR 2D	0.03164845	0.8956902	0.3875831	0.824276795
1456681_at	AI848149	EXPRESSED SEQUENCE AI848149	0.031682509	0.05672674	0.4888948	1.183703886
1455489_at	LRRTM2	LEUCINE RICH REPEAT TRANSMEMBRANE NEURONAL 2	0.0316883	0.000262469	0.60411859	1.11219033
1439476_at	Dsg2	desmoglein 2	0.03168942	0.580713	0.8403996	0.642351594
1438891_at	CTNND2	CATENIN (CADHERIN ASSOCIATED PROTEIN), DELTA 2	0.0317482	0.8283647	0.976292	1.240191722
1437355_at	ZCCHC5	ZINC FINGER, CCHC DOMAIN CONTAINING 5	0.03174995	0.192816	0.5815586	0.347698731
1428800_a_at	Pus7l	pseudouridylate synthase 7 homolog (S. cerevisiae)-like	0.0317539	0.07973516	0.6068485	0.635113249
1431612_at	1700007J10RIK	RIKEN CDNA 1700007J10 GENE	0.03176565	0.4539628	0.09087265	1.982803653
1435100_at	A130042E20RIK	RIKEN CDNA A130042E20 GENE	0.031770069	0.2063585	0.842418	0.671905963
1440106_at	TCF4	TRANSCRIPTION FACTOR 4	0.03185422	0.05051813	0.483011	1.19690161
1430101_at	6330406O05RIK	RIKEN CDNA 6330406O05 GENE	0.03188319	0.3144583	0.5544736	1.267100325
1427592_at	PCDH7	PROTOCADHERIN 7	0.03190216	0.002170475	0.1366964	1.142315205
1431464_a_at	PMM2	PHOSPHOMANNOMUTASE 2	0.03192455	0.06601168	0.6232466	1.193567759
1454330_at	4933415J04RIK	RIKEN CDNA 4933415J04 GENE	0.03197283	0.000586316	0.05918698	0.492390867
1447364_x_at	Myo1b	myosin IB	0.03199798	0.1185481	0.8613172	1.613186561
1441994_at	PCDHB16	PROTOCADHERIN BETA 16	0.03201265	6.8551E-05	0.05410128	0.835964402
1450172_at	PKNOX1	PBX/KNOTTED 1 HOMEOBOX	0.03206403	0.008348616	0.9646947	1.193897503
1416823_a_at	OSBPL1A	OXYSTEROL BINDING PROTEIN-LIKE 1A	0.03208172	0.06784011	0.0666436	1.063426891
1455840_at	RAPGEF5	RAP GUANINE NUCLEOTIDE EXCHANGE FACTOR (GEF) 5	0.03211797	0.04517515	0.9304426	0.863493438
1439843_at	Camk4	calcium/calmodulin-dependent protein kinase IV	0.03214712	0.2932491	0.2942442	1.141039659
1431151_at	AI747699	EXPRESSED SEQUENCE AI747699	0.03217191	0.8340157	0.3424783	0.606211505
1434909_at	Rragd	Ras-related GTP binding D	0.0321773	0.2357366	0.2639043	1.26125827
1443205_at	D5BUC30E	DNA SEGMENT, CHR 5, BUCAN 30 EXPRESSED	0.0322358	0.3141249	0.2193167	0.855662431
1457876_at	D9ERTD496E	DNA SEGMENT, CHR 9, ERATO DOI 496, EXPRESSED	0.03224007	0.1485661	0.392302	2.579891928
1458754_at	---	---	0.03224353	0.3148941	0.5260082	0.510029046
1425279_at	PDIK1L	PDLIM1 INTERACTING KINASE 1 LIKE	0.032252669	0.08480186	0.2935657	1.11653828
1431869_at	5730419F03RIK	RIKEN CDNA 5730419F03 GENE	0.03226135	0.3019943	0.4382975	0.559725768
1420563_at	GRIA3	GLUTAMATE RECEPTOR, IONOTROPIC, AMPA3 (ALPHA 3)	0.03226265	0.00430477	0.5601857	1.158807437
1417483_at	NFKBIZ	NUCLEAR FACTOR OF KAPPA LIGHT POLYPEPTIDE GENE ENHANCER IN B-CELLS INHIBITOR, ZETA	0.03226283	0.3262189	0.4286226	0.695472734
1453573_at	HIST1H3D	HISTONE 2, H3C1	0.03230355	0.6445705	0.910942	1.33118314
1427635_at	KIF5C	KINESIN FAMILY MEMBER 5C	0.03231055	0.5447145	0.3047797	1.296516796
1433050_at	5730478J17RIK	RIKEN CDNA 5730478J17 GENE	0.03234512	0.01394186	0.1356634	1.407517259
1447683_x_at	METTL1	METHYLTRANSFERASE-LIKE 1	0.03237271	0.000786769	0.3377759	0.679897336
1439712_at	4921521J11RIK	RIKEN CDNA 4921521J11 GENE	0.03240486	0.7355018	0.2673396	1.432188053
1429305_at	ANKRD10	ANKYRIN REPEAT DOMAIN 10	0.03243991	0.001695122	0.2527724	1.201230001
1424905_a_at	SLC39A11	SOLUTE CARRIER FAMILY 39 (METAL ION TRANSPORTER), MEMBER 11	0.03244235	0.4899847	0.965818	0.859248231
1440336_at	PRMT6	PROTEIN ARGININE N-METHYLTRANSFERASE 6	0.03246264	0.5787422	0.108171	1.304969098
1423853_at	6330527O06RIK	RIKEN CDNA 3110035N03 GENE	0.032485729	0.4292645	0.8949885	0.727871471
1448935_at	CDYL	CHROMODOMAIN PROTEIN, Y CHROMOSOME-LIKE	0.0324944	0.5169909	0.1211965	1.276155481
1431106_a_at	9530053H05RIK	RIKEN CDNA 9530053H05 GENE	0.0324955	0.05697061	0.5071856	1.360417512
1428342_at	Rcor3	REST corepressor 3	0.03250442	0.04585413	0.4743517	0.731108061
1437074_at	SNX5	SORTING NEXIN 5	0.03254664	0.07486961	0.3909595	0.773430393
1447244_at	DAK	DIHYDROXYACETONE KINASE 2 HOMOLOG (YEAST)	0.0325567	0.5574686	0.823766	0.398840269
1445240_at	AU019796	EXPRESSED SEQUENCE AU019796	0.03262334	0.577335	0.1564708	0.63150629
1439499_at	AA415398	EXPRESSED SEQUENCE AA415398	0.0326583	0.8264058	0.4180878	0.752354507
1458215_at	SETDB1	ERG-ASSOCIATED PROTEIN	0.03271348	0.6426604	0.8478235	0.644411105
1432524_at	4930550G17RIK	RIKEN CDNA 4930550G17 GENE	0.032727319	0.8427106	0.2303635	0.680643876
1449931_at	CPEB4	CYTOPLASMIC POLYADENYLATION ELEMENT BINDING PROTEIN 4	0.03278377	0.0178657	0.4315665	1.197942565
1419984_s_at	ZFP644	ZINC FINGER PROTEIN 644	0.032803	0.003810318	0.7789127	0.844801686
1438750_at	ATRX	ALPHA THALASSEMIA/MENTAL RETARDATION SYNDROME X-LINKED HOMOLOG (HUMAN)	0.03281546	0.2032703	0.5199898	0.769881797
1458746_at	A430107O13RIK	RIKEN CDNA A430107O13 GENE	0.03282731	0.5104839	0.2751589	2.834908002
1433994_at	4931406P16RIK	RIKEN CDNA 4931406P16 GENE	0.03284457	0.08158164	0.1205159	1.089604546
1429189_at	1110007C02RIK	RIKEN CDNA 1110007C02 GENE	0.0328816	0.6667218	0.2501563	0.826957
1456789_at	Zfp462	Zinc finger protein 462	0.03289322	0.4745259	0.9474466	0.750524339
1424515_at	B230354K17RIK	RIKEN CDNA B230354K17 GENE	0.03292239	2.33565E-09	0.07591277	0.835781456
1424726_at	BC014685	CDNA SEQUENCE BC014685	0.03303992	0.5693221	0.08524554	1.159179742
1434138_at	PRUNE	PRUNE HOMOLOG (DROSOPHILA)	0.03306694	0.1776885	0.1382616	1.081430129
1446129_at	---	---	0.03309573	0.4928533	0.61090219	0.774535985
1435339_at	KCTD15	POTASSIUM CHANNEL TETRAMERISATION DOMAIN CONTAINING 15	0.03311747	0.1531547	0.3552005	1.145357359
1421159_at	CGNL1	CINGULIN-LIKE 1	0.03314334	0.6361594	0.8088903	3.078014502
1440602_at	GRIK2	GLUTAMATE RECEPTOR, IONOTROPIC, KAINATE 2 (BETA 2)	0.03315276	0.08469167	0.9439325	2.074650593
1442272_at	---	---	0.033165	0.04535551	0.7301195	0.629933281
1453901_at	ALDOA-PS2	ALDOLASE 1, A ISOFORM	0.03319721	0.09506367	0.2341636	0.378174197
1428200_a_at	NTAN1	N-TERMINAL ASN AMIDASE	0.03319882	0.9369554	0.922464	0.784110937
1443157_at	---	---	0.03325073	0.02289211	0.05192224	0.76298145
1418454_at	MFAP5	MICROFIBRILLAR ASSOCIATED PROTEIN 5	0.03327126	0.2505815	0.05317259	2.001989233
1444912_at	---	---	0.03332964	0.006068337	0.5306162	0.725942686
1417586_at	TIMELESS	TIMELESS HOMOLOG (DROSOPHILA)	0.03335939	0.2702577	0.910411	0.615691258
1449951_at	NFKBIL1	NUCLEAR FACTOR OF KAPPA LIGHT POLYPEPTIDE GENE ENHANCER IN B-CELLS INHIBITOR-LIKE 1	0.03337646	0.05707799	0.3217753	0.763131452
1442809_at	SCN9A	SODIUM CHANNEL, VOLTAGE-GATED, TYPE IX, ALPHA	0.03337765	0.9884192	0.3706025	0.505117838
1421350_a_at	GRIP1	GLUTAMATE RECEPTOR INTERACTING PROTEIN 1	0.033403839	0.3894599	0.6176099	0.748919109
1454424_at	2610040L17RIK	RIKEN CDNA 2610040L17 GENE	0.0334263	0.00020392	0.1886568	1.218254238
1445784_at	4933435A13Rik	RIKEN cDNA 4933435A13 gene	0.03342734	0.7659344	0.4733461	1.596743192
1459068_at	TMEM109	TRANSMEMBRANE PROTEIN 109	0.03345989	0.8399177	0.07603008	0.8609883
1425642_at	---	---	0.0335102	0.02392595	0.4110219	0.708339311
1446790_at	C78948	EXPRESSED SEQUENCE C78948	0.0335427	0.3676271	0.7459491	1.828255988
1425818_at	4930520O04RIK	RIKEN CDNA 4930520O04 GENE	0.03355124	0.6032379	0.9118107	0.573100032
1449520_at	AI428795	CDNA SEQUENCE BC002262	0.03360949	5.70363E-05	0.9657244	0.737491586
1438515_at	ZFP207	ZINC FINGER PROTEIN 207	0.03361596	0.6742237	0.180039	1.249165474
1430210_at	C330024D12RIK	RIKEN CDNA C330024D12 GENE	0.03362784	0.3787913	0.2337118	0.731732934
1422287_at	PHXR2	PER-HEXAMER REPEAT GENE 2	0.03363049	0.5080953	0.1803749	1.830277406
1454390_at	LRCH4	LEUCINE-RICH REPEATS AND CALPONIN HOMOLOGY (CH) DOMAIN CONTAINING 4	0.03377658	0.00458478	0.8569227	0.810817031
1431491_at	9430087N24RIK	RIKEN CDNA 9430087N24 GENE	0.03382349	0.8903093	0.06057233	0.816015619
1454180_at	ARMC2	ARMADILLO REPEAT CONTAINING 2	0.03382379	0.1299316	0.05504515	0.8510952
1444723_at	6530418L21Rik	RIKEN cDNA 6530418L21 gene	0.03387008	0.000520625	0.6995653	1.177173026
1452207_at	CITED2	CBP/P300-INTERACTING TRANSACTIVATOR, WITH GLU/ASP-RICH CARBOXY-TERMINAL DOMAIN, 2	0.03390016	0.2211882	0.7146817	0.852162874
1449166_at	S100A14	S100 CALCIUM BINDING PROTEIN A14	0.03390533	0.5290678	0.5853338	2.368406969
1437453_s_at	PCSK9	PROPROTEIN CONVERTASE SUBTILISIN/KEXIN TYPE 9	0.03397619	0.7585979	0.4054893	0.588002716
1420709_s_at	DAO1	D-AMINO ACID OXIDASE 1	0.0339803	0.4998962	0.648992	0.615125723
1454946_at	MYBL2	MYELOBLASTOSIS ONCOGENE-LIKE 2	0.03399505	0.8659674	0.08213426	1.631973329
1445109_at	WDR4	WD REPEAT DOMAIN 4	0.03399603	0.1419791	0.2078563	0.697969385
1449716_s_at	NRD1	NARDILYSIN, N-ARGININE DIBASIC CONVERTASE, NRD CONVERTASE 1	0.03407208	0.5194289	0.5694737	0.880677089
1433805_at	JAK1	JANUS KINASE 1	0.03408024	0.002910987	0.4977259	0.80672305
1423757_x_at	Igfbp4	insulin-like growth factor binding protein 4	0.03409992	0.7572461	0.9974344	1.279516007
1455204_at	Pitpnc1	phosphatidylinositol transfer protein, cytoplasmic 1	0.03416177	0.7389013	0.8518733	1.273999224
1429637_at	2210419I08RIK	RIKEN CDNA 2210419I08 GENE	0.03416528	0.8172003	0.5061972	1.543744589
1434572_at	HDAC9	HISTONE DEACETYLASE 9	0.03417097	0.4248855	0.7129647	1.266034833
1424900_at	SLC29A4	SOLUTE CARRIER FAMILY 29 (NUCLEOSIDE TRANSPORTERS), MEMBER 4	0.03417657	0.001991134	0.5415968	0.649312522
1420567_at	PRKCN	PROTEIN KINASE C, NU	0.03418463	0.0269634	0.4089421	1.249365892
1425316_at	B3GAT1	BETA-1,3-GLUCURONYLTRANSFERASE 1 (GLUCURONOSYLTRANSFERASE P)	0.03421077	0.983729	0.2549395	1.342374968
1425090_s_at	KCNC4	POTASSIUM VOLTAGE GATED CHANNEL, SHAW-RELATED SUBFAMILY, MEMBER 4	0.03425071	0.6528623	0.3926349	0.764852051
1427051_at	TNKS1BP1	TANKYRASE 1 BINDING PROTEIN 1	0.03428747	0.6263691	0.245045	1.226852656
1452089_at	CACNB4	CALCIUM CHANNEL, VOLTAGE-DEPENDENT, BETA 4 SUBUNIT	0.03431745	0.04217331	0.1297595	1.137887835
1438380_at	DDX47	DEAD (ASP-GLU-ALA-ASP) BOX POLYPEPTIDE 47	0.03432892	0.3725565	0.4164173	2.225995629
1424944_at	PCP2	PURKINJE CELL PROTEIN 2 (L7)	0.03433466	0.3831385	0.2219188	1.968834608
1452009_at	9130422G05RIK	RIKEN CDNA 9130422G05 GENE	0.03433512	0.7252271	0.6500447	1.190925546
1420048_at	C78859	EXPRESSED SEQUENCE C78859	0.03437392	0.6399688	0.7403554	0.832452812
1424856_at	ATP1A3	ATPASE, NA+/K+ TRANSPORTING, ALPHA 3 POLYPEPTIDE	0.03444234	0.01419535	0.6198142	0.784483413
1454157_a_at	PLA2G2D	PHOSPHOLIPASE A2, GROUP IID	0.03455451	0.1154856	0.2668851	0.571135738
1431642_at	EIF2S3Y	EUKARYOTIC TRANSLATION INITIATION FACTOR 2, SUBUNIT 3, STRUCTURAL GENE Y-LINKED	0.034567	0.4656364	0.7374582	0.724117683
1434663_at	2410129H14RIK	RIKEN CDNA 2410129H14 GENE	0.03461474	4.2256E-05	0.6278242	0.910776711
1418358_at	SMCP	SPERM MITOCHONDRIA-ASSOCIATED CYSTEINE-RICH PROTEIN	0.03466002	0.6972465	0.4750296	0.329190753
1416614_at	CRI1	CREBBP/EP300 INHIBITORY PROTEIN 1	0.03470814	0.722358	0.3697848	0.808572352
1433674_a_at	Snord22	small nucleolar RNA, C/D box 22	0.03472052	0.007361358	0.5582314	0.618255464
1434069_at	BC067047	CDNA SEQUENCE BC067047	0.03476655	0.1849044	0.3950437	1.247576457
1450024_at	SUFU	SUPPRESSOR OF FUSED HOMOLOG (DROSOPHILA)	0.03476747	0.3919825	0.4278802	0.87470196
1447117_at	Ctnna2	Catenin (cadherin associated protein), alpha 2	0.03478023	0.7567966	0.1642349	1.204606032

1427357_at	CDA	CYTIDINE DEAMINASE	0.03478602	6.22154E-06	0.308009	1.327959623
1444796_at	C76876	EXPRESSED SEQUENCE C76876	0.03478856	0.5045629	0.1750963	1.704227238
1441623_at	---	---	0.03479689	0.1272233	0.4170674	2.342947664
1436426_at	5730509K17RIK	RIKEN CDNA 5730509K17 GENE	0.03484504	0.008591909	0.32936349	0.811872278
1420589_at	HAS3	HYALURONAN SYNTHASE 3	0.03487623	0.01816901	0.6327899	1.960961065
1424546_at	BC003965	CDNA SEQUENCE BC003965	0.03497071	0.792418	0.881497	0.836661492
1452244_at	6330406I15RIK	RIKEN CDNA 6330406I15 GENE	0.03497887	0.3957583	0.7071891	0.627141481
1436615_a_at	OTC	ORNITHINE TRANSCARBAMYLASE	0.03497959	0.3703814	0.5500729	0.483681707
1447281_at	---	---	0.03498865	0.1546845	0.05522284	2.035547544
1422277_at	GLRA1	GLYCINE RECEPTOR, ALPHA 1 SUBUNIT	0.0350244	0.01135665	0.7663103	0.698751056
1449548_at	Efnb2	ephrin B2	0.03504837	0.006027242	0.8444251	1.266037629
1444084_at	PRRG4	PROLINE RICH GLA (G-CARBOXYGLUTAMIC ACID) 4 (TRANSMEMBRANE)	0.03505481	0.05815142	0.7274314	1.544994771
1445424_at	ADRA1D	ADRENERGIC RECEPTOR, ALPHA 1D	0.03509085	0.05413668	0.960257	1.427562684
1447344_at	---	---	0.03511888	0.03743914	0.3973927	1.214344746
1427571_at	SHH	SONIC HEDGEHOG	0.03513537	0.0168973	0.1651339	0.62268636
1434156_at	RAB11FIP4	RAB11 FAMILY INTERACTING PROTEIN 4 (CLASS II)	0.03513577	0.999138	0.5888399	1.203682177
1442755_at	PANK2	PANTOTHENATE KINASE 2 (HALLERVORDEN-SPATZ SYNDROME)	0.03514545	0.7019035	0.3601418	1.552095438
1424703_at	HEMK1	HEMK METHYLTRANSFERASE FAMILY MEMBER 1	0.0351528	0.005062818	0.909476	1.207293355
1429845_at	1700019L03RIK	RIKEN CDNA 1700019L03 GENE	0.03517118	0.8051304	0.33817	1.586663206
1429313_at	ROR1	RECEPTOR TYROSINE KINASE-LIKE ORPHAN RECEPTOR 1	0.03517229	0.3576804	0.4175476	0.590700849
1422791_at	PAFAH1B2	PLATELET-ACTIVATING FACTOR ACETYLHYDROLASE, ISOFORM 1B, ALPHA2 SUBUNIT	0.03521533	0.006429635	0.60873809	1.079248836
1416111_at	CD83	CD83 ANTIGEN	0.03524227	0.6747701	0.5330676	0.715717038
1416173_at	PES1	PESCADILLO HOMOLOG 1, CONTAINING BRCT DOMAIN (ZEBRAFISH)	0.0353552	0.5930617	0.5929732	0.845496181
1417164_at	DUSP10	DUAL SPECIFICITY PHOSPHATASE 10	0.03536202	0.0791314	0.09094224	0.84288562
1424880_at	TRIB1	TRIBBLES HOMOLOG 1 (DROSOPHILA)	0.03538977	0.8013604	0.08764636	1.935572517
1438265_at	WWC2	DNA SEGMENT, CHR 8, ERATO DOI 594, EXPRESSED	0.03540212	0.7025498	0.1895193	0.878902109
1429234_s_at	Sept11	septin 11	0.03546521	0.9630746	0.8400161	1.1973214
1452280_at	FARP1	FERMRHOGEF (ARHGEF) AND PLECKSTRIN DOMAIN PROTEIN 1 (CHONDROCYTE-DERIVED)	0.03552745	0.4076252	0.2399914	1.187096722
1419178_at	CD3G	CD3 ANTIGEN, GAMMA POLYPEPTIDE	0.03559366	0.8784198	0.792674	2.170575406
1433125_at	5330422M15RIK	RIKEN CDNA 5330422M15 GENE	0.03560893	0.00916222	0.074575	0.908384889
1458051_at	A230048O21RIK	RIKEN CDNA A230048O21 GENE	0.03561235	0.5886284	0.5556388	3.133665267
1441481_at	MFAP3L	MICROFIBRILLAR-ASSOCIATED PROTEIN 3-LIKE	0.03563561	0.6714651	0.466439	1.174076521
1438243_at	RPS6KA4	RIBOSOMAL PROTEIN S6 KINASE, POLYPEPTIDE 4	0.03564408	0.3171436	0.5000109	1.915407334
1420307_a_at	PITPNB	PHOSPHATIDYLINOSITOL TRANSFER PROTEIN, BETA	0.03567077	0.04512988	0.490272	1.108568881
1437124_at	A630052C17RIK	RIKEN CDNA A630052C17 GENE	0.03567287	0.7853696	0.500554	0.731295478
1420785_at	GAB2	GROWTH FACTOR RECEPTOR BOUND PROTEIN 2-ASSOCIATED PROTEIN 2	0.03567978	0.003108082	0.06144965	1.318624516
1429200_at	SAAL1	SERUM AMYLOID A-LIKE 1	0.03571079	0.01372933	0.3900767	1.157531451
1442056_at	Zfp608	Zinc finger protein 608	0.03572325	0.02006874	0.8856658	1.265459681
1430505_at	1110032O16RIK	RIKEN CDNA 1110032O16 GENE	0.03573083	0.8684091	0.111376	1.560767839
1438033_at	TEF	THYROTROPH EMBRYONIC FACTOR	0.03576254	0.0567297	0.5480686	0.807021523
1424138_at	RHBDF1	RHOMBOID FAMILY 1 (DROSOPHILA)	0.03580473	0.08568331	0.3073806	1.130289113
1454229_a_at	4933424N09RIK	RIKEN CDNA 4933424N09 GENE	0.03584423	0.000190787	0.1932982	0.816027124
1422115_a_at	FLT3L	FMS-LIKE TYROSINE KINASE 3 LIGAND	0.03587873	0.4549742	0.681934	2.316915058
1447222_at	Hspa12a	heat shock protein 12A	0.03595962	0.0764281	0.5660456	1.281696062
1422673_at	PRKCM	PROTEIN KINASE C, MU	0.03600258	0.4704506	0.6252491	0.7547607
1455756_at	F730014I05RIK	RIKEN CDNA F730014I05 GENE	0.03602228	0.05595735	0.8799683	1.173180134
1427782_a_at	Crhr1	corticotropin releasing hormone receptor 1	0.03603002	0.763944	0.6988662	1.295099052
1429350_at	1700027M21RIK	RIKEN CDNA 1700027M21 GENE	0.0361848	0.9665933	0.6726316	0.455889303
1455262_at	THSD4	THROMBOSPONDIN, TYPE I, DOMAIN CONTAINING 4	0.03619179	0.6239446	0.2507276	0.84374421
1452862_at	Rreb1	ras responsive element binding protein 1	0.0362479	0.7603489	0.9385186	0.715456085
1458052_at	---	---	0.03626233	0.4173477	0.7564731	1.581505906
1436338_at	PPP2R1B	PROTEIN PHOSPHATASE 2 (FORMERLY 2A), REGULATORY SUBUNIT A (PR 65), BETA ISOFORM	0.03634942	0.2994423	0.4921862	0.842654208
1459544_at	---	---	0.03636331	0.678946	0.6784757	1.408581825
1434766_at	PRKAA2	PROTEIN KINASE, AMP-ACTIVATED, ALPHA 2 CATALYTIC SUBUNIT	0.03637836	0.4531977	0.07732672	1.09147061
1453059_at	2310046A06RIK	RIKEN CDNA 2310046A06 GENE	0.036379	0.07078024	0.559972	1.141663404
1420666_at	Doc2b	double C2, beta	0.03642034	0.8584059	0.4693911	0.76083515
1416518_at	H1FOO	H1 HISTONE FAMILY, MEMBER O, OOCYTE-SPECIFIC	0.03643326	0.07830445	0.08780129	0.380565076
1428432_at	2310047A01RIK	RIKEN CDNA 2310047A01 GENE	0.03644216	0.2985226	0.5143689	1.147426167
1424494_s_at	2810417J12Rik	RIKEN cDNA 2810417J12 gene	0.03644929	0.003900393	0.288433	1.136661596
1450157_a_at	HMMR	HYALURONAN MEDIATED MOTILITY RECEPTOR (RHAMM)	0.03646173	0.7022962	0.5970584	0.443347171
1441226_at	Spon1	spondin 1, (f-spondin) extracellular matrix protein	0.03648614	0.9548653	0.2482077	0.795839379
1442071_at	ABCE1	ATP-BINDING CASSETTE, SUB-FAMILY E (OABP), MEMBER 1	0.0365683	0.4797357	0.09710683	0.837732712
1445821_at	IL1RAPL1	INTERLEUKIN 1 RECEPTOR ACCESSORY PROTEIN-LIKE 1	0.0365868	0.8761647	0.7752718	0.391979987
1442923_at	PTK6	PTK6 PROTEIN TYROSINE KINASE 6	0.03671299	0.93981	0.6019147	0.2952553
1422643_at	MOXD1	MONOOXYGENASE, DBH-LIKE 1	0.03672313	0.6515107	0.732727	0.780698002
1460302_at	THBS1	THROMBOSPONDIN 1	0.03674687	0.4013688	0.4642201	1.901402656
1429666_at	KCTD16	POTASSIUM CHANNEL TETRAMERISATION DOMAIN CONTAINING 16	0.03674742	0.01267784	0.1430754	1.110526474
1439905_at	---	---	0.03675854	0.883746	0.1378564	0.650713072
1460197_a_at	STEAP4	STEAP FAMILY MEMBER 4	0.03677358	0.06498151	0.07279315	1.753675631
1421941_at	Camk4	calcium/calmodulin-dependent protein kinase IV	0.03677744	0.2216339	0.9284385	1.267500203
1417277_at	CYP4F16	CYTOCHROME P450, FAMILY 4, SUBFAMILY F, POLYPEPTIDE 16	0.03678203	0.5167429	0.2423883	1.31964586
1422894_at	SFMBT1	SCM-LIKE WITH FOUR MBT DOMAINS 1	0.03683474	0.5370218	0.3645638	1.229044885
1458721_at	Pcdhgc3	Protocadherin gamma subfamily C, 3	0.03685041	0.9709793	0.9399757	1.397670722
1422040_at	SEMA7A	SEMA DOMAIN, IMMUNOGLOBULIN DOMAIN (IG), AND GPI MEMBRANE ANCHOR, (SEMAPHORIN) 7A	0.03688238	0.003700965	0.2333336	1.193421562
1428914_at	2310014D11RIK	RIKEN CDNA 2310014D11 GENE	0.03689256	0.2878318	0.3379987	0.820183512
1443594_at	AI043046	EXPRESSED SEQUENCE AI043046	0.03691911	0.1422711	0.8273471	1.653303883
1425562_s_at	TRNT1	TRNA NUCLEOTIDYL TRANSFERASE, CCA-ADDING, 1	0.03694244	0.1471864	0.9823527	0.802559787
1446598_at	PRKCA	PROTEIN KINASE C, ALPHA	0.03702378	0.6952257	0.14689369	1.223595336
1422826_at	IGFALS	INSULIN-LIKE GROWTH FACTOR BINDING PROTEIN, ACID LABILE SUBUNIT	0.03703573	0.2138523	0.9495626	1.85778973
1454419_at	4930537H20RIK	RIKEN CDNA 4930537H20 GENE	0.03704614	0.3820263	0.1878241	0.748196287
1444268_at	BC004004	CDNA SEQUENCE BC004004	0.03706061	0.000974454	0.2425042	1.136044169
1430280_at	1810062G17RIK	RIKEN CDNA 1810062G17 GENE	0.0370901	0.4466981	0.05256132	0.709871694
1445313_at	COQ9	COENZYME Q9 HOMOLOG (YEAST)	0.0371088	0.5685119	0.93934	1.475843265
1436366_at	PPP1R15B	EXPRESSED SEQUENCE AI606441	0.03711112	7.08974E-06	0.1190014	1.151872829
1419985_s_at	CCDC69	COILED-COIL DOMAIN CONTAINING 69	0.03713158	0.4522742	0.7518328	0.599642924
1418360_at	ZFP179	ZINC FINGER PROTEIN 179	0.03713497	0.01494607	0.911157	0.812957823
1434177_at	ECE1	ENDOTHELIN CONVERTING ENZYME 1	0.03715498	0.000115403	0.08311039	1.036664652
1440781_at	B830007D08RIK	RIKEN CDNA B830007D08 GENE	0.03717799	0.0580138	0.4489302	0.634560506
1437476_at	RRM2B	RIBONUCLEOTIDE REDUCTASE M2 B (TP53 INDUCIBLE)	0.03718333	0.816184	0.2714641	0.777109586
1429198_at	1810030O07RIK	RIKEN CDNA 1810030O07 GENE	0.03719645	0.3501306	0.4596373	0.897686183
1421516_at	NR6A1	NUCLEAR RECEPTOR SUBFAMILY 6, GROUP A, MEMBER 1	0.0372129	0.54719759	0.1504084	0.780758105
1441272_at	MATR3	MATRIN 3	0.03730503	0.2337305	0.3568538	0.864510811
1436235_x_at	4732471D19Rik	RIKEN cDNA 4732471D19 gene	0.03731361	0.6974062	0.4727847	0.747188745
1425788_a_at	ECHDC2	ENOYL COENZYME A HYDRATASE DOMAIN CONTAINING 2	0.03736113	0.05094055	0.2722415	0.796371941
1442455_at	C79845	EXPRESSED SEQUENCE C79845	0.03740399	0.493259	0.8347304	1.61694247
1425831_at	ZFP101	ZINC FINGER PROTEIN 101	0.03746962	0.7634346	0.4645326	0.542773591
1439373_x_at	WNT5B	WINGLESS-RELATED MMTV INTEGRATION SITE 5B	0.03747366	0.05166941	0.6470712	1.338324134
1420585_a_at	NXF2	NUCLEAR RNA EXPORT FACTOR 2	0.03754243	0.1789662	0.7084279	0.682293006
1427242_at	DDX4	DEAD (ASP-GLU-ALA-ASP) BOX POLYPEPTIDE 4	0.03754455	0.016150979	0.2283058	0.678911522
1446560_at	Prss23	protease, serine, 23	0.03756983	0.55354879	0.882816	1.41914799
1452198_at	FBXL10	F-BOX AND LEUCINE-RICH REPEAT PROTEIN 10	0.03759686	0.2330091	0.64700539	0.863739109
1454421_at	4930423D22RIK	RIKEN CDNA 4930423D22 GENE	0.03760412	0.3750684	0.7732308	1.850140177
1417289_at	Plekha2	pleckstrin homology domain-containing, family A (phosphoinositide binding specific) member 2	0.03760892	1.42174E-07	0.2870586	0.851321598
1440360_at	ARL6IP5	ADP-RIBOSYLATION FACTOR-LIKE 6 INTERACTING PROTEIN 5	0.03768249	0.4726307	0.5155841	0.810384445
1421817_at	GSR	GLUTATHIONE REDUCTASE 1	0.03772589	0.3194454	0.3410377	1.143290606
1443948_at	TTC9C	TETRATRICOPEPTIDE REPEAT DOMAIN 9C	0.03776007	0.2251336	0.2659143	1.093151992
1459484_at	Tmem2	Transmembrane protein 2	0.03777495	0.016688189	0.1123311	0.575460268
1420625_at	2410016F19RIK	RIKEN CDNA 2410016F19 GENE	0.03779138	0.1953572	0.05503196	1.123114233
1448804_at	CYP11A1	CYTOCHROME P450, FAMILY 11, SUBFAMILY A, POLYPEPTIDE 1	0.0378002	0.3493393	0.08513874	1.824912534
1440364_a_at	A230062G08RIK	RIKEN CDNA A230062G08 GENE	0.03780257	0.0357136	0.6583346	0.532187154
1423977_at	4930453N24RIK	RIKEN CDNA 4930453N24 GENE	0.03787715	0.739717	0.1203158	1.478774548
1453144_at	4933439C20RIK	RIKEN CDNA 4933439C20 GENE	0.03788009	0.2064399	0.2734304	1.550886521
1425136_x_at	DNM2	DYNAMIN 2	0.0378992	0.3994296	0.1805981	0.900064141
1442538_at	Rcor3	REST corepressor 3	0.03790881	0.05444616	0.5395958	0.814786628
1420665_at	Itgb3bp	integrin beta 3 binding protein (beta3-endonexin)	0.03791066	0.3705958	0.5707408	0.722316626
1451811_at	CACNG6	CALCIUM CHANNEL, VOLTAGE-DEPENDENT, GAMMA SUBUNIT 6	0.03793727	0.2401259	0.1414077	0.798980697
1419204_at	DLL1	DELTA-LIKE 1 (DROSOPHILA)	0.0379909	0.1748785	0.870344	0.806320979
1418678_at	HAS2	HYALURONAN SYNTHASE 2	0.03799443	0.1258642	0.1502396	0.771575989
1422046_at	ITGAM	INTEGRIN ALPHA M	0.03800174	0.06501819	0.1942311	0.847084013
1421905_at	NCOA6IP	NUCLEAR RECEPTOR COACTIVATOR 6 INTERACTING PROTEIN	0.03800632	2.12108E-05	0.05120063	1.035632457
1432371_a_at	1700109K24RIK	RIKEN CDNA 1700109K24 GENE	0.03800982	0.1266138	0.3809868	2.980011158
1440200_at	9630031F12Rik	RIKEN cDNA 9630031F12 gene	0.03804129	0.009567261	0.06095473	1.162445423
1438749_at	2600013E07RIK	RIKEN CDNA 2600013E07 GENE	0.03812473	0.830078	0.0570907	0.690966223
1443570_at	COPS3	COP9 (CONSTITUTIVE PHOTOMORPHOGENIC) HOMOLOG, SUBUNIT 3 (ARABIDOPSIS THALIANA)	0.03815169	0.3308918	0.05869758	2.067377372
1416675_s_at	Plcd1	phospholipase C, delta 1	0.03820661	0.02702919	0.534505	1.156303426
1439980_at	D14ERTD725E	DNA SEGMENT, CHR 14, ERATO DOI 725, EXPRESSED	0.03822712	0.268324	0.819466	1.461271906
1440494_at	MRPS7	MITOCHONDRIAL RIBOSOMAL PROTEIN S7	0.03824512	0.3565187	0.30701479	1.534922409
1434837_at	MDC1	MEDIATOR OF DNA DAMAGE CHECKPOINT 1	0.03825726	0.08500567	0.5428016	0.825976176
1430161_at	DLST	DIHYDROLIPOAMIDE S-SUCCINYLTRANSFERASE (E2 COMPONENT OF 2-OXO-GLUTARATE COMPLEX)	0.03836982	0.4493322	0.5507744	1.512518092
1443895_at	---	---	0.03848255	0.5548944	0.5455291	1.892181389
1459345_at	---	---	0.03855885	0.3844617	0.418951	1.639187163
1421943_at	Tgfa	transforming growth factor alpha	0.03857756	0.4729983	0.5635454	0.603111763
1452042_a_at	5730537D05RIK	RIKEN CDNA 5730537D05 GENE	0.03863207	0.527045	0.5380317	1.179506087
1418691_at	RGS9	REGULATOR OF G-PROTEIN SIGNALING 9	0.03864116	0.1038733	0.5139316	0.825835285
1455898_x_at	Slc2a3	solute carrier family 2 (facilitated glucose transporter), member 3	0.03871135	0.1117494	0.6385862	0.722234415
1428820_at	MAPRE1	MICROTUBULE-ASSOCIATED PROTEIN, RP/EB FAMILY, MEMBER 1	0.03873648	0.002506041	0.05262596	1.050078346
1460606_at	HSD17B13	HYDROXYSTEROID (17-BETA) DEHYDROGENASE 13	0.03876598	0.6451903	0.3433107	0.414566081
1447414_at	---	---	0.03877463	0.7171219	0.5635705	2.19113053
1441353_at	---	---	0.03878588	0.30010759	0.2982837	0.580693936
1440695_at	---	---	0.03880131	2.69319E-07	0.5221065	0.760263155
1434592_at	SLC16A10	SOLUTE CARRIER FAMILY 16 (MONOCARBOXYLIC ACID TRANSPORTERS), MEMBER 10	0.03881207	0.001661413	0.4179974	0.614328191
1442305_at	Gtpbp2	GTP binding protein 2	0.03885703	0.008710349	0.2845514	1.160498795
1429523_a_at	SLC39A5	SOLUTE CARRIER FAMILY 39 (METAL ION TRANSPORTER), MEMBER 5	0.03886485	0.4164753	0.7014453	0.542163692
1444665_at	4930402E16RIK	RIKEN CDNA 4930402E16 GENE	0.03903907	0.2617136	0.2783131	1.24098929
1431903_at	4930524C18RIK	RIKEN CDNA 4930524C18 GENE	0.03905523	0.9196325	0.2263888	2.390066279
1438217_at	A2BP1	ATAXIN 2 BINDING PROTEIN 1	0.03905961	0.7210725	0.9398877	1.214035609
1435985_at	STK25	SERINE/THREONINE KINASE 25 (YEAST)	0.03906486	0.01309629	0.1696551	1.117223868
1417000_at	ABTB1	ANKYRIN REPEAT AND BTB (POZ) DOMAIN CONTAINING 1	0.03906787	0.9436551	0.4767339	1.116415924
1460101_at	Nrxn3	Neurexin III	0.0391053	0.008550425	0.3349771	1.295317602
1423631_at	NR2E3	NUCLEAR RECEPTOR SUBFAMILY 2, GROUP E, MEMBER 3	0.03914208	0.9417375	0.84945	2.667442669
1435618_at	PNMA2	PARANEOPLASTIC ANTIGEN MA2	0.03914237	0.7553693	0.9317215	0.835713956
1429378_x_at	AY761184	RIKEN CDNA 2010300L12 GENE	0.03918147	0.9174716	0.95209	0.508906105
1439479_at	LCT	LACTASE	0.0391909	0.01036771	0.6962358	0.718054188
1419008_at	NPY5R	NEUROPEPTIDE Y RECEPTOR Y5	0.03922995	0.05271115	0.9992674	1.226486315
1453055_at	SEMA6D	RIKEN CDNA 1110067B02 GENE	0.03925502	0.01192106	0.1837188	1.137622074
1439839_at	D130051D11RIK	RIKEN CDNA D130051D11 GENE	0.0392724	0.1038729	0.4159365	1.54597603
1418824_at	ARF6	ADP-RIBOSYLATION FACTOR 6	0.03927463	0.0516323	0.2520027	0.624358038
1417856_at	RELB	AVIAN RETICULOENDOTHELIOSIS VIRAL (V-REL) ONCOGENE RELATED B	0.03927741	0.4433693	0.1939911	1.312847693
1455355_at	6030408C04RIK	RIKEN CDNA 6030408C04 GENE	0.03929367	0.000168756	0.1404803	1.122384538

1421034_a_at	IL4RA	INTERLEUKIN 4 RECEPTOR, ALPHA	0.03931418	0.7158558	0.07024461	1.516127539
1419668_at	SGCB	SARCOGLYCAN, BETA (DYSTROPHIN-ASSOCIATED GLYCOPROTEIN)	0.03933531	0.4466463	0.3949115	0.850701587
1423978_at	SBK1	SH3-BINDING KINASE 1	0.03933532	0.114866	0.08920523	1.456480051
1424037_at	ITPKA	INOSITOL 1,4,5-TRISPHOSPHATE 3-KINASE A	0.03933578	0.1924209	0.9349646	1.2360436
1445403_at	---	---	0.03936924	0.665872	0.560154	1.732524766
1451714_a_at	MAP2K3	MITOGEN ACTIVATED PROTEIN KINASE KINASE 3	0.03943159	0.3251309	0.2217997	0.891258977
1434567_at	4732496O08RIK	RIKEN CDNA 4732496O08 GENE	0.03949924	0.07966028	0.08136688	1.173047385
1417489_at	NPY2R	NEUROPEPTIDE Y RECEPTOR Y2	0.03951918	0.7741262	0.3823943	0.681643298
1460574_at	FAT4	HYPOTHETICAL PROTEIN 9430004M15	0.03952528	0.00245924	0.7554875	0.751255562
1447906_at	---	---	0.0395263	0.194397	0.1423083	0.734722357
1420462_at	IL1RAPL2	INTERLEUKIN 1 RECEPTOR ACCESSORY PROTEIN-LIKE 2	0.0395757	0.1012278	0.2740104	0.580306922
1441455_at	Arpp19	CAMP-regulated phosphoprotein 19	0.0396327	0.1191993	0.6464816	1.652690338
1448432_at	Plcd1	phospholipase C, delta 1	0.03965826	0.2054446	0.5520881	1.359138575
1448751_at	Ap3m2	adaptor-related protein complex 3, mu 2 subunit	0.03966598	0.04621616	0.743643	1.104011497
1429794_a_at	P2RX1	PURINERGIC RECEPTOR P2X, LIGAND-GATED ION CHANNEL, 1	0.0396715	0.1781295	0.7071697	0.472864397
1433992_at	APXL	APICAL PROTEIN, XENOPUS LAEVIS-LIKE	0.03969003	0.1946388	0.5230953	0.726757697
1417324_at	MAST2	MICROTUBULE ASSOCIATED SERINE/THREONINE KINASE 2	0.0397511	0.2966847	0.8987348	1.137501176
1424498_at	5730596K20RIK	RIKEN CDNA 4633402C03 GENE	0.03978462	0.1006777	0.5992489	0.690514684
1447894_x_at	VPS52	SAC2 (SUPRESSOR OF ACTIN MUTATIONS 2, HOMOLOG)-LIKE (S. CEREVISIAE)	0.03981602	0.4731091	0.3746967	0.785088604
1419507_at	KRTAP15	KERATIN ASSOCIATED PROTEIN 15	0.03982942	0.04634807	0.05978838	0.480615138
1423644_at	ACO1	ACONITASE 1	0.03984307	0.0318159	0.8612423	0.88131189
1452342_at	APBB2	AMYLOID BETA (A4) PRECURSOR PROTEIN-BINDING, FAMILY B, MEMBER 2	0.03984751	0.2647544	0.1613914	1.126597448
1437716_x_at	KIF22	EXPRESSED SEQUENCE AL033313	0.03988169	0.1096493	0.4024961	1.153642121
1458117_at	GARNL1	GTPASE ACTIVATING RANGAP DOMAIN-LIKE 1	0.03988346	0.8037641	0.148682	0.858772127
1452199_at	2700094F01Rik	RIKEN cDNA 2700094F01 gene	0.03988458	0.0176538	0.2671309	0.819719151
1435727_s_at	D15ERTD366E	DNA SEGMENT, CHR 15, ERATO DOI 366, EXPRESSED	0.03991537	0.8808587	0.7920333	0.779906266
1434625_at	4930432O21RIK	RIKEN CDNA 4930432O21 GENE	0.03996235	0.5831588	0.590779	0.760594421
1447690_at	LSR	LIPOLYSIS STIMULATED LIPOPROTEIN RECEPTOR	0.03996314	0.1701539	0.3768962	3.516295004
1432130_a_at	Ttc14	tetratricopeptide repeat domain 14	0.03997319	0.006549569	0.728671	0.821383199
1446623_at	---	---	0.03999678	0.1991666	0.3590131	1.430328734
1459931_a_at	2810021G02RIK	RIKEN CDNA 2810021G02 GENE	0.04001911	0.04212297	0.7280052	1.191852111
1445840_at	---	---	0.04006027	0.6387497	0.46489	1.959428735
1443244_at	ZADH1	ZINC BINDING ALCOHOL DEHYDROGENASE, DOMAIN CONTAINING 1	0.04011303	0.08894987	0.3071416	0.818985528
1438982_s_at	2810417J12Rik	RIKEN cDNA 2810417J12 gene	0.04014717	0.02568177	0.1677187	1.231671617
1429741_at	Kcnv1	potassium channel, subfamily V, member 1	0.04015715	0.4183579	0.8531571	1.216059089
1419097_a_at	STOM	STOMATIN	0.04015716	0.07118483	0.6884252	0.869455942
1452418_at	1200016E24RIK	RIKEN CDNA 1200016E24 GENE	0.04016549	0.04785405	0.4591458	1.212915018
1432062_at	MDGA1	MAM DOMAIN CONTAINING GLYCOSYLPHOSPHATIDYLINOSITOL ANCHOR 1	0.04016909	0.4616441	0.8121055	0.606368422
1426015_s_at	Asph	aspartate-beta-hydroxylase	0.04019006	0.08803171	0.30384489	0.828080232
1442166_at	CPNE5	COPINE V	0.040204	0.03805735	0.8789466	1.190821903
1437793_at	TNKS2	TANKYRASE, TRF1-INTERACTING ANKYRIN-RELATED ADP-RIBOSE POLYMERASE 2	0.04025739	0.1388897	0.6028426	0.889233955
1428952_at	PDIA2	PROTEIN DISULFIDE ISOMERASE ASSOCIATED 2	0.04026329	0.1683541	0.16277349	1.380799379
1421382_at	PRLR	PROLACTIN RECEPTOR	0.04026489	0.5915604	0.3161141	1.917193617
1448923_at	PRKRA	PROTEIN KINASE, INTERFERON INDUCIBLE DOUBLE STRANDED RNA DEPENDENT ACTIVATOR	0.04028884	0.1230992	0.400271	0.76732408
1435980_x_at	WNT6	WINGLESS-RELATED MMTV INTEGRATION SITE 6	0.04031214	0.6696085	0.9939472	0.615937145
1456137_at	Nrxn3	neurexin III	0.04033586	0.007904957	0.8455167	1.253074831
1431040_at	LOC638977	RIKEN CDNA 4930578F06 GENE	0.04036721	0.2784062	0.1471355	1.479056006
1450438_at	Ncam1	neural cell adhesion molecule 1	0.04039822	0.00955198	0.3040202	0.830334034
1429279_at	WARS2	TRYPTOPHANYL TRNA SYNTHETASE 2 (MITOCHONDRIAL)	0.04041123	0.5344065	0.3306681	0.868262769
1454960_at	Smad3	MAD homolog 3 (Drosophila)	0.04041919	0.2889854	0.2369899	1.108040382
1432705_at	5330428N10RIK	RIKEN CDNA 5330428N10 GENE	0.04043486	0.003890396	0.1810214	1.142922083
1452567_at	GCAP8	GRANULE CELL ANTISERUM POSITIVE 8	0.04044179	0.5644831	0.1353456	0.680518006
1454913_at	9930104L06RIK	RIKEN CDNA 9930104L06 GENE	0.04050819	0.1154806	0.8318309	0.751606015
1457936_at	MAPK8	MITOGEN ACTIVATED PROTEIN KINASE 8	0.04056446	0.00343909	0.1616713	1.048749466
1442377_at	---	---	0.04057586	0.94316	0.05910081	0.732232654
1446389_at	NRIP1	NUCLEAR RECEPTOR INTERACTING PROTEIN 1	0.04057891	0.5233231	0.5013798	2.054635626
1458529_at	LOC215714	HYPOTHETICAL PROTEIN A930010C09	0.04057932	0.543908	0.1717178	0.622876098
1460527_at	4933407C09RIK	RIKEN CDNA 4933407C09 GENE	0.04059859	0.06312508	0.2095288	1.524127353
1425646_at	BC016495	CDNA SEQUENCE BC016495	0.04066483	0.2055058	0.3137412	0.721165634
1425994_a_at	ASAH2	N-ACYLSPHINGOSINE AMIDOHYDROLASE 2	0.0406955	0.08219898	0.2653245	1.32777286
1450716_at	ADAMTS1	A DISINTEGRIN-LIKE AND METALLOPEPTIDSE (REPROLYSIN TYPE) WITH THROMBOSPONDIN TYPE 1 MOTIF, 1	0.04080299	0.6004644	0.7021431	0.782916531
1434096_at	Slc4a4	solute carrier family 4 (anion exchanger), member 4	0.04085548	0.7274648	0.6043668	0.811684106
1455462_at	Adcy2	adenylate cyclase 2	0.04086269	0.01314885	0.2318252	1.090585642
1437702_at	TGM6	TRANSGLUTAMINASE 6	0.04101583	0.8850442	0.06417721	0.675515321
1434966_at	SFRS8	SPLICING FACTOR, ARGININE/SERINE-RICH 8	0.04102276	0.001013382	0.2004278	1.045731878
1456809_at	---	---	0.04102776	0.08256196	0.8834819	0.753301967
1423968_at	UGT3A2	EXPRESSED SEQUENCE AI313915	0.04103782	0.851254	0.1151358	1.855160579
1455739_at	6430704N06	HYPOTHETICAL PROTEIN 6430704N06	0.04105071	0.0389716	0.1727044	0.921448956
1445029_at	A330019N05Rik	RIKEN cDNA A330019N05 gene	0.04111433	0.2619983	0.9801267	0.745589138
1441184_at	TRPC4AP	TRANSIENT RECEPTOR POTENTIAL CATION CHANNEL, SUBFAMILY C, MEMBER 4 ASSOCIATED PROTEIN	0.04114258	0.7423148	0.9987894	0.446694078
1456328_at	BANK1	B-CELL SCAFFOLD PROTEIN WITH ANKYRIN REPEATS 1	0.0411667	0.4505757	0.5587626	0.772048556
1448400_a_at	SMARCD2	SWI/SNF RELATED, MATRIX ASSOCIATED, ACTIN DEPENDENT REGULATOR OF CHROMATIN, SUBFAMILY D, MEMBER 2	0.04117877	0.722602	0.4800987	0.748543363
1441654_at	GNAL	GUANINE NUCLEOTIDE BINDING PROTEIN, ALPHA 10	0.04123801	0.0581567	0.5843277	1.425863725
1427522_at	ARHGAP20	RIKEN CDNA 6530403F17 GENE	0.04128877	0.001446296	0.16944	1.09762023
1444704_at	---	7 days neonate cerebellum cDNA, RIKEN full-length enriched library, clone:A730050L22 product:unclassifiable, full insert sequence	0.04133876	0.02658393	0.4556495	1.297322969
1433071_at	6430605C03RIK	RIKEN CDNA 6430605C03 GENE	0.04136794	0.1029711	0.65154	0.417385031
1448050_s_at	Map4k4	mitogen-activated protein kinase kinase kinase kinase 4	0.04136971	0.2159237	0.146195	1.253029298
1435723_at	A530089L17	HYPOTHETICAL PROTEIN A530089L17	0.04139959	0.1489666	0.3496524	0.616008926
1450096_at	GNA12	GUANINE NUCLEOTIDE BINDING PROTEIN, ALPHA 12	0.04144853	0.2423687	0.09866665	0.78809644
1438375_at	Fbln2	Fibulin 2	0.04145537	0.1702177	0.4032543	1.770720848
1427950_at	ZFP294	ZINC FINGER PROTEIN 294	0.04147385	0.001009917	0.3585136	1.084012291
1421268_at	UGCG	UDP-GLUCOSE CERAMIDE GLUCOSYLTRANSFERASE	0.0415474	1.60436E-06	0.1019116	1.020211639
1438018_at	Hook1	hook homolog 1 (Drosophila)	0.04155642	0.04953124	0.8504175	0.814518827
1454501_at	6330419E04RIK	RIKEN CDNA 6330419E04 GENE	0.04160884	0.08643595	0.3676444	0.770308601
1455727_at	U2AF1-RS2	U2 SMALL NUCLEAR RIBONUCLEOPROTEIN AUXILIARY FACTOR (U2AF) 1, RELATED SEQUENCE 2	0.04163797	0.000732838	0.6483704	0.909654489
1433590_at	HERC3	HECT DOMAIN AND RLD 3	0.04166161	0.000199118	0.156692	0.792673909
1446321_at	B230208B08RIK	RIKEN CDNA B230208B08 GENE	0.04167567	0.169558	0.5596275	1.620999372
1425896_a_at	FBN1	FIBRILLIN 1	0.04171001	0.6627323	0.7223801	0.698771894
1442498_at	C78662	EST C78662	0.04171729	0.29777369	0.1682583	2.361260577
1437433_at	B3GALT2	UDP-GAL:BETAGLCNAC BETA 1,3-GALACTOSYLTRANSFERASE, POLYPEPTIDE 2	0.04171847	0.01249913	0.681291	0.736327319
1448551_a_at	TRIM2	TRIPARTITE MOTIF PROTEIN 2	0.04174904	2.00516E-11	0.4322264	0.867132157
1454355_at	1810021M19RIK	RIKEN CDNA 1810021M19 GENE	0.04174979	0.6289159	0.2434267	1.71601755
1431170_at	EFNA3	EPHRIN A3	0.04175097	0.1981382	0.3671144	0.910798355
1446503_at	---	---	0.04181307	0.9471918	0.276065	1.682335674
1455303_at	RFXAP	REGULATORY FACTOR X-ASSOCIATED PROTEIN	0.0418197	0.006821534	0.3889789	0.822019315
1441168_at	---	---	0.04182056	0.7757319	0.2742589	0.681506621
1446344_at	NYX	NYCTALOPIN	0.04187372	0.02239268	0.1644147	0.882887422
1439628_x_at	Rab38	Rab38, member of RAS oncogene family	0.04191669	0.000288176	0.95154	1.277557127
1423062_at	IGFBP3	INSULIN-LIKE GROWTH FACTOR BINDING PROTEIN 3	0.04199732	0.691479	0.6661325	1.261052224
1420701_at	KLK1B1	KALLIKREIN 1-RELATED PEPTIDASE B1	0.04204837	0.6355304	0.9293355	0.5300998
1441859_x_at	---	---	0.0420592	0.2575559	0.5233095	1.708815837
1430859_at	5330427O13RIK	RIKEN CDNA 5330427O13 GENE	0.04206392	0.4549421	0.05076199	1.383485433
1441466_at	SFRS10	SPLICING FACTOR, ARGININE/SERINE-RICH 10 (TRANSFORMER 2 HOMOLOG, DROSOPHILA)	0.04207264	0.2647683	0.7095547	0.766966745
1440918_at	RASGRF2	RAS PROTEIN-SPECIFIC GUANINE NUCLEOTIDE-RELEASING FACTOR 2	0.04212968	0.4403666	0.4667366	0.836757293
1432798_at	9530023I19RIK	RIKEN CDNA 9530023I19 GENE	0.04219689	0.6351202	0.7793788	2.508478125
1456852_at	PAIP1	POLYADENYLATE BINDING PROTEIN-INTERACTING PROTEIN 1	0.04221872	0.1969476	0.08424033	1.153250963
1457003_at	AW490526	EXPRESSED SEQUENCE AW490526	0.04222413	0.1263791	0.662518	0.885426681
1423756_s_at	Igfbp4	insulin-like growth factor binding protein 4	0.04225297	0.1758014	0.6883638	1.226115217
1446057_at	RLF	EXPRESSED SEQUENCE AI195322	0.04231788	0.728385	0.1268864	0.883502649
1443024_at	---	---	0.04232458	0.2843929	0.1855106	0.575522843
1438489_at	SMN1	SURVIVAL MOTOR NEURON 1	0.04235575	0.9518344	0.6488737	1.318602412
1436866_at	Efna5	ephrin A5	0.04239805	0.8822063	0.4916796	1.258871906
1442585_at	B230220N19RIK	RIKEN CDNA B230220N19 GENE	0.0424023	0.5242651	0.8324451	1.325192472
1447995_x_at	1700026B20RIK	RIKEN CDNA 1700026B20 GENE	0.04240378	0.6843676	0.3098076	0.898154267
1443247_at	---	---	0.04240543	0.156836	0.6547152	1.258469087
1439412_at	Arpp21	Cyclic AMP-regulated phosphoprotein, 21	0.04241973	0.02259647	0.814121	1.25918609
1447012_at	APP	AMYLOID BETA (A4) PRECURSOR PROTEIN	0.04250646	0.9373188	0.7406668	0.607981503
1455297_at	SPIN2	SPINDLIN FAMILY, MEMBER 2	0.04254499	0.0703654	0.3529875	0.827342449
1443459_at	C86415	EXPRESSED SEQUENCE C86415	0.04256792	0.6317538	0.200485	0.484848545
1415857_at	EMB	EMBIGIN	0.04258417	0.005131567	0.2998548	1.220503819
1427297_at	MRPL9	MITOCHONDRIAL RIBOSOMAL PROTEIN L9	0.04259202	0.004755652	0.3053118	0.88748579
1443385_at	A930012N16RIK	RIKEN CDNA A930012N16 GENE	0.04260148	0.2683173	0.1483327	0.782179092
1435875_at	Prkab2	protein kinase, AMP-activated, beta 2 non-catalytic subunit	0.04263521	0.000311382	0.6460892	0.791031124
1421876_at	MAPK9	MITOGEN ACTIVATED PROTEIN KINASE 9	0.04266754	0.001674827	0.5406086	1.092196971
1427573_at	Chic1	cysteine-rich hydrophobic domain 1	0.04271805	0.3911302	0.8082144	1.2473532
1452075_at	4933435A13Rik	RIKEN cDNA 4933435A13 gene	0.0427343	0.003913093	0.2643898	1.060108736
1432029_a_at	SMAP1	STROMAL MEMBRANE-ASSOCIATED PROTEIN 1	0.0427645	0.00020698	0.07286719	1.025973741
1454152_a_at	GPRK2L	G PROTEIN-COUPLED RECEPTOR KINASE 2, GROUCHO GENE RELATED (DROSOPHILA)	0.04278666	0.9851072	0.1436295	0.694240791
1421087_at	Per3	period homolog 3 (Drosophila)	0.04281458	0.00754347	0.1593168	0.858399128
1453194_at	4930432K21RIK	RIKEN CDNA 4930432K21 GENE	0.04284827	0.4538265	0.6497161	0.700198325
1426551_at	SIDT1	SID1 TRANSMEMBRANE FAMILY, MEMBER 1	0.04286495	0.4711043	0.09708047	0.773059231
1446369_at	ARMC1	ARMADILLO REPEAT CONTAINING 1	0.04287204	0.2237163	0.1291142	0.606275252
1440787_s_at	1300007O09RIK	RIKEN CDNA 1300007O09 GENE	0.0429156	0.950572	0.6524065	0.833517942
1431034_at	GPR125	G PROTEIN-COUPLED RECEPTOR 125	0.04294022	0.05257938	0.3575525	0.549500481
1441324_at	ZFP395	ZINC FINGER PROTEIN 395	0.04296162	0.5921322	0.4493887	1.516325165
1442697_at	---	---	0.04296187	0.2307288	0.66151629	0.663124614
1423926_at	SLC39A13	SOLUTE CARRIER FAMILY 39 (METAL ION TRANSPORTER), MEMBER 13	0.04297755	0.2450632	0.4440412	1.16686904
1421093_at	SLC7A10	SOLUTE CARRIER FAMILY 7 (CATIONIC AMINO ACID TRANSPORTER, Y+ SYSTEM), MEMBER 10	0.04307557	4.00647E-05	0.0915079	1.037047767
1437894_at	Prox1	prospero-related homeobox 1	0.04311456	2.77005E-06	0.8727204	0.686328993
1449237_at	ALOXE3	ARACHIDONATE LIPOXYGENASE 3	0.0431499	0.02860414	0.1663587	0.902916117
1459335_at	---	---	0.04320818	0.4321698	0.4818067	1.315462376
1427826_a_at	SLCO1B2	SOLUTE CARRIER FAMILY 21 (ORGANIC ANION TRANSPORTER), MEMBER 6	0.04335838	0.8217499	0.4263992	3.669549578
1437304_at	Cblb	Casitas B-lineage lymphoma b	0.04341141	0.2487203	0.8583916	0.818290772
1450417_a_at	RPS20	RIBOSOMAL PROTEIN S20	0.04346936	0.2699943	0.3528599	0.7680017
1419707_at	KRTAP14	KERATIN ASSOCIATED PROTEIN 14	0.04347773	0.1861836	0.807317	2.15765648
1459563_x_at	---	---	0.04349227	0.01182145	0.7796722	0.848902152
1435656_at	GMPS	GUANINE MONPHOSPHATE SYNTHETASE	0.04356546	0.2387311	0.7690016	0.756547744
1431773_at	CBX1	CHROMOBOX HOMOLOG 1 (DROSOPHILA HP1 BETA)	0.04360784	0.3232995	0.08126327	0.759773942
1452511_at	SOX5	SRY-BOX CONTAINING GENE 5	0.04361906	0.1437396	0.5763336	1.476931282
1421379_at	ZFP354B	ZINC FINGER PROTEIN 354B	0.04366174	0.1109207	0.1425587	0.865133737
1423349_at	SOCS5	SUPPRESSOR OF CYTOKINE SIGNALING 5	0.04369287	6.3937E-05	0.2936787	1.055170717
1439106_at	Zfp462	zinc finger protein 462	0.04374902	0.7841526	0.8706682	0.892942468
1416376_at	TMEM97	TRANSMEMBRANE PROTEIN 97	0.04376832	0.1117056	0.7681628	1.335411768
1420561_at	TRPC7	TRANSIENT RECEPTOR POTENTIAL CATION CHANNEL, SUBFAMILY C, MEMBER 7	0.04377709	0.004962339	0.3969108	1.317292705
1448437_a_at	Gtpbp2	GTP binding protein 2	0.04378962	0.706844	0.7772032	1.521845404
1430302_at	1500041B16RIK	RIKEN CDNA 1500041B16 GENE	0.04380838	0.01777048	0.07301971	0.880301435
1459219_at	RAPGEF2	RAP GUANINE NUCLEOTIDE EXCHANGE FACTOR (GEF) 2	0.04383565	0.07277706	0.891458	0.776512316
1457637_at	PRICKLE1	PRICKLE LIKE 1 (DROSOPHILA)	0.0438689	0.1914805	0.746682	1.332114666
1447099_at	Grit	Rho GTPase-activating protein	0.0439326	0.07299701	0.3395133	0.72735329

1453340_at	1700057D03RIK	RIKEN CDNA 1700057D03 GENE	0.04394419	0.3812653	0.2058694	0.415445505
1455533_at	A930025D01RIK	RIKEN CDNA A930025D01 GENE	0.04395134	0.5292156	0.806574	0.85043535
1436696_x_at	CEACAM11	CEA-RELATED CELL ADHESION MOLECULE 11	0.04400186	0.4751955	0.33598	2.604067444
1418908_at	PAM	PEPTIDYLGLYCINE ALPHA-AMIDATING MONOOXYGENASE	0.0440267	0.004597639	0.236254	1.139251614
1433675_at	Snord22	small nucleolar RNA, C/D box 22	0.04404044	0.05633047	0.7061879	0.624963827
1421718_at	STRM	STRIAMIN	0.04404096	0.1360005	0.1033266	1.906304955
1458448_at	AA408296	EXPRESSED SEQUENCE AA408296	0.04408097	0.03681782	0.7208608	1.611197213
1433840_a_at	KIAA0415	KIAA0415 PROTEIN	0.0440903	0.5568368	0.5758398	0.868625303
1416732_at	Top2b	topoisomerase (DNA) II beta	0.04412542	0.000384931	0.9663446	1.131136879
1424754_at	MS4A7	MEMBRANE-SPANNING 4-DOMAINS, SUBFAMILY A, MEMBER 7	0.04417628	0.1041287	0.4126719	1.459673852
1442077_at	2310076G05RIK	RIKEN CDNA 2310076G05 GENE	0.04417796	0.9599294	0.5471479	1.196210333
1444921_at	D15ERTD466E	DNA SEGMENT, CHR 15, ERATO DOI 466, EXPRESSED	0.04418241	0.6215804	0.6466755	2.225239777
1422065_at	KLRB1D	KILLER CELL LECTIN-LIKE RECEPTOR SUBFAMILY B MEMBER 1D	0.0442118	0.7678845	0.8043314	0.413676028
1429075_a_at	1700018B08RIK	RIKEN CDNA 1700018B08 GENE	0.04430128	0.0952688	0.09999106	1.465612447
1454943_a_at	PAXIP1	PAX INTERACTING (WITH TRANSCRIPTION-ACTIVATION DOMAIN) PROTEIN 1	0.04430184	0.7668282	0.1199572	0.824314917
1442340_x_at	CYR61	CYSTEINE RICH PROTEIN 61	0.04430472	0.5025667	0.06614327	1.593166018
1420403_at	Atp2b2	ATPase, Ca++ transporting, plasma membrane 2	0.04431103	0.000151675	0.4040829	1.150962152
1425256_a_at	DIXDC1	RIKEN CDNA 4930563F16 GENE	0.04434045	0.6627731	0.09655336	1.501229467
1443623_a_at	6430571L13RIK	RIKEN CDNA 6430571L13 GENE	0.04437651	0.4886309	0.3272387	0.461175455
1452718_at	EDD1	E3 UBIQUITIN PROTEIN LIGASE, HECT DOMAIN CONTAINING, 1	0.04440343	0.3307605	0.8536469	0.878616527
1443273_at	EPHA3	EPH RECEPTOR A3	0.04441256	0.1225804	0.3117249	0.818543236
1447861_x_at	Mrg1	Myeloid ecotropic viral integration site-related gene 1	0.04442718	0.04716395	0.9700014	0.742973629
1449789_x_at	LY6G6C	LYMPHOCYTE ANTIGEN 6 COMPLEX, LOCUS G6C	0.04443495	0.0463636	0.30515759	1.607712353
1434626_at	RPUSD3	RNA PSEUDOURIDYLATE SYNTHASE DOMAIN CONTAINING 3	0.04449289	0.7156474	0.6816527	0.844666275
1445483_at	ABL2	V-ABL ABELSON MURINE LEUKEMIA VIRAL ONCOGENE 2 (ARG, ABELSON-RELATED GENE)	0.04459949	0.003364498	0.8121848	1.618969934
1444259_at	DMGDH	DIMETHYLGLYCINE DEHYDROGENASE PRECURSOR	0.04460209	0.1232559	0.8094244	0.850708223
1435227_at	---	---	0.04461161	0.1992015	0.4959341	0.629379047
1455802_x_at	AGR2	ANTERIOR GRADIENT 2 (XENOPUS LAEVIS)	0.04464285	0.58378909	0.28307149	1.862353568
1435708_at	Gls	Glutaminase	0.04472765	0.2746796	0.1833876	1.386378376
1434807_s_at	MTX3	METAXIN 3	0.04475449	0.7683758	0.6472061	0.821336239
1416929_at	RBM12	RNA BINDING MOTIF PROTEIN 12	0.04479263	0.5115889	0.8761251	0.793861387
1433331_at	C030046M01RIK	RIKEN CDNA C030046M01 GENE	0.0448101	0.408859	0.4636006	0.509826136
1418946_at	ST3GAL1	ST3 BETA-GALACTOSIDE ALPHA-2,3-SIALYLTRANSFERASE 1	0.04485729	0.6388476	0.08329223	1.221674983
1456786_at	Ldb2	LIM domain binding 2	0.04488264	0.2065505	0.3486264	1.389486674
1438144_x_at	ATXN2	ATAXIN 2	0.04488327	0.002665765	0.7850621	1.32641385
1443799_at	PPP1R14C	PROTEIN PHOSPHATASE 1, REGULATORY (INHIBITOR) SUBUNIT 14C	0.04491691	0.06037621	0.3354756	0.762256074
1434402_at	SAMD8	STERILE ALPHA MOTIF DOMAIN CONTAINING 8	0.04497363	0.8636842	0.5143653	0.789086572
1417994_a_at	ACCN1	AMILORIDE-SENSITIVE CATION CHANNEL 1, NEURONAL (DEGENERIN)	0.04501307	0.7698365	0.1253123	0.897859495
1447163_x_at	---	---	0.04508262	0.8648927	0.1533985	1.979514894
1435538_at	0610030E20RIK	RIKEN CDNA 0610030E20 GENE	0.04512488	0.04359704	0.9488425	1.187688955
1419389_at	Pde10a	phosphodiesterase 10A	0.04518683	0.1758686	0.944067	0.830742622
1444923_at	---	---	0.04523419	0.4820385	0.9738973	0.801185762
1419639_at	Efnb2	ephrin B2	0.0452725	0.5883859	0.2020478	1.244851389
1426777_a_at	WASL	WISKOTT-ALDRICH SYNDROME-LIKE (HUMAN)	0.04527822	0.1541494	0.5351303	1.199846242
1449524_at	Eda	ectodysplasin-A	0.04527922	0.2739541	0.902095	0.610287488
1434847_at	CNNM4	CYCLIN M4	0.04535188	0.500355499	0.5693085	0.763351458
1433342_at	5730416F02RIK	RIKEN CDNA 5730416F02 GENE	0.04539249	0.7839371	0.8433754	0.436916786
1443344_at	4932409I22RIK	RIKEN CDNA 4932409I22 GENE	0.04547066	0.002315226	0.8528676	0.836621357
1449248_at	CLCN2	CHLORIDE CHANNEL 2	0.04548468	0.00345307	0.8387598	1.170330918
1452339_at	ADAMTS7	A DISINTEGRIN-LIKE AND METALLOPEPTIDASE (REPROLYSIN TYPE) WITH THROMBOSPONDIN TYPE 1 MOTIF, 7	0.04552291	0.4891338	0.2196926	2.459253156
1451031_at	SFRP4	SECRETED FRIZZLED-RELATED SEQUENCE PROTEIN 4	0.0456234	0.000573655	0.969928	1.314231517
1419332_at	EGFL6	EGF-LIKE-DOMAIN, MULTIPLE 6	0.04566597	0.01778257	0.2818353	0.764624409
1449541_x_at	1700010I14RIK	RIKEN CDNA 1700010I14 GENE	0.04568579	0.7322593	0.6470989	0.557580341
1453512_at	5830407P18RIK	RIKEN CDNA 5830407P18 GENE	0.04570401	0.00126377	0.9441656	1.16075539
1441614_at	---	---	0.04576268	0.6868879	0.06879906	3.650514628
1443033_at	RBM14	RNA BINDING MOTIF PROTEIN 14	0.04578468	0.1172059	0.26988069	0.689116571
1447371_at	LOC236469	SIMILAR TO BLOODTHIRSTY	0.04581455	0.002114818	0.937371	1.69501647
1429053_at	1110012J17RIK	RIKEN CDNA 1110012J17 GENE	0.04598837	0.04078824	0.9034762	1.149908199
1428617_at	Hcfc2	host cell factor C2	0.04603007	0.01457336	0.6575083	0.81269129
1422634_a_at	VSIG2	V-SET AND IMMUNOGLOBULIN DOMAIN CONTAINING 2	0.04603234	0.58003059	0.6848077	2.486208887
1453708_a_at	GSTO2	GLUTATHIONE S-TRANSFERASE OMEGA 2	0.0460536	0.8900247	0.7453888	0.661625606
1416613_at	CYP1B1	CYTOCHROME P450, FAMILY 1, SUBFAMILY B, POLYPEPTIDE 1	0.04608691	0.2546359	0.3542178	0.764994486
1429354_at	THEM5	THIOESTERASE SUPERFAMILY MEMBER 5	0.04610351	0.4233295	0.4017412	0.584415329
1433101_at	9030419F21RIK	RIKEN CDNA 9030419F21 GENE	0.0461043	0.7845699	0.6507721	0.835756375
1436813_x_at	KHSRP	KH-TYPE SPLICING REGULATORY PROTEIN	0.04611411	0.01595247	0.8962657	0.726372643
1434881_s_at	KCTD12	EXPRESSED SEQUENCE AW538430	0.04611849	0.5001721	0.5323439	0.733850177
1454358_at	2900060N12RIK	RIKEN CDNA 2900060N12 GENE	0.0462579	0.2984858	0.602957	1.272304615
1446719_at	---	---	0.04629599	0.8461043	0.7252972	1.69877642
1455712_at	HIST3H2A	RIKEN CDNA 4930534G10 GENE	0.04630782	0.739934	0.9345096	0.742536265
1429987_at	9930013L23RIK	RIKEN CDNA 6330404C01 GENE	0.04633164	0.3376052	0.4206435	0.760046228
1451288_s_at	1810043G02RIK	RIKEN CDNA 1810043G02 GENE	0.04636717	0.4600545	0.6626569	0.851111349
1458878_at	Yes1	Yamaguchi sarcoma viral (v-yes) oncogene homolog 1	0.04638255	0.269915	0.3438086	0.732674782
1416498_at	PPIC	PEPTIDYLPROLYL ISOMERASE C	0.04639045	0.51578	0.8717211	0.792759458
1447280_at	DTNA	DYSTROBREVIN ALPHA	0.04643545	0.3971804	0.6974171	1.489493994
1419005_at	CRYBB3	CRYSTALLIN, BETA B3	0.04647171	0.04132488	0.3477504	0.805292922
1451056_at	PSMD7	PROTEASOME (PROSOME, MACROPAIN) 26S SUBUNIT, NON-ATPASE, 7	0.04648035	0.001650097	0.08921371	1.050631911
1451423_at	TBCC	TUBULIN-SPECIFIC CHAPERONE C	0.04650313	0.54575359	0.2394199	0.854410577
1420497_a_at	CEBPZ	CCAAT/ENHANCER BINDING PROTEIN ZETA	0.04651124	0.001261425	0.144838	1.074694117
1426885_a_at	CDK2AP1	CDK2 (CYCLIN-DEPENDENT KINASE 2)-ASSOCIATED PROTEIN 1	0.04657932	0.4881159	0.8479115	1.240696847
1459310_at	---	---	0.0466122	0.5235605	0.66827289	2.209462797
1426561_a_at	Npnt	nephronectin	0.04661764	0.3492287	0.2010062	0.485948003
1424214_at	9130213B05RIK	RIKEN CDNA 9130213B05 GENE	0.04663603	0.03246229	0.7304484	1.143951501
1435310_at	Fbxo7	F-box only protein 7	0.04665068	0.2785339	0.3251697	1.34964723
1457032_at	ZZZ3	ZINC FINGER, ZZ DOMAIN CONTAINING 3	0.04665573	0.1003191	0.772762	0.744681138
1447167_at	AL033314	EXPRESSED SEQUENCE AL033314	0.04665802	0.908217	0.1459587	0.907475386
1421036_at	NPAS2	NEURONAL PAS DOMAIN PROTEIN 2	0.04670141	0.6776079	0.5975239	1.287757521
1451339_at	SUOX	SULFITE OXIDASE	0.04670695	0.06567688	0.8541942	0.847855331
1415961_at	ITM2C	INTEGRAL MEMBRANE PROTEIN 2C	0.04676984	0.07905616	0.5419709	0.760042279
1456489_at	PCF11	RIKEN CDNA 2500001H09 GENE	0.04680097	0.4813906	0.5866207	1.192317114
1455011_at	STARD4	RIKEN CDNA 4632419C16 GENE	0.04688656	0.1601602	0.4815964	1.156890243
1442918_at	NAV3	NEURON NAVIGATOR 3	0.04689345	0.02243558	0.4542318	1.100511406
1416050_a_at	SCARB1	SCAVENGER RECEPTOR CLASS B, MEMBER 1	0.04689898	0.1334222	0.4987985	1.207535718
1433204_at	2810436B12RIK	RIKEN CDNA 2810436B12 GENE	0.04694537	0.07878955	0.3922931	0.832244627
1418514_at	MTF2	METAL RESPONSE ELEMENT BINDING TRANSCRIPTION FACTOR 2	0.04696955	0.00393346	0.7490012	1.091038491
1435741_at	PDE8B	PHOSPHODIESTERASE 8B	0.04697406	0.7934646	0.9400881	0.805282769
1429655_at	NUDCD1	NUDC DOMAIN CONTAINING 1	0.0469847	0.000199676	0.2787764	0.83835222
1456842_at	---	---	0.04698837	0.0703547	0.6855822	0.512122308
1442252_at	KIF13A	KINESIN FAMILY MEMBER 13A	0.04704626	0.1269475	0.4679884	1.28650028
1453334_at	B230216N24RIK	RIKEN CDNA B230216N24 GENE	0.04706328	0.3385182	0.1327724	0.855726278
1440393_at	ODZ2	ODD OZ/TEN-M HOMOLOG 2 (DROSOPHILA)	0.0470718	0.2569302	0.3454676	2.147812722
1436865_at	SLC26A11	SOLUTE CARRIER FAMILY 26, MEMBER 11	0.04711666	0.2871879	0.1985105	0.864549489
1420066_s_at	Cd160	CD160 antigen	0.04715023	0.9950167	0.6295227	2.404890959
1451863_at	UTS2R	UROTENSIN 2 RECEPTOR	0.04716319	0.4020848	0.3713586	2.023008255
1448973_at	SULT1D1	SULFOTRANSFERASE FAMILY 1D, MEMBER 1	0.04721128	0.9682193	0.660649	2.130348765
1421353_at	PDE7B	PHOSPHODIESTERASE 7B	0.04722454	0.04854857	0.0798614	0.843343078
1451264_at	FRMD6	RIKEN CDNA 2610019M19 GENE	0.04733276	0.0123229	0.2093062	1.084155069
1439717_at	B230362M20RIK	RIKEN CDNA B230362M20 GENE	0.04737068	0.185559	0.1109268	1.230652859
1451241_at	LAMB1-1	LAMININ B1 SUBUNIT 1	0.04737183	0.01098234	0.4360869	0.725918521
1424792_at	RPP40	RIBONUCLEASE P 40 SUBUNIT (HUMAN)	0.04742137	0.1509233	0.7937372	0.812625892
1427072_at	STARD8	START DOMAIN CONTAINING 8	0.04749449	0.7165273	0.1256604	1.159241743
1432445_at	2310016G11RIK	RIKEN CDNA 2310016G11 GENE	0.04750288	0.4989227	0.6868409	0.517267801
1434033_at	Tle1	transducin-like enhancer of split 1, homolog of Drosophila E(spl)	0.04751332	0.2190797	0.750294	0.789460292
1460280_at	GRAP2	GRB2-RELATED ADAPTOR PROTEIN 2	0.04752155	0.9872165	0.9675172	0.535986028
1452390_at	AA881470	EST AA881470	0.04752478	0.6920191	0.9442278	0.782620465
1449546_a_at	ZFP617	ZINC FINGER PROTEIN 617	0.04754155	0.001572041	0.5653789	0.922321291
1434927_at	HSPB7	HEAT SHOCK PROTEIN FAMILY, MEMBER 7 (CARDIOVASCULAR)	0.04754859	0.6974744	0.05273378	1.550024372
1449703_at	ZFAND2A	ZINC FINGER, AN1-TYPE DOMAIN 2A	0.04760037	0.1613036	0.5538821	1.450077705
1445791_at	---	---	0.04760671	0.3971967	0.6219735	0.537952679
1442287_at	---	---	0.04763797	0.0511998	0.8620464	0.709888188
1436906_at	RNF166	RING FINGER PROTEIN 166	0.04767417	0.05222808	0.3883039	1.167072038
1432600_at	2310061A09RIK	RIKEN CDNA 2310061A09 GENE	0.04768353	0.6461741	0.7575488	1.865477308
1421802_at	EAR1	EOSINOPHIL-ASSOCIATED, RIBONUCLEASE A FAMILY, MEMBER 1	0.04769022	0.08804683	0.1767906	1.742051888
1454901_at	6430570G24	HYPOTHETICAL PROTEIN 6430570G24	0.047761	0.8481759	0.1133572	1.102152382
1436680_s_at	Ddb2	damage specific DNA binding protein 2	0.04776537	0.3098209	0.7235758	0.674029912
1418492_at	GREM2	GREMLIN 2 HOMOLOG, CYSTEINE KNOT SUPERFAMILY (XENOPUS LAEVIS)	0.04778352	0.003988799	0.619594	0.893125003
1427066_a_at	4933439F18Rik	RIKEN cDNA 4933439F18 gene	0.04779284	0.1131068	0.6863996	0.87510567
1455661_at	---	---	0.04788955	0.169288	0.4279387	0.863784883
1419356_at	KLF7	KRUPPEL-LIKE FACTOR 7 (UBIQUITOUS)	0.04790842	0.01451508	0.2808072	1.112759858
1418173_at	4631426H08RIK	RIKEN CDNA 4631426H08 GENE	0.04794301	0.8111408	0.1639699	1.675172071
1417139_at	1700022L09RIK	RIKEN CDNA 1700022L09 GENE	0.0479817	0.007302657	0.5010492	0.906495766
1460608_at	CACNA1B	CALCIUM CHANNEL, VOLTAGE-DEPENDENT, N TYPE, ALPHA 1B SUBUNIT	0.04798914	0.01110302	0.4267102	0.784468008
1416716_at	EFS	EMBRYONAL FYN-ASSOCIATED SUBSTRATE	0.04803427	0.6699558	0.2617228	1.148816303
1456624_at	WIPI1	WD REPEAT DOMAIN, PHOSPHOINOSITIDE INTERACTING 1	0.04804782	0.4776073	0.5532971	0.839079859
1430013_at	2210008F06RIK	RIKEN CDNA 2210008F06 GENE	0.04804901	0.5438651	0.4007743	0.827243058
1429420_at	4931414P19RIK	RIKEN CDNA 4931414P19 GENE	0.0480615	0.3781661	0.5451111	1.243912438
1435187_at	TOMM20	TRANSLOCASE OF OUTER MITOCHONDRIAL MEMBRANE 20 HOMOLOG (YEAST)	0.04807666	0.893321	0.1979543	0.789136755
1459618_at	TREX1	THREE PRIME REPAIR EXONUCLEASE 1	0.04812628	0.3567602	0.60179629	1.78624223
1438820_at	Rnf17	ring finger protein 17	0.04813961	0.2765398	0.06813612	0.807623485
1444077_at	---	---	0.04815178	0.04560829	0.2824612	1.160198367
1440152_x_at	EDF1	ENDOTHELIAL DIFFERENTIATION-RELATED FACTOR 1	0.04815642	0.2940041	0.5495637	0.671681222
1429764_at	1500005K14Rik	RIKEN cDNA 1500005K14 gene	0.04817339	0.1954672	0.1171256	0.704977162
1451237_s_at	RBM7	RNA BINDING MOTIF PROTEIN 7	0.04820581	0.1612778	0.4096986	0.869803846
1449469_at	PKD2L2	POLYCYSTIC KIDNEY DISEASE 2-LIKE 2	0.04823415	0.8605565	0.8696432	1.964517655
1439300_at	---	---	0.04825146	0.1753883	0.6006179	1.186672171
1452906_at	GTL2	GTL2, IMPRINTED MATERNALLY EXPRESSED UNTRANSLATED MRNA	0.04828828	0.002760447	0.3087387	1.08024547
1430129_a_at	COMMD8	COMM DOMAIN CONTAINING 8	0.04832253	0.2239668	0.8624208	0.784033864
1435580_at	C230081A13RIK	HYPOTHETICAL PROTEIN BC026466	0.04834106	0.5858896	0.7550405	1.196082852
1453028_at	4631424J17RIK	RIKEN CDNA 4631424J17 GENE	0.04834637	0.1645962	0.4815774	0.77674829
1442307_at	---	---	0.04836256	0.1487589	0.9774475	1.220947842
1436588_at	RTTN	EXPRESSED SEQUENCE AI666264	0.04837762	0.771536	0.6334997	0.674197948
1437130_at	---	---	0.0484098	0.6668088	0.9810506	0.679818283
1421762_at	KCNJ5	POTASSIUM INWARDLY-RECTIFYING CHANNEL, SUBFAMILY J, MEMBER 5	0.04843568	0.0847796	0.9336595	1.551186163
1426356_at	6330578E17RIK	RIKEN CDNA 6330578E17 GENE	0.04845262	0.006414642	0.1318802	1.04200033
1452682_at	4632404H22RIK	RIKEN CDNA 4632404H22 GENE	0.04847843	0.7254901	0.6162935	0.85709612
1424265_at	NPL	N-ACETYLNEURAMINATE PYRUVATE LYASE	0.04853471	0.7036266	0.2604217	0.848107673
1429417_at	LOC635492	RIKEN CDNA 4833446K15 GENE	0.04859732	0.006769585	0.8337263	1.634457347
1430904_at	ARFGAP3	ADP-RIBOSYLATION FACTOR GTPASE ACTIVATING PROTEIN 3	0.04860432	0.08994825	0.3310423	1.465792825
1417172_at	UBE2L6	UBIQUITIN-CONJUGATING ENZYME E2L 6	0.04863313	0.1063631	0.6510403	1.569081387
1427770_a_at	Slc2a3	solute carrier family 2 (facilitated glucose transporter), member 3	0.04865111	0.05778833	0.2810637	0.758355147

1420582_at	CD209E	CD209E ANTIGEN	0.0487239	0.5692887	0.4050335	2.637701798
1422747_at	CHEK2	CHK2 CHECKPOINT HOMOLOG (S. POMBE)	0.04876758	0.1684693	0.4513832	0.740062663
1448618_at	MVP	MAJOR VAULT PROTEIN	0.04882369	0.2394245	0.303222	1.225705278
1441936_x_at	---	---	0.04885487	0.2471208	0.8148172	0.795712589
1443164_at	VPS13D	VACUOLAR PROTEIN SORTING 13D (YEAST)	0.04885491	0.7419631	0.4574515	1.559262908
1460399_at	BC018601	CDNA SEQUENCE BC018601	0.04892584	0.004775218	0.6618422	1.190310396
1422169_a_at	Bdnf	brain derived neurotrophic factor	0.04894924	0.9389392	0.236067	1.353794539
1441806_at	CHD1L	CHROMODOMAIN HELICASE DNA BINDING PROTEIN 1-LIKE	0.04899539	0.7018542	0.4968076	1.647511038
1449752_at	SPSB1	SPLA/RYANODINE RECEPTOR DOMAIN AND SOCS BOX CONTAINING 1	0.0490082	0.6756749	0.4316653	1.503045893
1440973_at	LOC552874	HYPOTHETICAL LOC552874	0.04902615	0.9342804	0.2780802	1.218307429
1448069_at	TM4SF1	TRANSMEMBRANE 4 SUPERFAMILY MEMBER 1	0.04903619	0.7234735	0.09881945	0.690652631
1445618_at	---	---	0.04909033	3.52451E-05	0.4813784	0.685338676
1432237_at	5730522E02RIK	RIKEN CDNA 5730522E02 GENE	0.04912053	0.3424898	0.3537554	0.816197829
1422125_at	HTR2B	5-HYDROXYTRYPTAMINE (SEROTONIN) RECEPTOR 2B	0.04916555	0.123756	0.6259502	2.206544508
1446927_at	---	---	0.04922584	0.06761749	0.8714413	0.600158594
1416935_at	TRPV2	TRANSIENT RECEPTOR POTENTIAL CATION CHANNEL, SUBFAMILY V, MEMBER 2	0.04923812	0.4231931	0.8176565	1.245555723
1430107_at	9230116B18RIK	RIKEN CDNA 9230116B18 GENE	0.04928557	0.5699192	0.57312149	0.57663568
1430260_at	---	---	0.04928807	0.09970136	0.05513704	1.399470449
1450472_s_at	Smad3	MAD homolog 3 (Drosophila)	0.04934396	0.07085675	0.4339804	1.230677983
1425149_a_at	PDCL	PHOSDUCIN-LIKE	0.04935565	0.002218962	0.7099298	0.834717191
1430030_at	5330426P16RIK	RIKEN CDNA 5330426P16 GENE	0.04938672	0.8652812	0.3068621	0.866986475
1439379_x_at	PRM1	PROTAMINE 1	0.04939434	0.1345723	0.1793334	2.791033024
1447392_s_at	CPD	CARBOXYPEPTIDASE D	0.04941198	0.01261584	0.4025655	1.154921776
1428057_a_at	AHNAK	AHNAK NUCLEOPROTEIN (DESMOYOKIN)	0.04944526	0.8237613	0.5122049	0.464858293
1447578_at	1700084J12RIK	RIKEN CDNA 1700084J12 GENE	0.04944995	0.3736071	0.08629759	0.553343262
1451971_at	CUL4A	RIKEN CDNA 2810470J21 GENE	0.04949131	0.01932693	0.9161423	0.866989789
1418164_at	EPIM	EPIMORPHIN	0.04953017	0.2798975	0.3302909	0.865720462
1450867_at	MRPL17	MITOCHONDRIAL RIBOSOMAL PROTEIN L17	0.04953106	0.5281225	0.15347259	0.862239186
1424213_at	UBIAD1	UBIA PRENYLTRANSFERASE DOMAIN CONTAINING 1	0.04955903	0.5533272	0.2124688	0.917210009
1438402_at	9630050M13RIK	RIKEN CDNA 9630050M13 GENE	0.04957938	0.05850923	0.2357729	1.142046982
1441416_at	INPP4B	INOSITOL POLYPHOSPHATE-4-PHOSPHATASE, TYPE II	0.04964201	0.5307435	0.6863671	0.603174976
1430566_at	4733401A01RIK	RIKEN CDNA 4733401A01 GENE	0.04968408	0.09466921	0.1454461	1.226113081
1418758_a_at	PSCD3	PLECKSTRIN HOMOLOGY, SEC7 AND COILED-COIL DOMAINS 3	0.04970199	0.03614459	0.9154211	0.841691319
1423433_at	TROVE2	TROVE DOMAIN FAMILY, MEMBER 2	0.04971025	0.007393724	0.8310785	1.373310808
1443719_x_at	DDX42	DEAD (ASP-GLU-ALA-ASP) BOX POLYPEPTIDE 42	0.04971952	0.004178711	0.9446287	1.361790863
1428727_at	4631422C13RIK	RIKEN CDNA 4631422C13 GENE	0.04973459	0.8813748	0.7875255	0.822474638
1437782_at	Cntnap2	contactin associated protein-like 2	0.04974123	0.3702485	0.8218268	0.725474645
1457531_a_at	A930017K11RIK	RIKEN CDNA A930017K11 GENE	0.04986262	0.06084651	0.8480383	0.832359182
1440412_at	IGSF4D	IMMUNOGLOBULIN SUPERFAMILY, MEMBER 4	0.04989325	0.1193036	0.7483153	1.545891783
1442358_at	AA409587	EXPRESSED SEQUENCE AA409587	0.04992148	0.001855428	0.06778104	0.893667064
1440969_at	BC030308	CDNA SEQUENCE BC030308	0.04997339	0.4522375	0.3780786	1.150506178
1421243_at	RNF144	RING FINGER PROTEIN 144	0.04999526	0.1684724	0.3477377	1.71022664
1445625_at	---	---	0.0499993	0.4762303	0.3682522	0.552382372

Table S2

Term	Count	Percentage	P-value
Biological Process			
cellular physiological process	852	42.58%	1.14E-11
development	213	10.64%	1.63E-07
protein modification	166	8.30%	3.14E-07
biopolymer modification	170	8.50%	5.21E-07
intracellular signaling cascade	112	5.60%	9.73E-07
protein amino acid phosphorylation	79	3.95%	1.26E-06
primary metabolism	572	28.59%	1.65E-06
biopolymer metabolism	247	12.34%	3.72E-06
cell adhesion	74	3.70%	4.97E-06
metabolism	630	31.48%	5.56E-06
protein metabolism	280	13.99%	3.23E-05
macromolecule metabolism	360	17.99%	3.45E-05
metal ion transport	52	2.60%	3.55E-05
locomotion	41	2.05%	5.34E-05
cellular morphogenesis	52	2.60%	8.12E-05
localization of cell	40	2.00%	8.33E-05
cell motility	40	2.00%	8.33E-05
cellular metabolism	580	28.99%	1.23E-04
cellular protein metabolism	261	13.04%	1.33E-04
cellular macromolecule metabolism	264	13.19%	1.38E-04
phosphorylation	80	4.00%	1.54E-04
localization	280	13.99%	2.16E-04
ion transport	81	4.05%	2.26E-04
establishment of localization	277	13.84%	2.76E-04
morphogenesis	99	4.95%	2.97E-04
phosphorus metabolism	91	4.55%	3.01E-04
phosphate metabolism	91	4.55%	3.01E-04
behavior	36	1.80%	3.74E-04
vasculature development	26	1.30%	4.62E-04
neuropeptide signaling pathway	18	0.90%	4.65E-04
cellular process	1010	50.47%	5.21E-04
axonogenesis	22	1.10%	6.00E-04
axon guidance	15	0.75%	7.11E-04
second-messenger-mediated signaling	19	0.95%	7.39E-04
cell migration	32	1.60%	8.93E-04
phosphoinositide-mediated signaling	10	0.50%	0.001216148
cation transport	57	2.85%	0.001340741
nervous system development	57	2.85%	0.001840581
system development	61	3.05%	0.001851433
blood vessel development	24	1.20%	0.001858189
positive regulation of lyase activity	6	0.30%	0.001965128
adenylate cyclase activation	6	0.30%	0.001965128
positive regulation of cyclase activity	6	0.30%	0.001965128
regulation of adenylate cyclase activity	6	0.30%	0.001965128
positive regulation of adenylate cyclase activity	6	0.30%	0.001965128
cell differentiation	92	4.60%	0.002102036
locomotory behavior	24	1.20%	0.002588194
regulation of biological process	298	14.89%	0.002813205
blood vessel morphogenesis	21	1.05%	0.003406136
cell ion homeostasis	17	0.85%	0.003609544
potassium ion transport	23	1.15%	0.003718339
positive regulation of biological process	72	3.60%	0.003957223
regulation of cyclase activity	6	0.30%	0.00403197
regulation of lyase activity	6	0.30%	0.00403197
cation homeostasis	16	0.80%	0.004394293
synaptic transmission	22	1.10%	0.004928679
neuron morphogenesis during differentiation	22	1.10%	0.005325219
neurite morphogenesis	22	1.10%	0.005325219
cellular morphogenesis during differentiation	24	1.20%	0.005533013
metal ion homeostasis	15	0.75%	0.005938089
positive regulation of cell differentiation	11	0.55%	0.00613525
G-protein signaling, coupled to IP3 second messenger (phospholipase C activa	8	0.40%	0.006346802
organ development	93	4.65%	0.00640895
glycoprotein metabolism	17	0.85%	0.007055098
glutamine metabolism	5	0.25%	0.00720438
endothelial cell differentiation	5	0.25%	0.00720438
positive regulation of positive chemotaxis	4	0.20%	0.007439389
induction of positive chemotaxis	4	0.20%	0.007439389
regulation of chemotaxis	4	0.20%	0.007439389
positive regulation of chemotaxis	4	0.20%	0.007439389
regulation of positive chemotaxis	4	0.20%	0.007439389
positive chemotaxis	4	0.20%	0.007439389
transmission of nerve impulse	24	1.20%	0.00836101
cell homeostasis	19	0.95%	0.008425675
ion homeostasis	18	0.90%	0.008816173
cell development	36	1.80%	0.009520367
feeding behavior	7	0.35%	0.009794733
cell-cell signaling	32	1.60%	0.010296953
glycoprotein biosynthesis	15	0.75%	0.010658793
regulation of development	24	1.20%	0.011532122
angiogenesis	17	0.85%	0.011771439
protein amino acid glycosylation	14	0.70%	0.011948245
sensory perception of pain	5	0.25%	0.01380451
transport	239	11.94%	0.014129248
G-protein signaling, adenylate cyclase activating pathway	7	0.35%	0.014370585
positive regulation of enzyme activity	16	0.80%	0.014507494
positive regulation of development	12	0.60%	0.014830012
neuron development	24	1.20%	0.016541785
biopolymer glycosylation	14	0.70%	0.018733563
regulation of cell differentiation	19	0.95%	0.018787525
regulation of enzyme activity	23	1.15%	0.020087568
glutamine family amino acid metabolism	7	0.35%	0.020253167
di-, tri-valent inorganic cation homeostasis	13	0.65%	0.021255789
regulation of cell shape	9	0.45%	0.024536457
monovalent inorganic cation transport	34	1.70%	0.025323587
muscle development	15	0.75%	0.028398115
regulation of cellular process	266	13.29%	0.029469295
L-glutamate transport	3	0.15%	0.031470461
determination of left/right symmetry	6	0.30%	0.032228758
calcium ion homeostasis	9	0.45%	0.034300203
positive regulation of myeloid cell differentiation	4	0.20%	0.035439407
L-amino acid transport	5	0.25%	0.036021384
determination of symmetry	6	0.30%	0.037837265
determination of bilateral symmetry	6	0.30%	0.037837265
muscle contraction	11	0.55%	0.038982737
organ morphogenesis	49	2.45%	0.040427467
homeostasis	24	1.20%	0.042172623
cAMP-mediated signaling	8	0.40%	0.0474288
regulation of physiological process	260	12.99%	0.048956065
cell-cell adhesion	20	1.00%	0.049323667
glycine catabolism	3	0.15%	0.049822082
acidic amino acid transport	3	0.15%	0.049822082
dendritic cell differentiation	3	0.15%	0.049822082
dendritic cell activation	3	0.15%	0.049822082

Term	Count	Percentage	P-value
Cellular component			
intracellular	645	32.23%	5.92E-06
chromatin	24	1.20%	2.83E-04
nucleus	325	16.24%	8.29E-04
membrane fraction	46	2.30%	0.002597032
chromosome	36	1.80%	0.002609631
cell fraction	49	2.45%	0.005004769
intracellular organelle	530	26.49%	0.005196371
organelle	530	26.49%	0.005907698
apicolateral plasma membrane	14	0.70%	0.006199355
apical junction complex	14	0.70%	0.006199355
intracellular membrane-bound organelle	465	23.24%	0.010257364
membrane-bound organelle	465	23.24%	0.011447643
microtubule cytoskeleton	32	1.60%	0.011999726
extracellular matrix	32	1.60%	0.015276231
nuclear envelope-endoplasmic reticulum network	13	0.65%	0.015501046
cytoplasm	290	14.49%	0.021446215
cell junction	22	1.10%	0.026821266
endoplasmic reticulum membrane	12	0.60%	0.02776193
synapse	19	0.95%	0.029746707
intercellular junction	18	0.90%	0.03254508
extracellular matrix (sensu Metazoa)	30	1.50%	0.034195217
endomembrane system	33	1.65%	0.03604724
tight junction	10	0.50%	0.047964206

Term	Count	Percentage	P-value
Molecular function			
metal ion binding	332	16.59%	3.19E-12
ion binding	332	16.59%	3.19E-12
binding	922	46.08%	1.57E-11
protein binding	401	20.04%	1.59E-11
cation binding	307	15.34%	5.50E-11
calcium ion binding	107	5.35%	3.19E-09
nucleotide binding	200	10.00%	5.45E-09
purine nucleotide binding	178	8.90%	2.01E-08
catalytic activity	482	24.09%	3.18E-08
adenyl nucleotide binding	149	7.45%	4.69E-08
protein serine/threonine kinase activity	71	3.55%	6.81E-08
phosphotransferase activity, alcohol group as acceptor	93	4.65%	7.95E-08
ATP binding	144	7.20%	9.01E-08
protein kinase activity	83	4.15%	9.42E-08
transferase activity	193	9.65%	1.71E-07
kinase activity	111	5.55%	5.42E-07
protein-tyrosine kinase activity	50	2.50%	2.14E-06
ion channel activity	51	2.55%	5.88E-06
transferase activity, transferring phosphorus-containing groups	118	5.90%	8.96E-06
GTPase regulator activity	41	2.05%	1.50E-05
MAP kinase activity	8	0.40%	2.22E-05
alpha-type channel activity	51	2.55%	5.91E-05
channel or pore class transporter activity	51	2.55%	2.18E-04
voltage-gated ion channel activity	26	1.30%	2.30E-04
small GTPase regulator activity	27	1.35%	5.06E-04
GTPase activity	21	1.05%	5.69E-04
enzyme activator activity	27	1.35%	7.11E-04
enzyme regulator activity	67	3.35%	8.41E-04
GTPase activator activity	21	1.05%	0.001155503
identical protein binding	23	1.15%	0.001159824
growth factor binding	11	0.55%	0.001166443
transition metal ion binding	190	9.50%	0.001706054
protein dimerization activity	23	1.15%	0.00312798
zinc ion binding	155	7.75%	0.003208255
hydrolase activity	190	9.50%	0.004106437
nucleic acid binding	268	13.39%	0.004154899
guanyl-nucleotide exchange factor activity	18	0.90%	0.004419441
UDP-galactose:beta-N-acetylglucosamine beta-1,3-galactosyltransferase activi	4	0.20%	0.006391092
calmodulin binding	16	0.80%	0.007081357
ion transporter activity	72	3.60%	0.007135401
alkali metal ion binding	21	1.05%	0.0077174
kinase regulator activity	13	0.65%	0.0080787
cAMP-dependent protein kinase activity	21	1.05%	0.010154351
cyclic nucleotide-dependent protein kinase activity	21	1.05%	0.010154351
beta-1,3-galactosyltransferase activity	4	0.20%	0.010586056
protein kinase CK2 activity	21	1.05%	0.010851123
transcription factor binding	23	1.15%	0.01295359
insulin-like growth factor binding	6	0.30%	0.015050779
protein homodimerization activity	12	0.60%	0.016871587
potassium ion binding	14	0.70%	0.017554617
galactosyltransferase activity	7	0.35%	0.021805542
cytoskeletal protein binding	35	1.75%	0.023601126
transferase activity, transferring glycosyl groups	26	1.30%	0.024534485
transferase activity, transferring one-carbon groups	20	1.00%	0.025078598
neurotransmitter receptor activity	14	0.70%	0.027898329
neurotransmitter binding	14	0.70%	0.027898329
RNA binding	48	2.40%	0.029031634
hydrolase activity, acting on acid anhydrides, in phosphorus-containing anhydri	49	2.45%	0.030172284
hydrolase activity, acting on acid anhydrides	49	2.45%	0.03338244
DNA binding	160	8.00%	0.034334446
transferase activity, transferring hexosyl groups	18	0.90%	0.036821029
methyltransferase activity	19	0.95%	0.03882894
pyrophosphatase activity	48	2.40%	0.039010075
exonuclease activity	9	0.45%	0.039115199
transporter activity	130	6.50%	0.03939801
protein kinase regulator activity	10	0.50%	0.03986377
extracellular matrix structural constituent	10	0.50%	0.03986377
lipid binding	25	1.25%	0.041043297
aminomethyltransferase activity	3	0.15%	0.04511114
peptide YY receptor activity	3	0.15%	0.04511114
UDP-glycosyltransferase activity	12	0.60%	0.045306344
GTP binding	33	1.65%	0.046853032

Table S3

Donor ID	CNS-related illness	Death Cause	Gender	Age	Cluster
#1	NONE	PNEUMONIA	FEMALE	79	A
#2	NONE	PNEUMONIA	MALE	79	A
#3	NONE	MYOCARDIAL INFARCTION	MALE	90<	A
#4	NONE	CARDIAC ARREST	FEMALE	90<	A
#5	NONE	UNKNOWN	MALE	43	B
#6	NONE	CONGESTIVE CARDIOMYOPATHY	FEMALE	36	A
#7	NONE	CARDIORESPIRATORY FAILURE	FEMALE	90<	B
#8	NONE	CARDIORESPIRATORY FAILURE	FEMALE	90	B
#9	NONE	CARDIORESPIRATORY FAILURE	MALE	75	A
#10	NONE	MYOCARDIAL INFARCTION	FEMALE	85	B
#11	NONE	CARDIAC ARREST	FEMALE	86	A
#12	NONE	ACUTE RESPIRATORY INSUFFICIENCY	FEMALE	90<	A
#13	NONE	CHRONIC RENAL FAILURE	FEMALE	83	B
#14	NONE	CARDIAC ARREST	MALE	53	A
#15	NONE	LUNG CANCER	FEMALE	87	B
#16	NONE	MOTOR VEHICLE ACCIDENT	MALE	28	A
#17	NONE	MYOCARDIAL INFARCTION	MALE	41	A
#18	NONE	MOTOR VEHICLE ACCIDENT	MALE	31	A
#19	NONE	CARDIAC ARREST	MALE	27	A
#20	NONE	CHRONIC OBSTRUCTIVE LUNG DISEAS	FEMALE	87	A
#21	NONE	TRAUMA	MALE	29	A
#22	NONE	GUNSHOT WOUND TO CHEST	MALE	22	A
#23	NONE	MULTIPLE GUNSHOT WOUND	MALE	20	A
#24	NONE	MOTOR VEHICLE ACCIDENT	MALE	70	B
#25	NONE	CARDIAC ARREST	MALE	90<	B
#26	NONE	CARDIAC ARREST	FEMALE	89	B
#27	NONE	CARDIAC ARREST	MALE	66	B
#28	NONE	CARDIAC ARREST	MALE	69	B
#29	NONE	CARDIAC ARREST	MALE	74	B
#30	NONE	CARDIAC ARRHYTHMIA	MALE	39	A
#31	NONE	MULTIPLE GUNSHOT WOUND	MALE	20	A
#32	NONE	MALIGNANT MELANOMA	FEMALE	85	A
#33	NONE	ASTHMA	MALE	34	A
#34	NONE	CARDIAC ARRHYTHMIA	MALE	26	A
#35	NONE	GUNSHOT WOUND	FEMALE	24	A
#36	NONE	MYOCARDIAL INFARCTION	MALE	52	A
#37	NONE	MYOCARDIAL INFARCTION	MALE	41	A
#38	NONE	MYOCARDIAL INFARCTION	MALE	44	A
#39	NONE	CARDIAC ARREST	FEMALE	89	B
#40	NONE	CARDIAC ARREST	FEMALE	90<	A
#41	NONE	ACUTE RESPIRATORY INSUFFICIENCY	MALE	90	A
#42	NONE	MYELODYSPLASTIC SYNDROME	MALE	90	B
#43	NONE	CARDIAC ARREST	FEMALE	85	A
#44	NONE	CARDIAC ARREST	FEMALE	82	A
#45	Schizophrenia	CARDIORESPIRATORY FAILURE	MALE	84	B
#46	Schizophrenia	CARDIORESPIRATORY FAILURE	MALE	87	B
#47	Schizophrenia	CARDIORESPIRATORY FAILURE	MALE	73	B
#48	Schizophrenia	CARDIORESPIRATORY FAILURE	MALE	66	B
#49	Schizophrenia	CARDIORESPIRATORY FAILURE	MALE	90<	B
#50	Schizophrenia	CARDIORESPIRATORY FAILURE	MALE	66	B
#51	Schizophrenia	CARDIAC FAILURE	MALE	82	B
#52	Schizophrenia	CARDIORESPIRATORY FAILURE	FEMALE	74	B
#53	Schizophrenia	SUICIDE BY HANGING	FEMALE	39	A
#54	Schizophrenia	ACUTE RESPIRATORY INSUFFICIENCY	MALE	40	B
#55	Schizophrenia	CARDIORESPIRATORY FAILURE	MALE	79	B
#56	Schizophrenia	CORONARY ARTERY DISEASE	FEMALE	65	B
#57	Schizophrenia	CARDIAC ARREST	MALE	57	B
#58	Schizophrenia	CARDIORESPIRATORY FAILURE	MALE	82	B
#59	Schizophrenia	CARDIORESPIRATORY FAILURE	FEMALE	90	B
#60	Schizophrenia	SUICIDE	MALE	30	B
#61	Schizophrenia	CARDIORESPIRATORY FAILURE	MALE	73	B
#62	Schizophrenia	SUICIDE BY HANGING	MALE	29	A
#63	Schizoaffective disorder	SUICIDE	MALE	18	B
#64	Bipolar disorder	SUICIDE BY HANGING	MALE	34	B
#65	Bipolar disorder	SUICIDE BY DRUG OVERDOSE	MALE	24	B

Table S4

Mouse Affimetrix probes	Corresponding mouse genes	Human orthologues	Human Affimetrix probes	% P flag in 166 human hippocampal data in BioExpress	F value for specific 21 psychiatric patients
1454653_at	RIKEN cDNA A730016F12 gene	LOC151835	239139_at	92.17	11.816
1417420_at	cyclin D1	CCND1	208712_at	52.41	7.9104
1441634_at	netrin G1	NTNG1	236088_at	23.49	6.6809
1419093_at	tryptophan 2,3-dioxygenase	TDO2	205943_at	52.41	4.8376
1416266_at	prodynorphin	PDYN	206803_at	40.36	4.1403
1444298_at	RIKEN cDNA A130090K04 gene	PIP3-E	214735_at	75.3	4.0516
1422711_a_at	pregnancy upregulated non-ubiquitously expressed CaM kinase	PNCK	229411_at	28.92	3.7059
1418754_at	adenylate cyclase 8	ADCY8	206811_at	90.96	3.4637
1437865_at	spermatogenesis associated 13	SPATA13	225564_at	96.99	2.9952
1428809_at	RIKEN cDNA 1810010H24 gene	LOC284018	226901_at	87.35	2.7169

Table S5

A

						genotype[e]			P-value	
	SNP ID[a]	position[b]	MAF[c] (%)	phenotype[d]	Nnmber	M/M	M/m	m/m	genotype	allele
1	rs1432832	1	17.2	SCZ	376	201	145	30	0.507	0.344
				BP	115	70	38	7	0.759	0.451
				CON	347	200	120	27		
2	rs873593	4071	0.8							
3	rs919740	7693	41.4	SCZ	376	123	176	77	**0.0263**	**0.0321**
				BP	111	27	64	20	0.0524	**0.0243**
				CON	346	126	175	45		
4	rs1432833	12389	47.7							
5	rs9686770	15341	44.5	SCZ	377	133	168	76	0.789	0.526
				BP	115	36	60	19	0.445	0.694
				CON	347	114	160	73		
6	rs3797616)	20538	7.0							
7	rs874084	22712	29.7	SCZ	377	182	156	39	0.233	0.630
				BP	115	54	49	12	0.609	0.630
				CON	343	151	163	29		
8	rs2304042	28735	28.9	SCZ	376	192	150	34	0.574	0.847
				BP	115	46	57	12	0.141	0.0556
				CON	347	174	148	25		
9	rs3822606	32387	28.9							
10	rs4958445	35182	29.7							
11	rs3776824	36931	30.5	SCZ	378	178	154	46	0.774	0.544
				BP	115	59	46	10	0.798	0.502
				CON	343	166	141	36		
12	rs10463294	37433	0							
13	rs10463293	37469	39.8							
14	rs880100)	38185	0							
15	rs2288799	42129	36.7	SCZ	376	147	174	55	0.696	0.391
				BP	115	48	56	11	0.592	0.644
				CON	347	145	157	45		
16	rs3756577	44898	22.7	SCZ	378	215	140	23	0.180	0.0726
				BP	116	64	45	7	0.298	0.127
				CON	344	215	116	13		
17	rs4958454	46790	41.4	SCZ	379	132	173	74	0.415	0.796
				BP	105	39	44	22	0.297	0.774
				CON	344	109	174	61		
18	rs 869192	47931	35.2	SCZ	376	149	163	64	0.509	0.277
				BP	115	43	51	21	0.927	0.776
				CON	347	123	160	64		
19	rs10066581	57861	21	SCZ	376	236	117	23	**0.0213**	0.164
				BP	115	69	39	7	0.0817	0.155
				CON	347	224	116	7		
20	rs3822607	61749	40.6	SCZ	376	109	185	82	0.202	0.294
				BP	115	36	61	18	0.916	0.693
				CON	347	102	187	58		
21	rs2053053	64149	18.0	SCZ	376	274	89	13	0.275	0.555
				BP	115	75	33	7	0.133	0.165
				CON	347	241	98	8		
22	rs7711408	692680	31.2	SCZ	376	182	156	38	0.204	0.507
				BP	115	46	52	17	**0.0195**	**0.0210**
				CON	347	167	157	23		
23	rs2241695	70718	32.8	SCZ	376	178	166	32	0.867	0.734
				BP	115	43	56	16	0.124	0.0558
				CON	347	158	160	29		
24	rs2241694	70934	13.3	SCZ	376	264	97	15	**0.0358**	**0.0303**
				BP	115	79	33	3	0.213	0.116
				CON	347	262	81	4		
25	rs957709	71716	16.4							
26	rs887346	77550	22.7	SCZ	376	179	160	37	0.305	0.134
				BP	115	46	54	15	**0.0331**	**0.00967**
				CON	347	181	141	25		

a. Gray box represents 'haplotype-tagging (ht) SNPs' for association analysis
b. NT029289.10
c. MAF=minor allele frequency of 64 controls
d. SCZ=schizophrenia, BP=bipolar disorder, CON=control
e. M=major allele, m=minor allele
f. Bold numbers represent signifincat P-values

B

		Global P-value[a]	
Block	Combination of htSNPs	SCZ	BP
II	SNP7-8	0.891	0.185
III	SNP11-15	0.791	0.344
IV	SNP17-18	0.287	0.461
V	SNP19-20	0.429	0.102
VI	SNP21-22-23-24	0.252	**0.0015**

a. Bold numbers represent signifincat P-values

C

			genotype			P-value	
SNPID	phenotype	Number	M/M	M/m	m/m	genotype	allele
3 rs919740	SCZ	367	108	204	55	0.455	0.871
	BP	149	43	80	26	0.860	0.745
	CON	382	119	196	67		
19 rs10066581	SCZ	357	224	117	16	0.273	0.293
	BP	149	95	50	4	0.140	0.117
	CON	380	225	128	27		
21 rs2053053	SCZ	367	268	95	4	0.211	0.294
	BP	149	93	54	2	0.0520	0.173
	CON	382	271	100	11		
22 rs7711408	SCZ	368	197	145	26	0.222	0.0878
	BP	149	66	69	14	0.631	0.589
	CON	381	184	159	38		
23 rs2241695	SCZ	367	172	164	31	0.110	0.169
	BP	149	69	65	15	0.609	0.486
	CON	378	170	158	50		
24 rs2241694	SCZ	366	282	75	9	0.0922	0.0529
	BP	149	112	29	8	0.0690	0.651
	CON	382	268	104	10		
26 rs887346	BP	149	69	64	16	0.772	0.946
	CON	370	176	148	46		

Supplementary Table Legends

Table S1. List of genes that were significantly upregulated or downregulated in the hippocampus of alpha-CaMKII+/- mice.

Table S2. Gene Ontology annotations of the genes that were significantly upregulated or downregulated in the hippocampus of alpha-CaMKII+/- mice.

Table S3. Donors offering hippocampi for GeneChip analysis. Expression data from total 166 hippocampi were used in BioExpress database including additional 98 donors with various CNS-related illness. A, Cluster A in Figure 5 (normal control-enriched cluster); B, Cluster B (schizophrenic psychiatric patient-enriched cluster).

Table S4. Affymetrix probes used as alpha-CaMKII+/- mice-specific hippocampal biomarkers. F value was calculated to segregate 21 schizophrenic or its related psychiatric patients shown in Additional Table 3 from the rest of 145 donors.

Table S5. Association analysis of alpha-CaMKII gene with schizophrenia and bipolar disorder. (A) Summary of haplotype-tagging single nucleotide polymorphisms (htSNPs) and association analysis of alpha-CaMKII in first-set samples. (B) Haplotype-wise analysis of first-set samples. (C) Summary of the second-set confirmation analysis of significant htSNPs in first-set samples.
